# Manipulations of phenylnorbornyl palladium species for multicomponent construction of a bridged polycyclic privileged scaffold

**DOI:** 10.1038/s42004-022-00759-4

**Published:** 2022-10-29

**Authors:** Lina Yin, Ting Guan, Jie Cheng, Dongchao Pan, Jinyang Lu, Jiahui Huang, Jiaqi Wu, Xiaoli Chen, Taiyun You, Xuting Huo, Yuting He, Jiayun Pang, Qingzhong Hu

**Affiliations:** 1 School of Pharmaceutical Sciences, Guangzhou University of Chinese Medicine, 232 East Waihuan Road, Panyu, 510006 Guangzhou, People’s Republic of China; 2 School of Science, Faculty of Engineering and Science, University of Greenwich Medway Campus, Central Avenue, Chatham Maritime, Chatham, ME4 3RL UK

**Keywords:** Synthetic chemistry methodology, Heterogeneous catalysis, Drug discovery and development

## Abstract

Hexahydromethanocarbazole is a privileged scaffold in the discovery of new drugs and photoactive organic materials due to its good balance between structural complexity and minimized entropy penalty upon receptor binding. To address the difficulty of synthesizing this highly desirable bridged polycyclic scaffold, we designed a convenient multicomponent reaction cascade as intercepted Heck addition/C-H activation/C-palladacycle formation/electrophilic attack of ANP/N-palladacycle formation/Buchwald amination. A distinguishing feature of this sophisticated strategy is the successive generation of two key phenylnorbornyl palladium species to control the reaction flow towards desired products. DFT calculations further reveal the crucial roles of Cs_2_CO_3_ and 5,6-diester substitutions on the norbornene reactant in preventing multiple side-reactions. This innovative method exhibits a broad scope with good yields, and therefore will enable the construction of natural-product-like compound libraries based on hexahydromethanocarbazole.

## Abstract (web summary)

Hexahydromethanocarbazole is a bridged polycyclic scaffold present in drugs and photoactive organic materials, however the efficient synthesis of this scaffold remains challenging. Here, the authors develop a multicomponent reaction cascade via phenylnorbornyl palladium species to generate hexahydromethanocarbazole-based libraries with CYP11B1 inhibition activity.

## Introduction

**Fig. 1:**
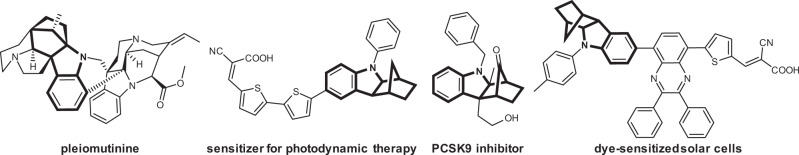
Presence of Hexahydromethanocarbazole. Hexahydromethanocarbazole moieties present in natural products, materials, solar cells, and potential therapeutic compounds.

In contrast to the immense chemical space, only limited molecular structural scaffolds have been explored for drug discovery until now. These scaffolds are largely characterized by flat conformations^1,2^, which, unfortunately, are frequently associated with attritions in the subsequent stages of drug development^3^. To solve this problem, increasing the number of *sp*^3^-hybridized carbons within a molecule has been recommended^4^. Through such a strategy, the resulting compounds are more “natural-product-like” and exhibit increased topological diversity and structural complexity, and hence could enable the probe of deeper and wider chemical space for biologically relevant molecular designs. Moreover, the abundance of *sp*^3^-hybridized carbons could lead to increased aqueous solubility via interfering crystal packing, reduced plasma protein binding, and thus could potentially improve the pharmacokinetic properties of drug candidates^5^. However, *sp*^3^-hybridized carbons tend to increase open-chained moieties’ flexibilities, which usually account for the entropy penalty upon binding of small-molecule ligands to their protein targets^6^. To counter this, cyclization of open-chained moieties will lead to saturated polycyclic skeletons with limited bond rotations; while insertion of an additional bridge will further rigidify molecular configurations, and thus mitigate the loss in entropy and binding affinity. The bridged aza bi- or tri-cycloakane scaffolds, in particular hexahydromethanocarbazole (Fig. 1), are examples of a good balance between structural complexity arising from the abundance of *sp*^3^-hybridized carbons and minimized entropy penalty upon binding, and are therefore potential privileged structures in drug discovery. Unfortunately, the syntheses of such bridged polycyclic scaffolds frequently suffer from lengthy synthetic routes, complex or harsh reaction conditions and mixtures of stereoisomers as a result.

**Fig. 2:**
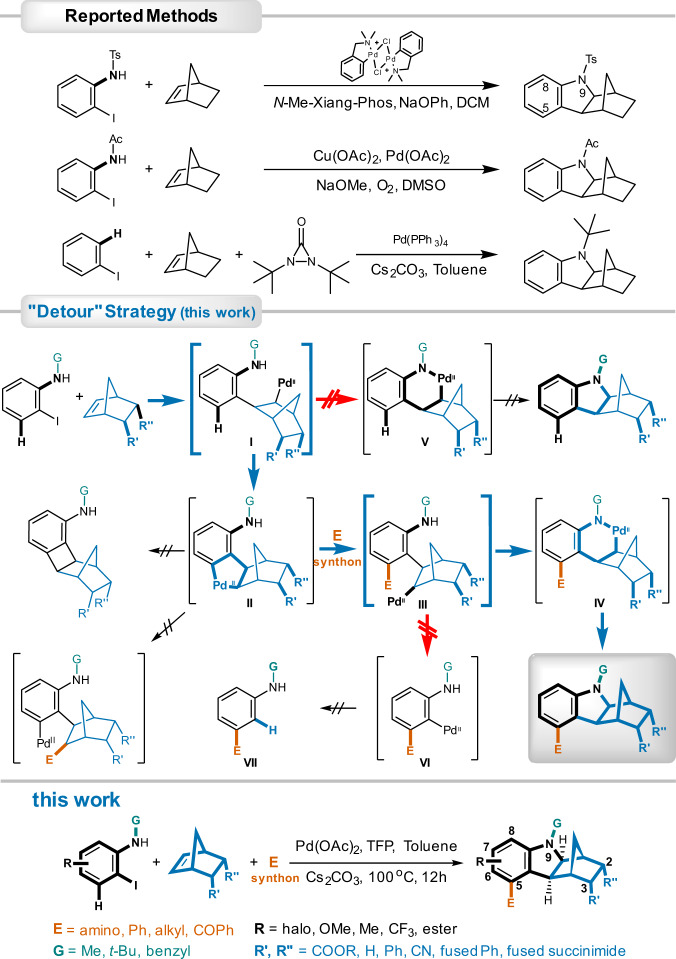
Strategies and methods in synthesizing hexahydro-1,4-methanocarbazole ring. Traditional methods involved palladium-catalyzed direct condensation, or electrophilic insertion by external amine surrogates. In contrast, “detour” strategy in this work employed successive generation of two key phenylnorbornyl palladium species to construct the hexahydro-1,4-methanocarbazole core and introduce 5-substitution in tandem. Ligands to Pd^II^, and electrophile-Pd^IV^ complexes were omitted when illustrating for the sake of clarity.

Hexahydromethanocarbazole is present in natural products including pleiomutinune^7^, and has been exploited as an essential pharmacophore in drug discovery against hyperlipidemia (PCSK9 inhibitors)^8^ (Fig. 1). Beyond that, due to its electron-donating nature, hexahydromethanocarbazole is also used in constructing D–π–A dyes as sensitizers for photodynamic anti-cancer therapies^9^ and solar cells^10^. Furthermore, based on the norbornene moiety of hexahydromethanocarbazole, it has the potential to be used in high-density fuels^11^, photoclick hydrogels for tissue engineering^12^, and membranes for targeted gas separations^13^. However, this highly desirable bridged aza-polycyclic core is far from being fully exploited as a privileged scaffold in these fields, partly due to its poor synthetic accessibility, especially when multi-substituted. In previously reported methods, two strategies have been employed for the synthesis of hexahydro-1*H*-1,4-methanocarbazole (Fig. 2), either by a palladium-catalyzed direct condensation between 2-iodoaniline and norbornene (NBE)^14–16^; or via an *ortho* C-H activation followed by an electrophilic attack by an external amine surrogate^17–19^. Unfortunately, these methods are not effective with regard to the synthesis of compounds with structural complexity, in particular 5-substituted analogs, with the 5-substituents mostly being the simple methyl group among the few examples that have been reported. This is because when adopting the existing synthetic methods, such a substituent would have to be pre-installed *ortho* to iodine in the starting materials, which are usually difficult to obtain both commercially and through in-house synthesis. Amine surrogates like diaziridinones are not readily available either. More importantly, there are potential adverse impacts on the oxidative addition of iodide to Pd(0) and NBE migratory insertion, as well as the risk of inducing NBE extrusion, if the pre-installed *ortho*-substituents are bulky. Strong bases like NaOPh and NaOMe that are necessary in most cases may not be compatible with sensitive groups as well.

We encountered the aforementioned difficulties and problems in our pursuit of the 5-substituted hexahydro-1*H*-1,4-methanocarbazoles as CYP11B1 inhibitors^20^. Since CYP11B1 is a crucial enzyme in the biosynthesis of the glucocorticoid cortisol, such an inhibition would reduce abnormally high levels of cortisol in plasma and tissues, and thus constituted a promising therapy for related severe diseases such as Cushing’s syndrome and diabetic foot ulcer. To address the synthetic problems, we resolved to develop a novel strategy to effectively synthesize the target molecules through manipulating phenylnorbornyl palladium species in a multicomponent reaction approach. Herein, we report the convenient synthesis of 52 of such natural-product-like molecules with structural complexity and diversity using this new strategy. We further report mechanistic insights into how the desired main synthetic route competes with possible side reactions based on density functional theory (DFT)-based calculations. This innovative method exhibits a broad scope to various reactants and will enable a wide range of multi-substituted analogs of hexahydromethanocarbazole to be explored for drug design, therapeutic interventions, and the development of advanced energy materials.

## Results

### Design concept

Our synthetic strategy was inspired by the intriguing reactive properties of the phenylnorbornyl palladium species exhibited in the Catellani-Lautens reactions^21–25^, where it mediated the *ortho*-substitution via activating the adjacent C(*sp*^2^)-H bonds. The subsequent elimination of the NBE moiety facilitated a nucleophilic *ipso*-substitution. With such a superiority of disubstitution in one-pot, the Catellani-Lautens reactions were widely employed in the synthesis of various type of chemicals^26–30^, in particular, natural products^31–33^. The use of analogous norbornadiene, in contrast, triggered retro-Diels-Alder and thus led to convenient synthesis of indoles^34^.

In the current work, two phenylnorbornyl palladium species (**I** & **III**) generated successively were exploited as switches to control the reaction flow towards the desired destiny via C- and N- aryl-NBE palladacycle (ANP) **II** and **IV**, respectively (Fig. 2). The initial key intermediate **I** was obtained from the migratory insertion by a modified NBE to the oxidative adduct of Pd(0) and an easily available iodoaniline as a staring material. Rather than forming the six-membered N-Pd^II^ coordinating palladacycle **V** (a possible mechanistic route of the reported direct condensation methods), the phenylnorbornyl palladium complex **I** was modulated into C-ANP **II** via *ortho* C-H activation. Subsequent oxidative addition and reductive elimination upon the ANP **II** by a proper electrophile agent not only introduced an *ortho* substituent into the molecule, but also brought the intermediate back to a second phenylnorbornyl palladium species **III**, which enabled the options of NBE elimination or sustainment within the compound. By avoiding NBE extrusion (a unique feature of the Catellani-Lautens reaction) from complex **III**, the reaction was guided into the construction of the *ortho* substituted six-membered N-Pd^II^ coordinating palladacycle **IV**. Consequent reductive elimination of the palladacycle **IV** yielded the ultimate product 5-substituted hexahydro-1*H*-1,4-methanocarbazoles. Apparently, competitions at each diverging point (i.e., phenylnorbornyl palladium species **I** & **III**) determined the destiny of this reaction. Some potential side reactions may occur, such as prematurely elimination of the ANP **II** to norbornyl benzocyclobutene, as well as migration of the electrophile to the norbornyl instead of the phenyl (Fig. 2). However, despite of these challenges, we achieved this sophisticated synthesis of 5-substituted hexahydro-1*H*-1,4-methanocarbazoles by means of modifications on NBE reactants, deliberate choices of proper electrophile and extensive optimizations of reaction conditions.

### Initial attempts and optimizations

**Fig. 3:**
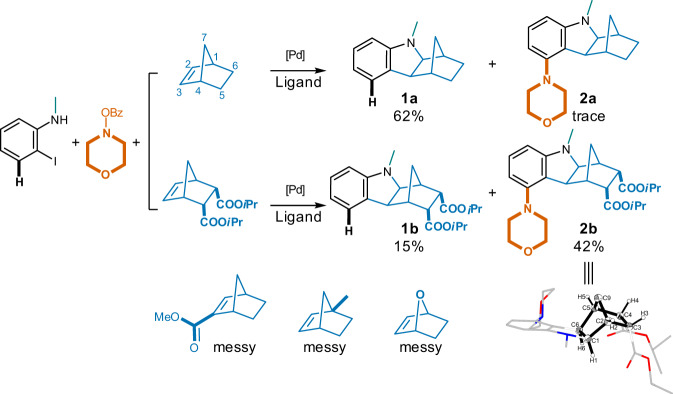
Initial investigations on NBE analogs capable of *ortho* C(sp^2^)-H activation to synthesize the 5-substituted hexahydro-1*H*-1,4-methanocarbazoles. Reaction conditions: 2-iodo-*N*-methylaniline (1.0 eq.), NBE analog (1.0 eq.), morpholino benzoate (1.3 eq.), Pd(OAc)_2_ (5% eq.), PPh_3_ (10% eq.), NaO*t*Bu (2.5 eq.), acetonitrile, 100 ^o^C, 12 h.

Since modifications on NBE would significantly influence its reactive and catalytic behavior^35^, five NBE analogs with distinct substitution patterns (Fig. 3) were first attempted. 2-Iodo-*N*-methylaniline (1 eq.) and morpholino benzoate (1.3 eq.) were employed as the reactants in the presence of Pd(OAc)_2_ (5% eq.), PPh_3_ (10% eq.) and NaO*t*Bu (2.5 eq.). It turned out that unmodified NBE yielded predominately unsubstituted methanocarbazole **1a** (62%); while analogs with 7-oxa, or groups on the bridge-head (1-Me) or the double bond (2-COOMe) led to messy results (Fig. 3). In contrast, NBE with 5,6-di(isopropyl carboxylate) substitution^36^ gave the desired 5-morpholino hexahydro-1,4-methanocarbazole **2b** in a moderate yield (42%, Fig. 3) together with minor amount of unsubstituted methanocarbazole (**1b**, 15%). Intriguingly, compound **2b** was obtained as a single stereoisomer, and its crystal structure (CCDC 2121980 (Deposition Number CCDC 2121980 contains the supplementary crystallographic data for this paper. These data are provided free of charge by the joint Cambridge Crystallographic Data Centre and Fachinformationszentrum Karlsruhe Access Structures service), Supplementary Data 1, Fig. 3) indicated an exo- cis- configuration for its rigid and somewhat strained scaffold. This is probably caused by the fact that the *Re* face is sterically much less hindered compare to the opposite *Si* face, and the reacting partners therefore approach predominantly from the *Re* face^37^.

Encouraged by this observation, the reaction conditions were further optimized using 5,6-diCOO*i*Pr NBE as the template as well as various Pd (pre)catalysts, ligands, bases and solvents (Table 1). Replacement of NaO*t*Bu with a milder base Cs_2_CO_3_ gave a similar yield of the desired **2a** but suppressed the production of the unsubstituted methanocarbazole by approximately fivefold, while simultaneous alteration of the solvent from acetonitrile to toluene increased the yield of the 5-morpholino product to 59% (Table 1, entries 1 & 2). However, other bases such as KOAc and K_3_PO_4_ did not exhibit many advantages in distinguishing 5-morpholino and the unsubstituted products. Moreover, the dicyclohexylphosphino ligands such as RuPhos, DavePhos, and XPhos were ineffective in constructing the methanocarbazole core, whereas tri(o-furyl)phosphine (TFP) doubled the yield of the 5-morpholino compound **2b** to 93% when compared to PPh_3_, and gave only trace of the unsubstituted by-product **1b** (Table 1, entry 5). When Pd_2_(dba)_3_, Pd(PPh_3_)_4_ or Pd(dppf)Cl_2_ was employed instead of the precatalyst Pd(OAc)_2_ or PdCl_2_, similar amounts of 5-morpholino and the unsubstituted compounds were formed in yields of ~35%. Finally, a significant influence of the solvent used was observed in that THF led to low production of the methanocarbazole scaffold, in contrast to ~70% yields of the 5-morpholino analog **2b** accompanied by a trace of the by-product in DMF or DME (Table 1, entries 10–12). Taking all aspects investigated into consideration, the optimal synthetic condition was determined to be Pd(OAc)_2_ (5% eq.), TFP (10% eq.) and Cs_2_CO_3_ (2.5 eq.) in toluene heating at 100 ^o^C for 12 h.

**Table 1:** Selected attempts in condition optimizations^a^.


No.	[Pd]	Ligand	Base	Solvent	Yield 2b (%)	Yield 1b (%)
1	Pd(OAc)_2_	PPh_3_	Cs_2_CO_3_	MeCN	48	3
2	Pd(OAc)_2_	PPh_3_	Cs_2_CO_3_	Toluene	59	6
3	Pd(OAc)_2_	PPh_3_	KOAC	Toluene	37	36
4	Pd(OAc)_2_	PPh_3_	K_3_PO_4_	Toluene	45	16
5	Pd(OAc)_2_	TFP	Cs_2_CO_3_	Toluene	93	Trace
6	Pd(OAc)_2_	RuPhos	Cs_2_CO_3_	Toluene	9	28
7	Pd_2_(dba)_3_	TFP	Cs_2_CO_3_	Toluene	35	31
8	Pd(PPh_3_)_4_	—	Cs_2_CO_3_	Toluene	32	38
9	Pd(dppf)Cl_2_	—	Cs_2_CO_3_	Toluene	37	24
10	Pd(OAc)_2_	TFP	Cs_2_CO_3_	THF	19	23
11	Pd(OAc)_2_	TFP	Cs_2_CO_3_	DMF	69	Trace
12	Pd(OAc)_2_	TFP	Cs_2_CO_3_	DME	75	Trace

^a^Unless indicated otherwise, 2-iodo-*N*-methylaniline (1.0 eq.), NBE-5,6-diCOO*i*Pr (1.0 eq.), morpholino benzoate (1.3 eq.), Pd (pre)catalyst (5% eq.), ligand (10% eq.), base (2.5 eq.), 100 ^o^C, 12 h.

### Reaction scopes

**Fig. 4:**
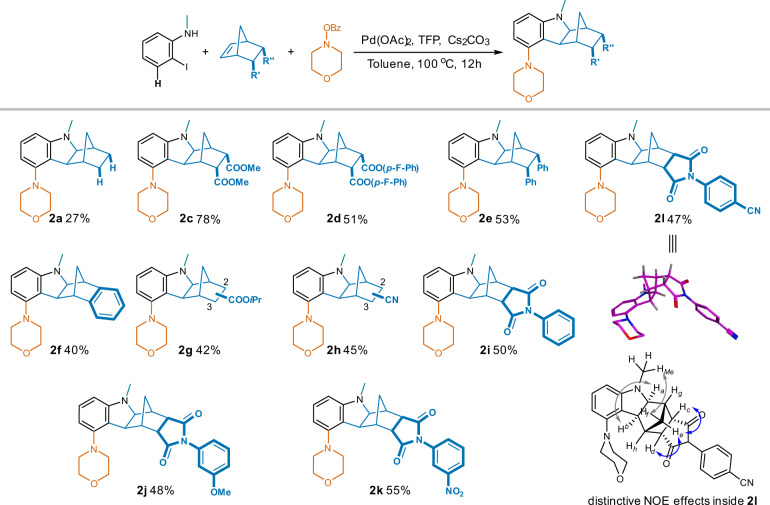
Influences of NBE substitutions in synthesizing the corresponding 5-substituted hexahydro-1*H*-1,4-methanocarbazoles. Reaction conditions: 2-iodo-*N*-methylaniline (1.0 eq.), NBE analog (1.0 eq.), morpholino benzoate (1.3 eq.), Pd(OAc)_2_ (5% eq.), TFP (10% eq.), Cs_2_CO_3_ (2.5 eq.), toluene, 100 ^o^C, 12 h.

Under this optimal condition, unsubstituted NBE gave the desired 5-morpholino compound **2a** in a significantly elevated yield of 27% compared to a trace under the initial condition, and the formation of **1a** (without 5-insertion) was suppressed by half to 35% at the meantime (Fig. 4). In contrast to the low yield from the unsubstituted NBE, the 5,6-dicarboxylic ester substitutions led to good to excellent yields of the 5-morpholino products (93% for isopropyl ester **2b**, 78% for methyl ester **2c**, and 51% for the much bulkier 4-fluorophenyl ester **2d**). Replacement of the carboxylic esters by 5,6-diphenyl (**2e**) or fused phenyl (**2f**) sustained adequate production of the desired 5-substituted analogs, indicating a broader scope of substitution choices in addition to the carbonyl containing groups in **2b**, **2c**, and **2d**. Different from the 5,6-disubstitution, the mono-substituted NBE starting materials resulted in mixtures of 2- and 3-substituted 5-morpholino hexahydro-1*H*-1,4- methanocarbazoles (**2g** and **2h**). The fused *N*-phenyl succinimides also led to their corresponding 5-morpholino products (**2i**-**2l**) in yields of ~50% regardless of the distinct stereoelectronic properties of substituents on *N*-phenyl. NOE effects among aliphatic protons in compound **2l** revealed an exo-configuration between the indoline and NBE moieties (in accordance with the crystal data of compound **2b**), however, an *endo*-configuration between the NBE and succinimide moieties (for configuration determination of compound **2l** using 2D-NMR spectra, see Supplementary Note 1).

**Fig. 5:**
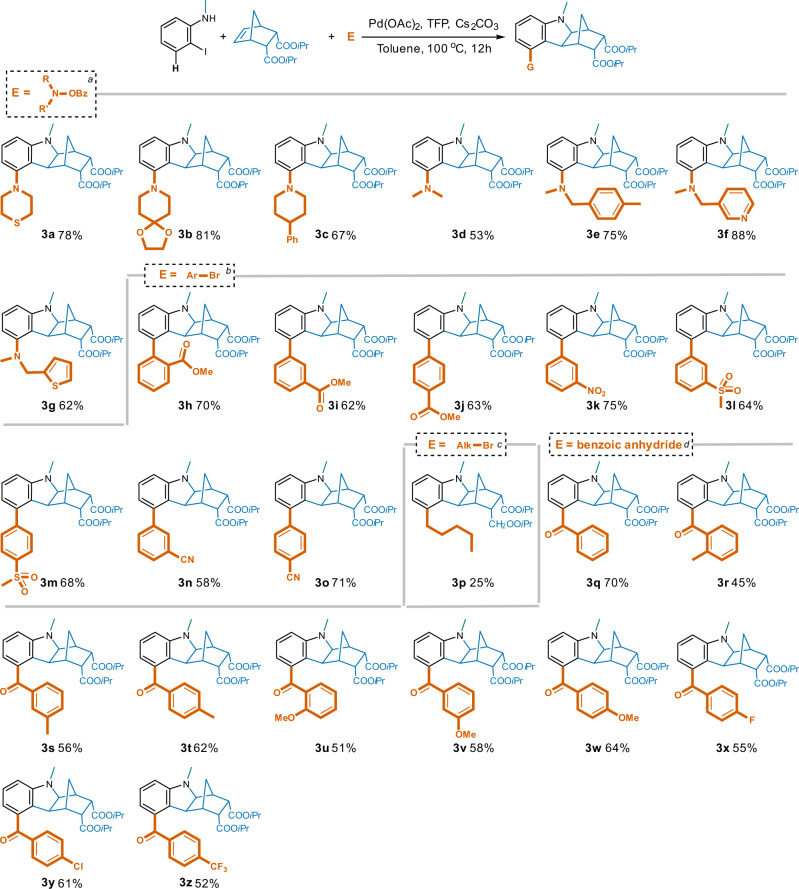
Generation of 5-substituted hexahydro-1*H*-1,4-methanocarbazoles with various electrophiles. Reaction conditions: ^[a]^2-iodo-*N*-methylaniline (1.0 eq.), NBE-5,6-diCOO*i*Pr (1.0 eq.), *N*-benzoyloxyamine (1.3 eq.), Pd(OAc)_2_ (5% eq.), TFP (10% eq.), Cs_2_CO_3_ (2.5 eq.), toluene, 100 ^o^C, 12 h; ^[b]^arylbromide (1.3 eq.), and DMF were used instead; ^[c]^*n-*pentylbromide (1.3 eq.), PPh_3_ (10% eq.), and DMF were used instead; ^[d]^ benzoic anhydride (1.3 eq.), PdCl_2_ (5% eq.), and DME were used instead.

To further investigate the scope of this reaction, various types of electrophiles were employed, and broad tolerance was demonstrated (Fig. 5). When using *N*-benzoyloxyamines to introduce amino groups (amination)^38,39^ at the 5-position, morpholino (**2b**), thiomorpholino (**3a**), and the simpler dimethylamino (**3d**), as well as the more structurally complex and bulkier analogs (**3b**, **3d** and **3f**–**3g**) were all successful with good to excellent yields. Similarly, aryl bromides bearing electron- withdrawn groups like cyano, nitro, methoxycarbonyl, and methylsulfonyl led to the desired 5-aryl hexahydro-1*H*-1,4-methanocarbazoles (**3h**–**3o**) in polar solvent DMF with good yields. Potential steric hindrance induced by *ortho*-substitution was overcome as well (**3** **h**, *o*-COOMe, 70% yield). By contrast, the introduction of an alkyl group or an aryl with electron-donating moiety was more challenging typically resulting in only a trace of the desired products. After further condition optimizations, 5-*n-* pentyl compound (**3p**) was isolated in 25% yield from DMF with PPh_3_ as the ligand. Nevertheless, introduction of the 5-benzoyl moieties (acylation)^40,41^ (**3q**–**3z**) using their corresponding benzoic anhydrides took place smoothly irrespective of the electronic properties and substitution patterns in good yields ranging between 45% and 70% catalyzed by PdCl_2_ in DME.

**Fig. 6:**
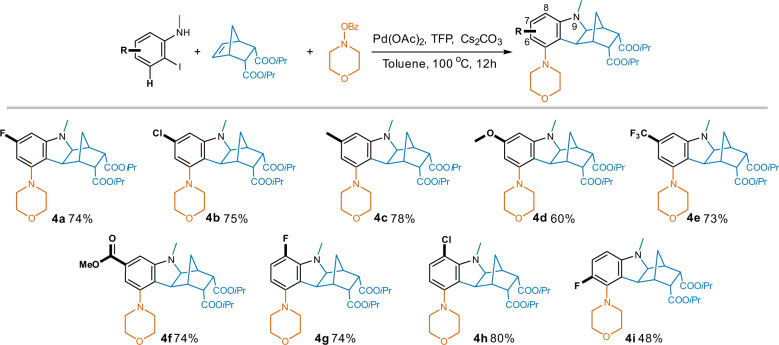
Compatibility of substituents on 2-iodo-*N*-methylaniline. Reaction conditions: substituted 2-iodo-*N*-methylaniline (1.0 eq.), NBE-5,6-diCOO*i*Pr (1.0 eq.), morpholino benzoate (1.3 eq.), Pd(OAc)_2_ (5% eq.), TFP (10% eq.), Cs_2_CO_3_ (2.5 eq.), toluene, 100 ^o^C, 12 h.

The high tolerance of this reaction to groups on 2-iodo-*N*-methylaniline reactants was shown in Fig. 6. Both electron-withdrawn (halogen, CF_3_ and COOMe) and electron-donating (Me and OMe) groups at the 7-position (*meta*- to morpholino) led to yields in the range of 60% to 78% (compounds **4a**–**4f**). There is a slight decrease in yield for the 6-F (*ortho*- to morpholino) analog **4i** (48%), probably due to a potential steric hindrance; while the 8-halo (*para*- to morpholino) compounds **4g** and **4h** were obtained in good yields (74% and 80%, respectively).

The high tolerance of this reaction to groups on 2-iodo-*N*-methylaniline reactants was shown in Fig. 6. Both electron-withdrawn (halogen, CF_3_ and COOMe) and electron-donating (Me and OMe) groups at the 7-position (*meta*- to morpholino) led to yields in the range of 60% to 78% (compounds **4a**–**4f**). There is a slight decrease in yield for the 6-F (*ortho*- to morpholino) analog **4i** (48%), probably due to a potential steric hindrance; while the 8-halo (*para*- to morpholino) compounds **4g** and **4h** were obtained in good yields (74% and 80%, respectively).

**Fig. 7:**
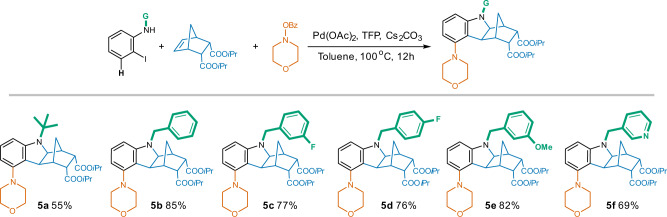
Influence of *N*-substituents on the production of 5-substituted hexahydro-1*H*-1,4-methanocarbazoles. Reaction conditions: *N*-substituted 2-iodoaniline (1.0 eq.), NBE-5,6-diCOO*i*Pr (1.0 eq.), morpholino benzoate (1.3 eq.), Pd(OAc)_2_ (5% eq.), TFP (10% eq.), Cs_2_CO_3_ (2.5 eq.), toluene, 100 ^o^C, 12 h.

Different *N*-substituents were also demonstrated to be compatible within this transformation (Fig. 7). Notwithstanding a reduction in yield to 55% after enlarging the *N*-group from Me to *t*Bu (**5a**), bulky phenyl with various substituents (H, F, OMe) at different positions achieved the corresponding analogs (**5b**-**5e**) with good yield of around 80%. Pyridyl methyl substituted aniline also resulted in the desired compound (**5f**) in 69% yield.

This three-component reaction was subsequently successfully applied in synthesizing compound **3f** in 2g scale with a yield of 85% using commercially available starting materials in one step (Supplementary Fig. 1), which has been identified as a potent CYP11B1 inhibitor with an IC_50_ of 255 nM. In contrast, a 5-step synthesis of the iodoaniline starting material was necessary if following the direct condensation method and only a moderate yield of 42% was observed in the core construction step. The method of external amine integration produced worse results as the desired compound could barely be obtained. These facts clearly demonstrated the superiority of our method regarding convenience and efficiency in constructing the 5-substituted hexahydro-1*H*-1,4-methanocarbazole privileged scaffold.

As manifested above, a broad scope was demonstrated for this multicomponent reaction with respect to various types of electrophiles, as well as distinct substituents at almost every topological position of each reactant. This advantage not only guarantees fast access of target molecules from easily available starting materials, but also provides the possibility to build “natural-product-like” compound libraries with high structural complexity and diversity within a short space of time via, e.g., a combinatory chemistry approach. The 52 compounds synthesized in this manuscript (see Supplementary Method 1 for synthetic details and characterization data, and see Supplementary Data 2 for spectra of ^1^H-, ^13^C-, and ^19^F-NMR as well as HRMS) comprised a small focused compound library and were evaluated for their inhibition of CYP11B1 (see Supplementary Method 2 for inhibitory assay conditions). Most compounds exhibited moderate to potent inhibition (Supplementary Table 1) and hence were considered as lead compounds for further drug discovery in treating diabetic foot and hypercortisolism.

### Computational investigations on mechanisms

Furthermore, we employed DFT-based calculations to provide some mechanistic insights into the reaction pathways to understand how the desired main synthetic route competes with possible side reactions at the two diverging points—the phenylnorbornyl palladium species **I** and **III**. The DFT optimizations, potential energy scan and frequency calculations were performed using B3LYP-D3 level of theory with additional M06-2X single point calculations carried out using the DFT optimized geometry. The free energy profiles that we used in the discussion are the sum of the M06-2X potential energy and thermal corrections to free energy from the B3LYP-D3 frequency calculations (see Supplementary Method 3 for full details of the computational methods, and see Supplementary Data 3 for Cartesian coordinates of all the structures reported and their absolute energies in Hartree).

1 $$5,6- 	{{{{{\rm{DiCOO}}}}}}{i}{{\Pr }}\,{{{{{\rm{NBE}}}}}}:{{{{{\bf{I}}}}}}\to {{{{{\bf{I}}}}}}{{{{{\bf{n}}}}}}{{{{{\bf{t}}}}}}\_{{{{{\bf{I}}}}}}\to {{{{{\bf{I}}}}}}{{{{{\bf{I}}}}}}\,vs\,{{{{{\bf{I}}}}}}\to {{{{{\bf{I}}}}}}{{{{{\bf{n}}}}}}{{{{{\bf{t}}}}}}\_{{{{{\bf{V}}}}}}\to \\ 	{{{{{\bf{V}}}}}}\to {{{{{\bf{b}}}}}}{{{{{\bf{y}}}}}}{{-}}{{{{{\bf{p}}}}}}{{{{{\bf{r}}}}}}{{{{{\bf{o}}}}}}{{{{{\bf{d}}}}}}{{{{{\bf{u}}}}}}{{{{{\bf{c}}}}}}{{{{{\bf{t}}}}}}\,1{{{{{\bf{b}}}}}}$$

**Fig. 8:**
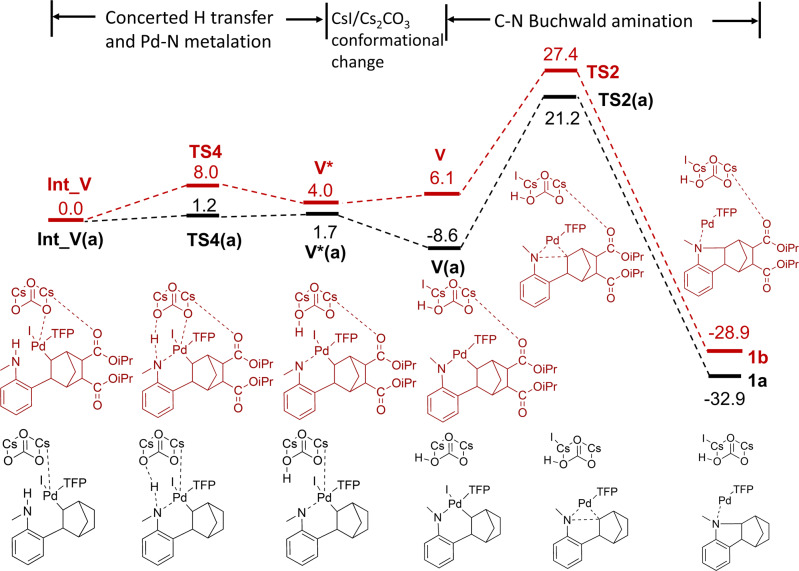
Computed free energy profile of **Int_V** → **V** → by-product **1a** and **1b**. Intermediates and transit states with 5,6-diCOO*i*Pr were illustrated in dark red, while unsubstituted ones were illustrated in black. The energies are given in kcal/mol.

The initial phenylnorbornyl palladium intermediate **I** is flexible and can either rotate for the reaction to flow towards **Int_I** → **II** or to follow the direction of **V** to **by-product 1b**. A previous computational study^34^ demonstrates that the energy associated with the rotation of **I** towards the two competing reaction pathways is nearly identical and is only 2–3 kcal/mol. Therefore, we assumed this step was unlikely to provide significant mechanistic insights and first calculated the energy profile related to the Pd-N metalation and intramolecular C(*sp*^3^)-N Buchwald amination to yield methanocarbazoles (by-product **1b**) for 5,6-diCOO*i*Pr NBE. We manually placed the base Cs_2_CO_3_ in Intermediate **I** in a similar position as seen in the previous computational study^34^ and optimized the system without any constraints. This resulted in a conformation where the distance between O in Cs_2_CO_3_ and H in the amine group is 1.73 Å, the distance between another O in Cs_2_CO_3_ and Pd is 2.1 Å and the distance between N in the amine group and Pd is 3.18 Å. It is noteworthy that the two Cs form interactions with the two carbonyl O in the 5,6-DiCOOiPr substitution on NBE (3.16 Å and 3.19 Å, respectively) (Supplementary Fig. 2). It seems Cs_2_CO_3_ forms multiple interactions with the intermediate to hold it in balance to form **Int_V**. The subsequent reaction involved two steps—Step 1 is the concerted hydrogen transfer from the amine group to the base Cs_2_CO_3_ and Pd-N metalation with a moderate reaction barrier of 8.0 kcal/mol and an overall reaction energy of 4.0 kcal/mol. Along the reaction pathway, the Pd-N distance is decreased from 3.18 Å to 2.14 Å while the O(Cs_2_CO_3_)-Pd distance is increased from 2.1 Å to 3.4 Å as the base is moving away from the reaction center upon protonation. Release of CsI from the reaction center is associated with a 2.1 kcal/mol increase in energy to form **V**. This is then followed by Step 2, the formation of the C–N bond to close the five-membered ring to form **1b**. The energy barrier for the C–N bond formation is 21.3 kcal/mol with an energy of reaction of –35.0 kcal/mol for 5,6-diCOO*i*Pr NBE (Fig. 8).

We then calculated the energy profile of generating **II** for 5,6-diCOO*i*Pr NBE, which is the key intermediate step leading to the formation of the desired product **2b**. Similar to the aforementioned approach, Intermediate **I** was rotated to a conformation suitable for C-H activation and Cs_2_CO_3_ was placed in a position close to the reaction center. After optimization, Cs_2_CO_3_ interacts with H in the C-H activation moiety, Pd and the carbonyl O in the 5,6-DiCOOiPr substitution to form **Int_I** (distances 2.16 Å, 2.09 Å, and 3.16 Å, respectively, Supplementary Fig. 3). This reaction is a concerted metalation–deprotonation process of the C-H activation and formation of the Pd–C bond, similar to what was observed in a previous study^42^.

**Fig. 9:**
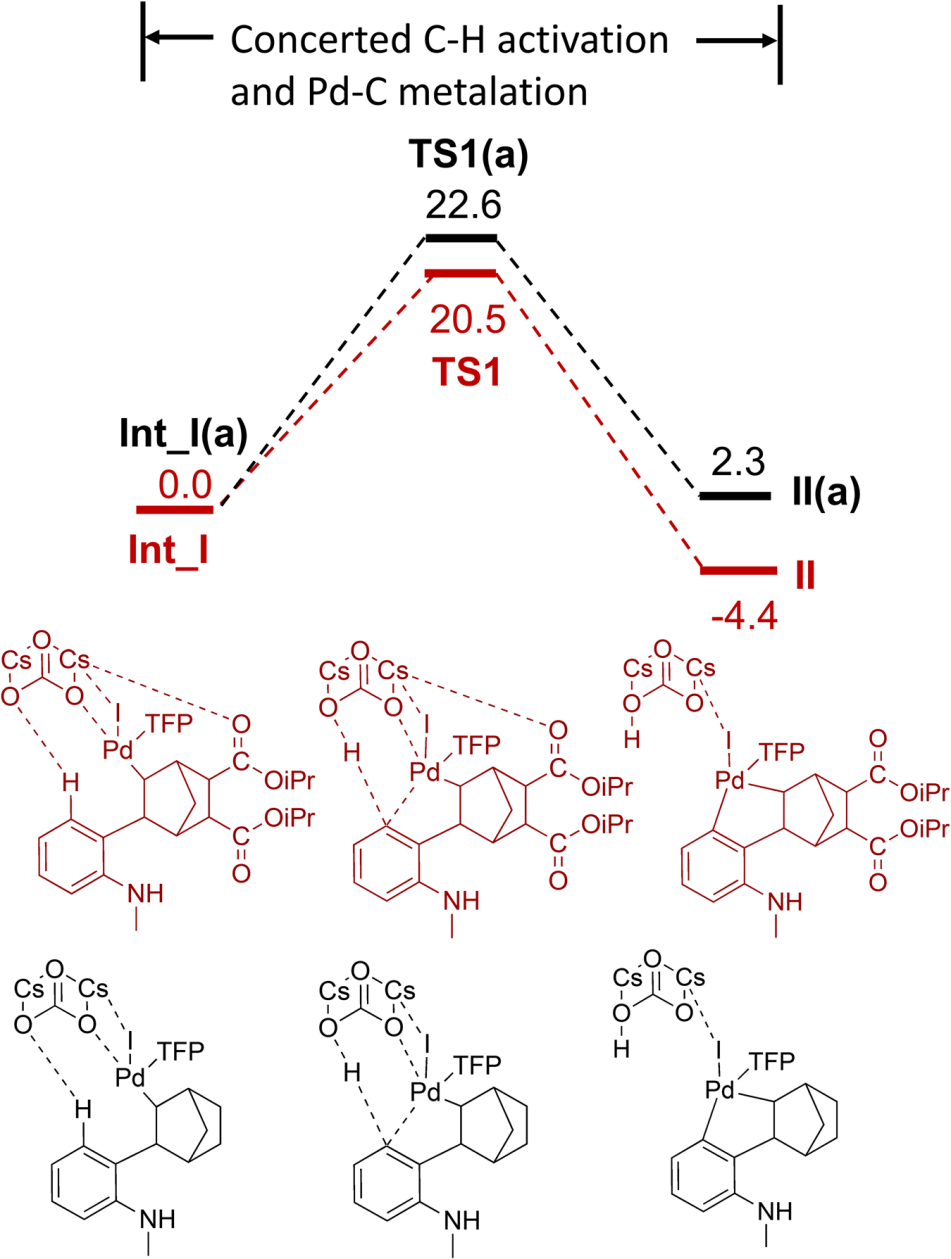
Computed free energy profile of **Int_I** → **TS1** → **II**. Intermediates and transit states with 5,6-diCOO*i*Pr were illustrated in dark red, while unsubstituted ones were illustrated in black. The energies are given in kcal/mol.

The reaction barrier height was calculated to be 20.5 kcal/mol and an energy of reaction –4.4 kcal/mol for this step (Fig. 9). The Pd-C distance is reduced from 3.16 Å to 2.03 Å along the reaction pathway. It is clear that for 5,6-diCOO*i*Pr NBE, the C-H activation direction of rection (**I** → **II)** is more kinetically and thermodynamically favored compared to the overall barrier of 27.4 kcal/mol for the Pd-N metalation/C-N Buchwald amination steps (**I** → **V**) (Fig. 8). This would explain why **2b** usually exhibited a higher yield than **1b** in both Fig. 3 and Table 1, strongly indicating that at the first diverging point, the reaction favors the direction of **I** → **II**.

Additionally, when TFP is replaced by DavePhos in **Int_I**, O in Cs_2_CO_3_ is further away from the hydrogen for C-H activation at 3.81 Å (Supplementary Fig. 3). This longer hydrogen transfer distance is expected to give rise to a higher energy barrier for the C-H activation with DavePhos, consistent with the experimental observation that bulky ligands such as RuPhos, DavePhos, and XPhos were ineffective in constructing the methanocarbazole core.

2 $$5,6- 	{{{{{\rm{DiCOO}}}}}}{i}{{\Pr }}\,{{{{{\rm{NBE}}}}}}:{{{{{\bf{I}}}}}}{{{{{\bf{I}}}}}}{{{{{\bf{I}}}}}}\to {{{{{\bf{I}}}}}}{{{{{\bf{V}}}}}}\to {{{{{\bf{p}}}}}}{{{{{\bf{r}}}}}}{{{{{\bf{o}}}}}}{{{{{\bf{d}}}}}}{{{{{\bf{u}}}}}}{{{{{\bf{c}}}}}}{{{{{\bf{t}}}}}}\,vs\,{{{{{\bf{I}}}}}}{{{{{\bf{I}}}}}}{{{{{\bf{I}}}}}}\to {{{{{\bf{V}}}}}}{{{{{\bf{I}}}}}}\to \\ 	{{{{{\bf{C}}}}}}{{{{{\bf{a}}}}}}{{{{{\bf{t}}}}}}{{{{{\bf{e}}}}}}{{{{{\bf{l}}}}}}{{{{{\bf{l}}}}}}{{{{{\bf{a}}}}}}{{{{{\bf{n}}}}}}{{{{{\bf{i}}}}}}\,{{{{{\bf{b}}}}}}{{{{{\bf{y}}}}}}-{{{{{\bf{p}}}}}}{{{{{\bf{r}}}}}}{{{{{\bf{o}}}}}}{{{{{\bf{d}}}}}}{{{{{\bf{u}}}}}}{{{{{\bf{c}}}}}}{{{{{\bf{t}}}}}}$$

The reaction mechanism of converting **III** to **IV** is different to what has been discussed for **Int_V** → **V** → **by-product**. After introducing a substitution group at the 5-position using morpholino in **III**, the concerted hydrogen transfer from the amine group to the base Cs_2_CO_3_ and Pd-N metalation occurs simultaneously along the optimization without a clear barrier. The energy barrier for the C-N formation is lowered to 17.0 kcal/mol and the energy of reaction is –43.0 kcal/mol, both lower compared to **V** → **by-product 1b** (Supplementary Fig. 4). Consistent with the study by Zhang et al.^34^, the barrier for the C-N Buchwald amination decreased significantly, which may be due to conjugation with the secondary amine group. It is noteworthy that the mechanism of this C-N Buchwald amination is similar to the DFT study reported by McMullin et al.^43^, but different from the study by Zhang et al.^34^, in which the bulkier *tert*-butyl (*t*Bu) and *tert*-butyloxycarbonyl (*t*BOC) groups were used on the nitrogen. In that case, the steric hindrance from the bulkier groups prevents Pd from attacking the nitrogen directly and the hydrogen transfer from the amine would occur with a considerable barrier (~16 kcal/mol), followed by Pd-N metalation also with a lower barrier (~9 kcal/mol) (15.5 kcal/mol if the hydrogen transfer and Pd-N metalation are concerted). In our DFT study, the smaller methyl group on the nitrogen enabled simultaneous hydrogen transfer and Pd-N metalation, leading to the rapid formation of **IV**. It is plausible that this introduces a catalytic advantage for the main reaction pathway (**III**–**IV**) over the side reaction of NBE extrusion in the Catellani-Lanterns reaction (**III**–**VI**). Consistent with insights from the DFT calculations, we observed a reduction in yield from 78% to 55% experimentally after enlarging the *N*-group from Me to *t*Bu (**5a**) (Fig. 7), which may be the result of an increased side reaction of **III** → **VI**. Interestingly, the steric hindrance of the *N*-groups seems to be most pronounced in the position right adjacent to the nitrogen, as various bulky benzyl analogs (a slim methylene presents in between) achieved good yields (Fig. 7).

3 $$	5,6- {{{{{\rm{DiCOO}}}}}}i \, {{\Pr }}\,vs\,{{{{{\rm{unsubstituted}}}}}}\,{{{{{\rm{NBE}}}}}}\,{{{{{\rm{in}}}}}}\,{{{{{\bf{I}}}}}}{{{{{\bf{n}}}}}}{{{{{\bf{t}}}}}}\_{{{{{\bf{I}}}}}}\to \\ 	{{{{{\bf{I}}}}}}{{{{{\bf{I}}}}}}\,({{{{{\bf{I}}}}}}{{{{{\bf{n}}}}}}{{{{{\bf{t}}}}}}\_{{{{{\bf{I}}}}}}({{{{{\bf{a}}}}}})\to {{{{{\bf{I}}}}}}{{{{{\bf{I}}}}}}({{{{{\bf{a}}}}}}))\,{{{{{\rm{and}}}}}}\,{{{{{\bf{I}}}}}}{{{{{\bf{n}}}}}}{{{{{\bf{t}}}}}}\_{{{{{\bf{V}}}}}}\to {{{{{\bf{V}}}}}}\to 1{{{{{\bf{b}}}}}}\,({{{{{\bf{I}}}}}}{{{{{\bf{n}}}}}}{{{{{\bf{t}}}}}}\_{{{{{\bf{V}}}}}}({{{{{\bf{a}}}}}})\to \\ 	{{{{{\bf{V}}}}}}({{{{{\bf{a}}}}}})\to 1{{{{{\bf{a}}}}}}),\,{{{{{\rm{respectively}}}}}}$$

**Fig. 10:**
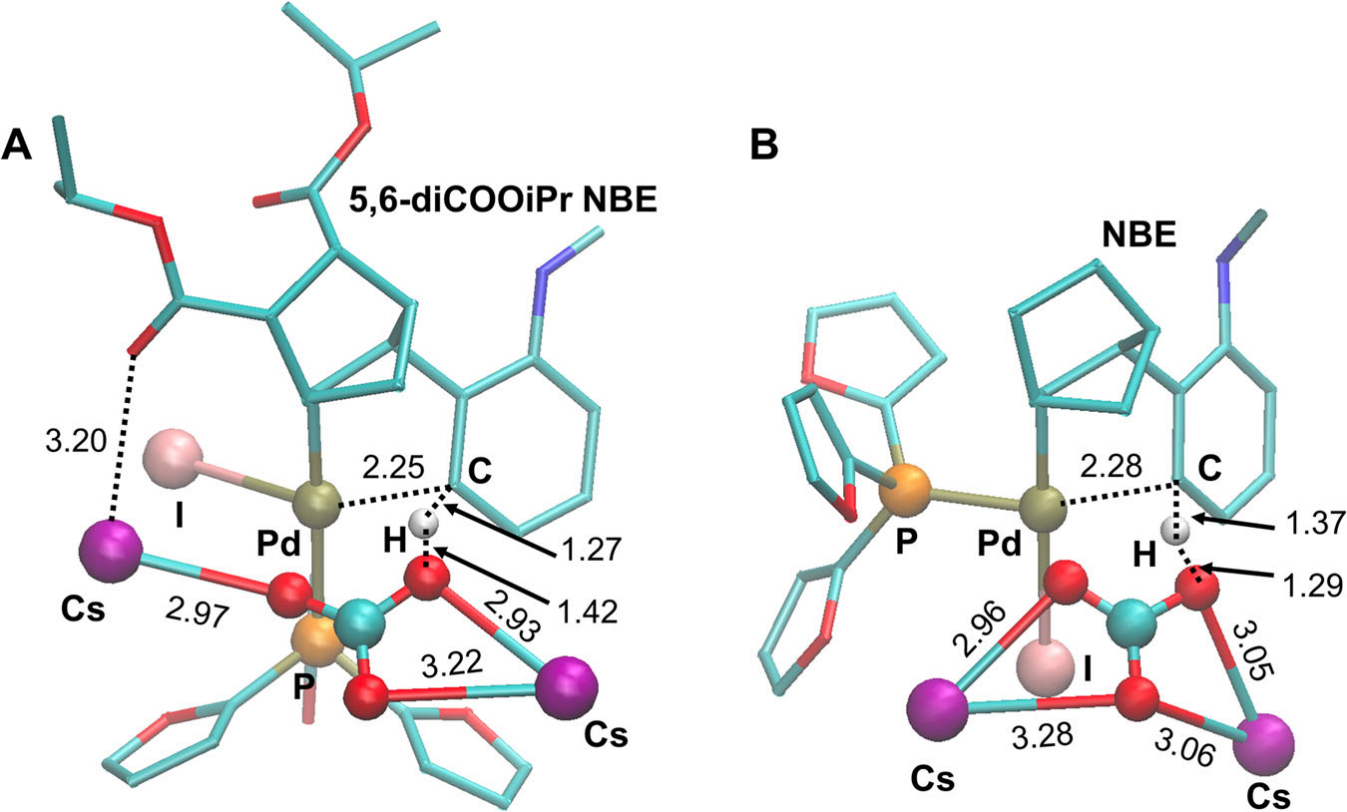
Transition state structures **TS1** and **TS1(a)**. Transition state structures of the Cs_2_CO_3_-induced C-H activation through a concerted metalation–deprotonation process to result in **II** and **II(a)**. **A** With 5,6-diCOO*i*Pr NBE (barrier height 20.5 kcal/mol), where a coordination between Cs and carbonyl O was observed, and **B** With the unsubstituted NBE (barrier height 22.6 kcal/mol). Different orientations of TFP were noted.

The effect of NBE substitutions was studied by comparing unsubstituted NBE with 5,6-diCOO*i*Pr NBE in **Int_I** → **II** and **Int_V** → **V** → **1a/1b**, respectively, at the first diverging point. Although the C-H activation in transforming from **Int_I** to **II** is the well-studied concerted metalation–deprotonation process, 5,6-diCOO*i*Pr NBE exhibits a slightly lower reaction barrier (20.5 kcal/mol) compared to that of the unsubstituted NBE (22.6 kcal/mol) (Fig. 9) and the transition state structures of these two are also noticeably different. The transition state structure of unsubstituted NBE from our calculation is similar to a methyl analog reported in a previous DFT study using the same level of theory^43^. However, in the transition state structure of 5,6-diCOO*i*Pr NBE, the Pd ligand TFP is rotated substantially because of the steric hindrance caused by the substitution, and a Cs atom from the base Cs_2_CO_3_ forms a coordination with a carbonyl in the NBE substitution (Fig. 10). We tried to delete the 5,6-diCOO*i*Pr and re-optimize the complex, but it did not give a valid structure, implying that the NBE substitutions along with Cs_2_CO_3_ may facilitate the C-H activation through an altered conformation in comparison to the unsubstituted NBE.

Along the **Int_V(a)**→**V(a)**→**by-product 1a** pathway, the distance between O in Cs_2_CO_3_ and H in the amine group is 1.60 Å, poised for H transfer and the distance between N in the amine group and Pd is 2.24 Å in **Int_V**^**a**^, significantly shorter than the 3.18 Å seen in **Int_V**. In addition, no coordination is formed between O in Cs_2_CO_3_ and Pd (Supplementary Fig. 2). Consequently, the concerted hydrogen transfer from the amine group to the base Cs_2_CO_3_ and Pd-N metalation occurs with a negligible 1.2 kcal/mol barrier and 1.7 kcal/mol for the energy of reaction to form **V(a)**. The barrier for the C–N bond formation from **V** to **1a** is 29.8 kcal/mol for the unsubstituted NBE. Comparison with unsubstituted and substituted NBE indicates that Cs_2_CO_3_ plays multiple roles in the substituted case-forming coordination with Pd, interacting with the 5,6-DiCOO*i*Pr substitution along with H abstraction, which raises the barrier in going from **Int_V** to **V** and pushes the reaction flow towards the main reaction pathway consequently. This indicated that interactions between Cs and the substitutions on NBE may modulate the reaction barriers. Taken together, the unsubstituted NBE is more likely to proceed through the C-N Buchwald amination from **I**. This is consistent with our experiment that the yield of **1a** (62%) was significantly higher than that of **1b** (15%) in Fig. 3. It is worth noting that some calculated barriers are sensitive to the level of DFT method used, in particular when they are associated with hydrogen transfer and the position of Cs_2_CO_3_ (Supplementary Table 2). This is due to factors such as the intrinsic errors of DFT methods and the limits of implicit solvent model and that energetics are derived from potential energy scan, which restricts conformational sampling. Nonetheless, the order of barriers from the current study is in line with previous computational studies where energy differences between different reaction pathways can be also rather small. More importantly, our DFT calculations are consistent with our experimental data and provide additional mechanistic insights, which reflects the strength of a combined experimental and computational approach.

## Discussions

The sophisticated but convenient reaction accomplished in our multicomponent strategy was a cascade consisted of intercepted Heck addition/C(*sp*^2^)-H activation/C-palladacycle (ANP) formation/electrophilic oxidative addition and reductive elimination/N-palladacycle formation/intramolecular Buchwald amination (Fig. 2). In contrast, the previously reported palladium-catalyzed direct condensation involved only the commencing and consequent stages, in which the initial phenylnorbornyl palladium species **I** was generated by intercepting a Heck reaction and then underwent an immediate Buchwald amination to yield the final compound. Compared to direct condensation, a detour was deliberately introduced in our approach, in which the initial phenylnorbornyl palladium species **I** was driven into an ANP **II** via *ortho* C(*sp*^2^)-H activation and a more complex phenylnorbornyl palladium species **III** for the final Buchwald amination was subsequently regenerated from an electrophilic attack of the ANP. Obviously, such a regeneration of reactive species provided the possibilities of introducing further structural complexity in a single reaction.

The first part of this method was alike to that of the Catellani-Lautens reaction in mechanism. However, a Catellani-Lautens reaction would not take place when a secondary amino group furnished *ortho* to the carbon being palladated. An intramolecular Buchwald amination would occur instead after the migratory insertion of NBE (as seen in method of direct condensation)^44,45^. Our study provided an unusual example of transforming a phenylnorbornyl palladium species into an ANP in the presence of an adjacent amino group. Moreover, a distinct divergence occurred upon the regenerated phenylnorbornyl palladium species **III**, which underwent a Buchwald amination to form the methanocarbazole core in our method, but would eliminate the NBE moiety resulting in a phenyl palladium complex **VI** in a Catellani-Lautens reaction. Nevertheless, as minor amount of NBE extruding product **VII** (<5%) was observed in some cases, it would be possible to achieve Catellani-Lautens reactions with suitable nucleophiles after additional modifications of NBE, *N*-substituents and reacting conditions. Apparently, the initial and regenerated phenylnorbornyl palladium species (**I** & **III**) served as switches and these two diverging points determined the outcomes of this complex reaction comprising of multiple concomitant electrophiles and nucleophiles.

In this study, the initial phenylnorbornyl palladium species **I** was generated via the migratory insertion of a modified NBE to a aryl-Pd(II) intermediate, which was yielded from the oxidative addition of an aryl halide (e.g., aminophenyl iodide) to a Pd(0) catalyst. Notably, such an aryl-Pd(II) intermediate could also be obtained by direct electrophilic palladation of heterocycles like thiophene^46^ and indole^47,48^. These heterocycles could be explored as the substrates, therefore further expanding the scope of this method. Similarly, other bridged bicyclic olefins as bioisosteres of NBE, such as 7-azanorbornene, vince lactam, and camphene, are likely to produce analogous aryl-bicycloalkyl palladium species for subsequent reaction cascade due to their strained and tethered conformations. These potential substrates could further increase the structural diversity of the end products when employing our method to build a natural-product-like compound library.

Furthermore, the phenylnorbornyl palladium species exploited in our study are a special case of σ-alkyl palladium complexes, which present in various C-H and C-C activations as reactive intermediates^49^. In most reports, only a single σ-alkyl palladium species was generated in situ and subsequently trapped by a reaction partner to terminate the reaction. However, besides interception of an Heck reaction, the σ-alkyl palladium complex could be generated in several ways, including directed C(*sp*^3^)−H activation assisted with coordinating auxiliaries and electrophilic attacks of certain palladacycles. Moreover, these σ-alkyl palladium species could also be captured in multiple approaches, such as reacting with various nucleophiles (e.g., boronic acids), and coupling with benzyne. Given these facts, the “detour by regeneration” strategy in exploiting phenylnorbornyl palladium species manifested in the current study could be feasible for other σ-alkyl palladium complexes, and therefore would facilitate their applications in synthesizing highly structurally complex molecules via multicomponent cascades within a single reaction.

## Conclusion

In conclusion, we have accomplished a convenient multicomponent construction of 5-substituted hexahydro-1*H*-1,4-methanocarbazoles as single stereoisomers, which were otherwise difficult to access. This was achieved through manipulation of two phenylnorbornyl palladium species generated successively by modifying the NBE reactant with distinct substituents and careful alterations of the reaction conditions. This method exhibited a broad scope to various reactants and would facilitate the construction of natural-product-like compound libraries with high structural complexity and diversity. Such a “detour by regeneration” concept in exploiting phenylnorbornyl palladium species could be feasible for the application of other σ-alkyl palladium complexes in sophisticated organic synthesis. We believe this innovative synthesis strategy will promote the implementation of hexahydromethanocarbazole as a privileged scaffold for the development of new drugs, materials and biomedical probes.

## Methods

A mixture of the corresponding substituted 2-iodo-*N*-substituted aniline (0.43 mmol), substituted norbornene derivatives (0.65 mmol), a proper electrophile reagent (such as amino benzoate, arylbromide, or benzoic anhydride, 0.86 mmol), Cs_2_CO_3_ (224 mg, 1.60 mmol), Pd(OAC)_2_ (9 mg, 0.04 mmol), and TFP (24 mg, 0.08 mmol) was stirred in toluene (2 mL) at 100 °C under an inert atmosphere for 12 h. After the reaction mixture was cooled down to room temperature, it was filtrated and extracted with ethyl acetate/brine for three times. The combined organic layers were dried over anhydrous Na_2_SO_4_ and concentrated to give the crude product, which was subsequently purified by column chromatography (petroleum ether/ethyl acetate) to yield the desired product.

### Reporting summary

Further information on research design is available in the Nature Research Reporting Summary linked to this article.

## Supplementary information

Supplementary Information

Description of Additional Supplementary Files

Supplementary Data 1

Supplementary Data 2

Supplementary Data 3

Reporting Summary

## Data Availability

Methods and experimental procedures of chemical synthesis, CYP11B1 inhibitory assay and DFT calculations, characterization of final compounds, as well as energy profiles and illustrations of key intermediates are available in the Supplementary Information. The X-ray crystallographic coordinates for structures reported in this Article have been deposited at the Cambridge Crystallographic Data Center (CCDC), under deposition number CCDC 2121980. These data can be obtained free of charge from The Cambridge Crystallographic Data Center via www.ccdc.cam.ac.uk/data_request/cif. Supplementary Data 1: crystal data of compound **2b**. Supplementary Data 2: The ^1^H-, ^13^C-, and ^19^F-NMR as well as HRMS spectra of final compounds. (10.6084/m9.figshare.21252192.v1). Supplementary Data 3: Cartesian coordinates of all the structures reported and their absolute energies in Hartree (10.6084/m9.figshare.21252711.v1).
