# Supplementary material for: Manipulations of phenylnorbornyl palladium species for multicomponent construction of a bridged polycyclic privileged scaffold
Source: Commun Chem. 2022 Oct 29;5:140. doi: 10.1038/s42004-022-00759-4 (PMC9814782; doi:10.1038/s42004-022-00759-4)
Supplement: Supplementary file 1 — Supplementary Information [file 42004_2022_759_MOESM1_ESM.pdf]

Manipulations of Phenylnorbornyl Palladium Species for Multicomponent Construction of a Bridged Polycyclic Privileged Scaffold

Lina Yin,*[a] Ting Guan,‡[a] Jie Cheng,‡[a] Dongchao Pan,‡[a] Jinyang Lu,‡[a] Jiahui Huang,[a] Jiaqi Wu,[a] Xiaoli Chen,[a] Taiyun You,[a] Xuting Huo,[a] Yuting He,[a] Jiayun Pang,*[b] and Qingzhong Hu*[a]

[a] T. Guan, J. Cheng, D. Pan, J. Lu, M. Liu, J. Huang, J. Wu, X. Chen, T. You, X. Huo, Y. He, Prof. Dr. L. Yin, and Prof. Dr. Q. Hu
School of Pharmaceutical Sciences, Guangzhou University of Chinese Medicine
232 East Waihuan Road, Panyu, Guangzhou, China PR
E-mail: huqqzh@gzucm.edu.cn (Q. Hu); linayin@gzucm.edu.cn (L. Yin)

[b] Dr. J Pang
School of Science, Faculty of Engineering and Science, University of Greenwich
Medway Campus, Central Avenue, Chatham Maritime, ME4 3RL, United Kingdom
Email: j.pang@gre.ac.uk (J. Pang)

‡ These authors contribute equally to this work.

Table of Contents

1. **Supplementary Figure 1.** Advantages of this "detour" strategy ······ 3
2. **Supplementary Figure 2:** B3LYP-D3-optimised structures of **Int_V** and **Int_V**[a] ······ 4
3. **Supplementary Figure 3**: B3LYP-D3-optimised structures of **Int_I** (with different ligands) ······ 5
4. **Supplementary Figure 4**: Computed free energy profile of **IV**→**V**→the product **2b** ······ 6
5. **Supplementary Table 1**: The inhibition of synthesized hexahydromethanocarbazoles against CYP11B1 ······ 7
6. **Supplementary Table 2**: B3LYP and M06-2X energy profiles of key intermediates ······ 8
7. **Supplementary Method 1**: The synthesis and characterization of final compounds ······ 9
8. **Supplementary Method 2**: Inhibitory assay of CYP11B1 ······ 30
9. **Supplementary Method 3**: DFT calculation ······ 31
10. **Supplementary Note 1**: Configuration determination of compound **2l** with HSQC, HMBC and NOESY spectra ······ 32
11. **Supplementary References** ······ 34

Supplementary Figure 1

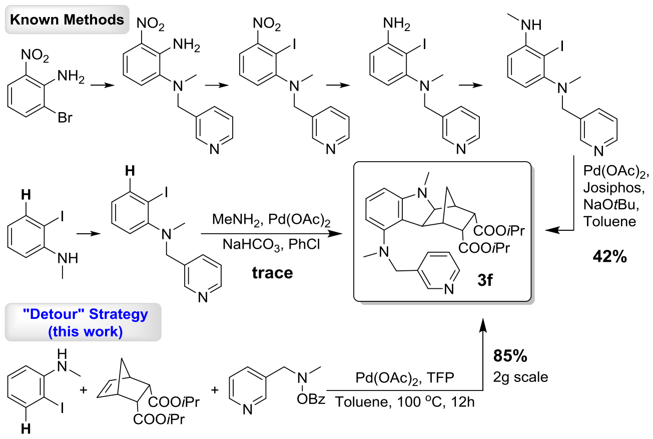

Figure S1. Advantages of this "detour" strategy over reported methods in synthesizing CYP11B1 inhibitor **3f**

Supplementary Figure 2

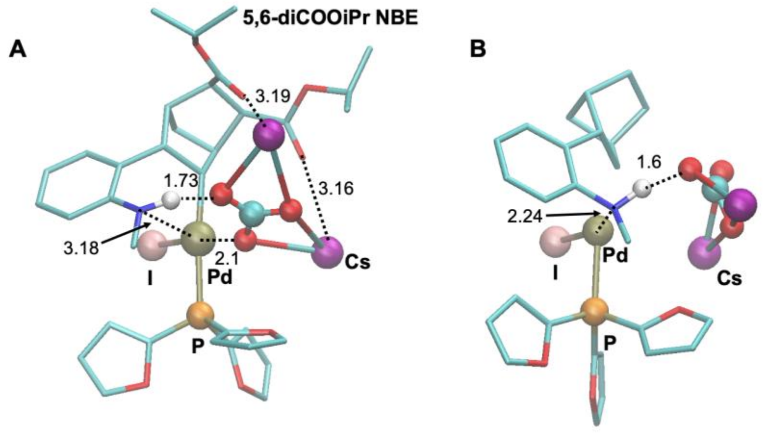

Figure S2: B3LYP-D3-optimised structures of **Int_V,** where a coordination between Cs and carbonyl O was observed (A, with 5,6-diCOO*i*Pr NBE) and **Int_V**^{a} (B, with the unsubstituted NBE). The distances are in Å.

Supplementary Figure 3

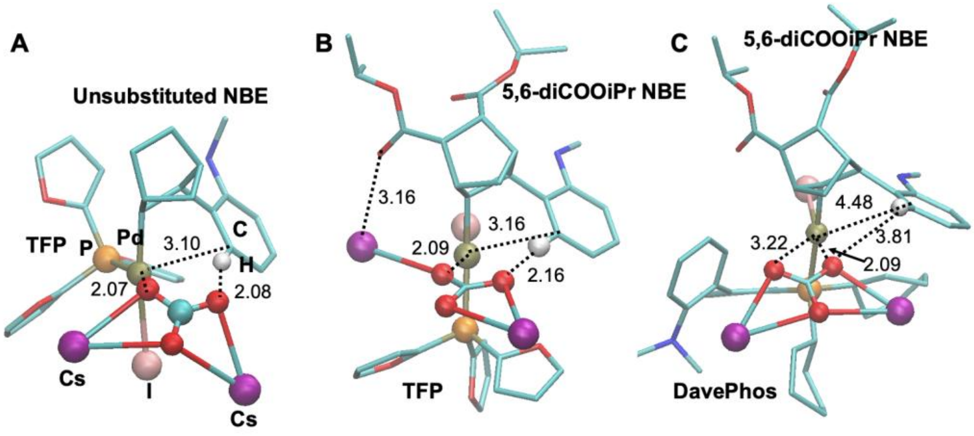

Figure S3: B3LYP-D3-optimised structures of **Int_I** for (A) unsubstituted NBE and TFP, (B) 5,6-diCOO*i*Pr NBE and TFP and (C) 5,6-diCOO*i*Pr NBE and DavePhos. The distances are in Å.

Supplementary Figure 4

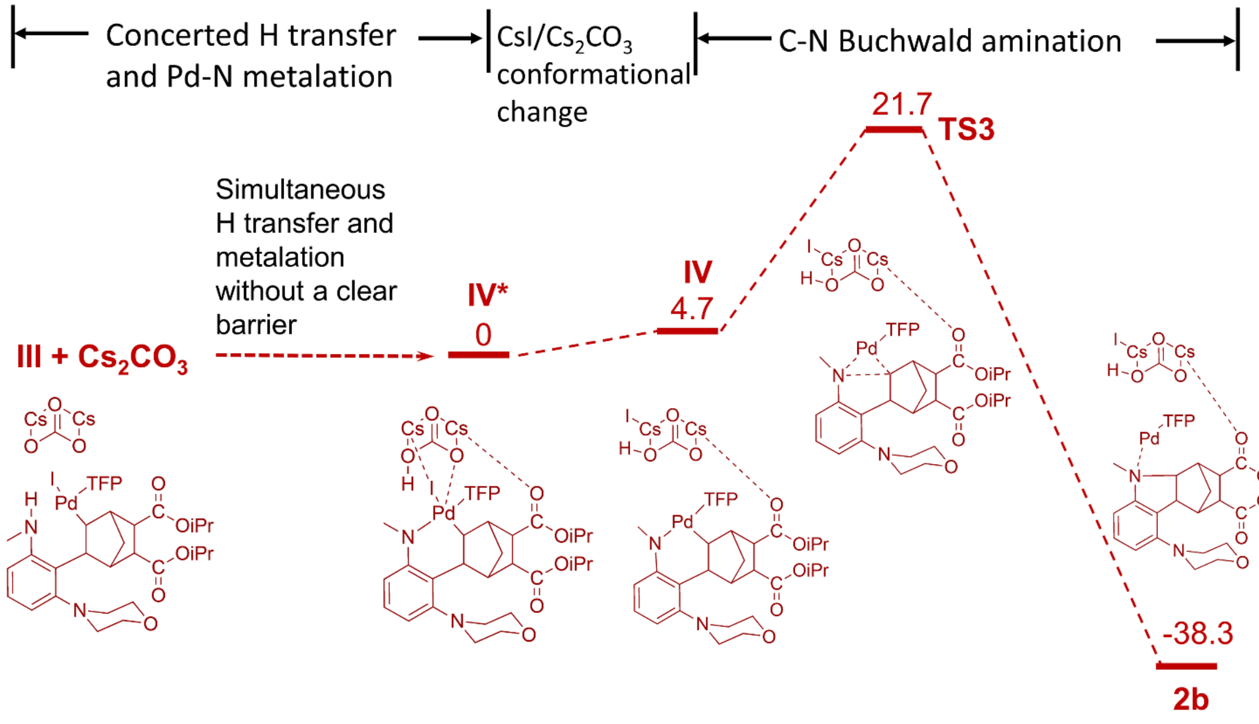

Figure S4: Computed free energy profile of **IV**→**V**→the product **2b**. The energies are given in kcal/mol.

Supplementary Table 1. The inhibition of synthesized hexahydromethanocarbazole compounds against CYP11B1.[a,b]

Compd.	IC_{50} (nM)	Compd.	IC_{50} (nM)	Compd.	IC_{50} (nM)	Compd.	IC_{50} (nM)
**2a**	573±35	**2b**	327±53	**2c**	501±64	**2d**	428±30
**2e**	586±25	**2f**	225±17	**2g**	297±32	**2h**	734±24
**2i**	796±49	**2j**	567±61	**2k**	335±38	**3a**	354±58
**3b**	636±41	**3c**	283±19	**3d**	546±61	**3e**	620±37
**3f**	463±39	**3g**	583±63	**3h**	324±28	**3i**	395±48
**3j**	988±74	**3k**	477±38	**3l**	571±63	**3m**	581±39
**3n**	636±45	**3o**	379±52	**3p**	891±84	**3q**	521±60
**3r**	387±53	**3s**	580±45	**3t**	537±61	**3u**	406±28
**3v**	563±69	**3w**	332±29	**3x**	428±67	**3y**	351±38
**3z**	492±72	**4a**	743±54	**4b**	884±65	**4c**	573±68
**4d**	308±26	**4e**	661±37	**4f**	305±48	**4g**	662±53
**4h**	367±46	**4i**	319±28	**5a**	449±32	**5b**	537±54
**5c**	537±62	**5d**	288±54	**5e**	691±51	**5f**	327±29

[a] Mean value of at least three experiments; standard deviation less than 20%; [b] CYP11B1 assay: Hamster fibroblasts expressing human CYP11B1 with 11-deoxycortisol (100 nM) as the substrate;

Supplementary Table 2: B3LYP and M06-2X energy profile of key intermediates presented in Figure 8,9 and 10. Numbers in brackets indicate energy associated with the particular step.

	B3LYP-D3 potential energy		B3LYP-D3 free energy		M06-2X free energy	
Int_I	0		0		0	
TS1	22.9		19.9		20.5	
II	-8.0		-9.9		-4.4	
Int_I(a)	0		0		0	
TS1(a)	23.5		19.9		22.6	
II(a)	0.7		-1.8		2.3	
Int_V	0		0		0	
TS4	10.4		8.2		8.0	
V*	2.5		3.0		4.0	
V	5.8	(0)	0.2	(0)	6.1	(0)
TS2	28.7	(22.9)	21.8	(21.6)	27.4	(23.1)
1b (by-product)	-26.6	(-32.4)	- 29.7	(-29.9)	-28.9	(-35.0)
Int_V(a)	0		0		0	
TS4(a)	1.5		-1.0		1.2	
V*(a)	-0.8		-2.5		1.7	
V[a]	-4.7	(0)	-9.4	(0)	-8.6	(0)
TS[a]	27.7	(32.4)	21.4	(30.8)	21.2	(29.8)
1a (by-product)	-31.6	(-26.9)	-35.6	(-26.2)	-32.9	(-24.3)
IV*	0		0		0	
IV	0.7	(0)	-1.4	(0)	4.7	(0)
TS3	20.7	(20.0)	17.0	(18.4)	21.7	(17.0)
2b (product)	-37.0	(-37.7)	-37.6	(-36.2)	-38.3	(-43.0)

Supplementary Method 1: The synthesis and characterization of final compounds:

General information: All evaporations were carried out *in vacuo* with a rotary evaporator. Analytical samples were dried *in vacuo* (1-5 mmHg) at room temperature. Thin layer chromatography (TLC) was performed on silica gel plates, spots were visualized by UV light (214 and 254 nm). Purification by column and flash chromatography was carried out using silica gel (200-300 mesh). Solvent systems are reported as mixtures by volume. All NMR spectra were recorded on a Bruker 400 (400 MHz) spectrometer. ^{1}H and ^{13}C chemical shifts are reported in δ values in ppm with the deuterated solvent as the internal standard. Data are reported as follows: chemical shift, multiplicity (s = singlet, d = doublet, t = triplet, q = quartet, br = broad, m = multiplet), coupling constant (Hz), integration. LCMS spectra were obtained on an Agilent 1200 series 6110 or 6120 mass spectrometer with electrospray ionization and excepted as otherwise indicated, the general LCMS condition was as follows: Waters X Bridge C18 column (50 mm x 4.6 mm x 3.5 um), Flow Rate: 2.0 mL/min, column temperature: 40 ℃.

General Method A

A mixture of the corresponding substituted 2-iodo-*N*-methylaniline (0.43 mmol), substituted norbornene derivatives (0.65 mmol), morpholino benzoate (230 mg, 0.86 mmol), Cs_2CO_3 (224 mg, 1.60 mmol), $Pd(OAC)_2$ (9 mg , 0.04 mmol), and TFP (24 mg, 0.08 mmol) in toluene (2 mL) was stirred at 100 ℃ for 12 h. After the reaction mixture was cooled down to room temperature, it was filtrated and extracted with ethyl acetate/brine for three times. The combined organic layers were dried over anhydrous Na_2SO_4 and concentrated to give the crude product, which was subsequently purified by column chromatography (petroleum ether/ethyl acetate) to yield the desire product.

General Method B

A mixture of the corresponding substituted 2-iodo-*N*-methylaniline (0.22 mmol), diisopropyl bicyclo[2.2.1]hept-5-ene-2,3-dicarboxylate(115 mg, 0.43 mmol), methyl 2-bromobenzoate (97 mg, 0.43 mmol), K_2CO_3 (119 mg, 0.86 mmol), $Pd(OAC)_2$ (5 mg, 0.02 mmol), and TFP (10 mg, 0.02 mmol) in DMF (2 mL) was stirred at 100 ℃ for 12 h. After the reaction mixture was cooled down to room temperature, it was filtrated and extracted with ethyl acetate/brine for three times. The combined organic layers were dried over anhydrous Na_2SO_4 and concentrated to give the crude product, which was subsequently purified by column chromatography (petroleum ether/ethyl acetate) to yield the desire product.

General Method C

A mixture of the corresponding substituted benzoic anhydride (0.80 mmol), 2-iodo-*N*-methylaniline (93 mg, 0.40 mmol), TFP (19 mg, 0.08 mmol), $PdCl_2$ (7 mg, 0.04 mmol), Cs_2CO_3 (521 mg, 1.60 mmol) and diisopropyl bicyclo[2.2.1]hept-5-ene-2,3-dicarboxylate (160 mg, 0.60 mmol) in DME (3 mL) was stirred at 100 ℃ for 12 h. After the reaction mixture was cooled down to room temperature, it was filtrated and extracted with ethyl acetate/brine for three times. The combined organic layers were dried over anhydrous Na_2SO_4 and concentrated to

give the crude product, which was subsequently purified by column chromatography (petroleum ether/ethyl acetate) to yield the desire product.

4-((1*S*,2*S*,3*R*,4*S*,4a*S*,9a*S*)-9-Methyl-2,3,4,4a,9,9a-hexahydro-1*H*-1,4-methanocarbazol-5-yl)morpholine (2a)

Synthesized according to General Method A using 2-iodo-*N*-methylaniline (93 mg, 0.40 mmol), bicyclo[2.2.1]hept-2-ene (56 mg, 0.60 mmol), morpholino benzoate (166 mg, 0.80 mmol), Cs_2CO_3 (521 mg, 1.60 mmol), $Pd(OAc)_2$ (9 mg, 0.04 mmol), and TFP (21 mg, 0.08 mmol) in toluene (3 mL). The crude product was purified by column chromatography (petroleum ether/ethyl acetate = 30/1) to give **2a** (31 mg, 27% yield) as a pale-yellow oil. HRMS (ESI): calcd. for $C_{18}H_{24}N_2O$ $[M+H]^+$: 285.1889, found: 285.1948; ^{1}H-NMR (400 MHz, $CDCl_3$): δ = 6.99 (t, *J* = 7.9 Hz, 1H), 6.23 (d, *J* = 7.9 Hz, 1H), 6.01 (d, *J* = 7.4 Hz, 1H), 3.80 (t, *J* = 8.4 Hz, 4H), 3.50 (d, *J* = 8.3 Hz, 1H), 3.26 (d, *J* = 7.9 Hz, 1H), 3.22–3.13 (m, 2H), 2.88–2.80 (m, 2H), 2.77 (d, *J* = 4.3 Hz, 3H), 2.68 (s, 1H), 2.37 (s, 1H), 1.56 (dd, *J* = 8.9, 3.3 Hz, 2H), 1.43–1.36 (m, 1H), 1.29 (s, 1H), 1.18 (dd, *J* = 7.5, 1.6 Hz, 1H), 1.10 (d, *J* = 10.1 Hz, 1H); ^{13}C-NMR (100 MHz, $CDCl_3$): δ = 149.16, 138.90, 129.50, 128.91, 122.53, 106.86, 72.79, 67.52, 51.20, 50.34, 40.55, 39.58, 32.91 (2C), 30.99, 28.86, 25.05 (2C).

Diisopropyl (1*S*,2*S*,3*R*,4*S*,4a*S*,9a*S*)-9-methyl-5-morpholino-2,3,4,4a,9,9a-hexahydro-1*H*-1,4-methanocarbazole-2,3-dicarboxylate (2b)

Synthesized according to General Method A using 2-iodo-*N*-methylaniline (100 mg, 0.43 mmol), diisopropyl bicyclo[2.2.1]hept-5-ene-2,3-dicarboxylate (178 mg, 0.65 mmol), morpholino benzoate (230 mg, 0.86 mmol), Cs_2CO_3 (224 mg, 1.60 mmol), $Pd(OAC)_2$ (9 mg, 0.04 mmol), and TFP (24 mg, 0.08 mmol) in toluene (2 mL) was stirred at 100 ℃ for 12 h. The crude product was purified by column chromatography (petroleum ether/ethyl acetate = 30/1) to give **2b** (182 mg, 93% yield) as a pale-yellow solid, mp 123-124 ℃. HRMS (ESI): calcd. for $C_{26}H_{36}N_2O_5$ $[M+H]^+$: 457.2697, found: 457.2688; ^{1}H-NMR (400 MHz, CDCl3): δ = 7.00 (t, *J* = 7.8 Hz, 1H), 6.24 (s, 1H), 6.06 (s, 1H), 5.10–4.89 (m, 2H), 4.21 (d, *J* = 8.2 Hz, 1H), 3.89 (dd, *J* = 8.8, 6.0 Hz, 2H), 3.85–3.76 (m, 3H), 3.35 (d, *J* = 8.8 Hz, 2H), 3.10 (dd, *J* = 11.7, 4.6 Hz, 1H), 3.05 (d, *J* = 2.6 Hz, 1H), 2.93 (dd, *J* = 11.7, 3.8 Hz, 1H), 2.82 (s, 2H), 2.78 (s, 3H), 2.66 (s, 1H), 1.60–1.57 (m, 1H), 1.29–1.19 (m, 13H); ^{13}C-NMR (100 MHz, CDCl3): δ = 171.76, 171.52, 155.77, 149.60, 129.11, 121.37, 106.67, 100.95, 68.01, 67.79 (2C), 67.66, 51.00 (2C), 46.39 (2C), 45.46 (2C), 45.28, 44.36, 42.46, 34.61, 22.32, 22.18, 22.05, 21.99.

Dimethyl (1*S*,2*S*,3*R*,4*S*,4a*S*,9a*S*)-9-methyl-5-morpholino-2,3,4,4a,9,9a-hexahydro-1*H*-1,4-methanocarbazole-2,3-dicarboxylate (2c)

Synthesized according to General Method A using 2-iodo-*N*-methylaniline (93 mg, 0.40 mmol), dimethyl bicyclo[2.2.1]hept-5-ene-2,3-dicarboxylate (126 mg, 0.60 mmol), morpholino benzoate (166 mg, 0.80 mmol), Cs_2CO_3 (521 mg, 1.60 mmol), $Pd(OAc)_2$ (9 mg, 0.04 mmol), and TFP (21 mg, 0.08 mmol) in toluene (3 mL). The crude product was purified by column chromatography (petroleum ether/ethyl acetate = 20/1) to give **2c** (109 mg, 68% yield) as a pale-yellow oil. HRMS (ESI): calcd. for $C_{22}H_{28}N_2O_5$ $[M+H]^+$: 401.1998, found: 401.2081; ^{1}H-NMR (400 MHz, $CDCl_3$): δ = 6.99 (t, *J* = 7.9 Hz, 1H), 6.22 (d, *J* = 8.0 Hz, 1H), 6.04 (d, *J* = 7.8 Hz, 1H), 4.00 (d, *J* = 8.5 Hz, 1H), 3.92–3.84 (m, 3H), 3.83–3.76 (m, 2H), 3.67 (d, *J* = 11.3 Hz, 6H), 3.28 (ddd, *J* = 9.0, 6.1, 2.4 Hz, 2H), 3.15–3.03 (m, 3H), 2.83–2.74 (m, 5H), 2.66 (d, *J* = 3.6 Hz, 1H), 1.60 (d, *J* = 10.5 Hz, 1H), 1.25 (dd, *J* = 13.0,

5.9 Hz, 1H); ^{13}C-NMR (100 MHz, $CDCl_3$): δ = 172.56 (2C), 155.55, 149.25, 129.04, 120.94, 106.62, 100.91, 67.48 (2C), 60.39, 51.58, 51.36, 50.80 (2C), 46.28, 45.00, 44.78, 44.45, 42.34, 34.41, 33.75.

Bis(4-fluorobenzyl) (1*S*,2*S*,3*R*,4*S*,4a*S*,9a*S*)-9-methyl-5-morpholino-2,3,4,4a,9,9a-hexahydro-1*H*-1,4-methanocarbazole-2,3-dicarboxylate (2d)

Synthesized according to General Method A using 2-iodo-*N*-methylaniline (93 mg, 0.40 mmol), bis(4-fluorobenzyl) bicyclo[2.2.1]hept-5-ene-2,3-dicarboxylate (239 mg, 0.60 mmol), morpholino benzoate (166 mg, 0.80 mmol), Cs_2CO_3 (521 mg, 1.60 mmol), $Pd(OAc)_2$ (9 mg, 0.04 mmol), and TFP (21 mg, 0.08 mmol) in toluene (3 mL). The crude product was purified by column chromatography (petroleum ether/ethyl acetate = 20/1) to give **2d** (120 mg, 51% yield) as a pale-yellow oil. HRMS (ESI): calcd. for $C_{34}H_{34}F_2N_2O_5$ $[M+H]^+$: 589.2436, found: 589.2492; ^{1}H-NMR (400 MHz, $CDCl_3$): δ = 7.28 (ddd, *J* = 12.9, 9.1, 4.7 Hz, 4H), 7.06–6.96 (m, 5H), 6.22 (d, *J* = 8.0 Hz, 1H), 6.07–5.99 (m, 1H), 5.15–5.03 (m, 2H), 4.91 (dt, *J* = 19.6, 9.1 Hz, 2H), 4.15 (d, *J* = 8.4 Hz, 1H), 3.94–3.83 (m, 2H), 3.80–3.72 (m, 2H), 3.62 (d, *J* = 8.4 Hz, 1H), 3.35–3.27 (m, 2H), 3.21 (dd, *J* = 11.6, 4.8 Hz, 1H), 3.06 (s, 1H), 3.00 (dd, *J* = 11.6, 3.7 Hz, 1H), 2.79 (s, 1H), 2.77 (dd, *J* = 7.0, 4.5 Hz, 2H), 2.63 (s, 3H), 1.59 (d, *J* = 10.6 Hz, 1H), 1.24–1.19 (m, 1H); ^{13}C-NMR (100 MHz, $CDCl_3$): δ = 171.95, 171.89, 162.62 ($^1J_{CF}$ = 247.4), 162.55 ($^1J_{CF}$ = 247.0), 149.42, 149.32, 131.71 ($^3J_{CF}$ = 7.0), 131.67 ($^3J_{CF}$ = 7.1), 130.72, 130.64, 130.51, 130.43, 129.13, 121.00, 115.71, 115.64, 115.55 ($^2J_{CF}$ = 21.6), 115.50, 115.49 ($^2J_{CF}$ = 21.6), 115.42, 67.78, 67.62 (2C), 67.42, 65.66, 65.48, 50.88 (2C), 46.28, 45.14, 44.95, 44.17, 42.31, 34.49; ^{19}F-NMR (377 MHz, $CDCl_3$): δ = -113.19, -113.52.

4-((1*S*,2*S*,3*R*,4*S*,4a*S*,9a*S*)-9-Methyl-2,3-diphenyl-2,3,4,4a,9,9a-hexahydro-1*H*-1,4-methanocarbazol-5-yl)morpholine (2e)

Synthesized according to General Method A using 2-iodo-*N*-methylaniline (93 mg, 0.40 mmol), 5,6-diphenylbicyclo[2.2.1]hept-2-ene (148 mg, 0.60 mmol), morpholino benzoate (166 mg, 0.80 mmol), Cs_2CO_3 (521 mg, 1.60 mmol), $Pd(OAc)_2$ (9 mg, 0.04 mmol), and TFP (21 mg, 0.08 mmol) in toluene (3 mL). The crude product was purified by column chromatography (petroleum ether/ethyl acetate = 20/1) to give **2e** (92 mg, 53% yield) as a pale-yellow oil. HRMS (ESI): calcd. for $C_{30}H_{32}N_2O$ $[M+H]^+$: 437.2515, found: 437.2599; ^{1}H-NMR (400 MHz, $CDCl_3$): δ = 7.09–6.87 (m, 11H), 6.29 (d, *J* = 8.0 Hz, 1H), 6.09 (d, *J* = 7.7 Hz, 1H), 3.85 (d, *J* = 8.3 Hz, 1H), 3.73 (s, 4H), 3.65 (d, *J* = 7.5 Hz, 1H), 3.46 (d, *J* = 9.4 Hz, 1H), 3.29 (d, *J* = 9.5 Hz, 1H), 3.26–3.17 (m, 2H), 3.12 (s, 1H), 2.86 (d, *J* = 17.3 Hz, 5H), 2.71 (s, 1H), 2.18 (d, *J* = 10.8 Hz, 1H), 1.77 (d, *J* = 10.7 Hz, 1H); ^{13}C-NMR (100 MHz, $CDCl_3$): δ = 155.42, 149.34, 142.40, 141.98, 129.36, 128.79 (2C), 128.70 (2C), 127.57 (2C), 127.39 (2C), 125.44, 125.15, 121.51, 107.11, 100.53, 73.66 (2C), 67.38, 53.81 (2C), 51.55, 51.24, 50.47, 47.11, 44.82, 33.17, 31.98.

4-((5a*S*,6*S*,11*S*,11a*S*)-5-Methyl-5a,6,11,11a-tetrahydro-5*H*-6,11-methanobenzo[b]carbazol-1-yl)morpholine (2f)

Synthesized according to General Method A using 2-iodo-*N*-methylaniline (93 mg, 0.40 mmol), 1,4-dihydro-1,4-methanonaphthalene (85 mg, 0.60 mmol), morpholino benzoate (166 mg, 0.80 mmol), Cs_2CO_3 (521 mg, 1.60 mmol), $Pd(OAc)_2$ (9 mg, 0.04 mmol), and TFP (21 mg, 0.08 mmol) in toluene (3 mL). The crude product was purified by column chromatography (petroleum ether/ethyl acetate = 20/1) to give **2f** (53 mg, 40% yield) as a pale-yellow oil. HRMS (ESI): calcd. for $C_{22}H_{24}N_2O$ $[M+H]^+$: 333.1889, found: 333.1955; ^{1}H-NMR (400 MHz, $CDCl_3$):

δ = 7.22 (dd, *J* = 6.7, 5.6 Hz, 2H), 7.16–7.07 (m, 2H), 7.04 (t, *J* = 7.9 Hz, 1H), 6.27 (d, *J* = 8.0 Hz, 1H), 6.08 (d, *J* = 7.8 Hz, 1H), 3.97–3.83 (m, 4H), 3.81 (s, 1H), 3.71 (d, *J* = 8.2 Hz, 1H), 3.52–3.44 (m, 2H), 3.25–3.17 (m, 2H), 2.93–2.84 (m, 5H), 1.84–1.76 (m, 2H); ^{13}C-NMR (100 MHz, $CDCl_3$): δ = 156.60, 149.96, 149.37, 145.99, 129.61, 126.56, 126.19, 122.42, 121.01, 120.53, 107.12, 101.09, 72.22, 67.88 (2C), 51.44 (2C), 50.56, 49.14, 47.02, 43.79, 33.37.

Isopropyl (1*S*,2*R*,4*R*,4a*S*,9a*S*)-9-methyl-5-morpholino-2,3,4,4a,9,9a-hexahydro-1*H*-1,4-methanocarbazole-2-carboxylate + Isopropyl (1*S*,3*S*,4*S*,4a*S*,9a*S*)-9-methyl-5-morpholino-2,3,4,4a,9,9a-hexahydro-1*H*-1,4-methanocarbazole-3-carboxylate (2g)

Synthesized according to General Method A using 2-iodo-*N*-methylaniline (93 mg, 0.40 mmol), dimethyl isopropyl bicyclo[2.2.1]hept-5-ene-2-carboxylate (108 mg, 0.60 mmol), morpholino benzoate (166 mg, 0.80 mmol), Cs_2CO_3 (521 mg, 1.60 mmol), $Pd(OAc)_2$ (9 mg, 0.04 mmol), and TFP (21 mg, 0.08 mmol) in toluene (3 mL). The crude product was purified by column chromatography (petroleum ether/ethyl acetate = 20/1) to give **2g** (62 mg, 42% yield) as a pale-yellow oil. HRMS (ESI): calcd. for $C_{22}H_{30}N_2O_3$ $[M+H]^+$: 371.2256, found: 371.2339; ^{1}H-NMR (400 MHz, $CDCl_3$): δ = 6.99 (dd, *J* = 11.0, 4.8 Hz, 1H), 6.21 (dd, *J* = 14.8, 8.0 Hz, 1H), 6.00 (t, *J* = 7.1 Hz, 1H), 5.01 (hept, *J* = 6.2 Hz, 1H), 3.90–3.75 (m, 4H), 3.55 (dd, *J* = 16.9, 8.4 Hz, 1H), 3.33 (dd, *J* = 38.7, 8.3 Hz, 1H), 3.23–3.10 (m, 2H), 2.89–2.80 (m, 2H), 2.78 (d, *J* = 6.0 Hz, 3H), 2.74 (d, *J* = 3.3 Hz, 1H), 2.60 (s, 1H), 2.46–2.35 (m, 1H), 2.02–1.87 (m, 1H), 1.60 (d, *J* = 9.9 Hz, 1H), 1.42–1.29 (m, 2H), 1.27–1.22 (m, 6H); ^{13}C-NMR (100 MHz, $CDCl_3$): δ = 175.05, 155.12, 129.17, 121.98, 107.23, 100.57, 100.44, 72.33, 67.68, 67.57, 67.52 (2C), 51.33, 49.93, 45.01, 42.99 (2C), 33.48, 33.12, 31.25, 21.90 (2C).

(1*S*,2*R*,4*R*,4a*S*,9a*S*)-9-Methyl-5-morpholino-2,3,4,4a,9,9a-hexahydro-1*H*-1,4-methanocarbazole-2-carbonitrile + (1*S*,3*S*,4*S*,4a*S*,9a*S*)-9-Methyl-5-morpholino-2,3,4,4a,9,9a-hexahydro-1*H*-1,4-methanocarbazole-3-carbonitrile (2h)

Synthesized according to General Method A using 2-iodo-*N*-methylaniline (93 mg, 0.40 mmol), bicyclo[2.2.1]hept-5-ene-2-carbonitrile (71 mg, 0.60 mmol), morpholino benzoate (166 mg, 0.80 mmol), Cs_2CO_3 (521 mg, 1.60 mmol), $Pd(OAc)_2$ (9 mg, 0.04 mmol), and TFP (21 mg, 0.08 mmol) in toluene (3 mL). The crude product was purified by column chromatography (petroleum ether/ethyl acetate = 20/1) to give **2h** (54 mg, 44% yield) as a pale-yellow oil. HRMS (ESI): calcd. for $C_{19}H_{23}N_3O$ $[M+H]^+$: 310.1841, found: 310.1901; ^{1}H-NMR (400 MHz, $CDCl_3$): δ = 7.03 (t, *J* = 7.9 Hz, 1H), 6.28 (d, *J* = 8.0 Hz, 1H), 6.09 (d, *J* = 7.8 Hz, 1H), 3.93–3.86 (m, 3H), 3.85–3.78 (m, 2H), 3.62 (d, *J* = 8.4 Hz, 1H), 3.28–3.20 (m, 2H), 3.00 (s, 1H), 2.87–2.71 (m, 6H), 2.50 (s, 1H), 2.14–2.04 (m, 1H), 1.61 (d, *J* = 10.7 Hz, 1H), 1.45 (dd, *J* = 12.9, 5.2 Hz, 1H), 1.22 (d, *J* = 10.1 Hz, 1H); ^{13}C-NMR (100 MHz, $CDCl_3$): δ = 149.30, 129.59, 122.15, 120.50, 107.46, 101.27, 100.00, 72.23, 67.42 (2C), 51.20 (2C), 45.51, 42.38, 40.83, 33.49 (2C), 30.66, 29.55.

(3a*S*,4*S*,4a*S*,9b*S*,10*S*,10a*R*)-5-Methyl-9-morpholino-2-phenyl-4,4a,5,9b,10,10a-hexahydro-4,10-methanopyrrolo[3,4-b]carbazole-1,3(2*H*,3a*H*)-dione (2i)

Synthesized according to General Method A using 2-iodo-*N*-methylaniline (93 mg, 0.40 mmol), 2-phenyl-3a,4,7,7a-tetrahydro-1*H*-4,7-methanoisoindole-1,3(2*H*)-dione (144 mg, 0.60 mmol), morpholino benzoate (166 mg, 0.80 mmol), Cs_2CO_3 (521 mg, 1.60 mmol), $Pd(OAc)_2$ (9 mg, 0.04 mmol), and TFP (21 mg, 0.08 mmol) in toluene

(3 mL). The crude product was purified by column chromatography (petroleum ether/ethyl acetate = 5/1) to give **2i** (86 mg, 50% yield) as a pale-yellow oil. HRMS (ESI): calcd. for $C_{26}H_{27}N_3O_3$ $[M+H]^+$: 430.2052, found: 430.2100; ^{1}H-NMR (400 MHz, $CDCl_3$): δ = 7.48 (t, *J* = 7.5 Hz, 2H), 7.41 (dd, *J* = 8.4, 6.4 Hz, 1H), 7.25–7.20 (m, 2H), 7.05 (t, *J* = 7.9 Hz, 1H), 6.31 (d, *J* = 8.0 Hz, 1H), 6.12 (d, *J* = 7.8 Hz, 1H), 3.97–3.90 (m, 2H), 3.87–3.80 (m, 2H), 3.73 (d, *J* = 8.4 Hz, 1H), 3.57 (d, *J* = 8.4 Hz, 1H), 3.44–3.40 (m, 1H), 3.39–3.32 (m, 2H), 3.20–3.13 (m, 2H), 3.03 (d, *J* = 5.1 Hz, 1H), 2.88–2.78 (m, 4H), 1.80 (d, *J* = 10.8 Hz, 1H), 1.59 (d, *J* = 10.8 Hz, 1H), 0.45 (s, 1H); ^{13}C-NMR (100 MHz, $CDCl_3$): δ = 176.77, 176.66, 154.91, 149.18, 131.75, 129.73, 129.38 (2C), 128.89, 126.64 (2C), 119.46, 107.23, 101.10, 67.96, 67.29 (2C), 50.84 (2C), 48.07, 46.00 (2C), 44.50, 41.86, 36.99, 33.52.

(3a*S*,4*S*,4a*S*,9b*S*,10*S*,10a*R*)-2-(3-Methoxyphenyl)-5-methyl-9-morpholino-4,4a,5,9b,10,10a-hexahydro-4,10-methanopyrrolo[3,4-b]carbazole-1,3(2*H*,3a*H*)-dione (2j)

Synthesized according to General Method A using 2-iodo-*N*-methylaniline (93 mg, 0.40 mmol), 2-(3-methoxyphenyl)-3a,4,7,7a-tetrahydro-1*H*-4,7-methanoisoindole-1,3(2*H*)-dione (162 mg, 0.60 mmol), morpholino benzoate (166 mg, 0.80 mmol), Cs_2CO_3 (521 mg, 1.60 mmol), $Pd(OAc)_2$ (9 mg, 0.04 mmol), and TFP (21 mg, 0.08 mmol) in toluene (3 mL). The crude product was purified by column chromatography (petroleum ether/ethyl acetate = 5/1) to give **2j** (88 mg, 48% yield) as a pale-yellow oil. HRMS (ESI): calcd. for $C_{27}H_{29}N_3O_4$ $[M+H]^+$: 460.2158, found: 460.2220; ^{1}H-NMR (400 MHz, $CDCl_3$): δ = 7.38 (t, *J* = 8.1 Hz, 1H), 7.04 (t, *J* = 7.9 Hz, 1H), 6.95 (dd, *J* = 8.4, 2.4 Hz, 1H), 6.79 (d, *J* = 7.9 Hz, 1H), 6.74 (s, 1H), 6.28 (d, *J* = 8.1 Hz, 1H), 6.09 (d, *J* = 7.8 Hz, 1H), 3.97–3.88 (m, 2H), 3.86–3.78 (m, 5H), 3.72 (d, *J* = 8.5 Hz, 1H), 3.55 (d, *J* = 8.5 Hz, 1H), 3.40 (t, *J* = 5.6 Hz, 1H), 3.37–3.30 (m, 2H), 3.19–3.11 (m, 2H), 3.00 (d, *J* = 4.8 Hz, 1H), 2.80 (d, *J* = 12.5 Hz, 4H), 1.79 (d, *J* = 10.8 Hz, 1H), 1.58 (d, *J* = 10.8 Hz, 1H), 1.26 (s, 1H); ^{13}C-NMR (100 MHz, $CDCl_3$): δ = 176.71, 176.57, 160.23, 154.83, 149.05, 132.73, 130.15, 129.79, 119.53, 118.85, 114.44, 112.84, 107.43, 101.31, 68.02, 67.25 (2C), 55.46, 50.89 (2C), 48.07, 45.99 (2C), 44.48, 41.92, 36.99, 33.65.

(3a*S*,4*S*,4a*S*,9b*S*,10*S*,10a*R*)-5-Methyl-9-morpholino-2-(3-nitrophenyl)-4,4a,5,9b,10,10a-hexahydro-4,10-methanopyrrolo[3,4-b]carbazole-1,3(2*H*,3a*H*)-dione (2k)

Synthesized according to General Method A using 2-iodo-*N*-methylaniline (93 mg, 0.40 mmol), 2-(3-nitrophenyl)-3a,4,7,7a-tetrahydro-1*H*-4,7-methanoisoindole-1,3(2*H*)-dione (170 mg, 0.60 mmol), morpholino benzoate (166 mg, 0.80 mmol), Cs_2CO_3 (521 mg, 1.60 mmol), $Pd(OAc)_2$ (9 mg, 0.04 mmol), and TFP (21 mg, 0.08 mmol) in toluene (3 mL). The crude product was purified by column chromatography (petroleum ether/ethyl acetate = 5/1) to give **2k** (85 mg, 45% yield) as a pale-yellow oil. HRMS (ESI): calcd. for $C_{26}H_{26}N_4O_5$ $[M+H]^+$: 475.1903, found: 475.1969; ^{1}H-NMR (400 MHz, $CDCl_3$): δ = 8.27 (d, *J* = 7.4 Hz, 1H), 8.19 (s, 1H), 7.67 (q, *J* = 8.3 Hz, 2H), 7.05 (t, *J* = 7.9 Hz, 1H), 6.28 (d, *J* = 8.0 Hz, 1H), 6.10 (d, *J* = 7.8 Hz, 1H), 3.98–3.88 (m, 2H), 3.86–3.77 (m, 2H), 3.72 (d, *J* = 8.4 Hz, 1H), 3.52 (d, *J* = 8.4 Hz, 1H), 3.42 (s, 2H), 3.17–3.09 (m, 2H), 3.03 (d, *J* = 4.0 Hz, 1H), 2.86–2.78 (m, 4H), 1.83 (d, *J* = 10.7 Hz, 1H), 1.61 (d, *J* = 10.8 Hz, 1H), 1.26 (dd, *J* = 12.3, 5.0 Hz, 2H); ^{13}C-NMR (100 MHz, $CDCl_3$): δ = 176.08, 175.90, 154.80, 149.13, 148.57, 132.69, 132.38, 130.15, 129.91, 123.45, 121.84, 119.45, 107.59, 101.41, 68.03, 67.25 (2C), 51.02 (2C), 48.18, 46.03 (2C), 44.55, 42.15, 37.05, 33.61.

4-((3a*S*,4*S*,4a*S*,9b*S*,10*S*,10a*R*)-5-Methyl-9-morpholino-1,3-dioxo-3,3a,4,4a,5,9b,10,10a-octahydro-4,10-methanopyrrolo[3,4-b]carbazol-2(1*H*)-yl)benzonitrile (2l)

Synthesized according to General Method A using 2-iodo-*N*-methylaniline (93 mg, 0.40 mmol), 4-(1,3-dioxo-1,3,3a,4,7,7a-hexahydro-2*H*-4,7-methanoisoindol-2-yl)benzonitrile (159 mg, 0.60 mmol), morpholino benzoate (166 mg, 0.80 mmol), Cs_2CO_3 (521 mg, 1.60 mmol), $Pd(OAc)_2$ (9 mg, 0.04 mmol), and TFP (21 mg, 0.08 mmol) in toluene (3 mL). The crude product was purified by column chromatography (petroleum ether/ethyl acetate = 5/1) to give **2l** (85 mg, 47% yield) as a pale-yellow oil. HRMS (ESI): calcd. for $C_{27}H_{26}N_4O_3$ $[M+H]^+$: 455.2005, found: 455.2065; ^{1}H-NMR (400 MHz, $CDCl_3$): δ = 7.77 (d, *J* = 8.5 Hz, 2H), 7.45 (d, *J* = 8.5 Hz, 2H), 7.04 (t, *J* = 7.9 Hz, 1H), 6.26 (d, *J* = 8.0 Hz, 1H), 6.08 (d, *J* = 7.8 Hz, 1H), 3.96–3.87 (m, 2H), 3.85–3.76 (m, 2H), 3.68 (d, *J* = 8.4 Hz, 1H), 3.49 (d, *J* = 8.5 Hz, 1H), 3.40 (dd, *J* = 12.9, 7.9 Hz, 3H), 3.11 (ddd, *J* = 11.0, 6.1, 2.3 Hz, 2H), 3.01 (d, *J* = 4.6 Hz, 1H), 2.83–2.77 (m, 4H), 1.81 (d, *J* = 10.8 Hz, 1H), 1.59 (d, *J* = 10.8 Hz, 1H), 1.27 (d, *J* = 10.7 Hz, 1H); ^{13}C-NMR (100 MHz, $CDCl_3$): δ = 176.24, 176.11, 155.03, 149.40, 135.81, 133.34 (2C), 130.09, 127.33 (2C), 119.53, 118.19, 112.66, 107.66, 101.47, 68.18, 67.49 (2C), 51.14 (2C), 48.36, 46.26, 46.24, 44.84, 42.24, 37.25, 33.74.

Diisopropyl (1*S*,2*S*,3*R*,4*S*,4a*S*,9a*S*)-9-methyl-5-thiomorpholino-2,3,4,4a,9,9a-hexahydro-1*H*-1,4-methanocarbazole-2,3-dicarboxylate (3a)

Synthesized according to General Method A using 2-iodo-*N*-methylaniline (100 mg, 0.43 mmol), diisopropyl bicyclo[2.2.1]hept-5-ene-2,3-dicarboxylate (172 mg, 0.65 mmol), thiomorpholino benzoate (192 mg, 0.86 mmol), Cs_2CO_3 (560 mg, 1.70 mmol), $Pd(OAC)_2$ (10 mg, 0.04 mmol), and TFP (23 mg, 0.09 mmol) in toluene (2 mL) was stirred at 100 ℃ for 12 h. The crude product was purified by column chromatography (petroleum ether/ethyl acetate = 30/1) to give **3a** (158 mg, 78% yield) as a white solid, mp 125-126 ℃. HRMS (ESI): calcd. for $C_{26}H_{36}N_2O_4S$ $[M+H]^+$: 473.2469, found: 473.2468; ^{1}H-NMR (400 MHz, $CDCl_3$): δ = 6.99 (s, 1H), 6.28 (s, 1H), 6.06 (s, 1H), 5.02 (m, 2H), 4.14 (d, *J* = 8.0 Hz, 1H), 3.89 (t, *J* = 11.1 Hz, 1H), 3.39 (s, 2H), 3.16–3.02 (m, 3H), 3.00–2.92 (m, 2H), 2.92–2.83 (m, 2H), 2.78 (s, 3H), 2.71 (dd, *J* = 23.5, 11.3 Hz, 3H), 1.58 (d, *J* = 10.1 Hz, 2H), 1.33–1.17 (m, 13H); ^{13}C-NMR (100 MHz, $CDCl_3$): δ = 171.49, 171.29, 150.72, 128.90, 122.99, 120.63, 108.12, 101.08, 67.80, 67.57, 53.72 (2C), 46.17 (2C), 45.24 (2C), 44.98, 43.94, 43.19, 34.43, 28.40 (2C), 22.13, 21.96, 21.83, 21.80.

Diisopropyl (1*S*,2*S*,3*R*,4*S*,4a*S*,9a*S*)-9-methyl-5-(1,4-dioxa-8-azaspiro[4.5]decan-8-yl)-2,3,4,4a,9,9a-hexahydro-1*H*-1,4-methanocarbazole-2,3-dicarboxylate (3b)

Synthesized according to General Method A using 2-iodo-*N*-methylaniline (100 mg, 0.43 mmol), diisopropyl bicyclo[2.2.1]hept-5-ene-2,3-dicarboxylate (178 mg, 0.65 mmol), 1,4-dioxa-8-azaspiro[4.5]decan-8-yl benzoate (230 mg, 0.86 mmol), Cs_2CO_3 (560 mg, 1.60 mmol), $Pd(OAC)_2$ (10 mg, 0.04 mmol), and TFP (24 mg, 0.08 mmol) in toluene (2 mL) was stirred at 100 ℃ for 12 h. The crude product was purified by column chromatography (petroleum ether/ethyl acetate = 50/1) to give **3b** (179 mg, 81% yield) as a white oil. HRMS (ESI): calcd. for $C_{29}H_{40}N_2O_6$ $[M+H]^+$: 513.2959, found: 513.2941; ^{1}H-NMR (400 MHz, $CDCl_3$): δ = 6.99–6.93 (m, 1H), 6.27 (d, *J* = 7.9 Hz, 1H), 6.01 (t, *J* = 7.0 Hz, 1H), 5.06–4.96 (m, 2H), 4.14 (dd, *J* = 15.0, 7.8 Hz, 1H), 3.98 (s, 4H), 3.90 (d, *J* = 8.4 Hz, 1H), 3.36–3.22 (m, 2H), 3.04 (t, *J* = 4.5 Hz, 1H), 3.01 (dd, *J* = 6.7, 3.7 Hz, 1H), 2.98 (d, *J* = 3.9 Hz, 1H), 2.96–2.86 (m, 3H), 2.78 (s, 3H), 2.62 (t, *J* = 5.4 Hz, 1H), 1.99–1.87 (m, 2H), 1.86–1.75 (m, 2H), 1.58 (d, *J* = 10.4 Hz, 1H), 1.25 (ddd, *J* = 11.2, 6.1, 3.7 Hz, 14H), 1.19 (d, *J* = 10.5 Hz, 1H); ^{13}C-NMR (100 MHz, $CDCl_3$): δ = 171.49, 171.35, 155.38, 149.78, 128.65, 121.79, 107.58, 107.39, 100.59, 67.67, 67.47, 67.42, 64.17 (2C), 49.03 (2C), 46.13, 45.36, 45.06, 44.34, 42.51, 35.51 (2C), 34.52, 33.73, 22.08, 21.95, 21.82, 21.79.

Diisopropyl (1*S*,2*S*,3*R*,4*S*,4a*S*,9a*S*)-9-methyl-5-(4-phenylpiperidin-1-yl)-2,3,4,4a,9,9a-hexahydro-1*H*-1,4-methanocarbazole-2,3-dicarboxylate (3c)

Synthesized according to General Method A using 2-iodo-*N*-methylaniline (58 mg, 0.25 mmol), diisopropyl bicyclo[2.2.1]hept-5-ene-2,3-dicarboxylate (111 mg, 0.38 mmol), 4-phenylpiperidin-1-yl benzoate (140 mg, 0.50 mmol), Cs_2CO_3 (324 mg, 1.0 mmol), $Pd(OAC)_2$ (8 mg, 0.025 mmol), and TFP (17 mg, 0.05 mmol) in toluene (2 mL) was stirred at 100 ℃ for 12 h. The crude product was purified by column chromatography (petroleum ether/ethyl acetate = 50/1) to give **3c** (89 mg, 67% yield) as a pale-yellow solid, mp 120-121 ℃. HRMS (ESI): calcd. for $C_{33}H_{42}N_2O_4$ $[M+H]^+$: 531.3217, found: 531.3219; ^{1}H-NMR (400 MHz, $CDCl_3$): δ = 7.36–7.26 (m, 4H), 7.20 (t, *J* = 6.7 Hz, 1H), 6.99 (t, *J* = 7.9 Hz, 1H), 6.30 (d, *J* = 8.0 Hz, 1H), 6.03 (d, *J* = 7.7 Hz, 1H), 5.08–4.90 (m, 2H), 4.16 (d, *J* = 8.4 Hz, 1H), 3.99 (d, *J* = 8.5 Hz, 1H), 3.66 (d, *J* = 11.6 Hz, 1H), 3.28 (d, *J* = 10.5 Hz, 1H), 3.11 (s, 1H), 3.02 (dd, *J* = 12.2, 7.7 Hz, 2H), 2.95 (dd, *J* = 21.2, 9.7 Hz, 1H), 2.80 (d, *J* = 7.1 Hz, 3H), 2.65 (s, 1H), 2.62–2.51 (m, 2H), 2.09–1.74 (m, 5H), 1.60 (d, *J* = 10.5 Hz, 1H), 1.30–1.15 (m, 13H); ^{13}C-NMR (100 MHz, $CDCl_3$): δ =171.61, 171.39, 165.20, 157.98, 155.47, 150.34, 146.73, 128.69, 128.30, 126.93, 125.98, 107.45, 100.46, 67.70 (2C), 67.28, 52.63, 51.17, 46.21, 45.50, 45.04, 44.53, 42.78, 42.57, 34.59, 34.12, 34.00, 33.68, 22.01, 21.97, 21.86, 21.79.

Diisopropyl (1*S*,2*S*,3*R*,4*S*,4a*S*,9a*S*)-5-(dimethylamino)-9-methyl-2,3,4,4a,9,9a-hexahydro-1*H*-1,4-methanocarbazole-2,3-dicarboxylate (3d)

Synthesized according to General Method A using 2-iodo-*N*-methylaniline (100 mg, 0.43 mmol), diisopropyl bicyclo[2.2.1]hept-5-ene-2,3-dicarboxylate (172 mg, 0.65 mmol), *O*-benzoyl-*N*,*N*-dimethylhydroxylamine (142 mg, 0.86 mmol), Cs_2CO_3 (560 mg, 1.70 mmol), $Pd(OAC)_2$ (10 mg, 0.04 mmol), and TFP (23 mg, 0.09 mmol) in toluene (2 mL) was stirred at 100 ℃ for 12 h. The crude product was purified by column chromatography (petroleum ether/ethyl acetate = 20/1) to give **3d** (94 mg, 53% yield) as a white oil. HRMS (ESI): calcd. for $C_{24}H_{34}N_2O_4$ $[M+H]^+$: 415.2591, found: 415.2584; ^{1}H-NMR (400 MHz, $CDCl_3$): δ = 6.96 (t, *J* = 7.9 Hz, 1H), 6.18 (d, *J* = 7.9 Hz, 1H), 5.97 (d, *J* = 7.7 Hz, 1H), 5.09–4.97 (m, 2H), 4.06 (t, *J* = 12.6 Hz, 1H), 4.00 (d, *J* = 8.4 Hz, 1H), 3.01 (d, *J* = 10.7 Hz, 2H), 2.95 (s, 1H), 2.79 (s, 9H), 2.62 (d, *J* = 15.9 Hz, 1H), 1.59 (d, *J* = 10.4 Hz, 1H), 1.25 (dd, *J* = 9.0, 4.0 Hz, 12H), 1.19 (d, *J* = 10.5 Hz, 1H); ^{13}C-NMR (100 MHz, $CDCl_3$): δ = 171.59, 171.29, 155.42, 150.01, 128.43, 119.08, 105.77, 99.23, 67.45, 67.40, 66.99, 46.43 (2C), 45.05, 44.80, 44.71, 42.68, 42.51, 34.15, 33.58, 21.86, 21.84, 21.70, 21.61.

Diisopropyl (1*S*,2*S*,3*R*,4*S*,4a*S*,9a*S*)-9-methyl-5-(methyl(4-methylbenzyl)amino)-2,3,4,4a,9,9a-hexahydro-1*H*-1,4-methanocarbazole-2,3-dicarboxylate (3e)

Synthesized according to General Method A using 2-iodo-*N*-methylaniline (100 mg, 0.43 mmol), diisopropyl bicyclo[2.2.1]hept-5-ene-2,3-dicarboxylate (172 mg, 0.65 mmol), *O*-benzoyl-*N*-methyl-*N*-(4-methylbenzyl)hydroxylamine (220 mg, 0.86 mmol), Cs_2CO_3 (560 mg, 1.70 mmol), $Pd(OAC)_2$ (10 mg, 0.04 mmol), and TFP (23 mg, 0.09 mmol) in toluene (2 mL) was stirred at 100 ℃ for 12 h. The crude product was purified by column chromatography (petroleum ether/ethyl acetate = 10/1) to give **3e** (163 mg, 75% yield) as a yellow oil. HRMS (ESI): calcd. for $C_{31}H_{40}N_2O_4$ $[M+H]^+$: 505.3061, found: 505.3046; ^{1}H-NMR (400 MHz, $CDCl_3$): δ =7.18 (d, *J* = 7.8 Hz, 2H), 7.10 (d, *J* = 7.8 Hz, 2H), 6.95 (t, *J* = 7.9 Hz, 1H), 6.22 (d, *J* = 8.0 Hz, 1H), 6.00 (d, *J* = 7.7 Hz,

1H), 5.00 (ddd, *J* = 24.8, 12.5, 6.2 Hz, 2H), 4.40 (d, *J* = 14.2 Hz, 1H), 4.10 (dd, *J* = 20.2, 8.4 Hz, 2H), 4.02 (d, *J* = 14.3 Hz, 1H), 3.11 (dd, *J* = 11.7, 4.1 Hz, 1H), 3.05 (s, 1H), 2.99 (dd, *J* = 11.6, 4.2 Hz, 1H), 2.82 (d, *J* = 9.7 Hz, 3H), 2.66 (s, 4H), 2.31 (d, *J* = 9.5 Hz, 3H), 1.64 (d, *J* = 10.3 Hz, 1H), 1.22 (ddd, *J* = 33.7, 18.7, 6.2 Hz, 13H); ^{13}C-NMR (100 MHz, $CDCl_3$): δ = 171.64, 171.47, 155.57, 149.81, 136.13, 135.97, 128.81 (2C), 128.62, 128.29 (2C), 120.53, 107.93, 99.66, 67.69, 67.59, 67.03, 58.25, 46.60, 45.25, 44.84, 43.49, 40.43, 34.34 (2C), 33.55, 22.00, 21.90, 21.85, 21.74, 21.06

Diisopropyl (1*S*,2*S*,3*R*,4*S*,4a*S*,9a*S*)-9-methyl-5-(methyl(pyridin-3-ylmethyl)amino)-2,3,4,4a,9,9a-hexahydro-1*H*-1,4-methanocarbazole-2,3-dicarboxylate (3f)

Synthesized according to General Method A using 2-iodo-*N*-methylaniline (100 mg, 0.43 mmol), diisopropyl bicyclo[2.2.1]hept-5-ene-2,3-dicarboxylate (172 mg, 0.65 mmol), *O*-benzoyl-*N*-methyl-*N*-(pyridin-3-ylmethyl)hydroxylamine (208 mg, 0.86 mmol), Cs_2CO_3 (560 mg, 1.70 mmol), $Pd(OAC)_2$ (10 mg, 0.04 mmol), and TFP (23 mg, 0.09 mmol) in toluene (2 mL) was stirred at 100 ℃ for 12 h. The crude product was purified by column chromatography (petroleum ether/ethyl acetate = 5/1) to give **3f** (186 mg, 88% yield) as a yellow oil. HRMS (ESI): calcd. for $C_{29}H_{37}N_3O_4$ $[M+H]^+$: 492.2857, found: 492.2853; ^{1}H-NMR (400 MHz, $CDCl_3$): δ = 8.53 (s, 1H), 8.47 (d, *J* = 3.7 Hz, 1H), 7.62 (d, *J* = 7.8 Hz, 1H), 7.20 (dd, *J* = 7.7, 4.8 Hz, 1H), 6.94 (t, *J* = 7.9 Hz, 1H), 6.19 (d, *J* = 8.0 Hz, 1H), 6.01 (d, *J* = 7.7 Hz, 1H), 5.07–4.91 (m, 2H), 4.47 (d, *J* = 14.5 Hz, 1H), 4.12 (d, *J* = 8.4 Hz, 1H), 4.03 (dd, *J* = 24.2, 11.5 Hz, 2H), 3.03 (dd, *J* = 7.9, 6.4 Hz, 2H), 2.97 (d, *J* = 9.8 Hz, 1H), 2.79 (s, 3H), 2.66 (s, 4H), 1.62 (d, *J* = 10.5 Hz, 1H), 1.29–1.13 (m, 13H); ^{13}C-NMR (100 MHz, $CDCl_3$): δ = 171.74, 171.53, 155.77, 150.01, 149.33, 148.25, 136.37, 134.57, 128.92, 123.37, 121.26, 108.09, 100.43, 67.81, 67.70, 67.47, 56.19, 46.66, 45.27, 44.99, 44.68, 43.53, 40.93, 34.41, 33.65, 22.11 (2C), 21.92, 21.90.

Diisopropyl (1*S*,2*S*,3*R*,4*S*,4a*S*,9a*S*)-9-methyl-5-(methyl(thiophen-2-ylmethyl)amino)-2,3,4,4a,9,9a-hexahydro-1*H*-1,4-methanocarbazole-2,3-dicarboxylate (3g)

Synthesized according to General Method A using 2-iodo-*N*-methylaniline (100 mg, 0.43 mmol), diisopropyl bicyclo[2.2.1]hept-5-ene-2,3-dicarboxylate (172 mg, 0.65 mmol), *O*-benzoyl-*N*-methyl-*N*-(thiophen-2-ylmethyl)hydroxylamine (215 mg, 0.86 mmol), Cs_2CO_3 (564 mg, 1.70 mmol), $Pd(OAC)_2$ (12 mg, 0.04 mmol), and TFP (24 mg, 0.09 mmol) in toluene (2 mL) was stirred at 100 ℃ for 12 h. The crude product was purified by column chromatography (petroleum ether/ethyl acetate = 50/1) to give **3g** (132 mg, 62% yield) as a pale-yellow solid, mp 120-121 ℃. HRMS (ESI): calcd. for $C_{28}H_{36}N_2O_4S$ $[M+H]^+$: 497.2469, found: 497.2459; ^{1}H-NMR (400 MHz, $CDCl_3$): δ = 7.16 (dd, *J* = 9.3, 4.6 Hz, 1H), 7.00–6.87 (m, 3H), 6.20 (d, *J* = 8.0 Hz, 1H), 6.02 (d, *J* = 7.7 Hz, 1H), 5.01 (ddq, *J* = 18.8, 12.5, 6.2 Hz, 2H), 4.63 (d, *J* = 14.7 Hz, 1H), 4.25 (d, *J* = 14.7 Hz, 1H), 4.14–4.04 (m, 2H), 3.14–3.07 (m, 2H), 3.01 (dd, *J* = 12.0, 4.1 Hz, 1H), 2.82 (s, 3H), 2.71 (s, 3H), 2.67 (d, *J* = 3.7 Hz, 1H), 1.63 (d, *J* = 10.5 Hz, 1H), 1.29–1.16 (m, 13H); ^{13}C-NMR (100 MHz, $CDCl_3$): δ = 171.67, 171.44, 155.51, 148.87, 142.56, 128.58, 126.15, 125.38, 124.41, 120.97, 107.91, 100.06, 67.63, 67.57, 67.07, 53.20, 46.52, 45.25, 44.81, 44.75, 43.19, 40.53, 34.39, 33.47, 21.97, 21.94, 21.83, 21.72

Diisopropyl (1*S*,2*S*,3*R*,4*S*,4aS,9a*S*)-5-(2-(methoxycarbonyl)phenyl)-9-methyl-2,3,4,4a,9,9a-hexahydro-1*H*-1,4-methanocarbazole-2,3-dicarboxylate (3h)

Synthesized according to General Method B using 2-iodo-*N*-methylaniline (50 mg, 0.22 mmol), diisopropyl bicyclo[2.2.1]hept-5-ene-2,3-dicarboxylate (115 mg, 0.43 mmol), methyl 2-bromobenzoate (93 mg, 0.43 mmol), K_2CO_3 (119 mg, 0.86 mmol), Pd(OAC)$_2$ (5 mg, 0.02 mmol), and TFP (10 mg, 0.02 mmol) in DMF (2 mL) was stirred at 100 ℃ for 12 h. The crude product was purified by column chromatography (petroleum ether/ethyl acetate = 30/1) to give **3h** (76 mg, 70% yield) as a dark brown black solid, mp 123-124 ℃. HRMS (ESI): calcd. for $C_{30}H_{35}NO_6$ $[M+H]^+$: 506.2537, found: 506.2524; ^{1}H-NMR (400 MHz, $CDCl_3$): δ = 7.84 (d, *J* = 7.6 Hz, 1H), 7.45 (t, *J* = 7.3 Hz, 1H), 7.36 (t, *J* = 7.6 Hz, 1H), 7.31–7.26 (m, 1H), 6.98 (t, *J* = 7.6 Hz, 1H), 6.22 (t, *J* = 7.7 Hz, 2H), 4.98 (dt, *J* = 12.4, 6.2 Hz, 1H), 4.80 (dt, *J* = 12.3, 6.2 Hz, 1H), 4.26 (d, *J* = 8.4 Hz, 1H), 3.57 (s, 3H), 3.44 (d, *J* = 2.6 Hz, 1H), 2.88 (s, 3H), 2.81 (dd, *J* = 18.6, 6.7 Hz, 2H), 2.62 (s, 1H), 2.14 (s, 1H), 1.65 (d, *J* = 10.2 Hz, 1H), 1.23–1.00 (m, 13H); ^{13}C-NMR (100 MHz, $CDCl_3$): δ = 171.28 (2C), 171.06, 153.96, 141.56, 138.13, 131.12 (2C), 129.59, 127.46 (2C), 126.74 (2C), 117.01, 103.48, 67.47, 67.44, 66.73, 51.73, 46.06, 45.13, 44.56 (2C), 44.37, 33.97, 33.27, 21.89, 21.84, 21.69, 21.55.

Diisopropyl (1*S*,2*S*,3*R*,4*S*,4aS,9a*S*)-5-(3-(methoxycarbonyl)phenyl)-9-methyl-2,3,4,4a,9,9a-hexahydro-1*H*-1,4-methanocarbazole-2,3-dicarboxylate (3i)

Synthesized according to General Method B using 2-iodo-*N*-methylaniline (50 mg, 0.22 mmol), diisopropyl bicyclo[2.2.1]hept-5-ene-2,3-dicarboxylate (115 mg, 0.43 mmol), methyl 3-bromobenzoate (93 mg, 0.43 mmol), K_2CO_3 (119 mg, 0.86 mmol), Pd(OAC)$_2$ (5 mg, 0.02 mmol), and TFP (10 mg, 0.02 mmol) in DMF (2 mL) was stirred at 100 ℃ for 12 h. The crude product was purified by column chromatography (petroleum ether/ethyl acetate = 30/1) to give **3i** (66 mg, 62% yield) as a yellow-green solid, mp 110-112 ℃. HRMS (ESI): calcd. for $C_{30}H_{35}NO_6$ $[M+H]^+$: 506.2537, found: 506.2525; ^{1}H-NMR (400 MHz, $CDCl_3$): δ = 8.08 (s, 1H), 7.98 (d, *J* = 7.8 Hz, 1H), 7.68 (d, *J* = 7.7 Hz, 1H), 7.45 (t, *J* = 7.7 Hz, 1H), 7.10 (t, *J* = 7.7 Hz, 1H), 6.51 (d, *J* = 7.1 Hz, 1H), 6.32 (s, 1H), 5.06–4.94 (m, 1H), 4.94–4.81 (m, 1H), 4.26 (d, *J* = 8.3 Hz, 1H), 4.17 (d, *J* = 8.4 Hz, 1H), 3.91 (s, 3H), 2.95–2.83 (m, 4H), 2.78 (dd, *J* = 11.9, 4.1 Hz, 1H), 2.68 (d, *J* = 15.1 Hz, 1H), 2.03 (d, *J* = 3.5 Hz, 1H), 1.58 (d, *J* = 10.5 Hz, 1H), 1.28–1.06 (m, 13H); ^{13}C-NMR (100 MHz, $CDCl_3$): δ = 171.35, 171.20, 167.32, 154.52, 141.45, 137.80, 132.98 (2C), 130.32, 129.76, 128.50, 128.43, 128.03, 117.52, 104.19, 67.86, 67.73, 67.37, 52.16 (2C), 46.19, 45.34, 44.95, 44.80, 44.01, 34.26, 22.09, 22.05, 21.89, 21.84.

Diisopropyl (1*S*,2*S*,3*R*,4*S*,4aS,9a*S*)-5-(4-(methoxycarbonyl)phenyl)-9-methyl-2,3,4,4a,9,9a-hexahydro-1*H*-1,4-methanocarbazole-2,3-dicarboxylate (3j)

Synthesized according to General Method B using 2-iodo-*N*-methylaniline (50 mg, 0.22 mmol), diisopropyl bicyclo[2.2.1]hept-5-ene-2,3-dicarboxylate (115 mg, 0.43 mmol), methyl 2-bromobenzoate (93 mg, 0.43 mmol), K_2CO_3 (119 mg, 0.86 mmol), Pd(OAC)$_2$ (5 mg, 0.02 mmol), and TFP (10 mg, 0.02 mmol) in DMF (2 mL) was stirred at 100 ℃ for 12 h. The crude product was purified by column chromatography (petroleum ether/ethyl acetate = 30/1) to give **3j** (68 mg, 63% yield) as a dark brown oil. HRMS (ESI): calcd. for $C_{30}H_{35}NO_6$ $[M+H]^+$: 506.2537, found: 506.2527; ^{1}H-NMR (400 MHz, $CDCl_3$): δ = 8.05 (d, *J* = 8.3 Hz, 2H), 7.54 (d, *J* = 8.3 Hz, 2H), 7.09 (t, *J* = 7.7 Hz, 1H), 6.50 (d, *J* = 7.5 Hz, 1H), 6.28 (d, *J* = 7.8 Hz, 1H), 5.05–4.88 (m, 2H), 4.30 (d, *J* = 8.5 Hz, 1H), 4.21 (d, *J* = 8.5 Hz, 1H), 3.92 (s, 3H), 2.93–2.85 (m, 4H), 2.81 (dd, *J* = 11.9, 4.1 Hz, 1H), 2.64 (d, *J* = 3.4 Hz, 1H), 2.03 (d, *J* = 3.5 Hz, 1H), 1.56 (d, *J* = 10.5 Hz, 1H), 1.28–1.14 (m, 12H), 1.10 (d, *J* = 10.5 Hz, 1H); ^{13}C-NMR (100 MHz,

$CDCl_3$): δ = 171.18, 171.15, 166.97, 154.55, 145.83, 137.33, 129.54 (2C), 128.38, 128.31 (2C), 128.29, 127.85, 117.29, 104.23, 67.73, 67.67, 67.00, 51.93, 46.02, 45.20, 44.76, 44.58, 43.88, 34.10, 33.27, 21.92, 21.88, 21.77, 21.65.

Diisopropyl (1*S*,2*S*,3*R*,4*S*,4aS,9a*S*)-9-methyl-5-(3-nitrophenyl)-2,3,4,4a,9,9a-hexahydro-1*H*-1,4-methanocarbazole-2,3-dicarboxylate (3k)

Synthesized according to General Method B using 2-iodo-*N*-methylaniline (50 mg, 0.22 mmol), diisopropyl bicyclo[2.2.1]hept-5-ene-2,3-dicarboxylate (115 mg, 0.43 mmol), 1-bromo-3-nitrobenzene (86 mg, 0.43 mmol), K_2CO_3 (119 mg, 0.86 mmol), $Pd(OAC)_2$ (5 mg, 0.02 mmol), and TFP (10 mg, 0.02 mmol) in DMF (2 mL) was stirred at 100 ℃ for 12 h. The crude product was purified by column chromatography (petroleum ether/ethyl acetate = 30/1) to give **3k** (80 mg, 75% yield) as a yellow solid, mp 140-141 ℃. HRMS (ESI): calcd. for $C_{28}H_{32}N_2O_6$ $[M+H]^+$: 493.2333, found: 493.2325; ^{1}H-NMR (400 MHz, $CDCl_3$): δ = 8.31 (t, *J* = 1.9 Hz, 1H), 8.16 (ddd, *J* = 8.2, 2.3, 1.0 Hz, 1H), 7.92–7.85 (m, 1H), 7.57 (t, *J* = 7.9 Hz, 1H), 7.11 (t, *J* = 7.8 Hz, 1H), 6.52 (dd, *J* = 7.6, 0.7 Hz, 1H), 6.31 (d, *J* = 7.8 Hz, 1H), 5.06 – 4.95 (m, 1H), 4.94–4.81 (m, 1H), 4.36 (d, *J* = 8.4 Hz, 1H), 4.03 (d, *J* = 8.4 Hz, 1H), 2.99 (dd, *J* = 11.8, 4.6 Hz, 1H), 2.84 (s, 3H), 2.71 (dd, *J* = 11.8, 4.0 Hz, 1H), 2.66 (d, *J* = 4.4 Hz, 1H), 1.98 (d, *J* = 3.2 Hz, 1H), 1.59 (d, *J* = 9.0 Hz, 1H), 1.19 (ddd, *J* = 35.2, 17.9, 6.4 Hz, 13H); ^{13}C-NMR (100 MHz, $CDCl_3$): δ = 171.12, 170.94, 154.68, 148.27, 142.54, 136.21, 134.70, 129.14, 128.67, 128.16, 123.55, 121.58, 117.36, 104.71, 67.86, 67.64, 67.56, 46.07, 45.07, 44.83, 44.67, 43.53, 33.99, 33.35, 26.89, 21.95, 21.77, 21.70.

Diisopropyl (1*S*,2*S*,3*R*,4*S*,4aS,9a*S*)-9-methyl-5-(3-(methylsulfonyl)phenyl)-2,3,4,4a,9,9a-hexahydro-1*H*-1,4-methanocarbazole-2,3-dicarboxylate (3l)

Synthesized according to General Method B using 2-iodo-*N*-methylaniline (50 mg, 0.22 mmol), diisopropyl bicyclo[2.2.1]hept-5-ene-2,3-dicarboxylate (115 mg, 0.43 mmol), 1-bromo-3-(methylsulfonyl)benzene (101 mg, 0.43 mmol), K_2CO_3 (119 mg, 0.86 mmol), $Pd(OAC)_2$ (5 mg, 0.02 mmol), and TFP (10 mg, 0.02 mmol) in DMF (2 mL) was stirred at 100 ℃ for 12 h. The crude product was purified by column chromatography (petroleum ether/ethyl acetate = 10/1) to give **3l** (71 mg, 64% yield) as a green-brown oil. HRMS (ESI): calcd. for $C_{29}H_{35}NO_6S$ $[M+H]^+$: 526.2258, found: 526.2251; ^{1}H-NMR (400 MHz, $CDCl_3$): δ = 8.02 (t, *J* = 1.5 Hz, 1H), 7.88 (d, *J* = 7.8 Hz, 1H), 7.82 (d, *J* = 7.8 Hz, 1H), 7.60 (q, *J* = 8.0 Hz, 1H), 7.10 (q, *J* = 8.1 Hz, 1H), 6.47 (t, *J* = 9.1 Hz, 1H), 6.29 (t, *J* = 8.7 Hz, 1H), 5.04–4.95 (m, 1H), 4.90–4.82 (m, 1H), 4.38 (d, *J* = 8.4 Hz, 1H), 3.92 (d, *J* = 8.4 Hz, 1H), 3.17 (d, *J* = 11.4 Hz, 3H), 3.02 (dt, *J* = 12.2, 6.1 Hz, 1H), 2.81 (d, *J* = 5.2 Hz, 3H), 2.71–2.62 (m, 2H), 2.00 (d, *J* = 3.0 Hz, 1H), 1.59 (d, *J* = 10.5 Hz, 1H), 1.26–1.10 (m, 13H); ^{13}C-NMR (100 MHz, $CDCl_3$): δ =171.06, 171.01, 154.53, 142.41, 140.51, 136.69, 133.69, 129.15, 128.47, 128.12, 127.40, 125.45, 117.75, 104.59, 67.80, 67.74, 67.37, 45.96, 44.99, 44.85, 44.60, 44.25, 43.42, 33.92, 33.38, 21.98, 21.92, 21.77, 21.62.

Diisopropyl (1*S*,2*S*,3*R*,4*S*,4aS,9a*S*)-9-methyl-5-(4-(methylsulfonyl)phenyl)-2,3,4,4a,9,9a-hexahydro-1*H*-1,4-methanocarbazole-2,3-dicarboxylate (3m)

Synthesized according to General Method B using 2-iodo-*N*-methylaniline (50 mg, 0.22 mmol), diisopropyl bicyclo[2.2.1]hept-5-ene-2,3-dicarboxylate (115 mg, 0.43 mmol), 1-bromo-4-(methylsulfonyl)benzene (102 mg, 0.43 mmol), K_2CO_3 (119 mg, 0.86 mmol), $Pd(OAC)_2$ (5 mg, 0.02 mmol), and TFP (10 mg, 0.02 mmol) in DMF (2 mL) was stirred at 100 ℃ for 12 h. The crude product was purified by column chromatography (petroleum

ether/ethyl acetate = 10/1) to give **3m** (70 mg, 68% yield) as a white solid, mp 150-151 ℃. HRMS (ESI): calcd. for $C_{29}H_{35}NO_6S$ $[M+H]^+$: 526.2258, found: 526.2254; ^{1}H-NMR (400 MHz, $CDCl_3$): δ = 7.95 (d, *J* = 8.2 Hz, 2H), 7.69 (d, *J* = 8.2 Hz, 2H), 7.10 (t, *J* = 7.7 Hz, 1H), 6.49 (d, *J* = 7.6 Hz, 1H), 6.30 (d, *J* = 7.8 Hz, 1H), 4.96 (ddp, *J* = 31.1, 12.4, 6.2 Hz, 2H), 4.37 (d, *J* = 8.4 Hz, 1H), 4.06 (d, *J* = 8.4 Hz, 1H), 3.06 (d, *J* = 11.0 Hz, 3H), 3.01–2.93 (m, 1H), 2.84 (s, 3H), 2.74 (dd, *J* = 11.8, 4.0 Hz, 1H), 2.65 (d, *J* = 3.3 Hz, 1H), 1.98 (d, *J* = 2.6 Hz, 1H), 1.56 (d, *J* = 10.4 Hz, 1H), 1.19 (ddd, *J* = 20.0, 13.3, 6.7 Hz, 12H), 1.14–1.08 (m, 1H); ^{13}C-NMR (100 MHz, $CDCl_3$): δ =171.09, 171.03, 154.57, 146.62, 138.48, 136.48, 129.32 (2C), 128.55, 128.15, 127.33 (2C), 117.31, 104.71, 67.78, 67.55, 67.43, 45.92, 45.09, 44.77, 44.55, 44.50, 43.62, 33.98, 33.31, 22.10, 21.91, 21.69 (2C).

Diisopropyl (1*S*,2*S*,3*R*,4*S*,4aS,9a*S*)-5-(3-cyanophenyl)-9-methyl-2,3,4,4a,9,9a-hexahydro-1*H*-1,4-methanocarbazole-2,3-dicarboxylate (3n)

Synthesized according to General Method B using 2-iodo-*N*-methylaniline (50 mg, 0.22 mmol), diisopropyl bicyclo[2.2.1]hept-5-ene-2,3-dicarboxylate (115 mg, 0.43 mmol), 3-bromobenzonitrile (78 mg, 0.43 mmol), K_2CO_3 (119 mg, 0.86 mmol), $Pd(OAC)_2$ (5 mg, 0.02 mmol), and TFP (10 mg, 0.02 mmol) in DMF (2 mL) was stirred at 100 ℃ for 12 h. The crude product was purified by column chromatography (petroleum ether/ethyl acetate = 8/1) to give **3n** (59 mg, 58% yield) as a yellow oil. HRMS (ESI): calcd. for $C_{29}H_{32}N_2O_4$ $[M+H]^+$: 473.2435, found: 473.2411; ^{1}H-NMR (400 MHz, $CDCl_3$): δ = 7.76 (d, *J* = 7.5 Hz, 2H), 7.59 (d, *J* = 7.7 Hz, 1H), 7.49 (t, *J* = 7.6 Hz, 1H), 7.09 (t, *J* = 7.8 Hz, 1H), 6.46 (d, *J* = 7.6 Hz, 1H), 6.30 (d, *J* = 7.9 Hz, 1H), 5.05–4.91 (m, 2H), 4.35 (d, *J* = 8.4 Hz, 1H), 4.03 (d, *J* = 8.4 Hz, 1H), 2.99 (dd, *J* = 11.9, 4.5 Hz, 1H), 2.82 (d, *J* = 11.8 Hz, 3H), 2.74 (dd, *J* = 11.8, 4.0 Hz, 1H), 2.65 (d, *J* = 4.0 Hz, 1H), 1.99 (d, *J* = 3.1 Hz, 1H), 1.58 (d, *J* = 10.5 Hz, 1H), 1.26–1.14 (m, 13H); ^{13}C-NMR (100 MHz, $CDCl_3$): δ = 171.12, 170.98, 154.63, 142.11, 136.29, 133.00, 132.06, 130.32, 129.02, 128.60, 128.06, 118.94, 117.40, 112.40, 104.62, 67.83, 67.61, 67.56, 46.00, 45.08, 44.83, 44.62, 43.52, 34.00, 33.38, 22.06, 21.95, 21.75, 21.73.

Diisopropyl (1*S*,2*S*,3*R*,4*S*,4aS,9a*S*)-5-(4-cyanophenyl)-9-methyl-2,3,4,4a,9,9a-hexahydro-1*H*-1,4-methanocarbazole-2,3-dicarboxylate (3o)

Synthesized according to General Method B using 2-iodo-*N*-methylaniline (50 mg, 0.22 mmol), diisopropyl bicyclo[2.2.1]hept-5-ene-2,3-dicarboxylate (115 mg, 0.43 mmol), 4-bromobenzonitrile (78 mg, 0.43 mmol), K_2CO_3 (119 mg, 0.86 mmol), $Pd(OAC)_2$ (5 mg, 0.02 mmol), and TFP (10 mg, 0.02 mmol) in DMF (2 mL) was stirred at 100 ℃ for 12 h. The crude product was purified by column chromatography (petroleum ether/ethyl acetate = 40/1) to give **3o** (80 mg, 71% yield) as a yellow oil. HRMS (ESI): calcd. for $C_{29}H_{32}N_2O_4$ $[M+H]^+$: 473.2435, found: 473.2429; ^{1}H-NMR (400 MHz, $CDCl_3$): δ = 7.75–7.65 (d, *J* = 8.3 Hz, 2H), 7.62 (d, *J* = 8.3 Hz, 2H), 7.10 (t, *J* = 7.8 Hz, 1H), 6.49 (d, *J* = 7.6 Hz, 1H), 6.30 (d, *J* = 7.8 Hz, 1H), 5.09–4.87 (m, 2H), 4.39 (d, *J* = 8.5 Hz, 1H), 4.05 (d, *J* = 8.5 Hz, 1H), 2.99 (dd, *J* = 11.8, 4.5 Hz, 1H), 2.84 (s, 3H), 2.75 (dd, *J* = 11.9, 4.0 Hz, 1H), 2.66 (d, *J* = 4.0 Hz, 1H), 1.97 (d, *J* = 4.0 Hz, 1H), 1.56 (d, *J* = 10.5 Hz, 1H), 1.29–1.17 (m, 12H), 1.13 (d, *J* = 10.5 Hz, 1H); ^{13}C-NMR (100 MHz, $CDCl_3$): δ = 171.07, 171.04, 154.59, 145.64, 136.55, 132.04 (2C), 129.20 (2C), 128.59, 128.04, 119.11, 117.25, 110.29, 104.76, 67.80, 67.51, 67.45, 45.94, 45.07, 44.78, 44.51, 43.55, 34.00, 33.34, 22.03, 21.91, 21.71 (2C).

Diisopropyl (1*S*,2*S*,3*R*,4*S*,4a*S*,9a*S*)-5-hexyl-9-methyl-2,3,4,4a,9,9a-hexahydro-1*H*-1,4-methanocarbazole-2,3-dicarboxylate (3p)

A mixture of diisopropyl bicyclo[2.2.1]hept-5-ene-2,3-dicarboxylate (159 mg, 0.60 mmol), 2-iodo-*N*-methylaniline (93 mg, 0.40 mmol), 1-bromopentane (302 mg, 2.00 mmol), Cs_2CO_3 (521 mg, 1.60 mmol), $Pd(OAc)_2$ (9 mg , 0.04 mmol), and PPh_3 (21 mg, 0.08 mmol) in DMF (3 mL) was stirred at 100 ℃ for 12 h. The crude product was purified by column chromatography (petroleum ether/ethyl acetate = 30/1) to give **3p** (66 mg, 25% yield) as a colorless oil. HRMS (ESI): calcd. for $C_{27}H_{39}NO_4$ $[M+H]^+$: 442.2879, found: 442.2941; ^{1}H-NMR (400 MHz, $CDCl_3$): δ = 6.98 (t, *J* = 7.7 Hz, 1H), 6.44 (d, *J* = 6.7 Hz, 1H), 6.16 (s, 1H), 5.13–4.99 (m, 2H), 3.09 (dd, *J* = 11.6, 4.5 Hz, 1H), 2.98 (dd, *J* = 11.6, 3.8 Hz, 1H), 2.77 (s, 3H), 2.66 (s, 2H), 2.60 (t, *J* = 7.8 Hz, 2H), 1.70 (d, *J* = 10.4 Hz, 1H), 1.68–1.53 (m, 3H), 1.40–1.34 (m, 4H), 1.30–1.23 (m, 14H), 0.90 (t, *J* = 7.0 Hz, 3H); ^{13}C-NMR (100 MHz, $CDCl_3$): δ = 171.45, 171.44, 154.40, 139.34, 128.82, 128.01, 116.91, 102.89, 67.74, 67.69, 47.22, 45.37, 44.88, 44.80, 44.14, 34.05, 32.01 (2C), 31.84, 30.42 (2C), 22.72, 22.10, 22.00, 21.87, 21.83, 14.09.

Diisopropyl (1*S*,2*S*,3*R*,4*S*,4a*S*,9a*S*)-5-benzoyl-9-methyl-2,3,4,4a,9,9a-hexahydro-1*H*-1,4-methanocarbazole-2,3-dicarboxylate (3q)

Synthesized according to General Method C using benzoic anhydride (181 mg, 0.80 mmol), 2-iodo-*N*-methylaniline (93 mg, 0.40 mmol), TFP (19 mg, 0.08 mmol), $PdCl_2$ (7 mg, 0.04 mmol), Cs_2CO_3 (521 mg, 1.60 mmol) and diisopropyl bicyclo[2.2.1]hept-5-ene-2,3-dicarboxylate (159 mg, 0.60 mmol) in DME (3 mL). The crude product was purified by column chromatography (petroleum ether/ethyl acetate = 20/1) to give **3q** (133 mg, 70% yield) as a yellow oil. HRMS (ESI): calcd. for $C_{29}H_{33}NO_5$ $[M+H]^+$: 476.2359, found: 476.2423; ^{1}H-NMR (400 MHz, $CDCl_3$): δ = 7.83–7.76 (m, 2H), 7.57–7.50 (m, 1H), 7.42 (t, *J* = 7.6 Hz, 2H), 7.04 (t, *J* = 7.8 Hz, 1H), 6.54 (d, *J* = 7.6 Hz, 1H), 6.38 (d, *J* = 7.8 Hz, 1H), 4.98 (ddt, *J* = 28.4, 12.5, 6.2 Hz, 2H), 4.35 (d, *J* = 8.3 Hz, 1H), 4.00 (d, *J* = 8.5 Hz, 1H), 3.05 (dd, *J* = 11.8, 4.5 Hz, 1H), 2.88 (d, *J* = 3.4 Hz, 3H), 2.85 (t, *J* = 5.9 Hz, 1H), 2.67 (d, *J* = 4.0 Hz, 2H), 1.64 (d, *J* = 10.5 Hz, 1H), 1.22 (ddd, *J* = 8.9, 8.3, 4.9 Hz, 13H); ^{13}C-NMR (100 MHz, $CDCl_3$): δ = 197.19, 171.76, 171.48, 138.20, 134.95, 134.85, 132.75, 131.09, 130.31 (2C), 128.41 (2C), 127.75, 117.71, 107.72, 68.34, 67.80, 67.73, 47.02, 46.70, 45.24, 45.20, 45.08, 34.34, 28.69, 22.28, 22.14, 21.96, 21.93.

Diisopropyl (1*S*,2*S*,3*R*,4*S*,4a*S*,9a*S*)-9-methyl-5-(2-methylbenzoyl)-2,3,4,4a,9,9a-hexahydro-1*H*-1,4-methanocarbazole-2,3-dicarboxylate (3r)

Synthesized according to General Method C using 2-methylbenzoic anhydride (203 mg, 0.80 mmol), 2-iodo-*N*-methylaniline (93 mg, 0.40 mmol), TFP (19 mg, 0.08 mmol), $PdCl_2$ (7 mg, 0.04 mmol), Cs_2CO_3 (521 mg, 1.60 mmol) and diisopropyl bicyclo[2.2.1]hept-5-ene-2,3-dicarboxylate (159 mg, 0.60 mmol) in DME (3 mL). The crude product was purified by column chromatography (petroleum ether/ethyl acetate = 20/1) to give **3r** (107 mg, 55% yield) as a yellow oil. HRMS (ESI): calcd. for $C_{30}H_{35}NO_5$ $[M+H]^+$: 490.2515, found: 490.2571; ^{1}H-NMR (400 MHz, $CDCl_3$): δ = 7.32 (d, *J* = 7.5 Hz, 1H), 7.30–7.25 (m, 1H), 7.21 (dd, *J* = 10.5, 7.6 Hz, 2H), 6.98 (t, *J* = 7.8 Hz, 1H), 6.45 (d, *J* = 7.7 Hz, 1H), 6.37 (d, *J* = 7.8 Hz, 1H), 5.10–5.00 (m, 2H), 4.41 (s, 1H), 4.18 (d, *J* = 8.4 Hz, 1H), 3.18 (dd, *J* = 11.7, 4.6 Hz, 1H), 2.95 (s, 1H), 2.91–2.85 (m, 4H), 2.68 (d, *J* = 3.0 Hz, 1H), 2.32 (s, 3H), 1.63 (d, *J* = 10.5 Hz, 1H), 1.36 (d, *J* = 6.3 Hz, 2H), 1.24 (ddd, *J* = 19.4, 10.1, 5.9 Hz, 11H); ^{13}C-NMR (100 MHz, $CDCl_3$): δ

= 198.98, 171.77, 171.59, 155.49, 139.75, 137.18, 134.42, 132.28, 130.96, 130.13, 129.14, 127.88, 125.15, 119.45, 107.90, 68.38, 67.64, 67.49, 47.01, 46.20, 46.19, 45.25, 45.16, 34.38, 33.54, 22.20, 22.13, 21.90, 21.82, 20.27.

Diisopropyl (1*S*,2*S*,3*R*,4*S*,4a*S*,9a*S*)-9-methyl-5-(3-methylbenzoyl)-2,3,4,4a,9,9a-hexahydro-1*H*-1,4-methanocarbazole-2,3-dicarboxylate (3s)

Synthesized according to General Method C using 3-methylbenzoic anhydride (203 mg, 0.80 mmol), 2-iodo-*N*-methylaniline (93 mg, 0.40 mmol), TFP (19 mg, 0.08 mmol), $PdCl_2$ (7 mg, 0.04 mmol), Cs_2CO_3 (521 mg, 1.60 mmol) and diisopropyl bicyclo[2.2.1]hept-5-ene-2,3-dicarboxylate (159 mg, 0.60 mmol) in DME (3 mL). The crude product was purified by column chromatography (petroleum ether/ethyl acetate = 20/1) to give **3s** (104 mg, 53% yield) as a yellow oil. HRMS (ESI): calcd. for $C_{30}H_{35}NO_5$ $[M+H]^+$: 490.2515, found: 490.2573; ^{1}H-NMR (400 MHz, $CDCl_3$): δ = 7.62 (s, 1H), 7.57 (d, *J* = 7.4 Hz, 1H), 7.33 (dt, *J* = 15.0, 7.5 Hz, 2H), 7.04 (t, *J* = 7.7 Hz, 1H), 6.53 (d, *J* = 7.6 Hz, 1H), 6.37 (d, *J* = 7.9 Hz, 1H), 5.06–4.91 (m, 2H), 4.34 (d, *J* = 8.5 Hz, 1H), 3.99 (d, *J* = 8.4 Hz, 1H), 3.06 (dd, *J* = 11.7, 4.4 Hz, 1H), 2.90–2.83 (m, 4H), 2.67 (d, *J* = 4.5 Hz, 2H), 2.39 (s, 3H), 1.65 (d, *J* = 10.5 Hz, 1H), 1.25–1.19 (m, 13H); ^{13}C-NMR (100 MHz, $CDCl_3$): δ = 197.17, 171.55, 171.26, 155.02, 137.98, 137.94, 134.93, 133.32, 130.70, 130.40, 128.05, 127.49, 127.45, 117.32, 106.64, 68.09, 67.55, 67.44, 46.79, 46.46, 45.00 (2C), 44.87, 34.13, 33.41, 22.05, 21.91, 21.71 (2C), 21.35.

Diisopropyl (1*S*,2*S*,3*R*,4*S*,4a*S*,9a*S*)-9-methyl-5-(4-methylbenzoyl)-2,3,4,4a,9,9a-hexahydro-1*H*-1,4-methanocarbazole-2,3-dicarboxylate (3t)

Synthesized according to General Method C using 4-methylbenzoic anhydride (203 mg, 0.80 mmol), 2-iodo-*N*-methylaniline (93 mg, 0.40 mmol), TFP (19 mg, 0.08 mmol), $PdCl_2$ (7 mg, 0.04 mmol), Cs_2CO_3 (521 mg, 1.60 mmol) and diisopropyl bicyclo[2.2.1]hept-5-ene-2,3-dicarboxylate (159 mg, 0.60 mmol) in DME (3 mL). The crude product was purified by column chromatography (petroleum ether/ethyl acetate = 20/1) to give **3t** (117 mg, 60% yield) as a yellow oil. HRMS (ESI): calcd. for $C_{30}H_{35}NO_5$ $[M+H]^+$: 490.2515, found: 490.2574; ^{1}H-NMR (400 MHz, $CDCl_3$): δ = 7.71 (d, *J* = 8.1 Hz, 2H), 7.22 (d, *J* = 7.9 Hz, 2H), 7.03 (t, *J* = 7.7 Hz, 1H), 6.52 (d, *J* = 7.5 Hz, 1H), 6.36 (d, *J* = 7.8 Hz, 1H), 4.97 (ddq, *J* = 37.9, 12.5, 6.2 Hz, 2H), 4.34 (d, *J* = 8.4 Hz, 1H), 3.96 (d, *J* = 8.3 Hz, 1H), 3.04 (dd, *J* = 11.7, 4.4 Hz, 1H), 2.91–2.81 (m, 4H), 2.64 (d, *J* = 4.4 Hz, 2H), 2.42 (d, *J* = 9.6 Hz, 3H), 1.64 (d, *J* = 10.5 Hz, 1H), 1.25–1.17 (m, 13H); ^{13}C-NMR (100 MHz, $CDCl_3$): δ = 196.73, 171.53, 171.24, 154.93, 143.37, 135.26, 135.19, 130.39, 130.29 (2C), 128.93 (2C), 127.48, 117.02, 106.51, 68.08, 67.56, 67.42, 46.76, 46.47, 44.99, 44.90, 44.85, 34.09, 33.44, 22.04, 21.90, 21.70 (2C), 21.66.

Diisopropyl (1*S*,2*S*,3*R*,4*S*,4a*S*,9a*S*)-5-(2-methoxybenzoyl)-9-methyl-2,3,4,4a,9,9a-hexahydro-1*H*-1,4-methanocarbazole-2,3-dicarboxylate (3u)

Synthesized according to General Method C using (isopropyl carbonic) 2-methoxybenzoic anhydride (191 mg, 0.80 mmol), 2-iodo-*N*-methylaniline (93 mg, 0.40 mmol), TFP (19 mg, 0.08 mmol), $PdCl_2$ (7 mg, 0.04 mmol), Cs_2CO_3 (521 mg, 1.60 mmol) and diisopropyl bicyclo[2.2.1]hept-5-ene-2,3-dicarboxylate (159 mg, 0.60 mmol) in DME (3 mL). The crude product was purified by column chromatography (petroleum ether/ethyl acetate = 20/1) to give **3u** (103 mg, 51% yield) as a yellow oil. HRMS (ESI): calcd. for $C_{30}H_{35}NO_6$ $[M+H]^+$: 506.2464, found: 506.2524; ^{1}H-NMR (400 MHz, $CDCl_3$): δ = 7.45–7.37 (m, 1H), 7.31 (dd, *J* = 7.5, 1.5 Hz, 1H), 6.96 (dt, *J* = 11.2, 8.0 Hz, 3H), 6.48 (d, *J* = 7.5 Hz, 1H), 6.37 (d, *J* = 6.6 Hz, 1H), 5.18–4.93 (m, 2H), 4.40 (d, *J* = 8.4 Hz, 1H), 4.19

(d, *J* = 8.4 Hz, 1H), 3.67 (s, 3H), 3.18 (dd, *J* = 11.7, 4.6 Hz, 1H), 2.97 (d, *J* = 4.1 Hz, 1H), 2.91–2.84 (m, 4H), 2.69 (s, 1H), 1.62 (d, *J* = 10.5 Hz, 1H), 1.36 (d, *J* = 6.3 Hz, 2H), 1.29–1.20 (m, 11H); ^{13}C-NMR (100 MHz, $CDCl_3$): δ = 197.12, 171.80, 171.63, 157.65, 157.23, 135.13, 131.73, 131.57, 130.65, 129.56, 127.72, 120.45, 119.22, 111.71, 107.80, 68.38, 67.63, 67.58, 55.84, 46.97, 46.25, 46.06, 45.37, 45.15, 34.41, 29.84, 22.20, 22.12, 21.96, 21.84.

Diisopropyl (1*S*,2*S*,3*R*,4*S*,4a*S*,9a*S*)-5-(3-methoxybenzoyl)-9-methyl-2,3,4,4a,9,9a-hexahydro-1*H*-1,4-methanocarbazole-2,3-dicarboxylate (3v)

Synthesized according to General Method C using 3-methoxybenzoic anhydride (229 mg, 0.80 mmol), 2-iodo-*N*-methylaniline (93 mg, 0.40 mmol), TFP (19 mg, 0.08 mmol), $PdCl_2$ (7 mg, 0.04 mmol), Cs_2CO_3 (521 mg, 1.60 mmol) and diisopropyl bicyclo[2.2.1]hept-5-ene-2,3-dicarboxylate (159 mg, 0.60 mmol) in DME (3 mL). The crude product was purified by column chromatography (petroleum ether/ethyl acetate = 20/1) to give **3v** (117 mg, 58% yield) as a yellow oil. HRMS (ESI): calcd. for $C_{30}H_{35}NO_6$ $[M+H]^+$: 506.2464, found: 506.2522; ^{1}H-NMR (400 MHz, $CDCl_3$): δ = 7.39 (d, *J* = 2.4 Hz, 1H), 7.32 (d, *J* = 5.0 Hz, 2H), 7.09 (s, 1H), 7.03 (d, *J* = 7.8 Hz, 1H), 6.55 (d, *J* = 7.6 Hz, 1H), 6.37 (d, *J* = 7.8 Hz, 1H), 5.07–4.91 (m, 2H), 4.33 (d, *J* = 8.4 Hz, 1H), 3.99 (t, *J* = 10.6 Hz, 1H), 3.84 (d, *J* = 6.9 Hz, 3H), 3.05 (dd, *J* = 11.8, 4.5 Hz, 1H), 2.92–2.83 (m, 4H), 2.66 (d, *J* = 3.8 Hz, 2H), 1.64 (d, *J* = 10.5 Hz, 1H), 1.25–1.19 (m, 13H); ^{13}C-NMR (100 MHz, $CDCl_3$): δ = 196.71, 171.53, 171.24, 159.53, 154.98, 139.31, 134.73, 130.87, 129.13, 127.49, 123.22, 119.13, 117.40, 113.92, 106.82, 68.09, 67.57, 67.50, 55.43, 46.79, 46.47, 44.97 (2C), 44.86, 34.11, 33.45, 22.05, 21.92, 21.72, 21.71.

Diisopropyl (1*S*,2*S*,3*R*,4*S*,4a*S*,9a*S*)-5-(4-methoxybenzoyl)-9-methyl-2,3,4,4a,9,9a-hexahydro-1*H*-1,4-methanocarbazole-2,3-dicarboxylate (3w)

Synthesized according to General Method C using 4-methoxybenzoic anhydride (229 mg, 0.80 mmol), 2-iodo-*N*-methylaniline (93 mg, 0.40 mmol), TFP (19 mg, 0.08 mmol), $PdCl_2$ (7 mg, 0.04 mmol), Cs_2CO_3 (521 mg, 1.60 mmol) and diisopropyl bicyclo[2.2.1]hept-5-ene-2,3-dicarboxylate (159 mg, 0.60 mmol) in DME (3 mL). The crude product was purified by column chromatography (petroleum ether/ethyl acetate = 20/1) to give **3w** (129 mg, 64% yield) as a yellow oil. HRMS (ESI): calcd. for $C_{30}H_{35}NO_6$ $[M+H]^+$: 506.2464, found: 506.2518; ^{1}H-NMR (400 MHz, $CDCl_3$): δ = 7.83–7.78 (m, 2H), 7.04 (t, *J* = 7.7 Hz, 1H), 6.94–6.87 (m, 2H), 6.50 (d, *J* = 7.4 Hz, 1H), 6.35 (d, *J* = 7.8 Hz, 1H), 4.95 (ddt, *J* = 43.1, 12.5, 6.3 Hz, 2H), 4.32 (d, *J* = 8.4 Hz, 1H), 3.91 (d, *J* = 8.9 Hz, 1H), 3.86 (s, 3H), 3.02 (dd, *J* = 11.7, 4.4 Hz, 1H), 2.90–2.81 (m, 4H), 2.63 (dd, *J* = 6.4, 4.8 Hz, 2H), 1.68–1.61 (m, 2H), 1.20 (ddd, *J* = 9.1, 6.2, 4.7 Hz, 12H); ^{13}C-NMR (100 MHz, $CDCl_3$): δ = 195.82, 171.52, 171.23, 163.36, 154.87, 135.60, 132.48 (2C), 130.59, 129.90, 127.52, 116.56, 113.49 (2C), 106.24, 68.04, 67.56, 67.42, 55.47, 46.74, 46.46, 44.97, 44.82, 44.80, 34.08, 33.43, 22.04, 21.88, 21.70 (2C).

Diisopropyl (1*S*,2*S*,3*R*,4*S*,4a*S*,9a*S*)-5-(4-fluorobenzoyl)-9-methyl-2,3,4,4a,9,9a-hexahydro-1*H*-1,4-methanocarbazole-2,3-dicarboxylate (3x)

Synthesized according to General Method C using 4-fluorobenzoic anhydride (210 mg, 0.80 mmol), 2-iodo-*N*-methylaniline (93 mg, 0.40 mmol), TFP (19 mg, 0.08 mmol), $PdCl_2$ (7 mg, 0.04 mmol), Cs_2CO_3 (521 mg, 1.60 mmol) and diisopropyl bicyclo[2.2.1]hept-5-ene-2,3-dicarboxylate (159 mg, 0.60 mmol) in DME (3 mL). The crude product was purified by column chromatography (petroleum ether/ethyl acetate = 15/1) to give **3x** (109 mg, 55% yield) as a yellow oil. HRMS (ESI): calcd. for $C_{29}H_{32}FNO_5$ $[M+H]^+$: 494.2265, found: 494.2317; ^{1}H-NMR

(400 MHz, $CDCl_3$): δ = 7.83 (dd, *J* = 8.8, 5.5 Hz, 2H), 7.09 (dd, *J* = 19.3, 10.6 Hz, 3H), 6.51 (d, *J* = 7.5 Hz, 1H), 6.37 (d, *J* = 7.8 Hz, 1H), 5.07–4.90 (m, 2H), 4.32 (d, *J* = 8.4 Hz, 1H), 3.98 (d, *J* = 8.4 Hz, 1H), 3.04 (dd, *J* = 11.7, 4.4 Hz, 1H), 2.93–2.83 (m, 4H), 2.65 (dd, *J* = 8.6, 4.0 Hz, 2H), 1.64 (d, *J* = 10.5 Hz, 1H), 1.25–1.18 (m, 13H); ^{13}C-NMR (100 MHz, $CDCl_3$): δ = 195.51, 171.48, 171.20, 165.52 ($^1J_{CF}$ = 253.9), 155.06, 134.56, 134.26, 134.23, 132.67 ($^3J_{CF}$ = 9.1), 130.65, 127.63 (2C), 116.98, 115.33 ($^2J_{CF}$ = 21.9), 106.78, 68.05, 67.61, 67.49, 46.75, 46.51, 44.96, 44.87, 44.83, 34.08, 33.36, 22.04, 21.89, 21.73, 21.71; ^{19}F-NMR (377 MHz, $CDCl_3$): δ = -106.08.

Diisopropyl (1*S*,2*S*,3*R*,4*S*,4a*S*,9a*S*)-5-(4-chlorobenzoyl)-9-methyl-2,3,4,4a,9,9a-hexahydro-1*H*-1,4-methanocarbazole-2,3-dicarboxylate (3y)

Synthesized according to General Method C using 4-chlorobenzoic anhydride (235 mg, 0.80 mmol), 2-iodo-*N*-methylaniline (93 mg, 0.40 mmol), TFP (19mg, 0.08 mmol), $PdCl_2$ (7 mg, 0.04 mmol), Cs_2CO_3 (521 mg, 1.60 mmol) and diisopropyl bicyclo[2.2.1]hept-5-ene-2,3-dicarboxylate (159 mg, 0.60 mmol) in DME (3 mL). The crude product was purified by column chromatography (petroleum ether/ethyl acetate = 15/1) to give **3y** (124 mg, 61% yield) as a yellow oil. HRMS (ESI): calcd. for $C_{29}H_{32}ClNO_5$ $[M+H]^+$: 510.1969, found: 510.2042; ^{1}H-NMR (400 MHz, $CDCl_3$): δ = 7.74 (d, *J* = 7.8 Hz, 2H), 7.40 (d, *J* = 8.3 Hz, 2H), 7.04 (t, *J* = 7.7 Hz, 1H), 6.50 (d, *J* = 7.6 Hz, 1H), 6.37 (d, *J* = 7.9 Hz, 1H), 5.07–4.89 (m, 2H), 4.32 (d, *J* = 8.5 Hz, 1H), 3.99 (d, *J* = 8.4 Hz, 1H), 3.09–2.97 (m, 1H), 2.88 (s, 4H), 2.65 (d, *J* = 9.3 Hz, 2H), 1.63 (d, *J* = 10.5 Hz, 1H), 1.22 (dd, *J* = 13.5, 6.1 Hz, 13H); ^{13}C-NMR (100 MHz, $CDCl_3$): δ = 195.73, 171.46, 171.20, 138.98, 136.31, 134.24, 131.45 (3C), 130.95, 128.55 (3C), 127.69, 107.16, 68.11, 67.63, 67.56, 46.74, 46.50, 44.95, 44.94, 44.81, 34.09, 33.50, 22.04, 21.90, 21.74, 21.7.

Diisopropyl (1*S*,2*S*,3*R*,4*S*,4a*S*,9a*S*)-9-methyl-5-(4-(trifluoromethyl)benzoyl)-2,3,4,4a,9,9a-hexahydro-1*H*-1,4-methanocarbazole-2,3-dicarboxylate (3z)

Synthesized according to General Method C using 4-(trifluoromethyl)benzoic anhydride (290 mg, 0.80 mmol), 2-iodo-*N*-methylaniline (93 mg, 0.40 mmol), TFP (19 mg, 0.08 mmol), $PdCl_2$ (7 mg, 0.04 mmol), Cs_2CO_3 (521 mg, 1.60 mmol) and diisopropyl bicyclo[2.2.1]hept-5-ene-2,3-dicarboxylate (159 mg, 0.60 mmol) in DME (3 mL). The crude product was purified by column chromatography (petroleum ether/ethyl acetate = 10/1) to give **3z** (113 mg, 52% yield) as a yellow oil. HRMS (ESI): calcd. for $C_{30}H_{32}F_3NO_5$ $[M+H]^+$: 544.2233, found: 544.2301; ^{1}H-NMR (400 MHz, $CDCl_3$): δ = 7.87 (d, *J* = 8.1 Hz, 2H), 7.70 (d, *J* = 8.2 Hz, 2H), 7.08 (t, *J* = 7.7 Hz, 1H), 6.58 (d, *J* = 7.5 Hz, 1H), 6.48 (d, *J* = 7.8 Hz, 1H), 5.01 (ddd, *J* = 23.2, 10.5, 6.3 Hz, 2H), 4.37 (d, *J* = 8.0 Hz, 1H), 4.09 (d, *J* = 8.3 Hz, 1H), 3.14 (s, 1H), 3.10–3.05 (m, 1H), 2.91 (d, *J* = 3.2 Hz, 3H), 2.74 (s, 1H), 2.67 (d, *J* = 4.2 Hz, 1H), 1.64 (d, *J* = 10.6 Hz, 1H), 1.24 (ddd, *J* = 17.3, 10.4, 6.4 Hz, 13H); ^{13}C-NMR (100 MHz, $CDCl_3$): δ = 195.81, 171.47, 171.21, 155.26, 141.21, 133.64 ($^2J_{CF}$ = 32.8), 133.78, 133.51, 131.61, 130.16 (2C), 127.76, 125.22 ($^3J_{CF}$ = 3.7), 125.94 ($^1J_{CF}$ = 268.55), 117.72, 107.39, 68.11, 67.63, 67.52, 46.76, 46.50, 45.10, 44.96, 44.85, 34.10, 33.30, 22.05, 21.90, 21.76, 21.71; ^{19}F-NMR (377 MHz, $CDCl_3$): δ = -62.95.

Diisopropy (1*S*,2*S*,3*R*,4*S*,4a*S*,9a*S*)-7-fluoro-9-methyl-5-morpholino-2,3,4,4a,9,9a-hexahydro-1*H*-1,4-methanocarbazole-2,3-dicarboxylate (4a)

Synthesized according to General Method A using 5-fluoro-2-iodo-*N*-methylaniline (100 mg, 0.40 mmol), diisopropyl bicyclo[2.2.1]hept-5-ene-2,3-dicarboxylate (55 mg, 0.60 mmol), morpholino benzoate (166 mg, 0.80 mmol), Cs_2CO_3 (521 mg, 1.6 mmol), $Pd(OAc)_2$ (9 mg, 0.04 mmol), and PPh_3 (21 mg, 0.08 mmol) in toluene (2

mL) was stirred at 100 ℃ for 12 h. The crude product was purified by column chromatography (petroleum ether/ethyl acetate = 30/1) to give **4a** (121 mg, 64% yield) as a white solid, mp 120-121 ℃. HRMS (ESI): calcd. for $C_{26}H_{35}FN_2O_5$ $[M+H]^+$: 474.2530, found: 474.2591; ^{1}H-NMR (400 MHz, $CDCl_3$): δ = 5.95–5.86 (d, 1H), 5.73 (d, J_{HF} = 9.6 Hz, 1H), 5.05–4.94 (m, 2H), 4.14 (d, *J* = 8.4 Hz, 1H), 3.92–3.74 (m, 5H), 3.37–3.28 (m, 2H), 3.10 (dd, *J* = 11.7, 4.7 Hz, 1H), 2.98 (d, *J* = 2.5 Hz, 1H), 2.91 (dd, *J* = 11.7, 3.9 Hz, 1H), 2.78 (ddd, *J* = 9.4, 6.3, 2.5 Hz, 2H), 2.74 (s, 3H), 2.67 (d, *J* = 4.0 Hz, 1H), 1.57 (d, *J* = 10.6 Hz, 1H), 1.27–1.18 (m, 13H); ^{13}C-NMR (100 MHz, $CDCl_3$): δ = 170.49, 170.21, 163.7 ($^1J_{CF}$ = 240.0), 155.2 ($^3J_{CF}$ = 10.3), 148.50 ($^3J_{CF}$ = 10.7), 114.68, 91.93 ($^2J_{CF}$ = 25.9), 87.26 ($^2J_{CF}$ = 26.8), 67.15, 66.86, 66.49, 66.39 (2C), 49.35 (2C), 45.01, 44.17, 43.81, 42.62, 41.07, 33.17, 32.14, 21.08, 20.95, 20.82, 20.75.

Diisopropyl (1*S*,2*S*,3*R*,4*S*,4a*S*,9a*S*)-7-chloro-9-methyl-5-morpholino-2,3,4,4a,9,9a-hexahydro-1*H*-1,4-methanocarbazole-2,3-dicarboxylate (4b)

Synthesized according to General Method A using 5-chloro-2-iodo-*N*-methylaniline (50 mg, 0.19 mmol), diisopropyl bicyclo[2.2.1]hept-5-ene-2,3-dicarboxylate(26 mg, 0.28 mmol), morpholino benzoate (78 mg, 0.37 mmol), Cs_2CO3 (244 mg, 0.75 mmol), $Pd(OAc)_2$ (4 mg, 0.02 mmol), and PPh_3 (10 mg, 0.04 mmol) in toluene (2 mL) was stirred at 100 ℃ for 12 h. The crude product was purified by column chromatography (petroleum ether/ethyl acetate = 40/1) to give **4b** (60 mg, 65% yield) as a yellow solid, mp 110-112 ℃. HRMS (ESI): calcd. for $C_{26}H_{35}ClN_2O_5$ $[M+H]^+$: 491.2307, found: 491.2292; ^{1}H-NMR (400 MHz, $CDCl_3$): δ = 6.17 (s, 1H), 5.98 (s, 1H), 5.00 (ddt, *J* = 12.5, 9.1, 6.3 Hz, 2H), 4.15 (d, *J* = 8.4 Hz, 1H), 3.91–3.82 (m, 3H), 3.81–3.75 (m, 2H), 3.36–3.28 (m, 2H), 3.11 (dd, *J* = 11.7, 4.7 Hz, 1H), 2.99 (d, *J* = 3.9 Hz, 1H), 2.93–2.88 (m, 1H), 2.82–2.75 (m, 2H), 2.74 (s, 3H), 2.66 (d, *J* = 4.1 Hz, 1H), 1.56 (d, *J* = 10.6 Hz, 1H), 1.27–1.19 (m, 13H); ^{13}C-NMR (100 MHz, $CDCl_3$): δ = 171.46, 171.18, 156.06, 149.40, 134.40, 119.24, 106.70, 100.68, 67.95, 67.88, 67.51, 67.39 (2C), 50.44 (2C), 45.99, 45.16, 44.80, 43.70, 42.02, 34.19, 33.08, 22.08, 21.95, 21.82, 21.74.

Diisopropyl (1*S*,2*S*,3*R*,4*S*,4a*S*,9a*S*)-7,9-dimethyl-5-morpholino-2,3,4,4a,9,9a-hexahydro-1*H*-1,4-methanocarbazole-2,3-dicarboxylate (4c)

Synthesized according to General Method A using 5-methyl-2-iodo-*N*-methylaniline (100 mg, 0.38 mmol), diisopropyl bicyclo[2.2.1]hept-5-ene-2,3-dicarboxylate (53 mg, 0.57 mmol),morpholino benzoate (157 mg, 0.76 mmol), Cs_2CO_3 (495 mg, 1.52 mmol), $Pd(OAc)_2$ (8 mg, 0.04 mmol), and PPh_3 (20mg, 0.08 mmol) in toluene (2 mL) was stirred at 100 ℃ for 12 h. The crude product was purified by column chromatography (petroleum ether/ethyl acetate = 30/1) to give **4c** (122 mg, 68% yield) as a pale-yellow solid, mp 133-134 ℃. HRMS (ESI): calcd. for $C_{26}H_{35}FN_2O_5$ $[M+H]^+$: 471.2853, found: 471.2838; ^{1}H-NMR (400 MHz, $CDCl_3$): δ = 6.05 (s, 1H), 5.89 (s, 1H), 5.06–4.95 (m, 2H), 4.15 (d, *J* = 8.4 Hz, 1H), 3.88 (dd, *J* = 8.8, 6.1 Hz, 2H), 3.79 (d, *J* = 7.9 Hz, 3H), 3.38–3.29 (m, 2H), 3.10 (dd, *J* = 11.7, 4.5 Hz, 1H), 3.01 (s, 1H), 2.92 (dd, *J* = 11.7, 3.6 Hz, 1H), 2.84–2.77 (m, 2H), 2.76 (s, 3H), 2.64 (s, 1H), 2.23 (d, *J* = 10.4 Hz, 3H), 1.59 (d, *J* = 10.4 Hz, 1H), 1.28–1.19 (m, 13H); ^{13}C-NMR (100 MHz, $CDCl_3$): δ = 171.53, 171.30, 155.55, 148.92, 138.79, 118.22, 107.33, 101.76, 68.01, 67.72, 67.57 (2C), 67.36, 50.69 (2C), 46.15, 45.19, 45.02, 43.83, 42.24, 34.29, 33.74, 22.07, 21.93, 21.81, 21.80, 21.74.

Diisopropyl (1*S*,2*S*,3*R*,4*S*,4a*S*,9a*S*)-7-methoxy-9-methyl-5-morpholino-2,3,4,4a,9,9a-hexahydro-1*H*-1,4-methanocarbazole-2,3-dicarboxylate (4d)

Synthesized according to General Method A using 2-iodo-5-methoxy-*N*-methylaniline (55 mg, 0.21 mmol), diisopropyl bicyclo[2.2.1]hept-5-ene-2,3-dicarboxylate (29 mg, 0.31 mmol),morpholino benzoate (87 mg, 0.42 mmol), Cs_2CO3 (274 mg, 0.84 mmol), $Pd(OAc)_2$ (5 mg, 0.02 mmol), and PPh_3 (11 mg, 0.04 mmol) in toluene (2 mL) was stirred at 100 ℃ for 12 h. The crude product was purified by column chromatography (petroleum ether/ethyl acetate = 10/1) to give **4d** (51 mg, 50% yield) as a yellow solid, mp 107-108 ℃. HRMS (ESI): calcd. for $C_{27}H_{38}N_2O_6$ $[M+H]^+$: 487.2803, found: 487.2781; ^{1}H-NMR (400 MHz, $CDCl_3$): δ = 5.77 (d, *J* = 1.8 Hz, 1H), 5.62 (dd, *J* = 9.8, 1.9 Hz, 1H), 4.99 (ddq, *J* = 12.5, 9.2, 6.3 Hz, 2H), 4.10 (d, *J* = 8.4 Hz, 1H), 3.87 (ddt, *J* = 11.4, 7.8, 3.9 Hz, 2H), 3.79 (ddd, *J* = 14.2, 7.0, 3.9 Hz, 3H), 3.75 (s, 3H), 3.37–3.28 (m, 2H), 3.09 (dd, *J* = 11.6, 4.7 Hz, 1H), 2.98 (d, *J* = 2.8 Hz, 1H), 2.94–2.88 (m, 1H), 2.80 (ddd, *J* = 9.2, 5.8, 2.4 Hz, 2H), 2.76 (s, 3H), 2.63 (d, *J* = 4.1 Hz, 1H), 1.57 (d, *J* = 10.5 Hz, 1H), 1.23 (ddd, *J* = 12.0, 6.3, 3.0 Hz, 13H); ^{13}C-NMR (100 MHz, $CDCl_3$): δ = 171.52, 171.28, 161.32, 156.33, 149.50, 113.16, 92.29, 86.96, 68.15, 67.72, 67.50 (2C), 67.36, 55.14, 50.45 (2C), 46.06, 45.16, 44.90, 43.57, 42.22, 34.18, 33.48, 22.06, 21.93, 21.79, 21.74.

Diisopropyl (1*S*,2*S*,3*R*,4*S*,4a*S*,9a*S*)-9-methyl-5-morpholino-7-(trifluoromethyl)-2,3,4,4a,9,9a-hexahydro-1*H*-1,4-methanocarbazole-2,3-dicarboxylate (4e)

Synthesized according to General Method A using 2-iodo-*N*-methyl-5-(trifluoromethyl) aniline (100 mg, 0.35 mmol), diisopropyl bicyclo[2.2.1]hept-5-ene-2,3-dicarboxylate (48 mg, 0.52 mmol), morpholino benzoate (144 mg, 0.70 mmol), Cs_2CO_3 (453 mg, 1.40 mmol), $Pd(OAc)_2$ (8 mg, 0.04 mmol), and PPh_3 (18 mg, 0.07 mmol) in toluene (2 mL) was stirred at 100 ℃ for 18 h. The crude product was purified by column chromatography (petroleum ether/ethyl acetate = 30/1) to give **4e** (116 mg, 63% yield) as a pale-yellow solid, mp 128-129 ℃. HRMS (ESI): calcd. for $C_{27}H_{35}F_3N_2O_5$ $[M+H]^+$: 525.2498, found:525.2566; ^{1}H-NMR (400 MHz, $CDCl_3$): δ = 6.42 (s, 1H), 6.17 (s, 1H), 5.05–4.97 (m, 2H), 4.24 (d, *J* = 8.5 Hz, 1H), 3.93–3.84 (m, 3H), 3.84–3.76 (m, 2H), 3.42–3.32 (m, 2H), 3.13 (dd, *J* = 11.7, 4.7 Hz, 1H), 3.03 (dd, *J* = 7.7, 2.2 Hz, 1H), 2.92 (dd, *J* = 11.7, 3.9 Hz, 1H), 2.85–2.79 (m, 2H), 2.78 (s, 3H), 2.69 (d, *J* = 4.6 Hz, 1H), 1.56 (d, *J* = 10.6 Hz, 1H), 1.24 (ddd, *J* = 15.4, 9.7, 3.9 Hz, 13H); ^{13}C-NMR (100 MHz, $CDCl_3$): δ = 170.38, 170.15, 154.55, 147.94, 130.60 ($^2J_{CF}$ = 31.3), 129.98 ($^2J_{CF}$ = 31.3), 123.28, 102.66 ($^3J_{CF}$ = 4.0), 95.59 ($^3J_{CF}$ = 3.6), 66.94, 66.83, 66.55, 66.38, 49.46, 44.98, 44.14, 43.90, 42.95, 40.90, 33.26, 32.00, 21.08, 20.95, 20.82, 20.73.

2,3-Diisopropyl 7-methyl (1*S*,2*S*,3*R*,4*S*,4a*S*,9a*S*)-9-methyl-5-morpholino-2,3,4,4a,9,9a-hexahydro-1*H*-1,4-methanocarbazole-2,3,7-tricarboxylate (4f)

Synthesized according to General Method A using methyl 4-iodo-3-(methylamino)benzoate (70 mg, 0.24 mmol), diisopropyl bicyclo[2.2.1]hept-5-ene-2,3-dicarboxylate (34 mg, 0.36 mmol), morpholino benzoate (99 mg, 0.48 mmol), Cs_2CO_3 (313 mg, 0.96 mmol), $Pd(OAc)_2$ (5 mg, 0.02 mmol), and PPh_3 (13 mg, 0.05 mmol) in toluene (2 mL) was stirred at 100 ℃ for 12 h. The crude product was purified by column chromatography (petroleum ether/ethyl acetate = 10/1) to give **4f** (80 mg, 64% yield) as a pale-yellow solid, mp 133-134 ℃. HRMS (ESI): calcd. for $C_{28}H_{38}N_2O_7$ $[M+H]^+$: 515.2752, found:515.2749; ^{1}H-NMR (400 MHz, $CDCl_3$): δ = 6.92 (s, 1H), 6.63 (s, 1H), 5.05–4.94 (m, 2H), 4.24 (d, *J* = 8.5 Hz, 1H), 3.93–3.83 (m, 6H), 3.83–3.74 (m, 2H), 3.42–3.32 (m, 2H), 3.11 (dd, *J* = 11.7, 4.7 Hz, 1H), 3.05 (d, *J* = 2.8 Hz, 1H), 2.91 (dd, *J* = 11.7, 3.8 Hz, 1H), 2.84–2.80 (m, 1H), 2.79 (s, 3H), 2.79–2.75 (m, 1H), 2.68 (d, *J* = 4.2 Hz, 1H), 1.54 (d, *J* = 10.6 Hz, 1H), 1.27–1.18 (m, 13H); ^{13}C-NMR (100

MHz, $CDCl_3$): δ = 171.44, 171.15, 167.73, 155.54, 148.82, 130.88, 126.51, 108.70, 100.84, 67.86, 67.77, 67.48, 67.44 (2C), 51.87, 50.63 (2C), 45.99, 45.14, 44.94, 44.10, 41.96, 34.29, 33.10, 22.07, 21.93, 21.80, 21.72.

Diisopropyl (1*S*,2*S*,3*R*,4*S*,4a*S*,9a*S*)-8-fluoro-9-methyl-5-morpholino-2,3,4,4a,9,9a-hexahydro-1*H*-1,4-methanocarbazole-2,3-dicarboxylate (4g)

Synthesized according to General Method A using methyl 2-fluoro-6-iodo-*N*-methylaniline (88 mg, 0.35 mmol), diisopropyl bicyclo[2.2.1]hept-5-ene-2,3-dicarboxylate (48 mg, 0.52 mmol), morpholino benzoate (143 mg, 0.70 mmol), Cs_2CO_3 (451 mg, 1.40 mmol), $Pd(OAc)_2$ (8 mg, 0.04 mmol), and PPh_3 (18 mg, 0.07 mmol) in toluene (2 mL) was stirred at 100 ℃ for 12 h. The crude product was purified by column chromatography (petroleum ether/ethyl acetate = 30/1) to give **4g** (106 mg, 64% yield) as a white solid, mp 132-133 ℃. HRMS (ESI): calcd. for $C_{26}H_{35}FN_2O_5$ $[M+H]^+$: 475.2603, found: 475.2594; ^{1}H-NMR (400 MHz, $CDCl_3$): δ = 6.72 (dd, J_{HF} = 11.7, 8.7 Hz, 1H), 6.25 (s, 1H), 5.07–4.94 (m, 2H), 4.28 (d, *J* = 8.5 Hz, 1H), 3.97–3.85 (m, 2H), 3.84–3.74 (m, 2H), 3.69 (d, *J* = 8.5 Hz, 1H), 3.27 (s, 2H), 3.11 (dd, *J* = 11.7, 4.7 Hz, 1H), 3.02 (s, 1H), 2.97–2.87 (m, 4H), 2.75 (s, 2H), 2.66 (d, *J* = 4.0 Hz, 1H), 1.61 (d, *J* = 10.5 Hz, 1H), 1.28–1.18 (m, 13H); ^{13}C-NMR (100 MHz, $CDCl_3$): δ = 171.47, 171.28, 153.64 ($^1J_{CF}$ = 149.9), 147.14 ($^3J_{CF}$ = 10.2), 146.98, ($^3J_{CF}$ = 10.1), 126.64, 115.56 ($^2J_{CF}$ = 19.0), 108.47 ($^2J_{CF}$ = 26.4), 69.91, 67.87 (2C), 67.47, 67.41, 51.50 (2C), 46.21, 45.65, 45.16, 45.03, 42.64, 37.36, 34.53, 22.11, 21.95, 21.82, 21.75.

Diisopropyl (1*S*,2*S*,3*R*,4*S*,4a*S*,9a*S*)-8-chloro-9-methyl-5-morpholino-2,3,4,4a,9,9a-hexahydro-1*H*-1,4-methanocarbazole-2,3-dicarboxylate (4h)

Synthesized according to General Method A using methyl 2-chloro-6-iodo-*N*-methylaniline (80 mg, 0.30 mmol), diisopropyl bicyclo[2.2.1]hept-5-ene-2,3-dicarboxylate (42 mg, 0.45 mmol), morpholino benzoate (124 mg, 0.60 mmol), Cs_2CO_3 (391 mg, 1.20 mmol), $Pd(OAc)_2$ (7 mg, 0.03 mmol), and PPh_3 (16 mg, 0.06 mmol) in toluene (2 mL) was stirred at 100 ℃ for 12 h. The crude product was purified by column chromatography (petroleum ether/ethyl acetate = 30/1) to give **4h** (103 mg, 70% yield) as a white solid, mp 131-133 ℃. HRMS (ESI): calcd. for $C_{26}H_{35}ClN_2O_5$ $[M+H]^+$: 491.2307, found: 491.2287; ^{1}H-NMR (400 MHz, $CDCl_3$): δ = 6.94 (d, *J* = 8.6 Hz, 1H), 6.29 (d, *J* = 8.6 Hz, 1H), 5.06–4.95 (m, 2H), 4.28 (d, *J* = 8.5 Hz, 1H), 3.93–3.85 (m, 2H), 3.82–3.74 (m, 2H), 3.69 (d, *J* = 8.5 Hz, 1H), 3.32–3.23 (m, 2H), 3.13–3.06 (m, 4H), 3.00 (s, 1H), 2.92 (dd, *J* = 11.8, 3.8 Hz, 1H), 2.73 (dd, *J* = 9.1, 6.7 Hz, 2H), 2.67 (s, 1H), 1.55 (d, *J* = 10.6 Hz, 1H), 1.28–1.19 (m, 13H); ^{13}C-NMR (100 MHz, $CDCl_3$): δ = 171.50, 171.28, 150.97, 147.96, 130.34, 126.52, 110.32, 110.06, 70.41, 67.87, 67.48, 67.41 (2C), 51.09 (2C), 46.17, 46.11, 45.28, 44.01, 42.44, 38.65, 34.44, 22.11, 21.96, 21.82, 21.74.

Diisopropyl (1*S*,2*S*,3*R*,4*S*,4a*S*,9a*S*)-6-fluoro-9-methyl-5-morpholino-2,3,4,4a,9,9a-hexahydro-1*H*-1,4-methanocarbazole-2,3-dicarboxylate (4i)

Synthesized according to General Method A using 4-fluoro-2-iodo-*N*-methylaniline (90 mg, 0.36 mmol), diisopropyl bicyclo[2.2.1]hept-5-ene-2,3-dicarboxylate (50 mg, 0.54 mmol), morpholino benzoate (150 mg, 0.72 mmol), Cs_2CO_3 (469 mg, 1.92 mmol), $Pd(OAc)_2$ (8 mg, 0.036 mmol), and PPh_3 (19 mg, 0.07 mmol) in toluene (2 mL) was stirred at 100 ℃ for 12 h. The crude product was purified by column chromatography (petroleum ether/ethyl acetate = 40/1) to give **4i** (82 mg, 48% yield) as a pale-yellow solid, mp 135-136 ℃. HRMS (ESI): calcd. for $C_{26}H_{35}FN_2O_5$ $[M+H]^+$: 475.2603, found: 475.2589; ^{1}H-NMR (400 MHz, $CDCl_3$): δ = 6.70 (dd, J_{HF} =

12.5, 8.5 Hz, 1H), 5.96 (s, 1H), 5.01 (dtd, J = 12.5, 6.3, 2.0 Hz, 2H), 4.04 (d, J = 8.3 Hz, 1H), 3.97 (d, J = 8.3 Hz, 1H), 3.88–3.81 (m, 2H), 3.80–3.71 (m, 2H), 3.19 (ddd, J = 13.2, 9.8, 2.4 Hz, 2H), 3.05–3.01 (m, 2H), 3.01–2.94 (m, 2H), 2.82 (s, 1H), 2.76 (d, J = 10.0 Hz, 3H), 2.64 (s, 1H), 1.63 (d, J = 10.5 Hz, 1H), 1.30–1.22 (m, 13H); ^{13}C-NMR (100 MHz, $CDCl_3$): δ =171.35, 171.27, 151.34 ($^1J_{CF}$ = 151.2), 135.66 ($^3J_{CF}$ = 12.4), 135.54, 128.54, 115.21 ($^2J_{CF}$ = 21.2), 101.39, 68.14, 67.76 (2C), 67.66 (2C), 51.32, 51.28, 46.38, 45.21, 45.16, 45.08, 44.17, 34.59, 34.21, 22.14, 21.96, 21.86, 21.75.

Diisopropyl (1*S*,2*S*,3*R*,4*S*,4a*S*,9a*S*)-9-(tert-butyl)-5-morpholino-2,3,4,4a,9,9a-hexahydro-1*H*-1,4-methanocarbazole-2,3-dicarboxylate (5a)

Synthesized according to general method B using *N*-(tert-butyl)-2-iodoaniline (85 mg, 0.31 mmol), diisopropyl bicyclo[2.2.1]hept-5-ene-2,3-dicarboxylate (124 mg, 0.47 mmol), morpholino benzoate (128 mg, 0.62 mmol), PPh_3 (16 mg, 0.062 mmol), $Pd(OAc)_2$ (7 mg,0.031 mmol), Cs_2CO_3 (404 mg, 1.24 mmol). The product was purified by flash column chromatography (petroleum ether/ethyl acetate = 10/1) to provide **5a** as a yellow oil (69 mg, 45% yield). HRMS (ESI): calcd. for $C_{29}H_{42}N_2O_5$ $[M+H]^+$: 499.3094, found: 499.3155; ^{1}H NMR (400 MHz, $CDCl_3$) δ = 6.92 (t, J = 8.0 Hz, 1H), 6.51 (d, J = 8.1 Hz, 1H), 6.27 (d, J = 7.9 Hz, 1H), 5.09-4.94 (m, 2H), 4.20 (d, J = 8.5 Hz, 1H), 4.12 (d, J = 8.6 Hz, 1H), 3.93-3.84 (m, 2H), 3.82-3.74 (m, 2H), 3.27 (m, 2H), 3.13 (s, 1H), 3.05-2.94 (m, 2H), 2.76 (m, 2H), 2.58 (d, J = 2.2 Hz, 1H), 1.57 (d, J = 10.5 Hz, 1H), 1.38 (s, 9H), 1.28-1.22 (m, 12H), 1.15 (d, J = 10.4 Hz, 1H). ^{13}C NMR (100 MHz, $CDCl_3$) δ = 171.90, 171.68, 154.23, 149.15, 127.90, 124.72, 107.31, 107.26, 67.95, 67.65 (2C), 67.57, 62.93, 55.20, 51.11, 49.40, 46.47, 46.23, 44.17, 42.46, 34.48, 29.21 (3C), 27.00, 22.23, 22.07, 21.99, 21.93.

Diisopropyl (1*S*,2*S*,3*R*,4*S*,4a*S*,9a*S*)-9-benzyl-5-morpholino-2,3,4,4a,9,9a-hexahydro-1*H*-1,4-methanocarbazole-2,3-dicarboxylate (5b)

Synthesized according to general method B using *N*-benzyl-2-iodoaniline (100 mg, 0.32 mmol), diisopropyl bicyclo[2.2.1]hept-5-ene-2,3-dicarboxylate (128 mg, 0.48 mmol), morpholino benzoate (133 mg, 0.64 mmol), PPh_3 (17 mg, 0.06 mmol), $Pd(OAc)_2$ (7 mg, 0.03 mmol), Cs_2CO_3 (417 mg, 1.28 mmol). The crude product was purified by flash column chromatography (petroleum ether/ethyl acetate = 10/1) to provide **5b** as a yellow oil (145 mg, 85% yield). HRMS (ESI): calcd. for $C_{32}H_{40}N_2O_5$ $[M+H]^+$: 533.2937, found: 533.2995; ^{1}H NMR (400 MHz, $CDCl_3$) δ = 7.30-7.21 (m, 5H), 6.96 (t, J = 7.9 Hz, 1H), 6.26 (d, J = 8.0 Hz, 1H), 6.06 (d, J = 7.8 Hz, 1H), 4.99 (m, 1H), 4.90 (m, 1H), 4.34 (d, J = 2.5 Hz, 2H), 4.31 (d, J = 8.4 Hz, 1H), 3.95 (d, J = 8.4 Hz, 1H), 3.93-3.87 (m, 2H), 3.86-3.79 (m, 2H), 3.45-3.34 (m, 2H), 3.10 (d, J = 2.6 Hz, 1H), 3.05 (dd, J = 11.7, 4.8 Hz, 1H), 2.89 (dd, J = 11.8, 3.8 Hz, 1H), 2.85-2.77 (m, 2H), 2.52 (d, J = 4.4 Hz, 1H), 1.73 (d, J = 10.5 Hz, 1H), 1.25-1.22 (m, 4H), 1.19 (dd, J = 6.3, 1.4 Hz, 6H), 1.11 (d, J = 6.2 Hz, 3H). ^{13}C NMR (100 MHz, $CDCl_3$) δ = 171.67, 171.35, 155.25, 149.55, 139.48, 128.96, 128.60 (2C), 127.25 (2C), 127.03, 121.14, 106.93, 101.11, 67.97, 67.73 (2C), 67.49, 66.92, 51.83, 50.93 (2C), 46.32, 45.72, 45.34, 44.30, 42.35, 34.53, 22.25, 21.95, 21.92, 21.86.

Diisopropyl (1*S*,2*S*,3*R*,4*S*,4a*S*,9a*S*)-9-(3-fluorobenzyl)-5-morpholino-2,3,4,4a,9,9a-hexahydro-1*H*-1,4-methanocarbazole-2,3-dicarboxylate (5c)

Synthesized according to general method B using *N*-(3-fluorobenzyl)-2-iodoaniline (100 mg, 0.31 mmol), diisopropyl bicyclo[2.2.1]hept-5-ene-2,3-dicarboxylate (124 mg, 0.47 mmol), morpholino benzoate (128 mg, 0.62

mmol), PPh_3 (16 mg, 0.06 mmol), $Pd(OAc)_2$ (7 mg,0.03 mmol), Cs_2CO_3 (404 mg, 1.24 mmol). The crude product was purified by flash column chromatography (petroleum ether/ethyl acetate = 10/1) to provide **5c** as a yellow oil (97 mg, 57% yield). HRMS (ESI): calcd. for $C_{32}H_{39}FN_2O_5$ $[M+H]^+$: 551.2843, found: 551.2899; ^{1}H NMR (400 MHz, $CDCl_3$) δ = 7.29-7.20 (m, 1H), 7.04 (d, *J* = 7.6 Hz, 1H), 6.93 (m, 3H), 6.27 (d, *J* = 8.0 Hz, 1H), 6.02 (d, *J* = 7.8 Hz, 1H), 5.09-4.97 (m, 1H), 4.95-4.87 (m, 1H), 4.33 (m, 2H), 4.31 (d, 1H), 3.96 (d, *J* = 8.6 Hz, 1H), 3.94-3.86 (m, 2H), 3.85-3.76 (m, 2H), 3.44-3.33 (m, 2H), 3.11 (d, *J* = 2.7 Hz, 1H), 3.06 (dd, *J* = 11.8, 4.7 Hz, 1H), 2.92 (dd, *J* = 11.8, 3.8 Hz, 1H), 2.82 (m, 2H), 2.52 (d, *J* = 4.5 Hz, 1H), 1.72 (d, *J* = 10.5 Hz, 1H), 1.25 (m, 4H), 1.20 (d, *J* = 6.3 Hz, 6H), 1.13 (d, *J* = 6.2 Hz, 3H). ^{13}C NMR (100 MHz, $CDCl_3$) δ = 171.62, 171.31, 163.16($^1J_{CF}$=246.2 Hz), 154.97, 149.63, 142.45 ($^3J_{CF}$ = 6.5 Hz), 130.10 ($^3J_{CF}$ = 8.1 Hz), 128.99, 122.68 ($^4J_{CF}$ = 2.8 Hz), 121.12, 114.05 ($^2J_{CF}$ = 21.4 Hz), 113.93 ($^2J_{CF}$ = 21.2 Hz), 107.20, 101.04, 67.97, 67.70 (2C), 67.53, 66.99, 51.59 ($^4J_{CF}$ = 1.6 Hz), 50.88 (2C), 46.26, 45.73, 45.31, 44.38, 42.28, 34.50, 22.22, 21.92, 21.8, 21.85.

Diisopropyl (1*S*,2*S*,3*R*,4*S*,4a*S*,9a*S*)-9-(4-fluorobenzyl)-5-morpholino-2,3,4,4a,9,9a-hexahydro-1*H*-1,4-methanocarbazole-2,3-dicarboxylate (5d)

Synthesized according to general method B using *N*-(4-fluorobenzyl)-2-iodoaniline (100 mg, 0.31 mmol), diisopropyl bicyclo[2.2.1]hept-5-ene-2,3-dicarboxylate (124 mg, 0.47 mmol), morpholino benzoate (128 mg, 0.62 mmol), PPh_3 (16 mg, 0.06 mmol), $Pd(OAc)_2$ (7 mg, 0.03 mmol), Cs_2CO_3 (404 mg, 1.24 mmol). The crude product was purified by flash column chromatography (petroleum ether/ethyl acetate = 10/1) to provide **5d** as a white solid (130 mg, 76% yield), mp 140-141 ℃. HRMS (ESI): calcd. for $C_{32}H_{39}FN_2O_5$ $[M+H]^+$: 551.2843, found: 551.2905; ^{1}H NMR (400 MHz, $CDCl_3$) δ = 7.21 (m, 2H), 6.96 (m, 3H), 6.27 (d, *J* = 7.6 Hz, 1H), 6.05 (d, *J* = 7.7 Hz, 1H), 5.05-4.95 (m, 1H), 4.94-4.84 (m, 1H), 4.31 (d, *J* = 2.1 Hz, 2H), 4.28 (d, *J* = 8.7 Hz, 1H), 3.98-3.87 (m, 3H), 3.86-3.74 (m, 2H), 3.44-3.30 (m, 2H), 3.10 (d, *J* = 2.6 Hz, 1H), 3.04 (dd, *J* = 11.8, 4.7 Hz, 1H), 2.90 (dd, *J* = 11.8, 3.8 Hz, 1H), 2.86-2.76 (m, 2H), 2.49 (d, *J* = 4.1 Hz, 1H), 1.69 (d, *J* = 10.5 Hz, 1H), 1.24 (m, 4H), 1.19 (d, *J* = 6.3 Hz, 6H), 1.12 (d, *J* = 6.2 Hz, 3H). ^{13}C NMR (100 MHz, $CDCl_3$) δ = 171.65, 171.35, 162.06 ($^1J_{CF}$ = 244.8 Hz), 155.06, 149.59, 135.07, 129.01, 128.82 ($^3J_{CF}$ = 8.0 Hz, 2C), 121.20, 115.42 ($^2J_{CF}$ = 21.4 Hz, 2C), 107.17, 101.11, 67.99, 67.70 (2C), 67.58, 66.74, 51.17, 50.93 (2C), 46.30, 45.70, 45.34, 44.38, 42.36, 34.52, 22.25, 21.97, 21.91, 21.88.

Diisopropyl (1*S*,2*S*,3*R*,4*S*,4a*S*,9a*S*)-9-(3-methoxybenzyl)-5-morpholino-2,3,4,4a,9,9a-hexahydro-1*H*-1,4-methanocarbazole-2,3-dicarboxylate (5e)

Synthesized according to general method B using 2-iodo-*N*-(3-methoxy benzyl) aniline (100 mg, 0.25 mmol), diisopropyl bicyclo[2.2.1]hept-5-ene-2,3-dicarboxylate (124 mg, 0.47 mmol), morpholino benzoate (128 mg, 0.62 mmol), PPh_3 (16 mg, 0.06 mmol), $Pd(OAc)_2$ (7 mg,0.03 mmol), Cs_2CO_3 (404 mg, 1.24 mmol). The crude product was purified by flash column chromatography (petroleum ether/ethyl acetate = 10/1) to provide **5e** as a yellow oil (119 mg, 85% yield). HRMS (ESI): calcd. for $C_{33}H_{42}N_2O_6$ $[M+H]^+$: 563.3043, found: 563.3096; ^{1}H NMR (400 MHz, $CDCl_3$) δ = 7.19 (t, *J* = 7.8 Hz, 1H), 6.95 (t, *J* = 7.9 Hz, 1H), 6.84 (d, *J* = 7.6 Hz, 1H), 6.82 (s, 1H), 6.76 (dd, *J* = 8.1, 2.3 Hz, 1H), 6.29-6.22 (m, 1H), 6.04 (d, *J* = 7.8 Hz, 1H), 5.05-4.96 (m, 1H), 4.94-4.85 (m, 1H), 4.39-4.22 (m, 3H), 3.99-3.87 (m, 3H), 3.86-3.78 (m, 2H), 3.76 (s, 3H), 3.39 (m, 2H), 3.09 (d, *J* = 2.7 Hz, 1H), 3.06 (dd, *J* = 11.8, 4.7 Hz, 1H), 2.90 (dd, *J* = 11.8, 3.8 Hz, 1H), 2.86-2.75 (m, 2H), 2.54 (d, *J* = 4.3 Hz, 1H), 1.74 (d, *J* = 10.4 Hz, 1H), 1.26-1.21 (m, 4H), 1.19 (d, *J* = 6.3 Hz, 6H), 1.12 (d, *J* = 6.2 Hz, 3H). ^{13}C NMR (100 MHz, $CDCl_3$) δ =

171.66, 171.35, 159.93, 155.27, 141.36, 134.92, 129.60, 128.97, 121.18, 119.49, 112.80, 112.39, 106.98, 101.19, 67.99, 67.85, 67.49, 67.15, 55.25, 52.09, 50.94, 48.55, 46.57, 46.32, 45.79, 45.33, 44.32, 42.36, 34.52, 22.25, 22.95, 21.92, 21.87.

Diisopropyl (1*S*,2*S*,3*R*,4*S*,4a*S*,9a*S*)-5-morpholino-9-(pyridin-3-ylmethyl)-2,3,4,4a,9,9a-hexahydro-1*H*-1,4-methanocarbazole-2,3-dicarboxylate (5f)

Synthesized according to general method B using 2-iodo-*N*-(pyridin-3-ylmethyl) aniline (100 mg, 0.32 mmol), diisopropyl bicyclo[2.2.1]hept-5-ene-2,3-dicarboxylate (124 mg, 0.47 mmol), morpholino benzoate (128 mg, 0.62 mmol), PPh_3 (16 mg, 0.06 mmol), $Pd(OAc)_2$ (7 mg,0.03 mmol), Cs_2CO_3 (404 mg, 1.24 mmol). The crude product was purified by flash column chromatography (petroleum ether/ethyl acetate = 5/1) to provide **5f** as a yellow oil (82 mg, 48% yield). HRMS (ESI): calcd. for $C_{31}H_{39}N_3O_5$ $[M+H]^+$: 534.2890, found: 534.2961; ^{1}H NMR (400 MHz, $CDCl_3$) δ = 8.54 (d, *J* = 1.3 Hz, 1H), 8.49 (d, *J* = 3.6 Hz, 1H), 7.59 (d, *J* = 7.8 Hz, 1H), 7.22 (dd, *J* = 7.8, 4.8 Hz, 1H), 6.96 (t, *J* = 7.9 Hz, 1H), 6.27 (d, *J* = 8.0 Hz, 1H), 6.04 (d, *J* = 7.8 Hz, 1H), 5.04-4.86 (m, 2H), 4.36 (d, *J* = 5.4 Hz, 2H), 4.27 (d, *J* = 8.5 Hz, 1H), 3.97 (d, *J* = 8.5 Hz, 1H), 3.93-3.86 (m, 2H), 3.84-3.75 (m, 2H), 3.41-3.30 (m, 2H), 3.10 (d, *J* = 2.7 Hz, 1H), 3.03 (dd, *J* = 11.8, 4.6 Hz, 1H), 2.92 (dd, *J* = 11.8, 3.8 Hz, 1H), 2.80 (m, 2H), 2.47 (d, *J* = 4.2 Hz, 1H), 1.68 (d, *J* = 10.5 Hz, 1H), 1.23 (m, 4H), 1.19 (d, *J* = 6.3 Hz, 6H), 1.13 (d, *J* = 6.2 Hz, 3H).^{13}C NMR (100 MHz, $CDCl_3$) δ = 171.61, 171.30, 154.77, 149.69, 148.89, 148.49, 135.23, 135.05, 129.05, 123.66, 121.22, 107.52, 101.12, 68.02, 67.68 (2C), 67.62, 66.88, 50.87 (2C), 49.63, 46.25, 45.81, 45.31, 44.49, 42.29, 34.49, 22.22, 21.99, 21.88, 21.86.

Supplementary Method 2: Inhibitory assay of CYP11B1

To V79 MZh cells expressing human CYP11B1 in 96-wells microplate were added testing compounds in desired concentrations. After an incubation of 30 minutes at 37 ^{o}C, it was added 11-deoxycortisol (100 nM) as the substrate, and further incubated for 6 hours. The convertion was stopped and the steroids were extracted with ethyl acetate. After the removal of ethyl acetate, the residues were redisolved with 20 μL methanol, and subjected to HPLC. The substrate 11-deoxycortisol and product cortisol were quantified with a PDA monitor at a 240 nM. Then the inhibitory potency (inhibitory percentage and IC_{50}) was calculated from the reduced substrate conversion.

Supplementary Method 3: DFT Computation

All DFT calculations were carried out using Gaussian16 package. [1] The initial geometries were taken from the crystal structure (compound 2b, CCDC 2121980) and a previous computational study [2] and modified into the current systems. All geometry optimisations and relaxed potential energy scans were performed at B3LYP-D3 level of theory in toluene solvent employing the Polarizable Continuum Model (PCM). Grimme's DFT-D3 dispersion correction was used to describe the van der Waals interactions. Pd, Cs, P and I atoms were described by LanL2DZ basis set. Additional polarization functions were added to Pd ($\zeta_d = 1.472$), P ($\zeta_d = 0.364$), Cs ($\zeta_d = 0.19$) and I ($\zeta_d = 0.294$) while additional diffuse functions were added to P ($\zeta_p = 0.0298$) and I ($\zeta_p = 0.0308$), consistant with a previous computational study for comparison [3]. The C, N, O and H atoms were represented by the 6-31G(d,p) basis set. Frequency calculations were performed to confirm all the local minima and transition state structures. Intrinsic reaction coordinate (IRC) calculations were carried out to confirm the minima connecting to the relevant transition state. The Gibbs free energies in solution were obtained by adding the thermodynamic corrections to the electronic energies. In addition, single point calculations with the PCM implicit solvent model (toluene solvent) were used to recalculate the electronic energies of the reactant, transition state and product to improve the accuracy of the energy profiles, where M06-2X functional was used with Pd, Cs and I atoms represented by the SDD pseudopotential and the C, N, O, H and P atoms by with the 6-311++G(d,p) basis set. The thermodynamic corrections obtained at the B3LYP-3D level of theory were added to derive the free energies at M06-2X level. In the energy profiles, Gibbs free energies (kcal/mol) were presented and used in the discussion.

Supplementary Note 1: Configuration determination of compound **2l** using 2D-NMR

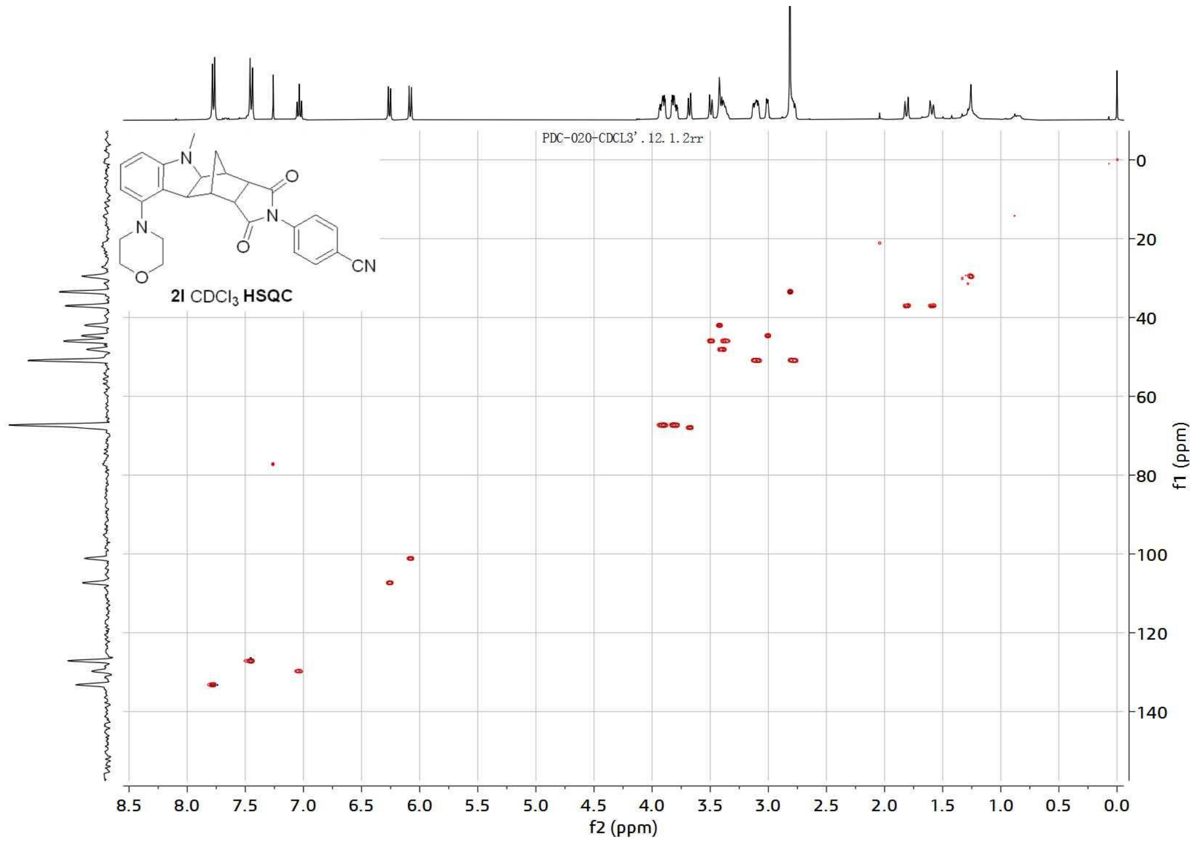

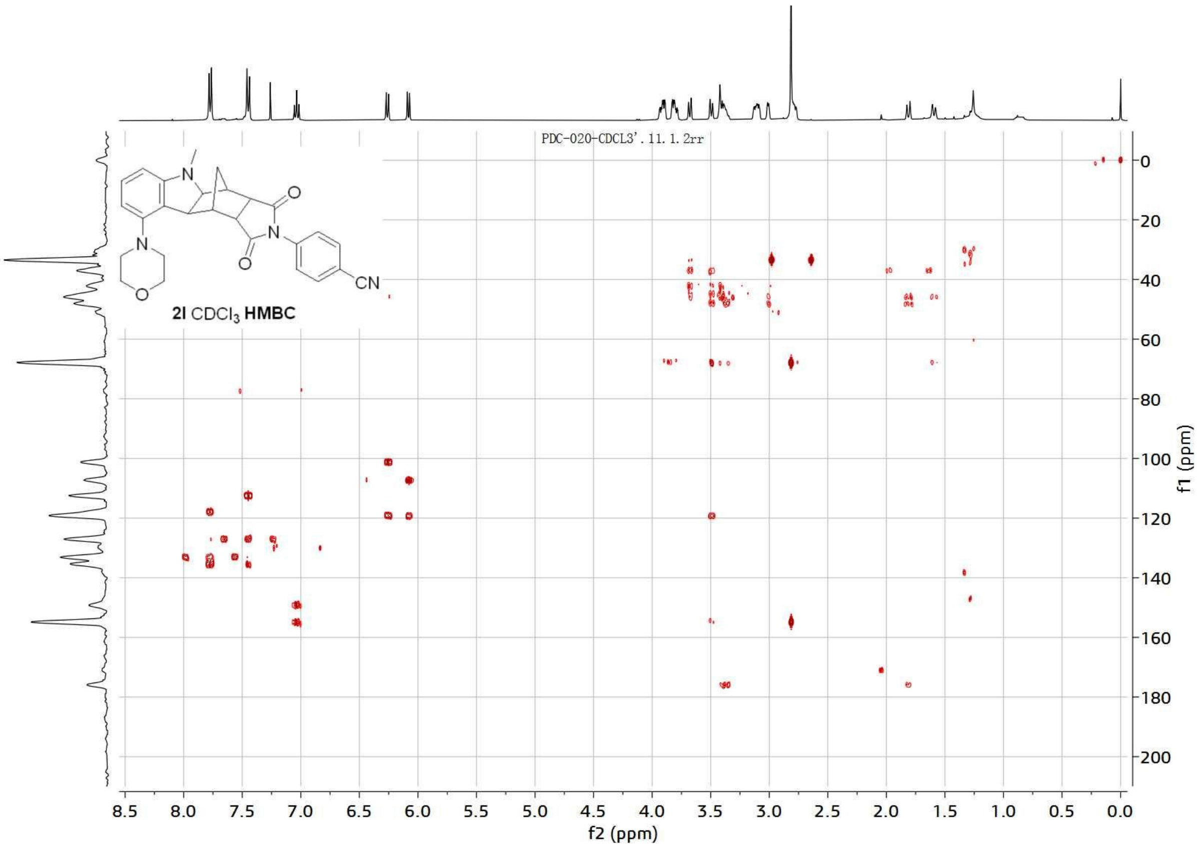

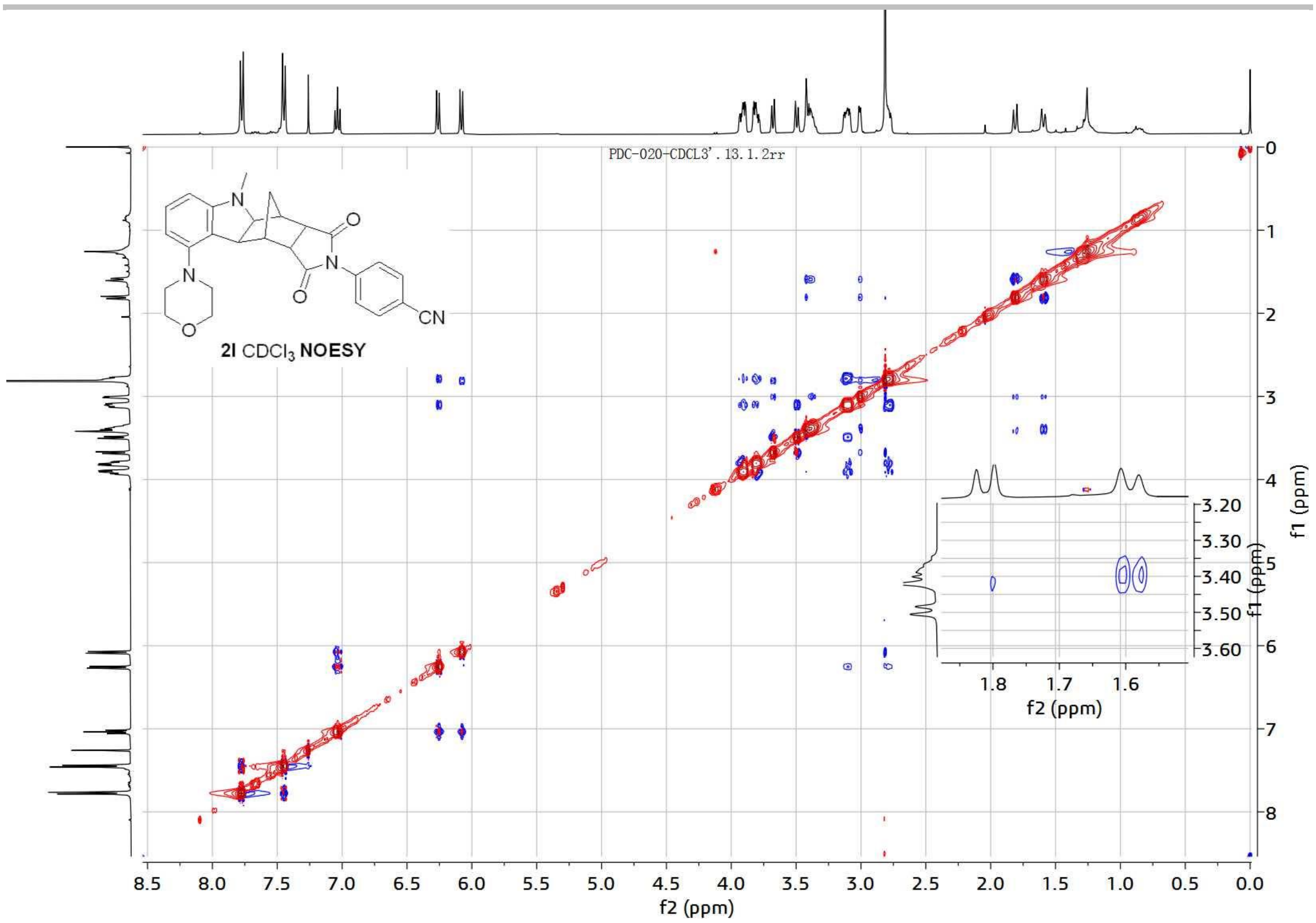

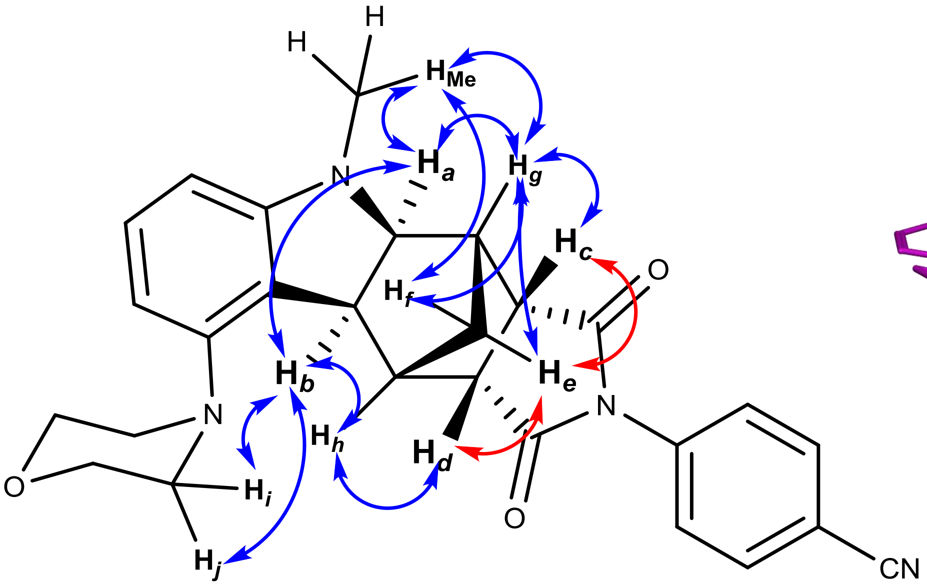

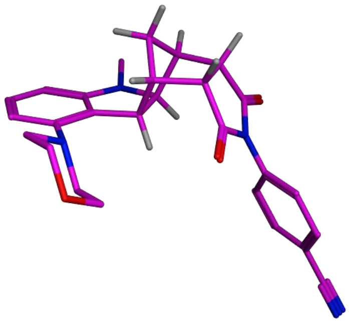

atoms	Chemical Shifts		atoms	Chemical Shifts		atoms	Chemical Shifts	
	^{1}H (ppm)	^{13}C (ppm)		^{1}H (ppm)	^{13}C (ppm)		^{1}H (ppm)	^{13}C (ppm)
**H_a / C_a**	3.68	68.1	**H_d / C_d**	3.48	46.0	**H_c / C_c**	3.39	48.2
**H_d / C_d**	3.37	46.0	**H_e / C_e**	1.59	36.9	**H_f / C_e**	1.81	36.9
**H_g / C_g**	3.01	44.5	**H_h / C_h**	3.42	41.9	**H_i / C_i**	3.11	50.9
**H_j / C_i**	2.79	50.9	**H_{Me} / C_{Me}**	2.81	33.4			

Supplementary References

(1) Gaussian 16, Revision C.01, M. J. Frisch, G. W. Trucks, H. B. Schlegel, G. E. Scuseria, M. A. Robb, J. R. Cheeseman, G. Scalmani, V. Barone, G. A. Petersson, H. Nakatsuji, X. Li, M. Caricato, A. V. Marenich, J. Bloino, B. G. Janesko, R. Gomperts, B. Mennucci, H. P. Hratchian, J. V. Ortiz, A. F. Izmaylov, J. L. Sonnenberg, D. Williams-Young, F. Ding, F. Lipparini, F. Egidi, J. Goings, B. Peng, A. Petrone, T. Henderson, D. Ranasinghe, V. G. Zakrzewski, J. Gao, N. Rega, G. Zheng, W. Liang, M. Hada, M. Ehara, K. Toyota, R. Fukuda, J. Hasegawa, M. Ishida, T. Nakajima, Y. Honda, O. Kitao, H. Nakai, T. Vreven, K. Throssell, J. A. Montgomery, Jr., J. E. Peralta, F. Ogliaro, M. J. Bearpark, J. J. Heyd, E. N. Brothers, K. N. Kudin, V. N. Staroverov, T. A. Keith, R. Kobayashi, J. Normand, K. Raghavachari, A. P. Rendell, J. C. Burant, S. S. Iyengar, J. Tomasi, M. Cossi, J. M. Millam, M. Klene, C. Adamo, R. Cammi, J. W. Ochterski, R. L. Martin, K. Morokuma, O. Farkas, J. B. Foresman, and D. J. Fox, Gaussian, Inc., Wallingford CT, 2016.

(2) Y. J. Liang, Y. Y. Jiang, Y. X. Liu, S. W. Bi, *Org Biomol Chem* **2017**, *15*, 6147-6156

(3) B. S. Zhang, Y. K. Li, Z. Zhang, Y. An, Y. H. Wen, X. Y. Gou, S. Q. Quan, X. G. Wang, Y. M. Liang, *J Am Chem Soc* **2019**, *141*, 9731-9738.
