# Supplementary material for: Manipulations of phenylnorbornyl palladium species for multicomponent construction of a bridged polycyclic privileged scaffold
Source: Commun Chem. 2022 Oct 29;5:140. doi: 10.1038/s42004-022-00759-4 (PMC9814782; doi:10.1038/s42004-022-00759-4)
Supplement: Supplementary file 2 — Description of Additional Supplementary Files [file 42004_2022_759_MOESM2_ESM.docx]

Description of Additional Supplementary Files

**File name:** Supplementary Data 1

**Description**: crystal data of compound **2b**

**File name:** Supplementary Data 2

**Description**: The ^1^H-, ^13^C- and ^19^F-NMR as well as HRMS spectra of final compounds

**File name:** Supplementary Data 3

**Description**: Cartesian coordinates of all the structures reported and their absolute energies in Hartree.
