# Supplementary material for: Manipulations of phenylnorbornyl palladium species for multicomponent construction of a bridged polycyclic privileged scaffold
Source: Commun Chem. 2022 Oct 29;5:140. doi: 10.1038/s42004-022-00759-4 (PMC9814782; doi:10.1038/s42004-022-00759-4)

Manipulations of Phenylnorbornyl Palladium Species for Multicomponent Construction of a Bridged Polycyclic Privileged Scaffold

Lina Yin,*^[a]^ Ting Guan,^‡[a]^ Jie Cheng,^‡[a]^ Dongchao Pan,^‡[a]^ Jinyang Lu,^‡[a]^ Jiahui Huang,^[a]^ Jiaqi Wu,^[a]^ Xiaoli Chen,^[a]^ Taiyun You,^[a]^ Xuting Huo,^[a]^ Yuting He,^[a]^ Jiayun Pang,*^[b]^ and Qingzhong Hu*^[a]^

[a] T. Guan, J. Cheng, D. Pan, J. Lu, M. Liu, J. Huang, J. Wu, X. Chen, T. You, X. Huo, Y. He, Prof. Dr. L. Yin, and Prof. Dr. Q. Hu
School of Pharmaceutical Sciences, Guangzhou University of Chinese Medicine
232 East Waihuan Road, Panyu, Guangzhou, China PR
E-mail: huqqzh@gzucm.edu.cn (Q. Hu); [linayin@gzucm.edu.cn](mailto:linayin@gzucm.edu.cn) (L. Yin)

[b] Dr. J Pang
School of Science, Faculty of Engineering and Science, University of Greenwich
Medway Campus, Central Avenue, Chatham Maritime, ME4 3RL, United Kingdom
Email: [j.pang@gre.ac.uk](mailto:j.pang@gre.ac.uk) (J. Pang)

^‡^ These authors contribute equally to this work.

**The ^1^H-, ^13^C- and ^19^F-NMR as well as HRMS spectra of final compounds:**


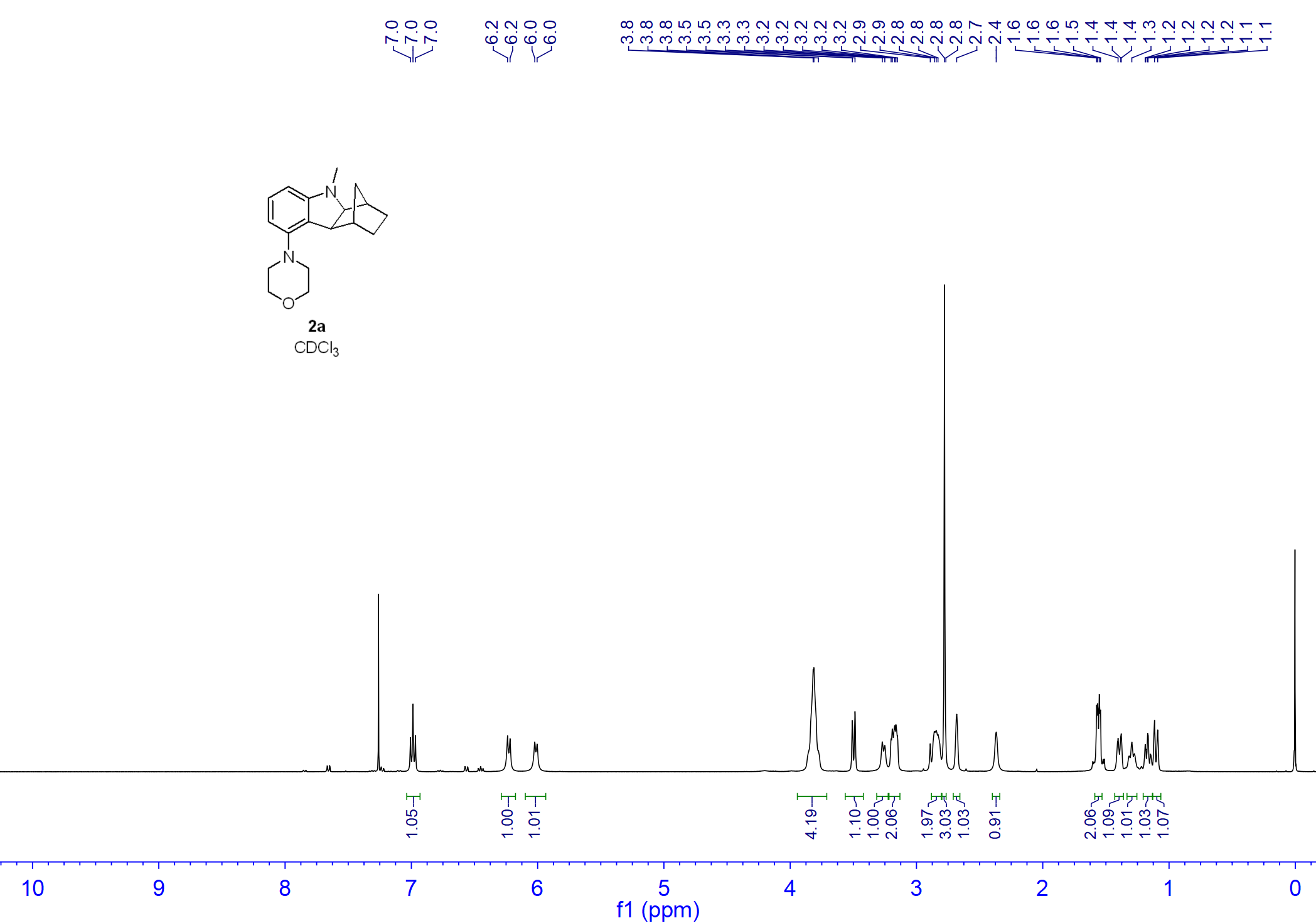


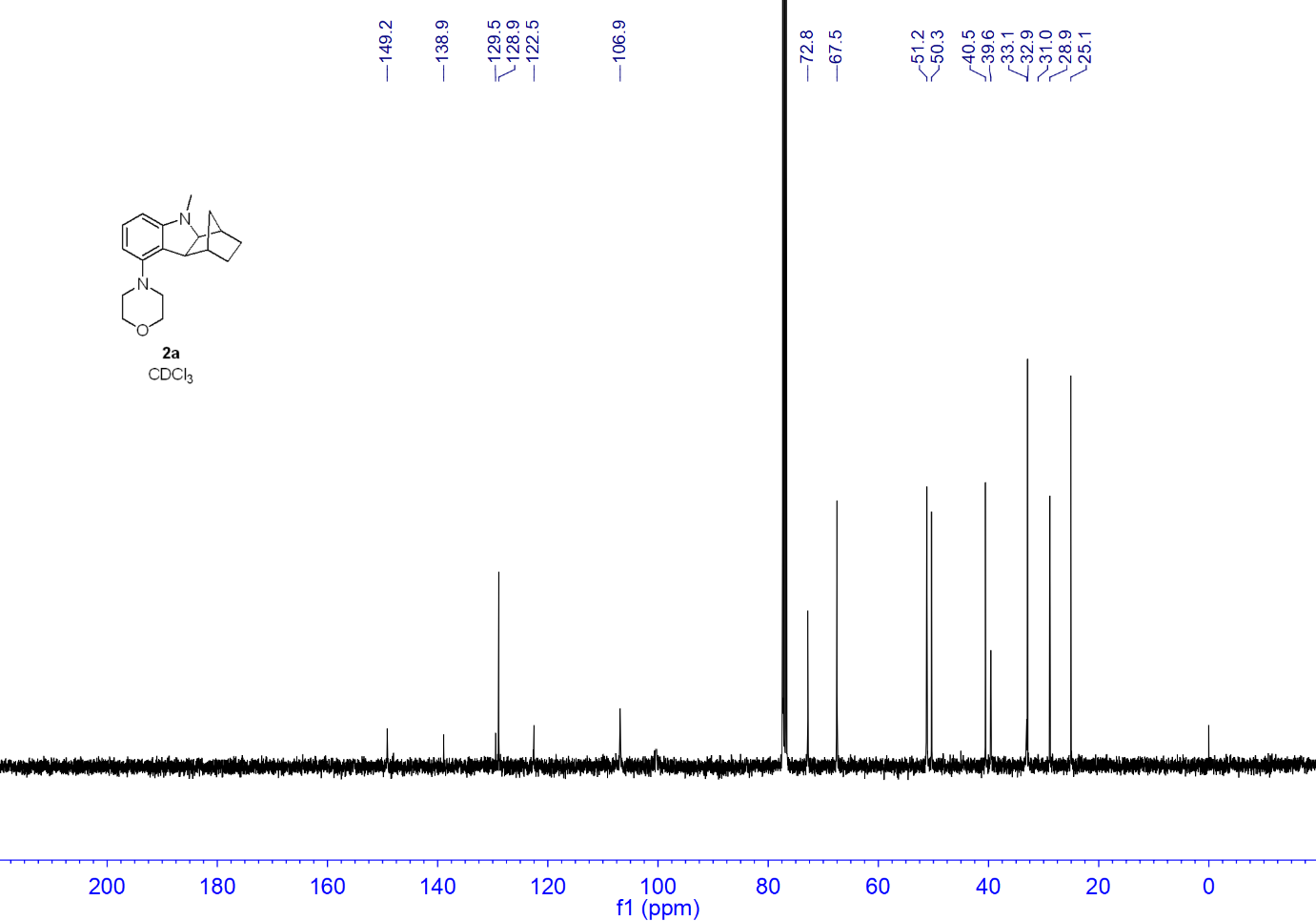

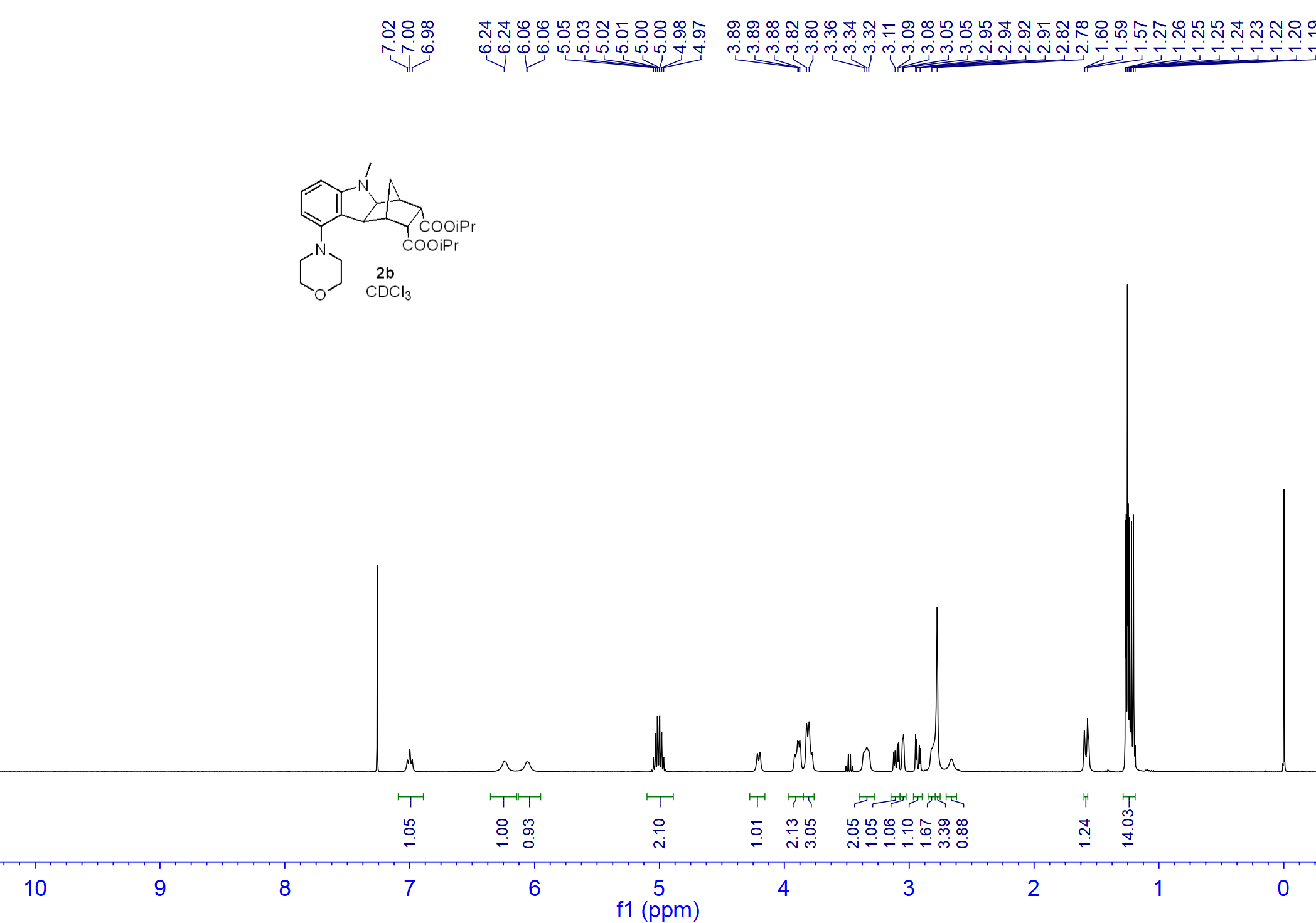


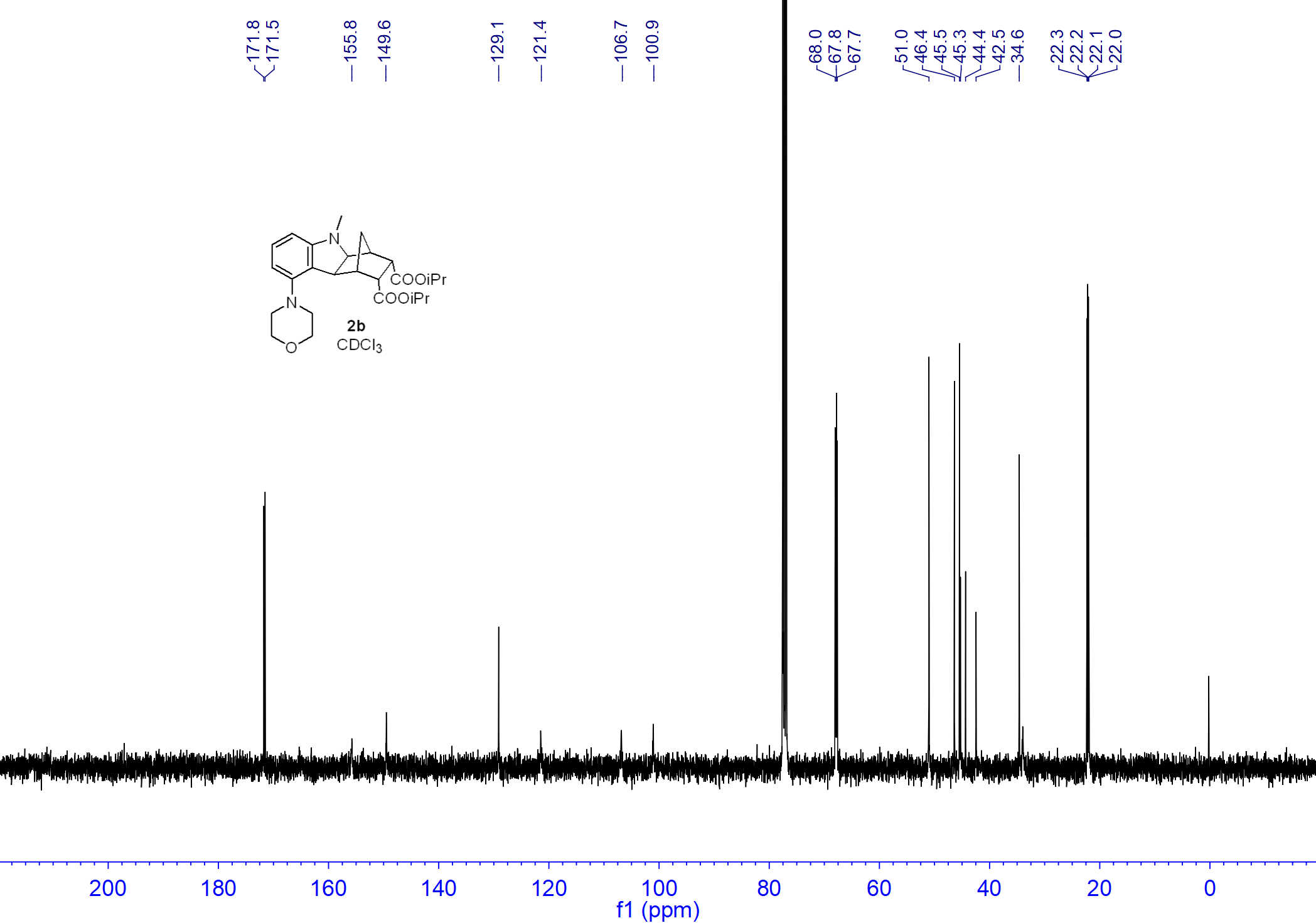

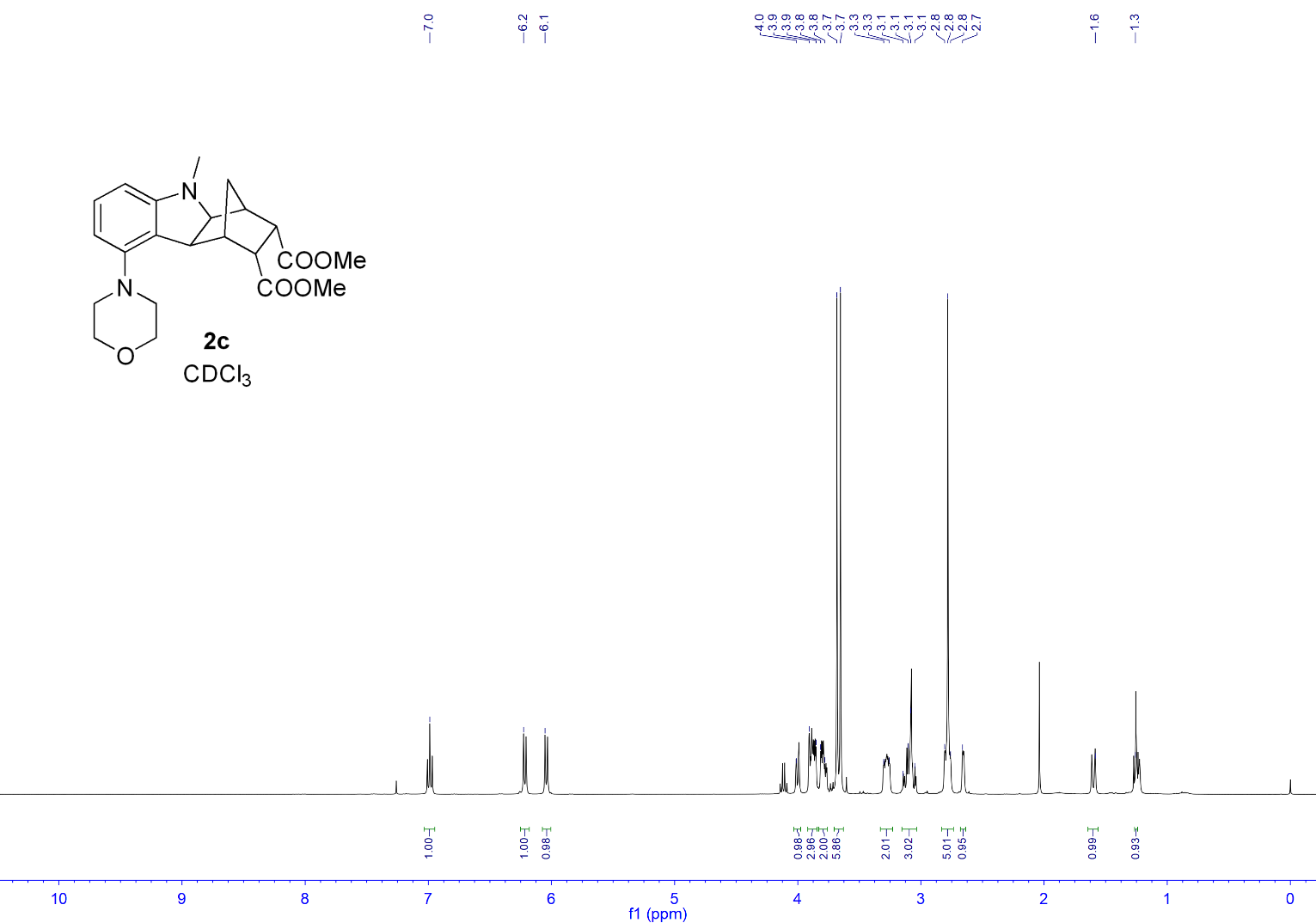


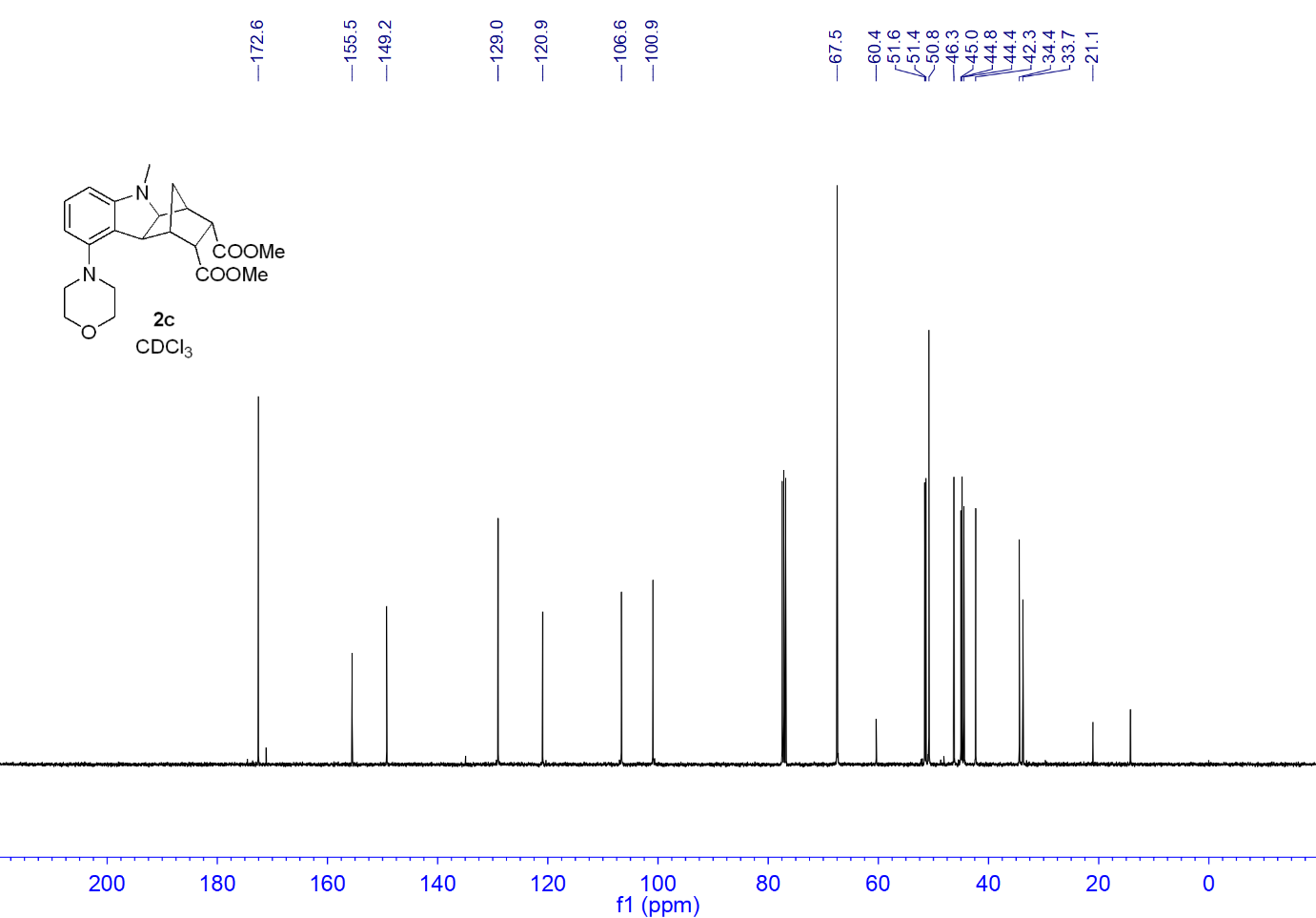

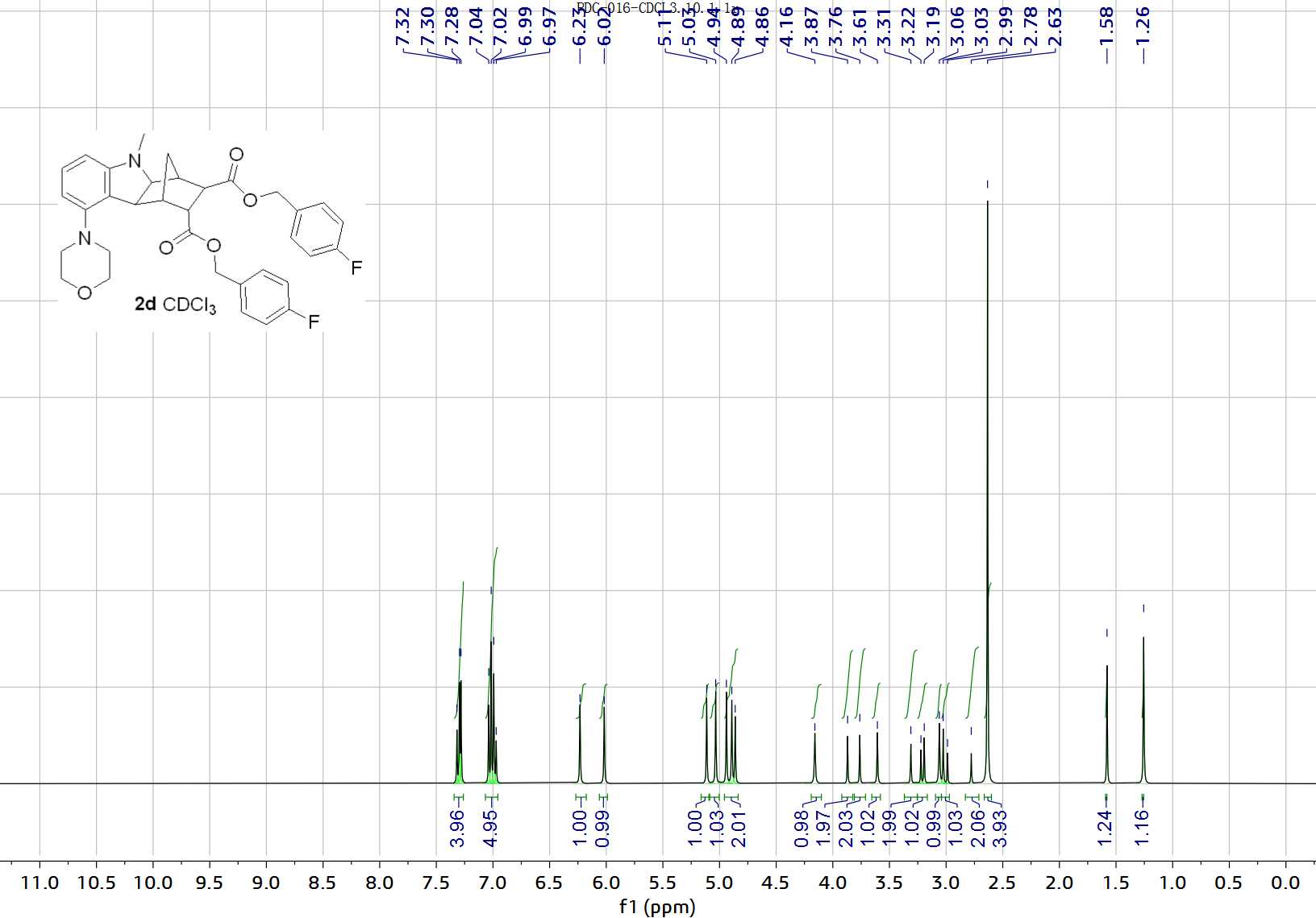


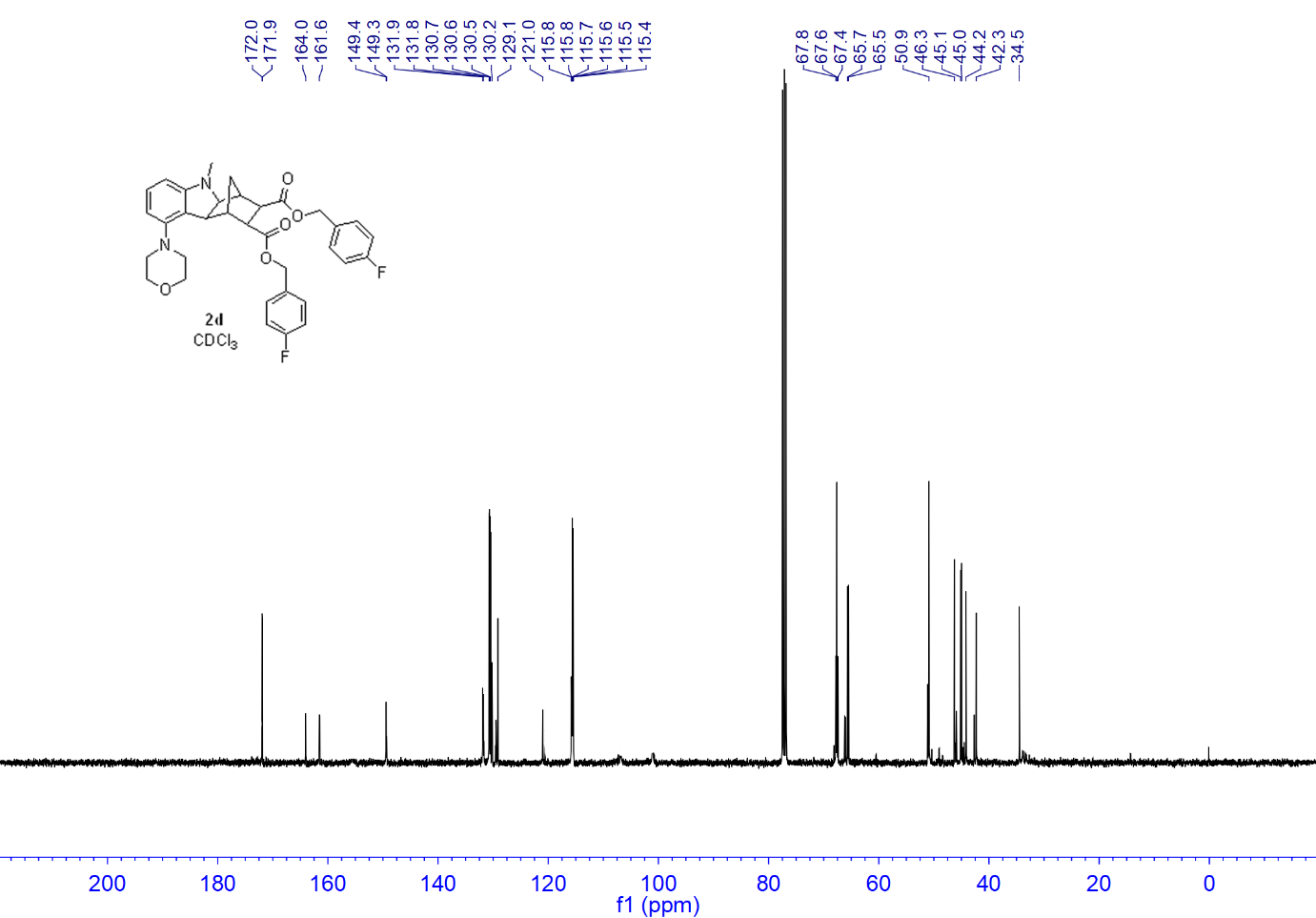


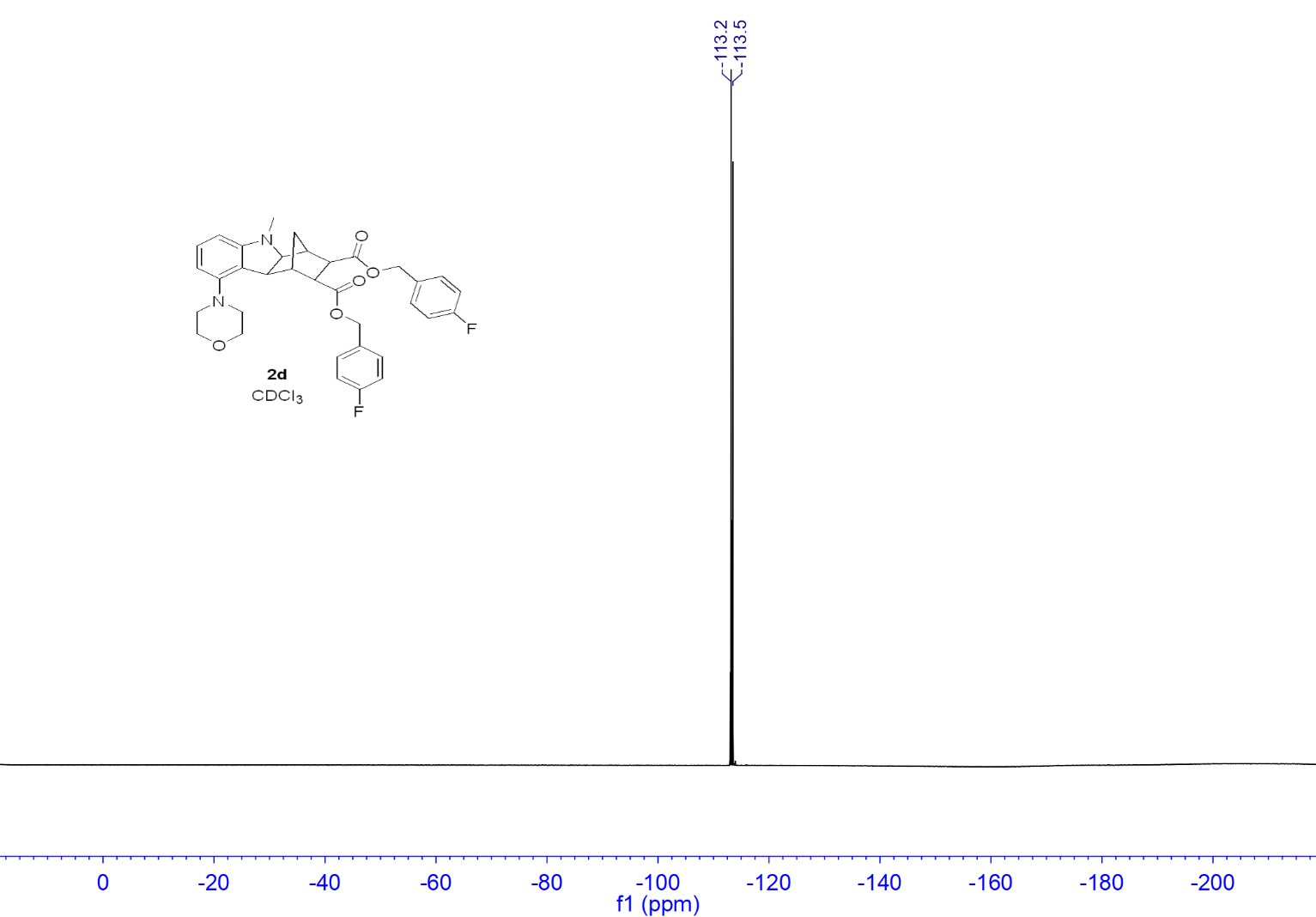

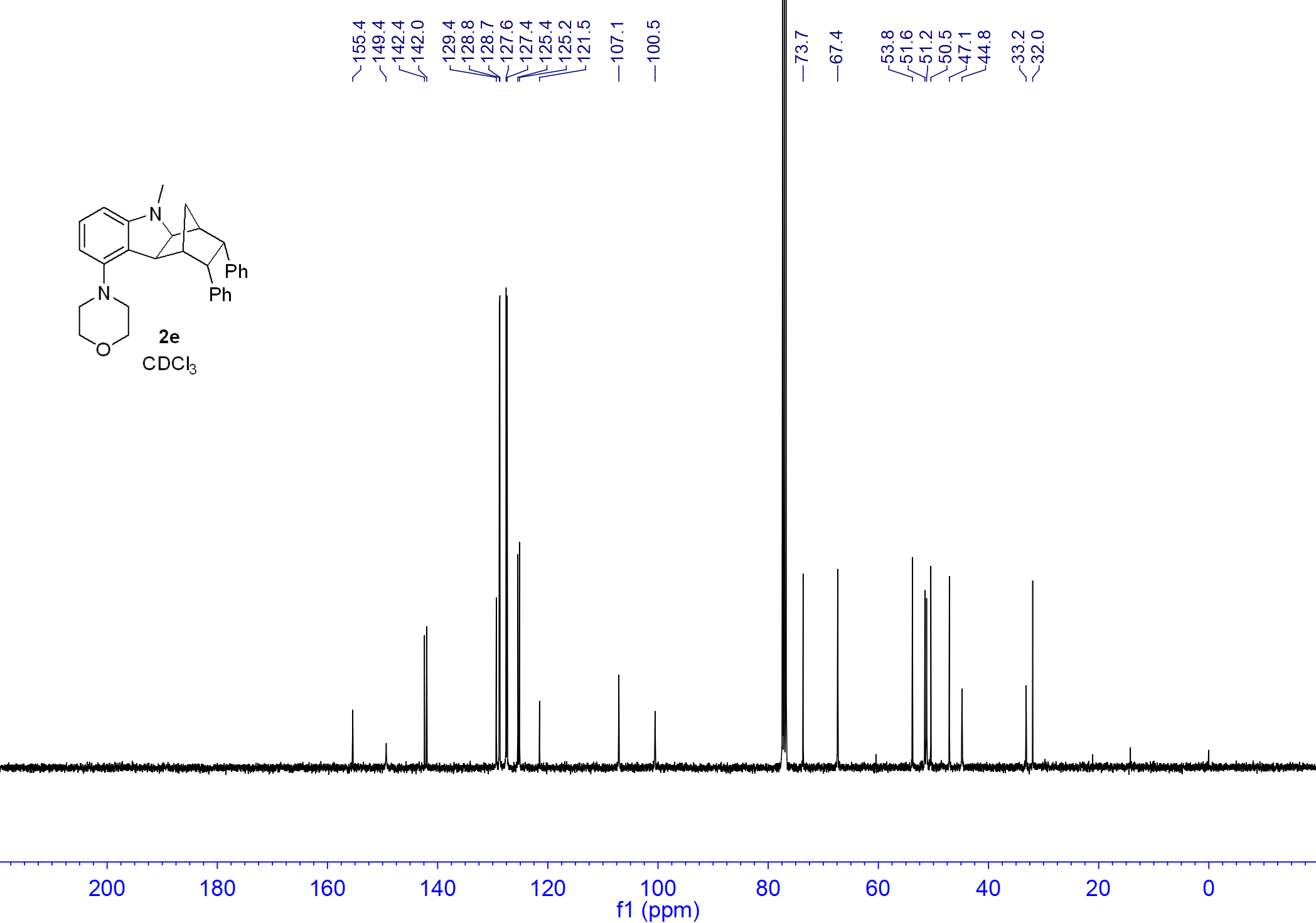


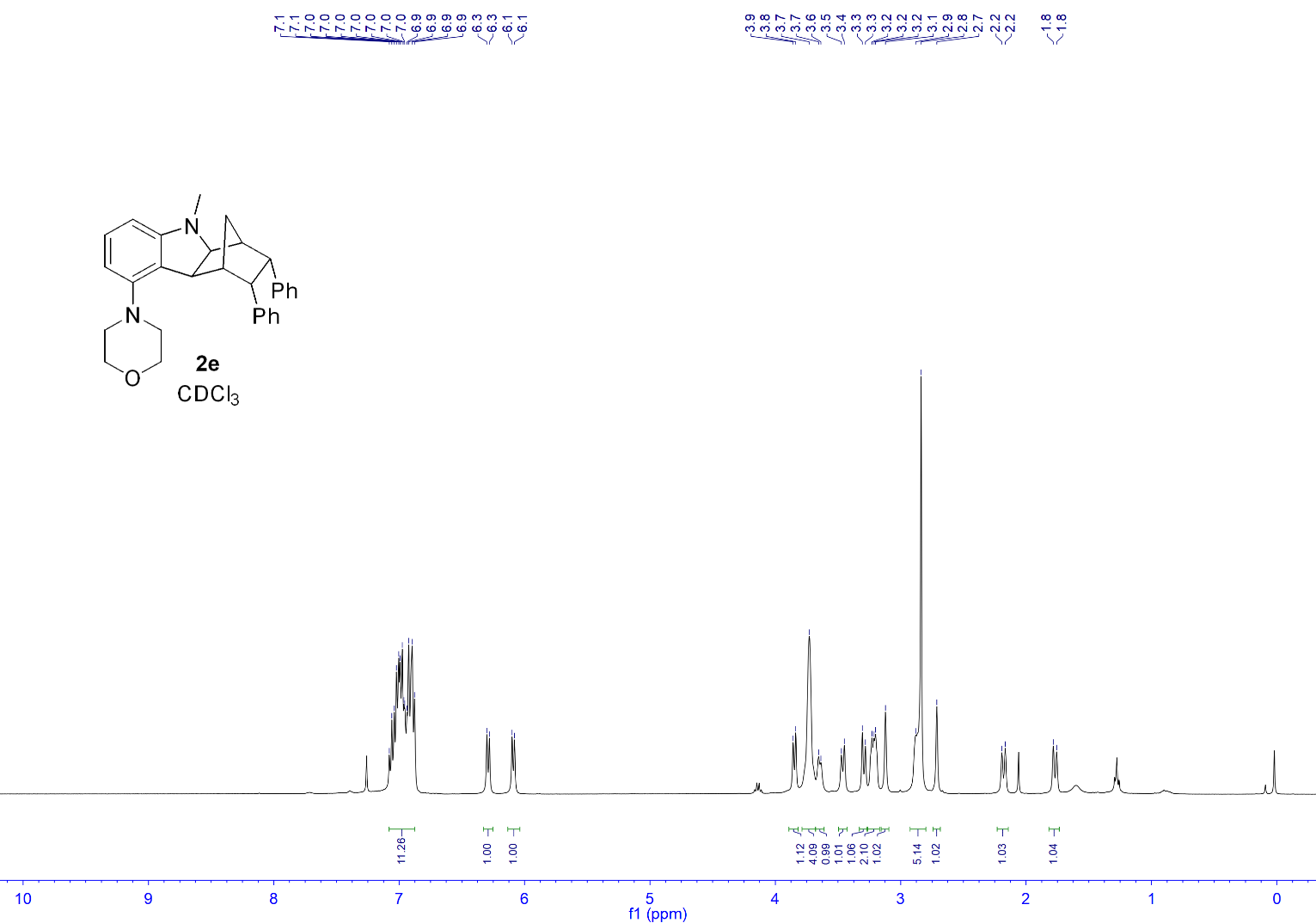

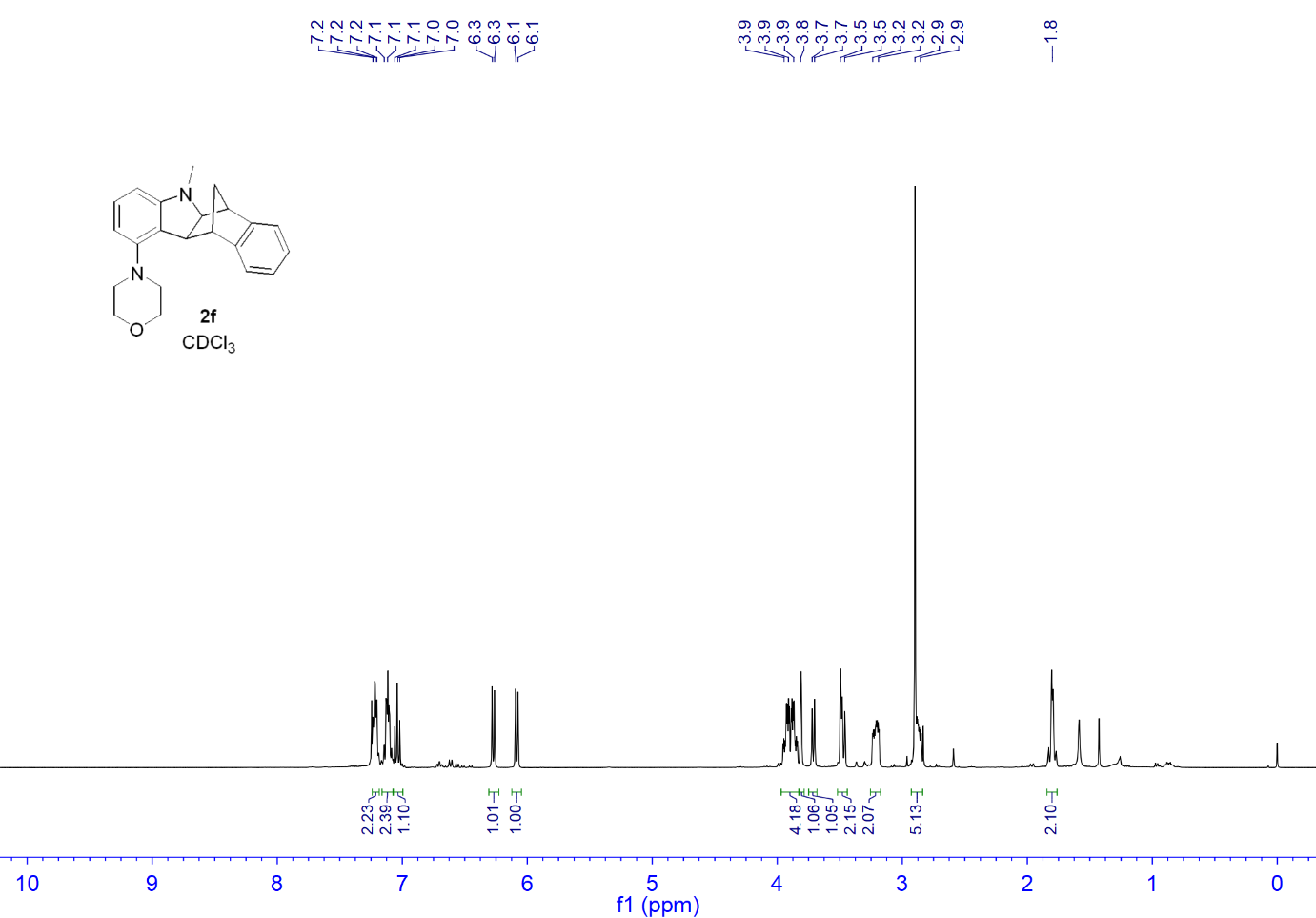


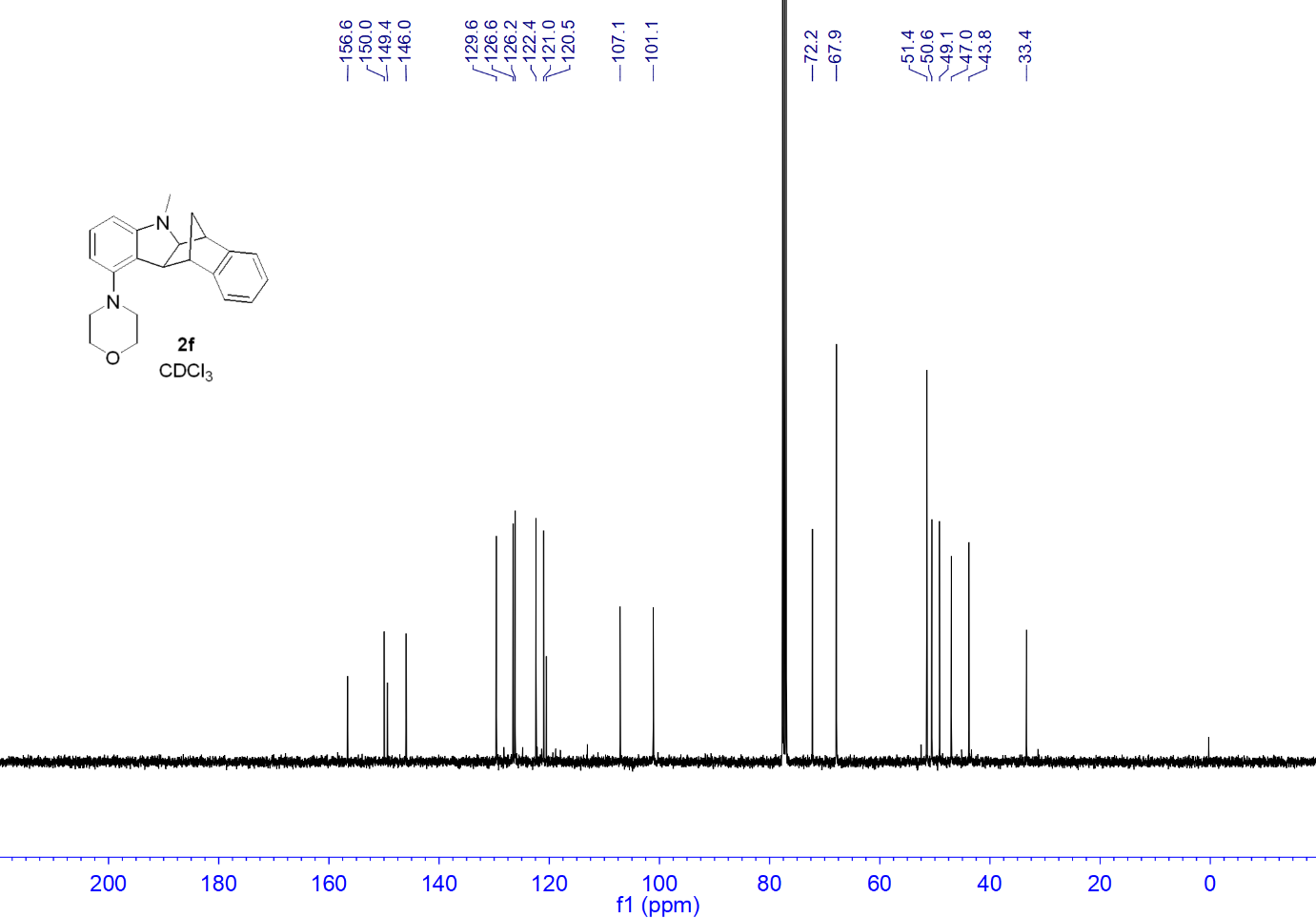


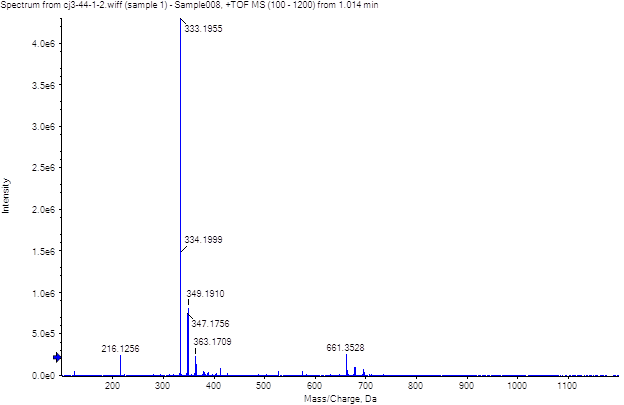


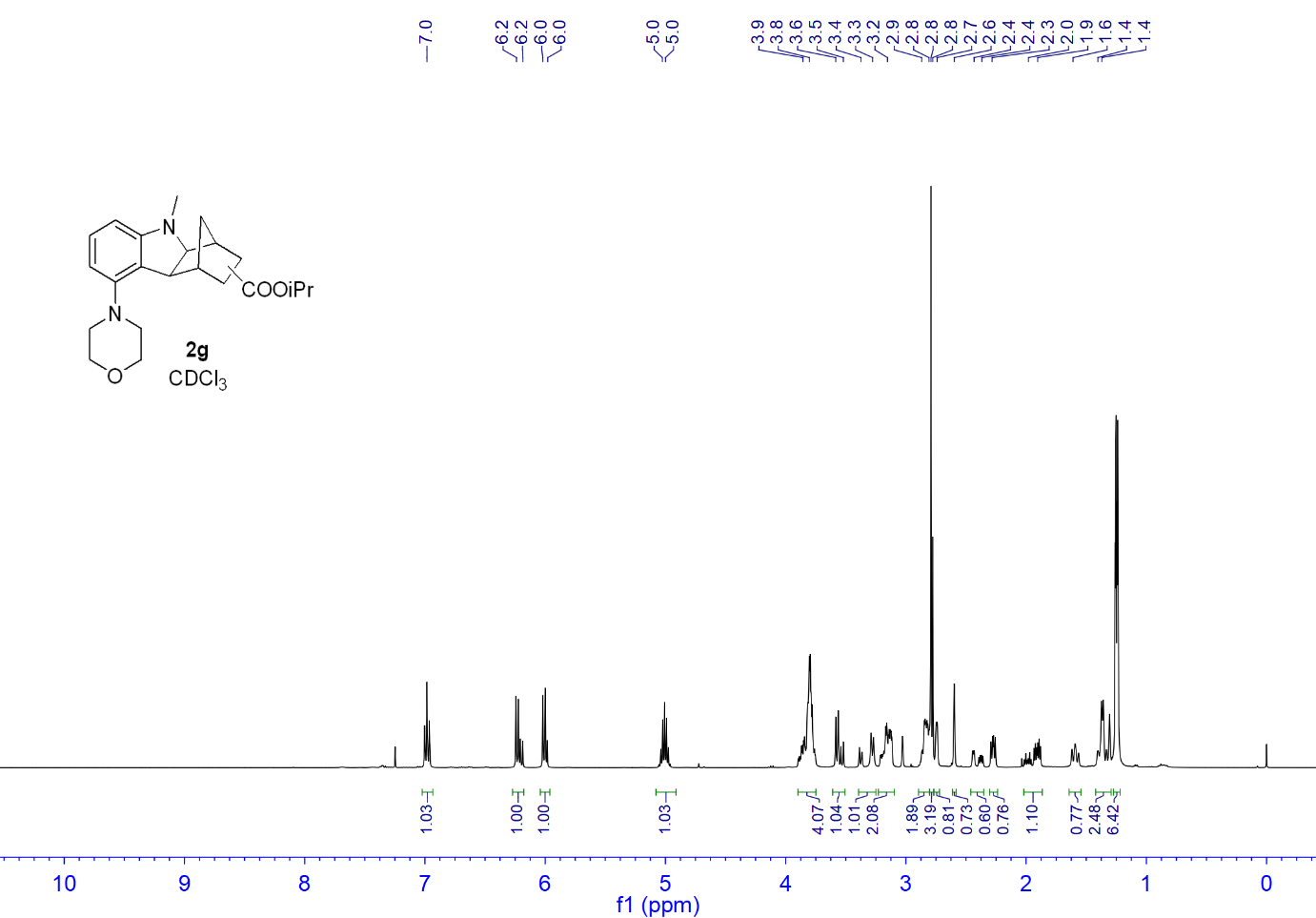


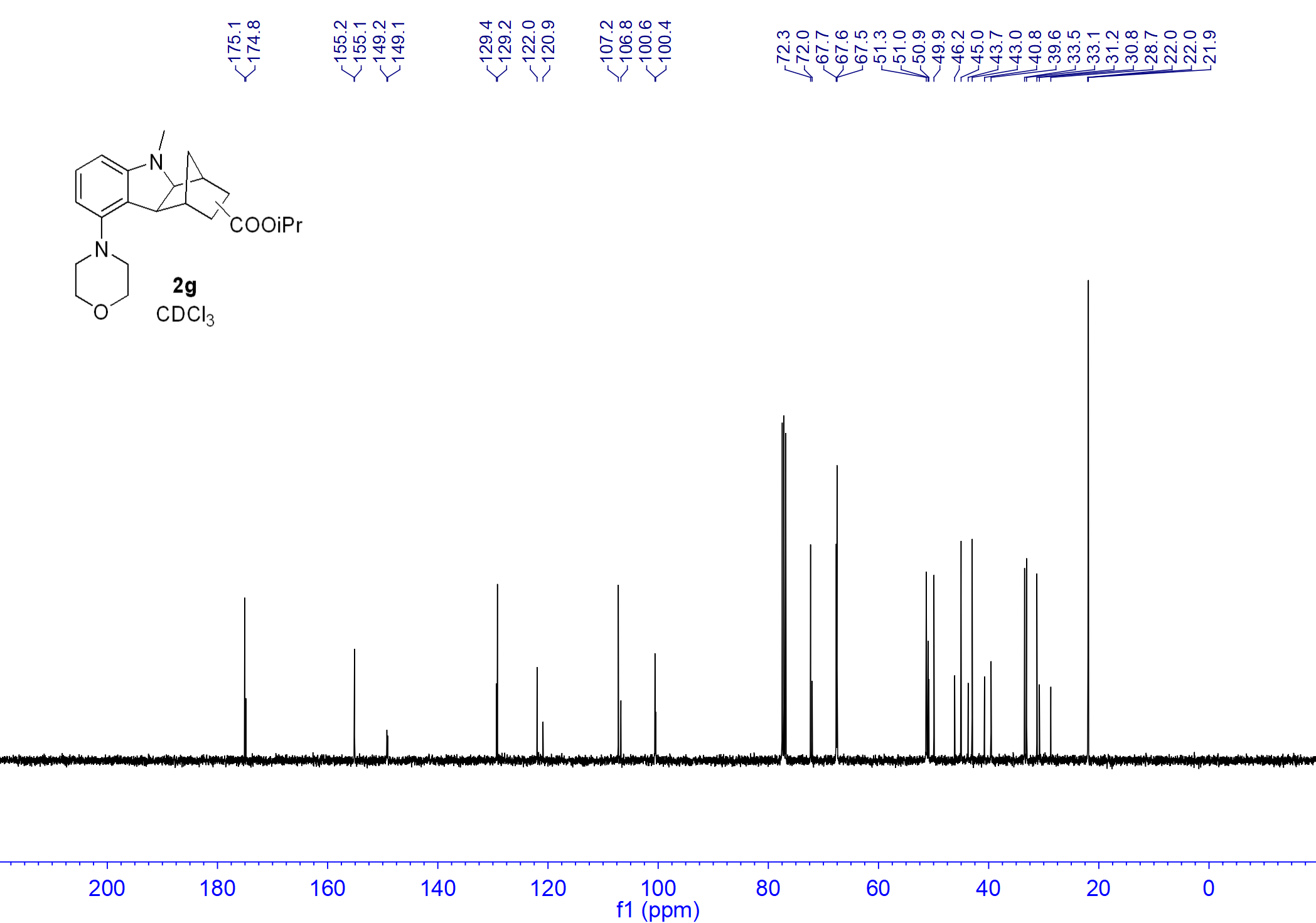

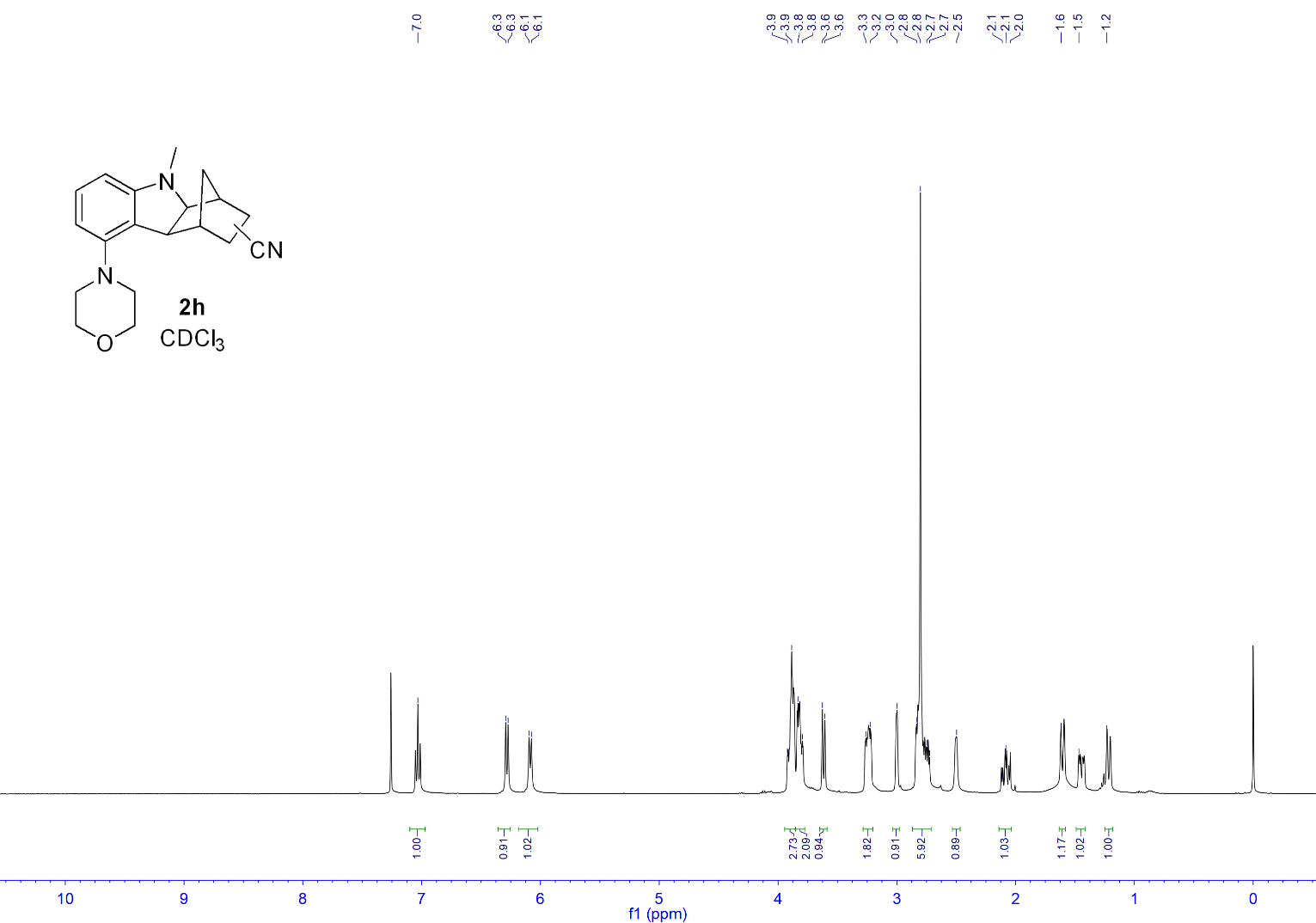


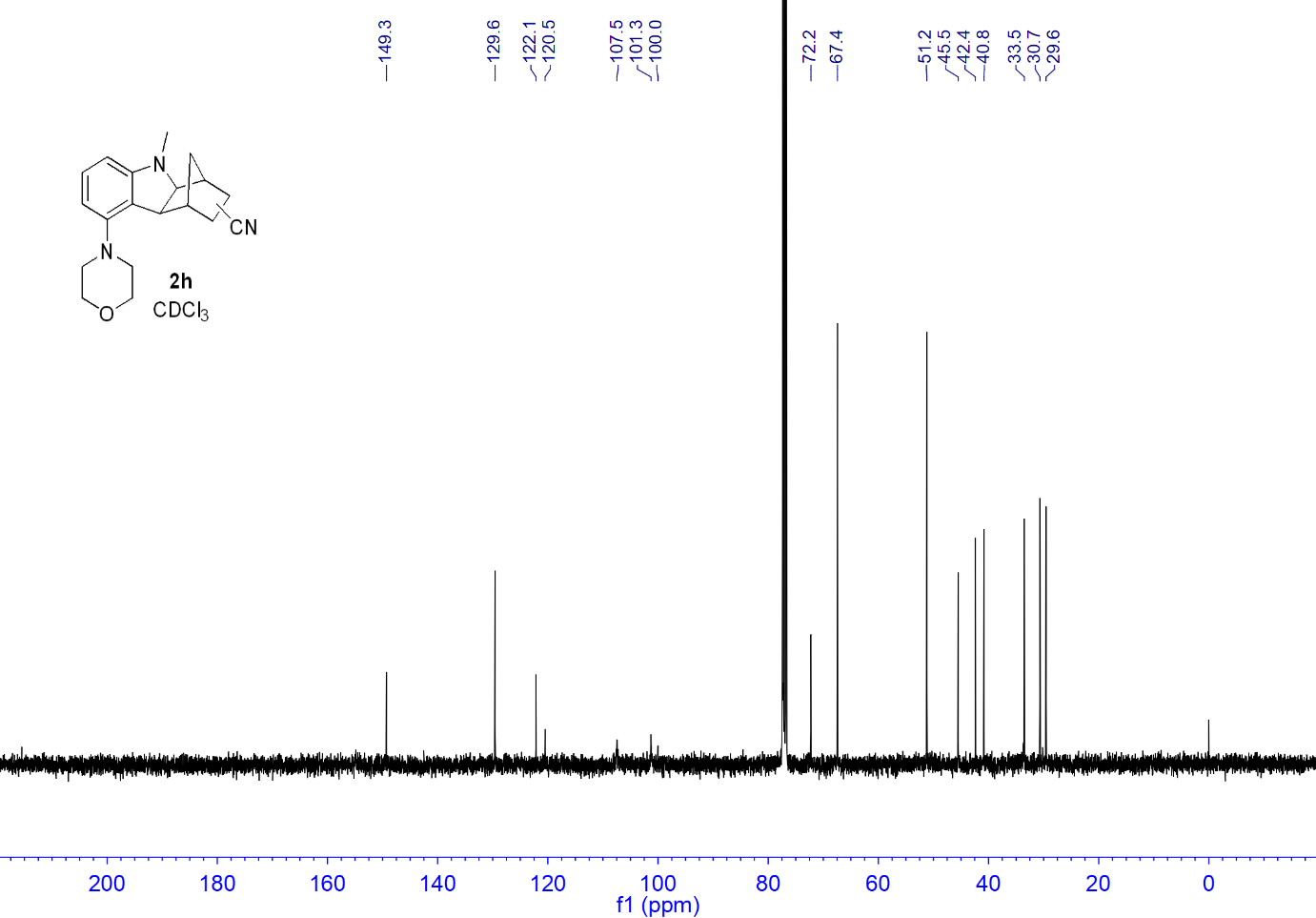

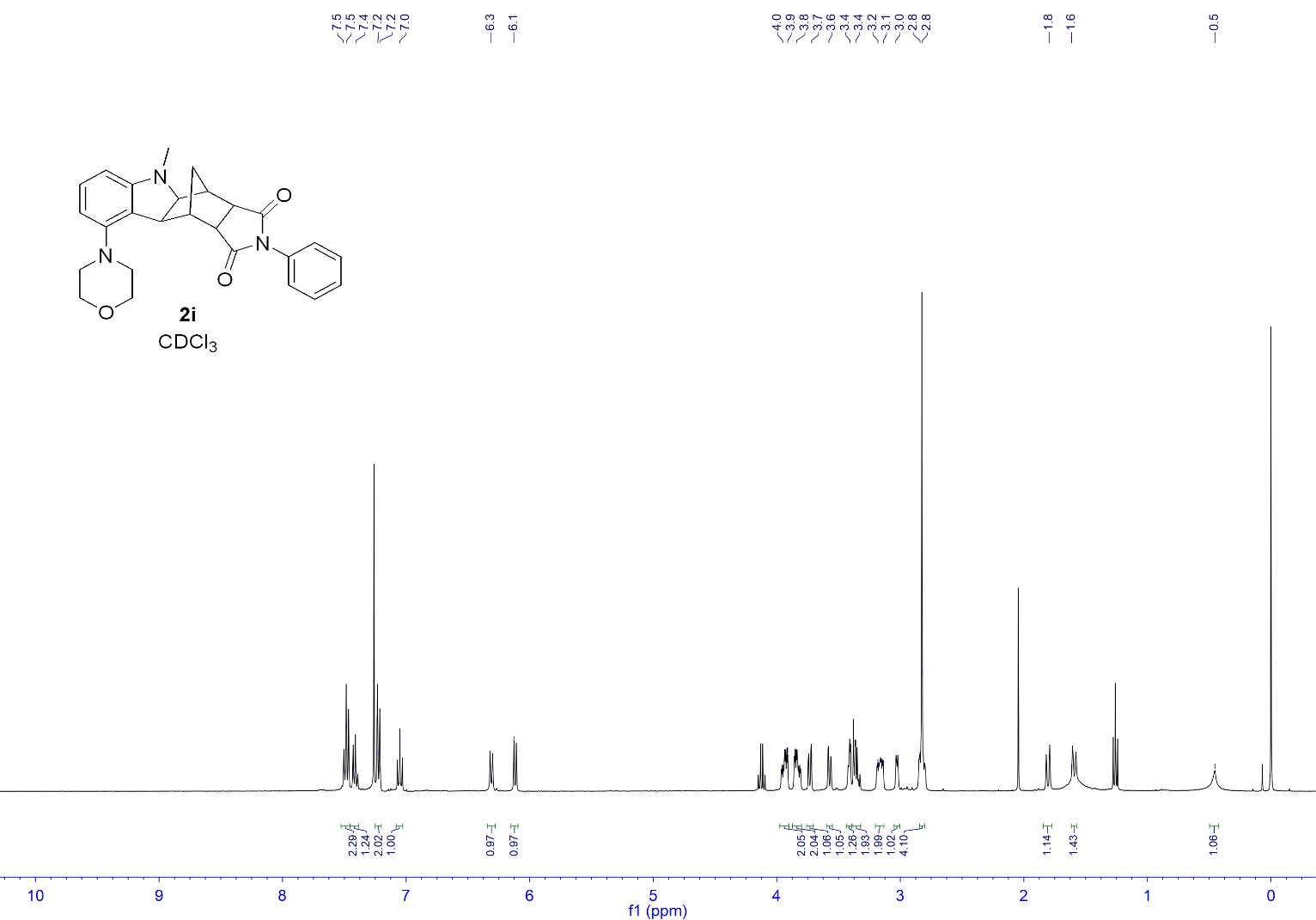


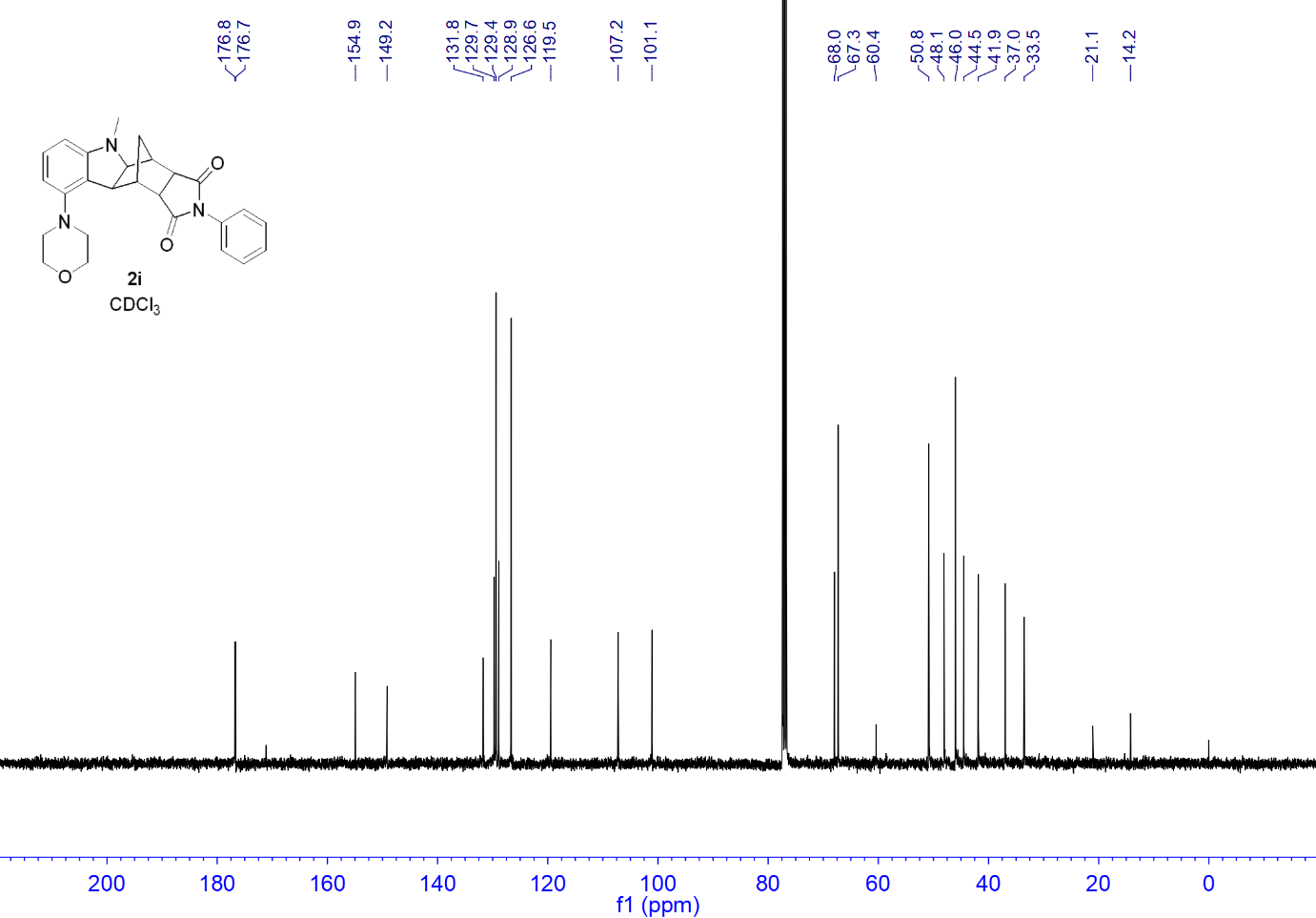

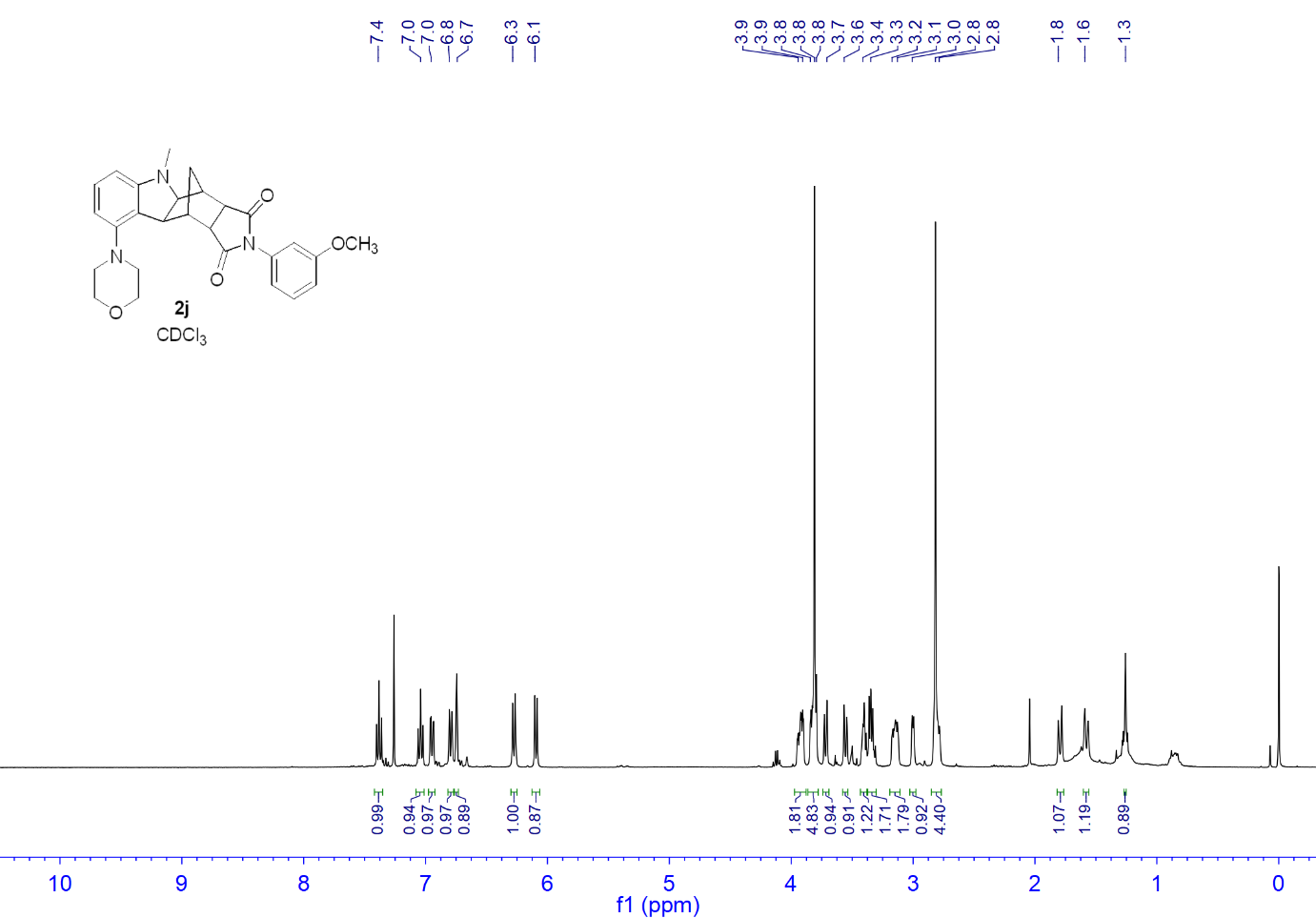


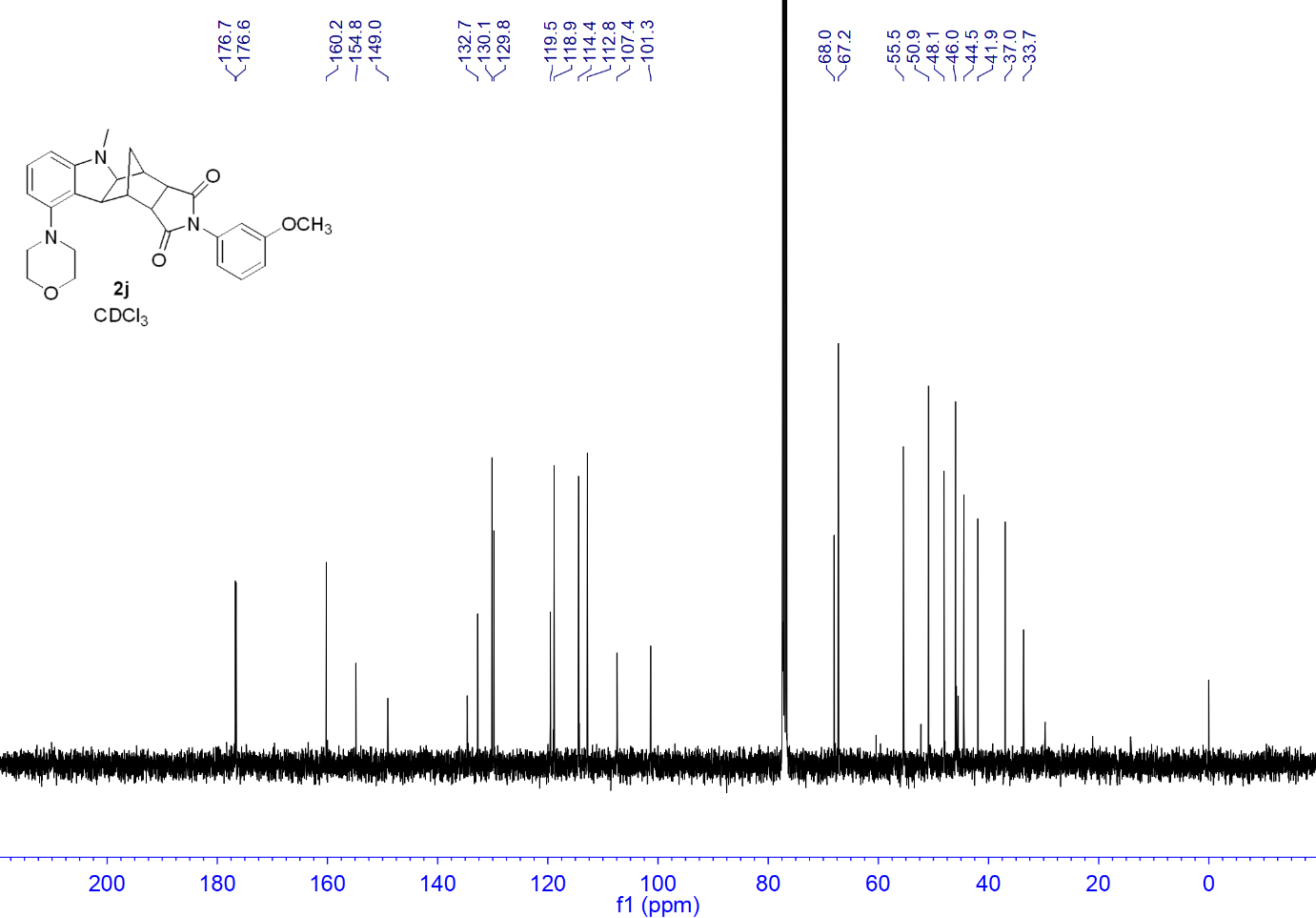

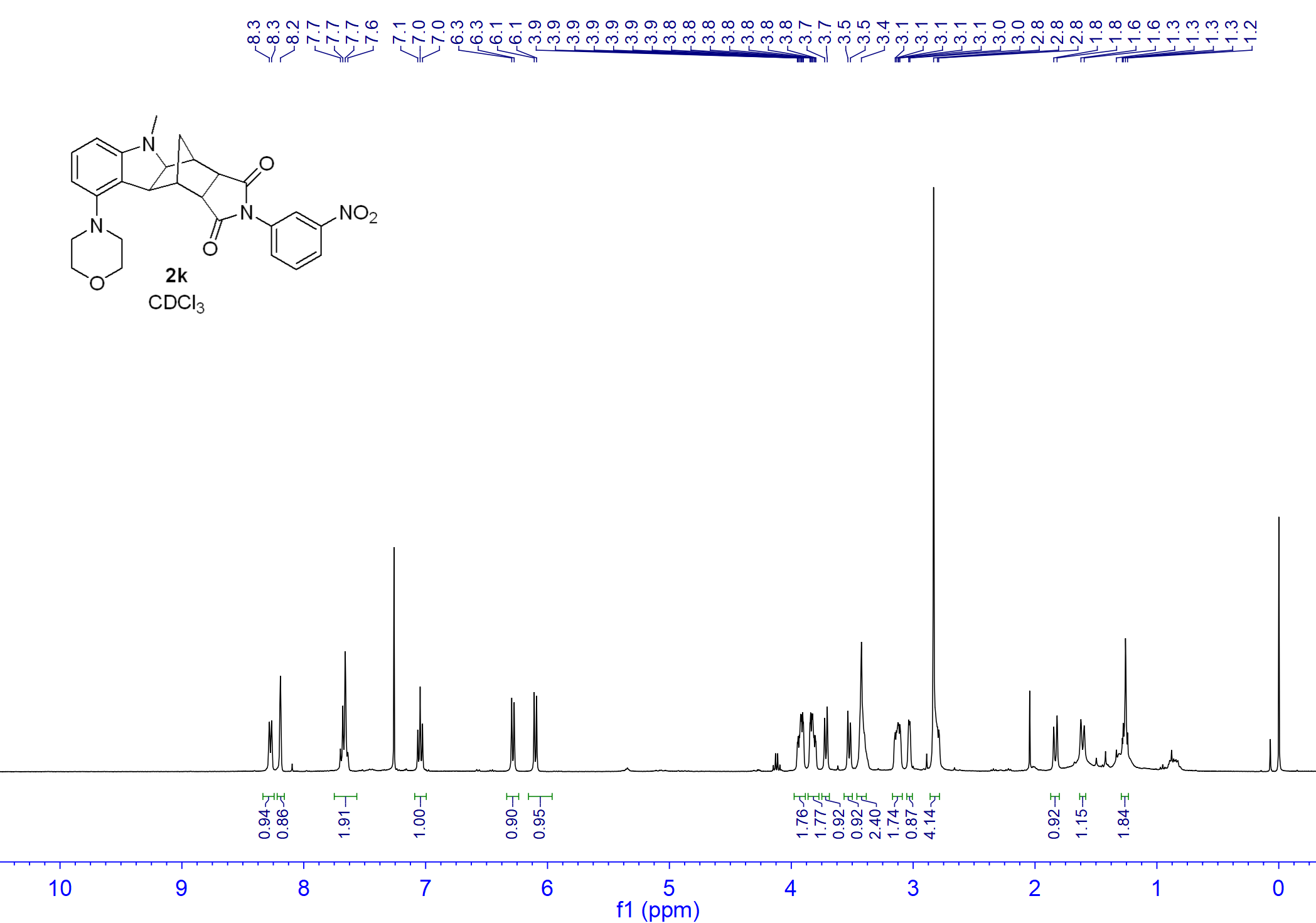


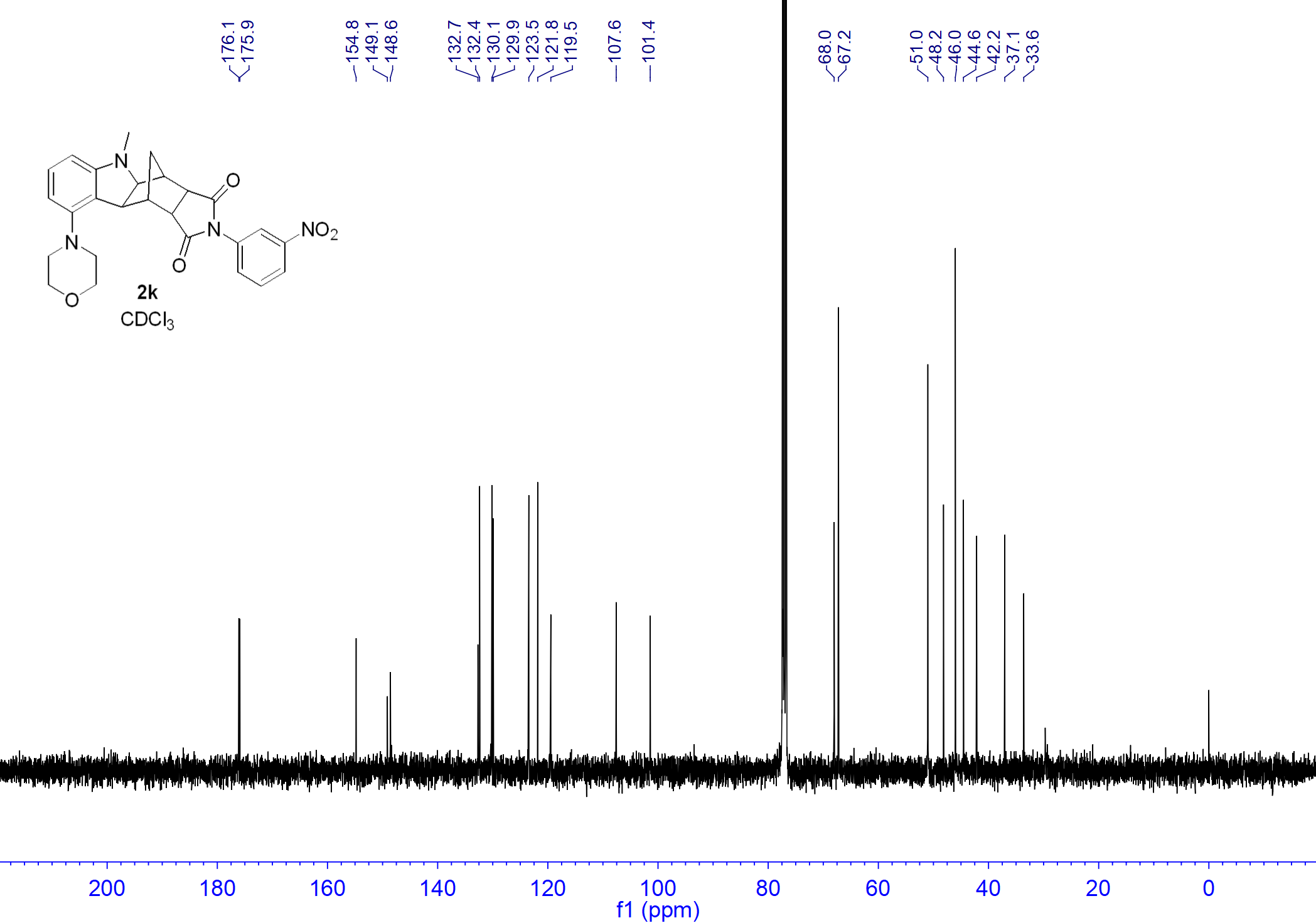

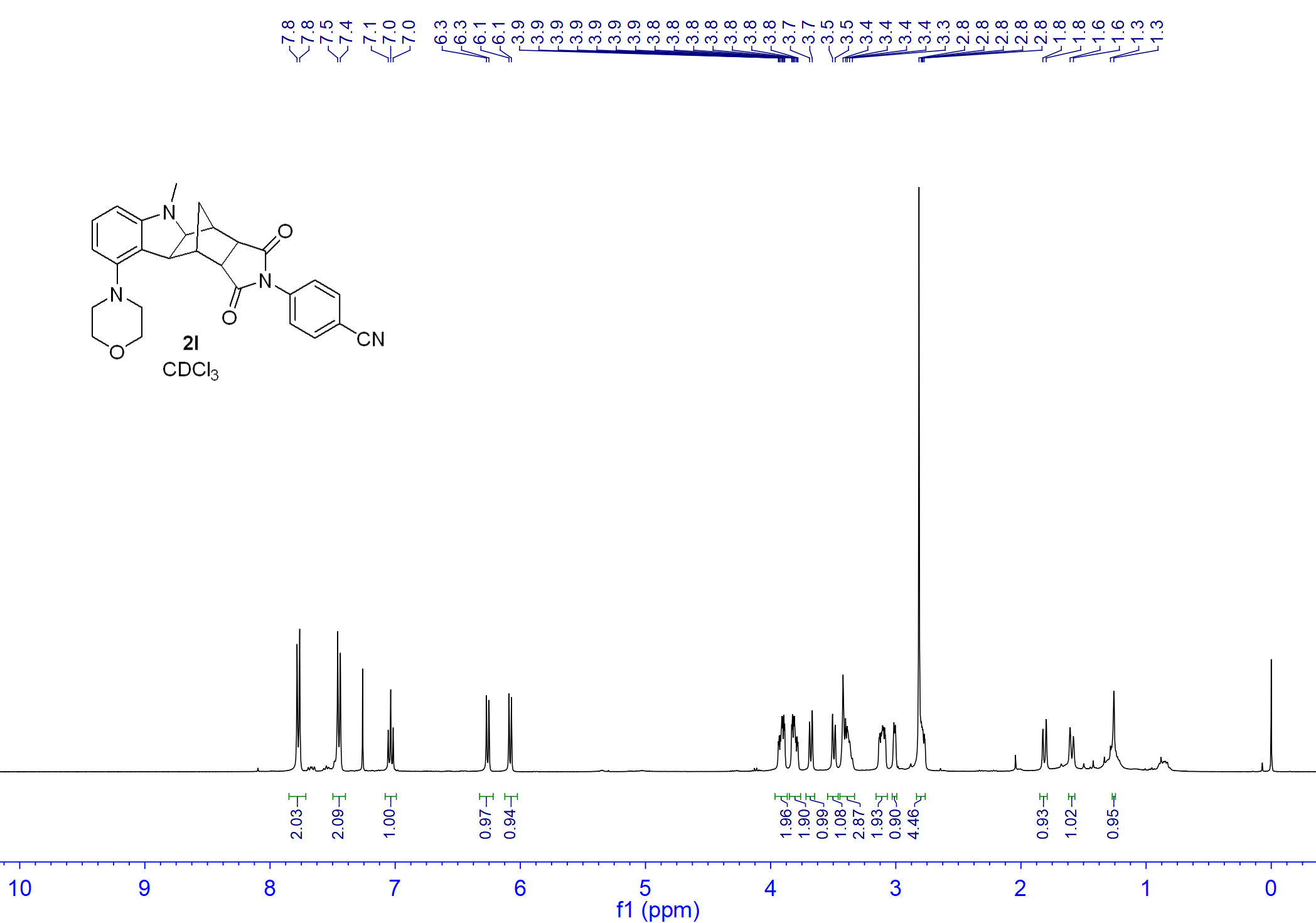


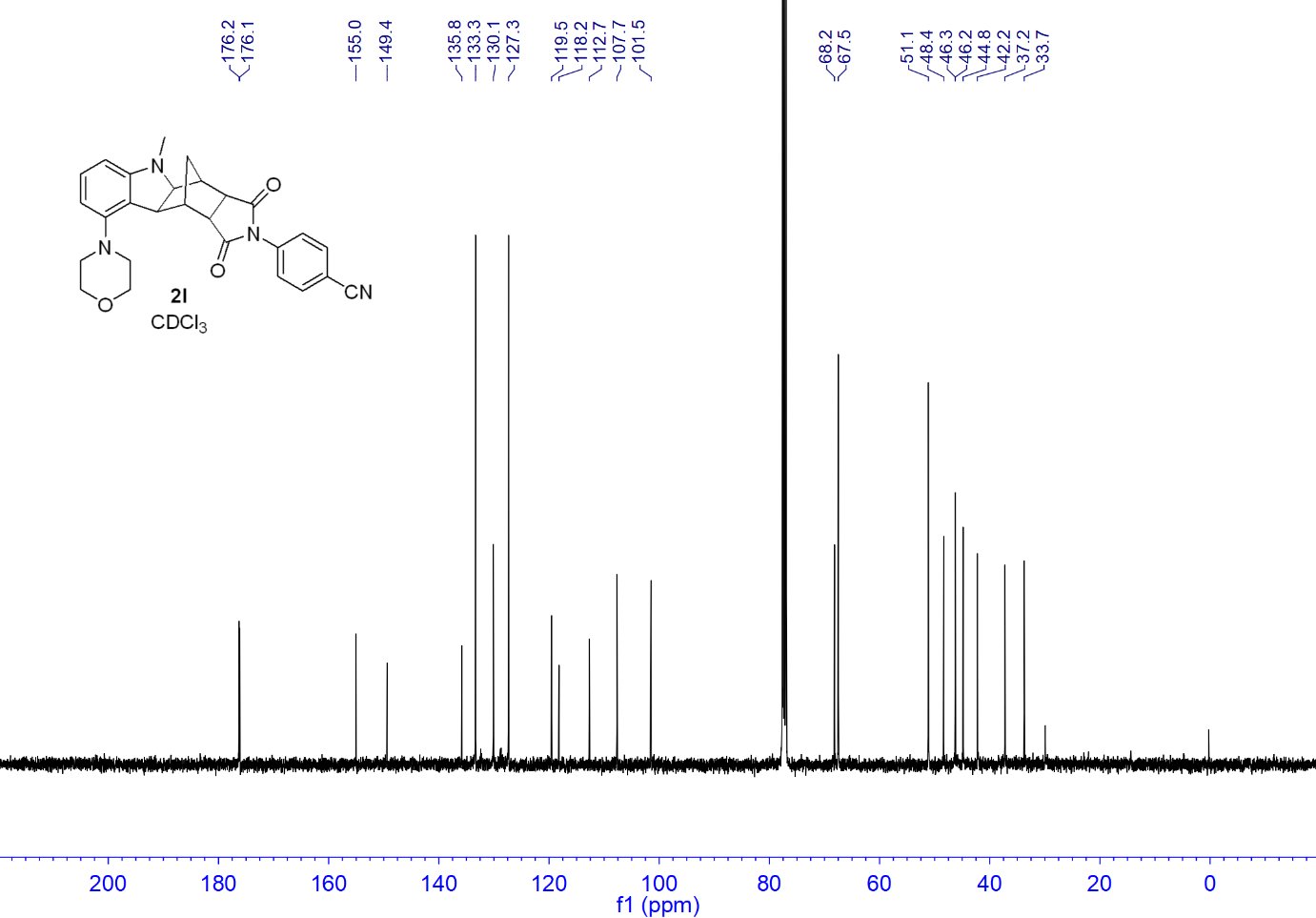

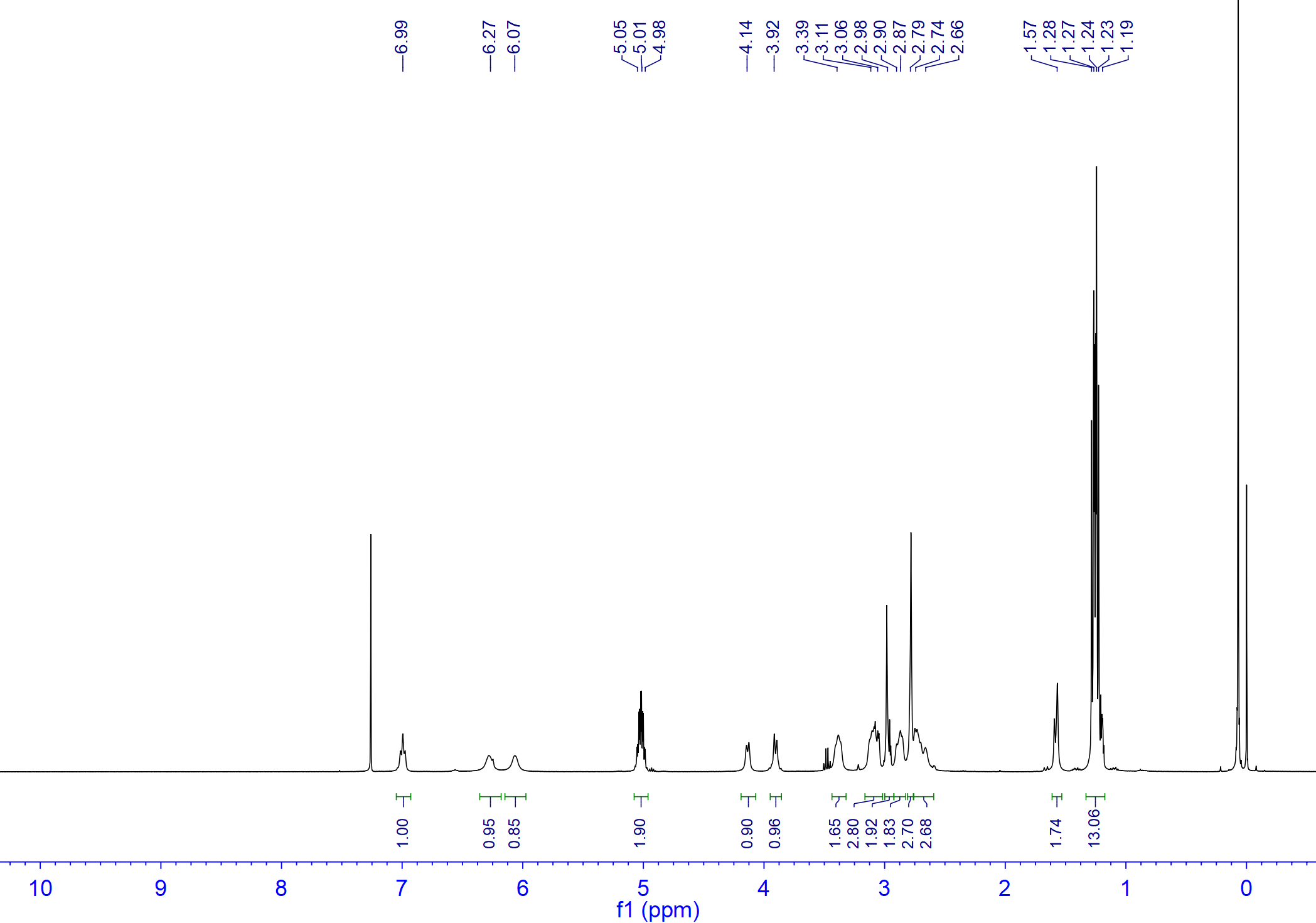


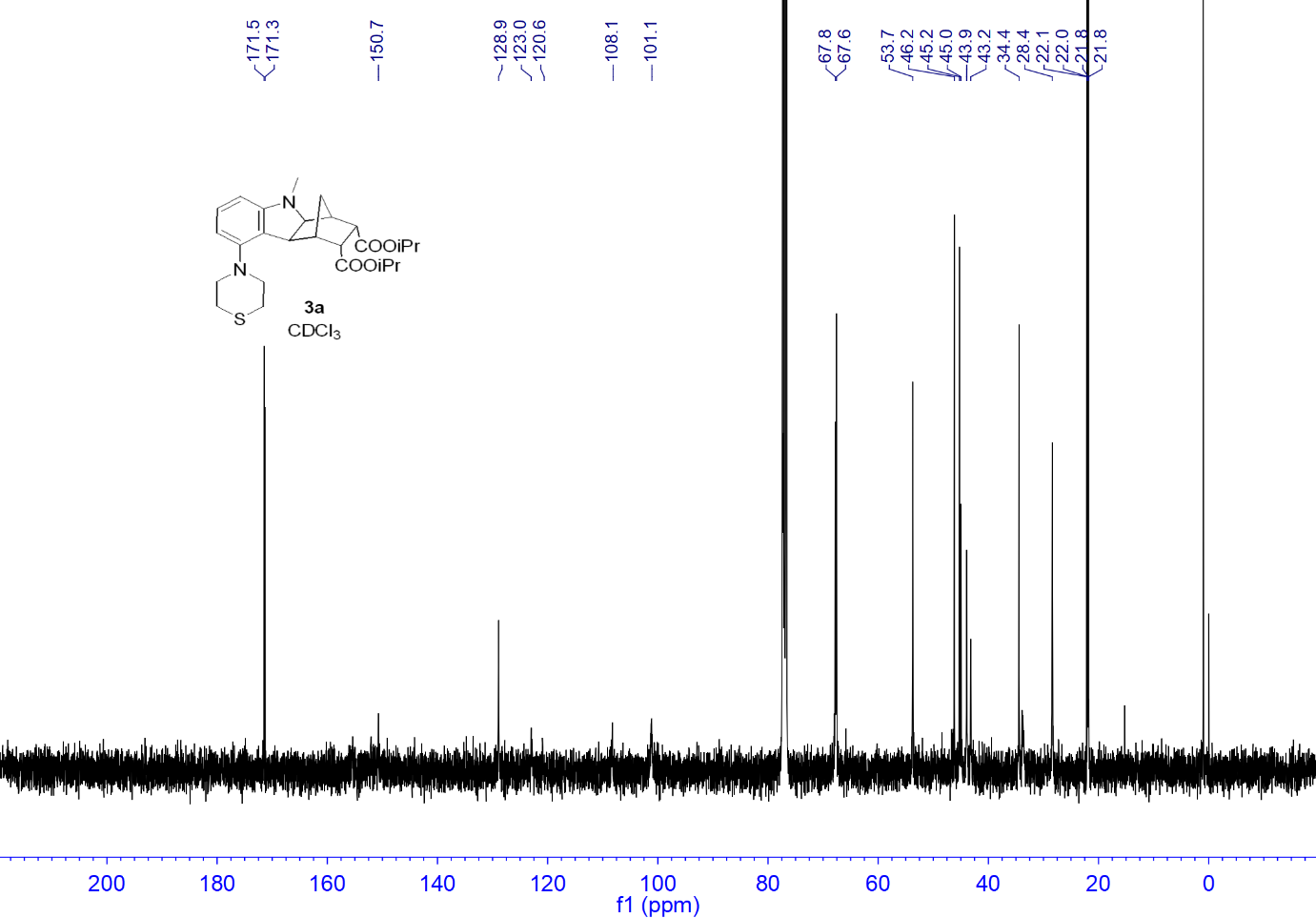

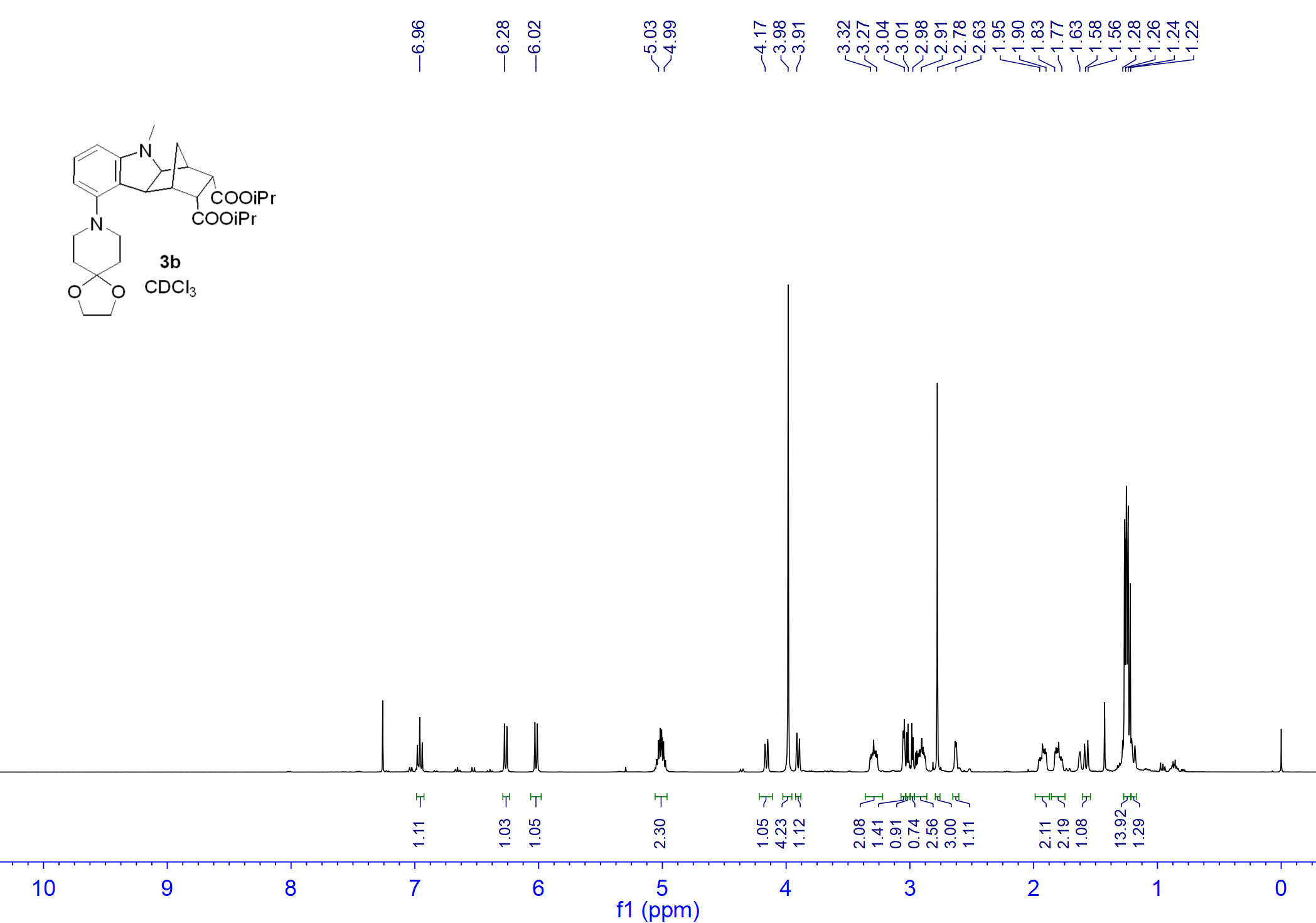


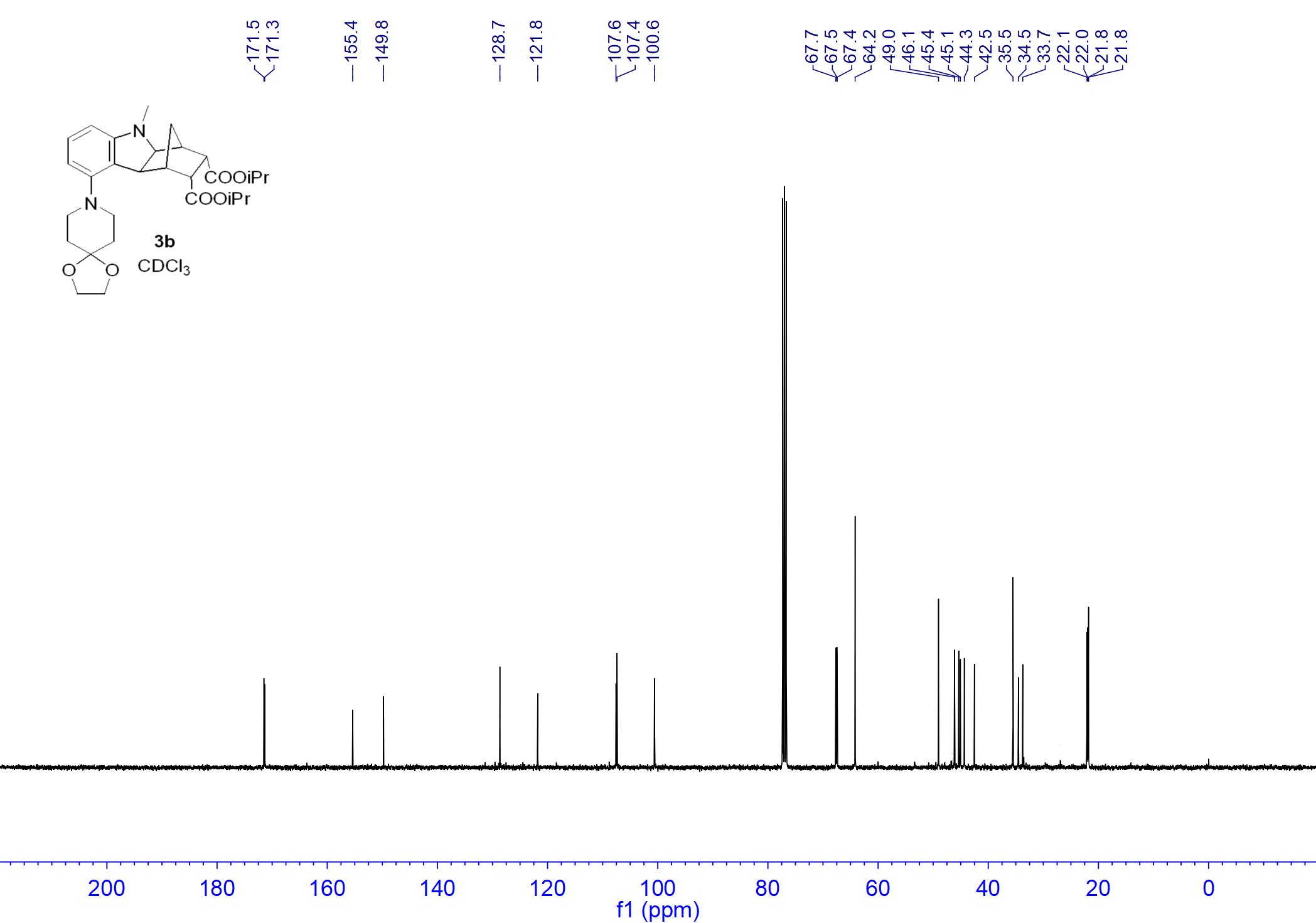

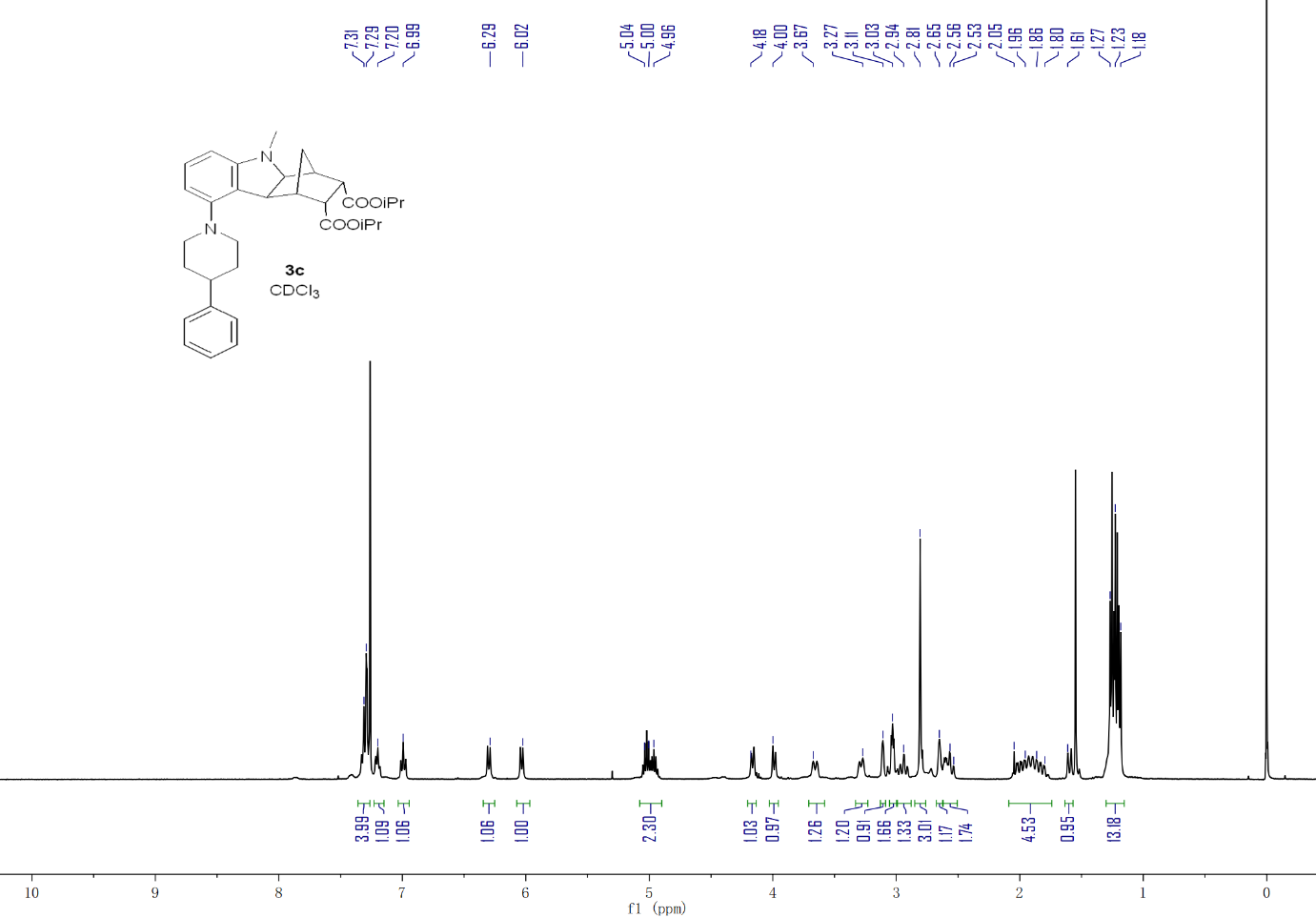


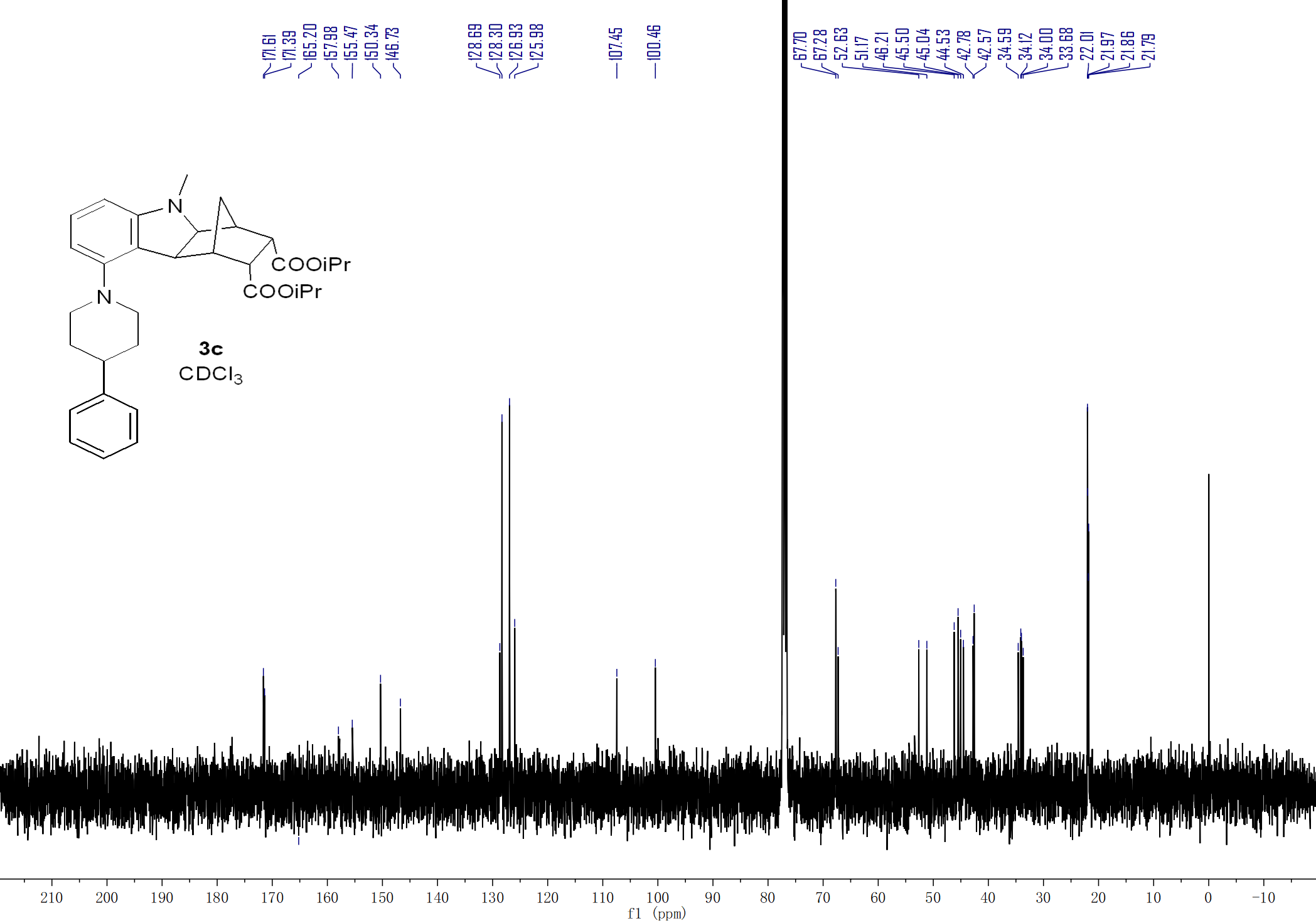

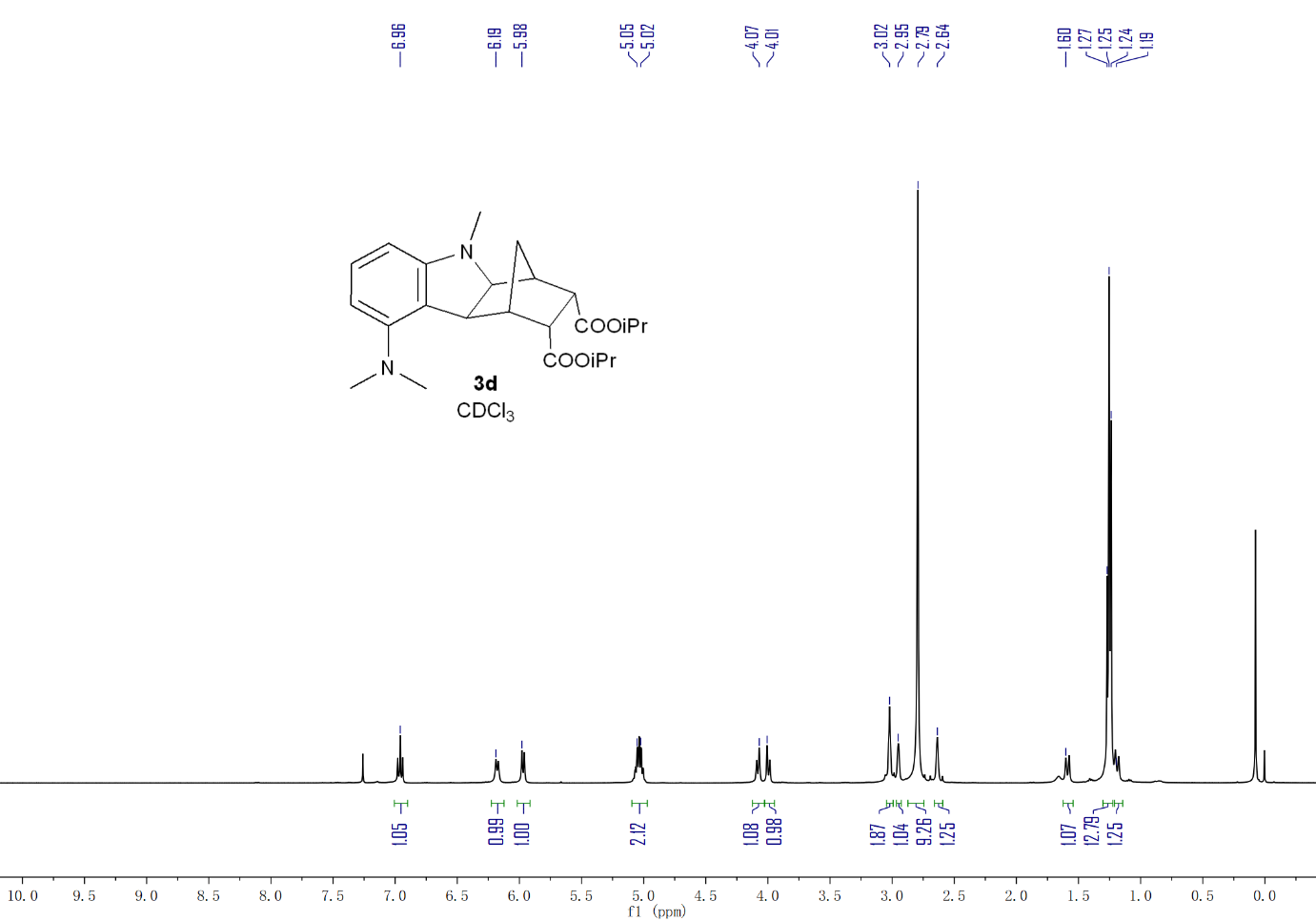


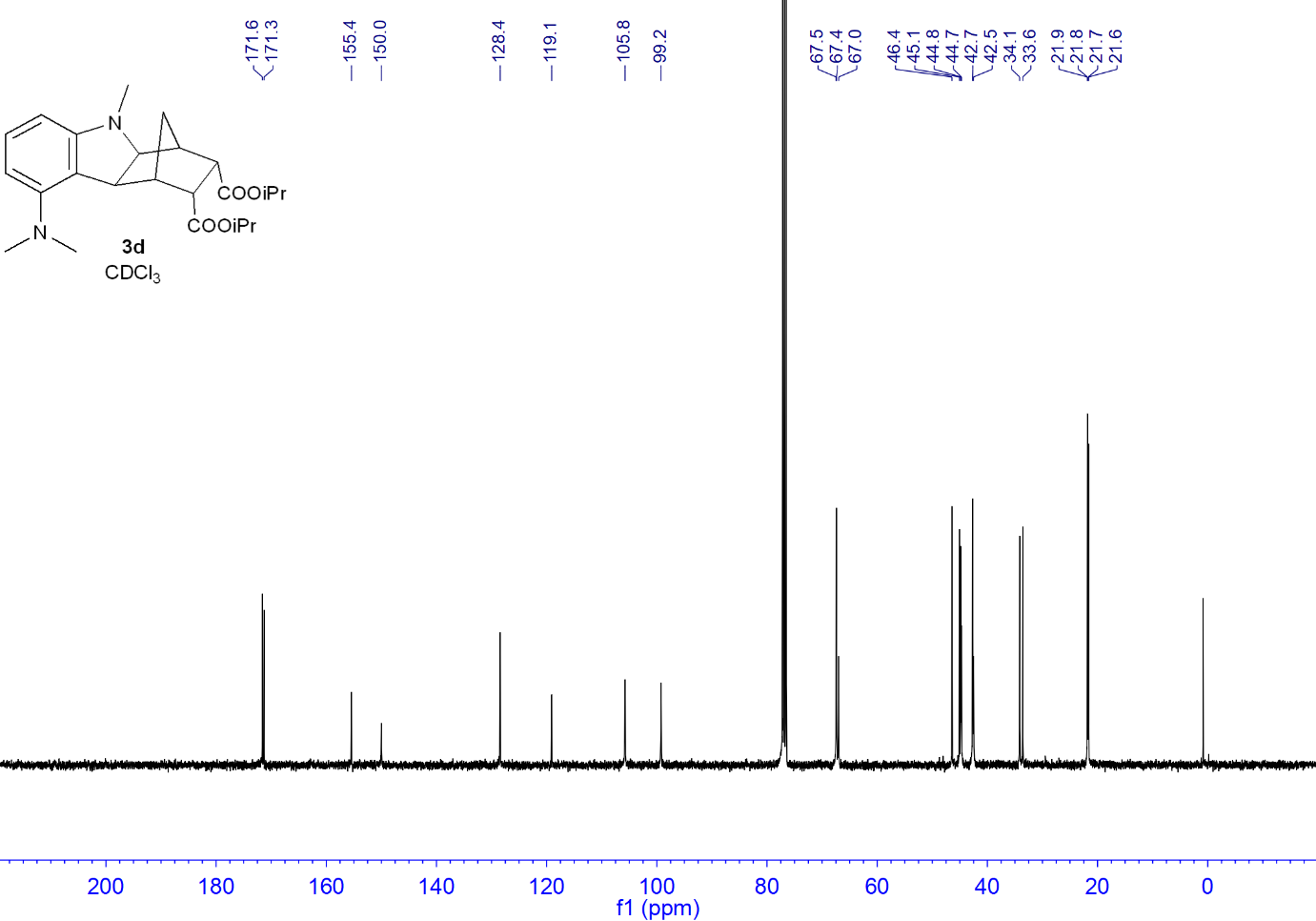


s

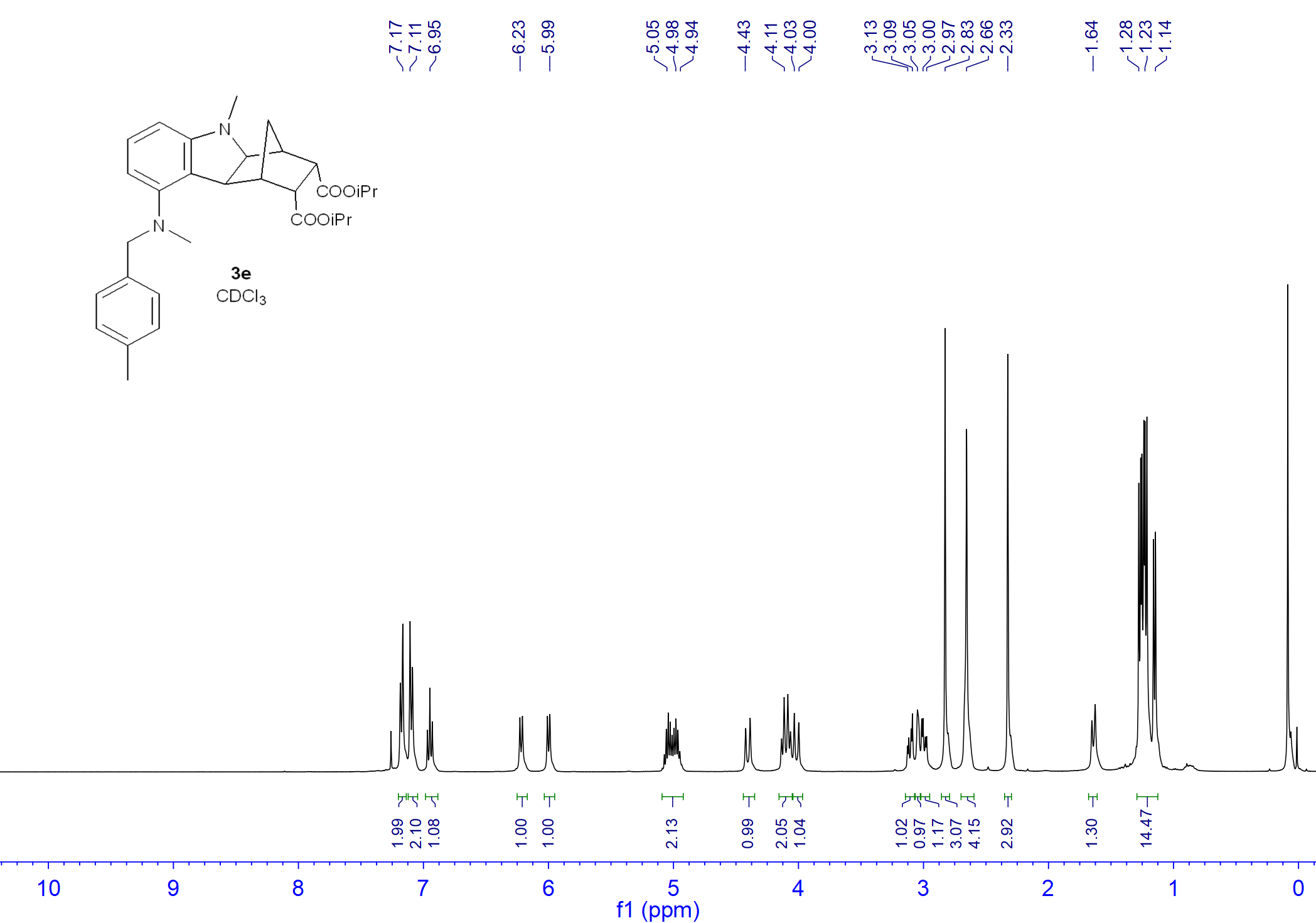


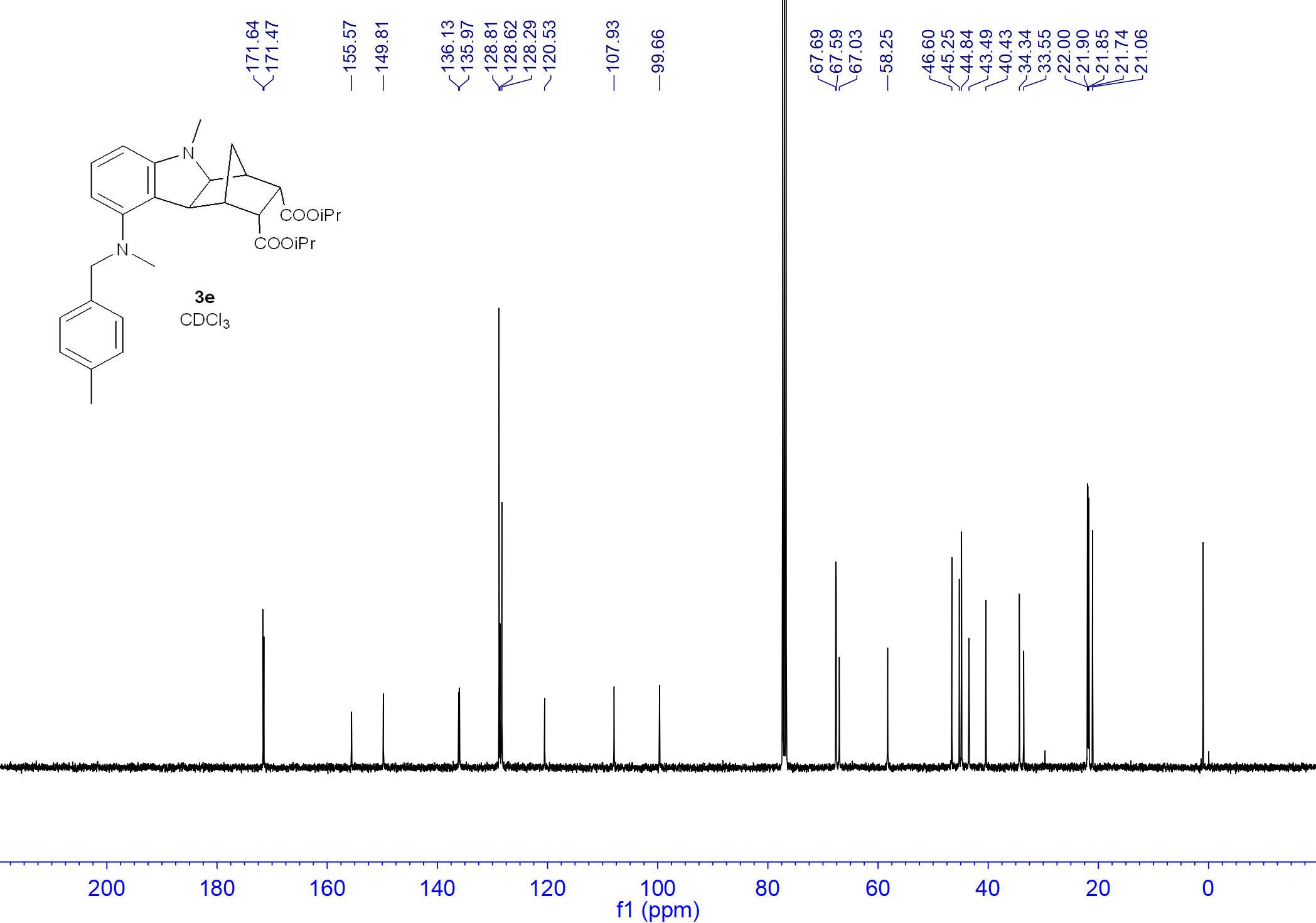

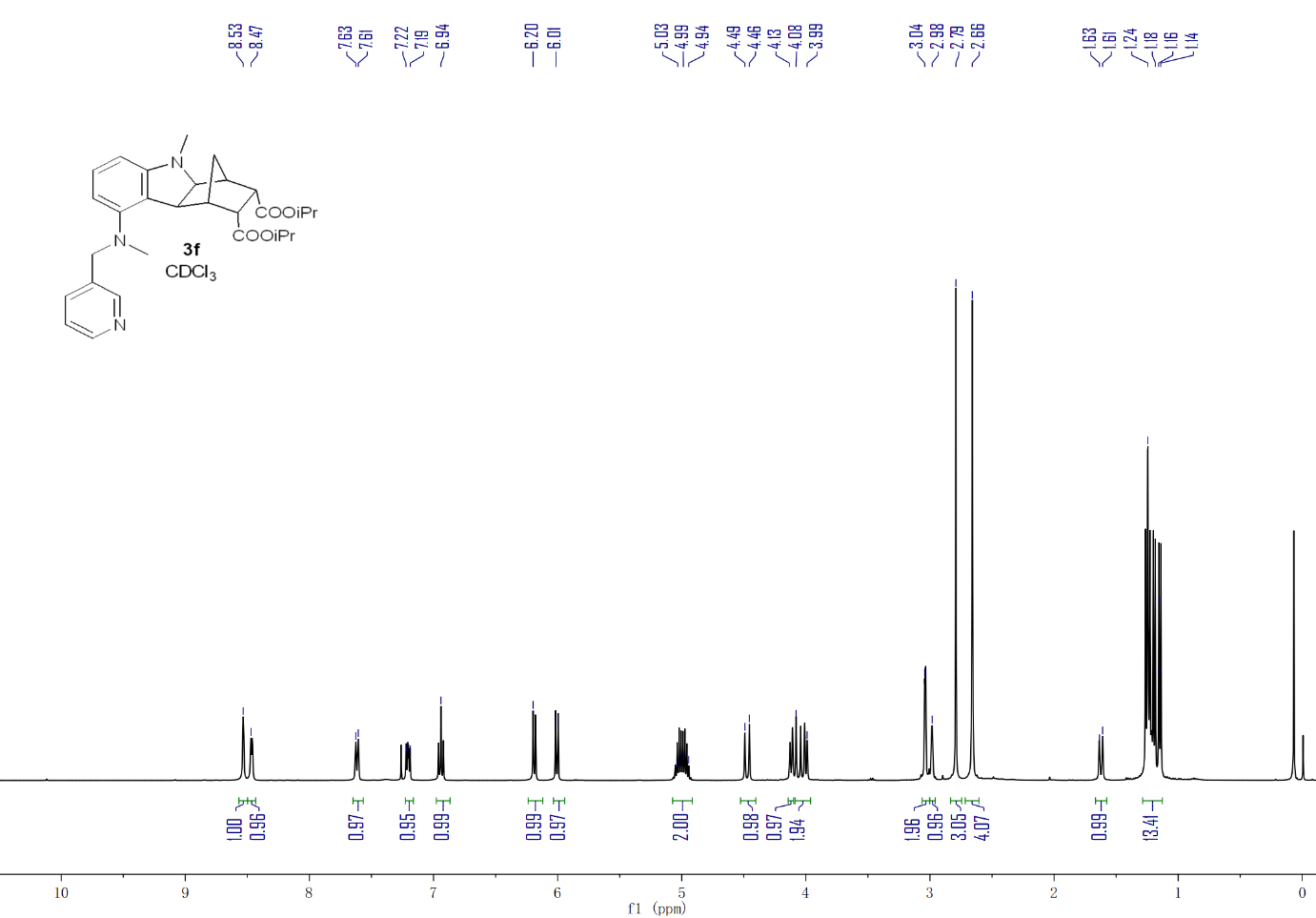


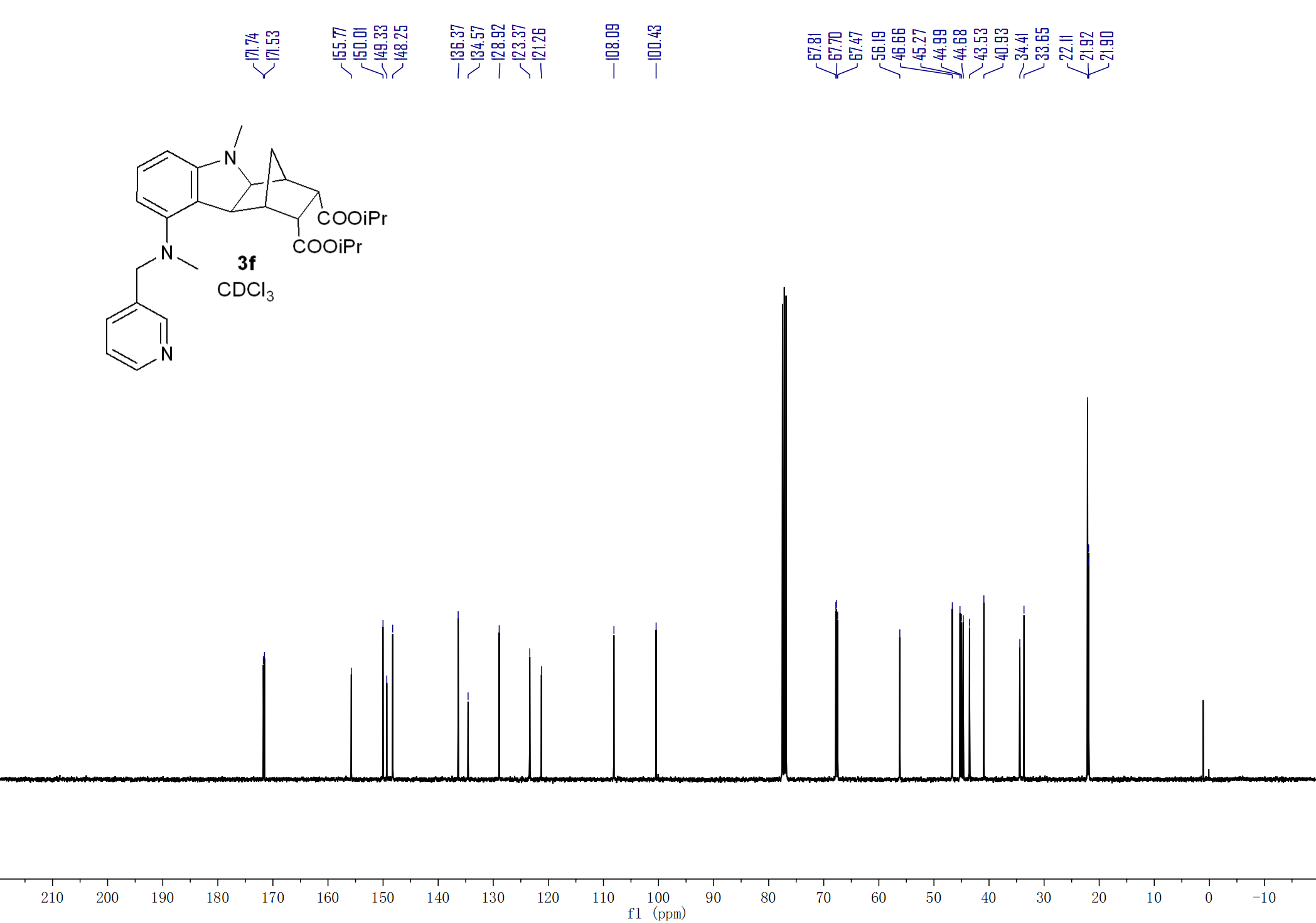

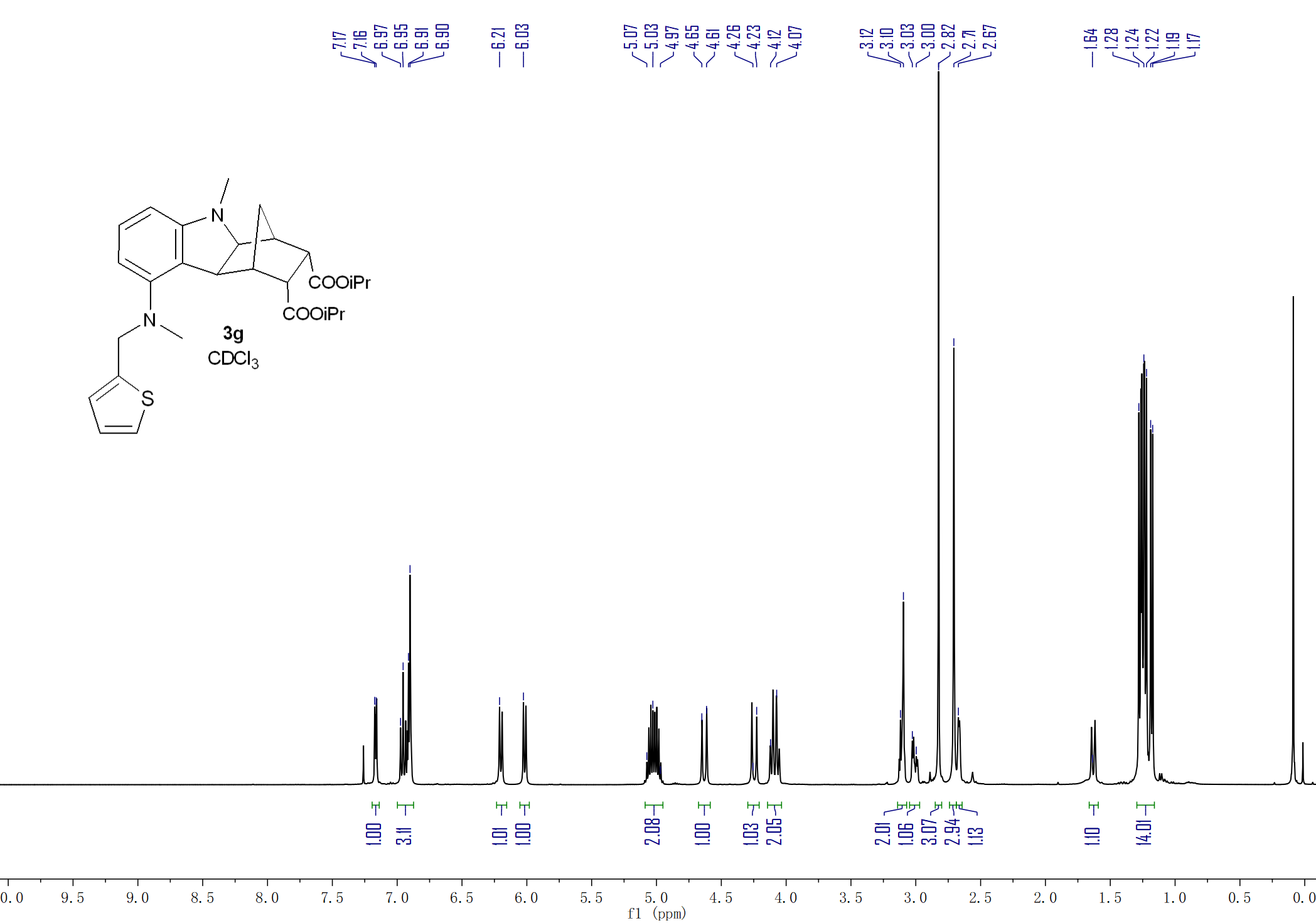


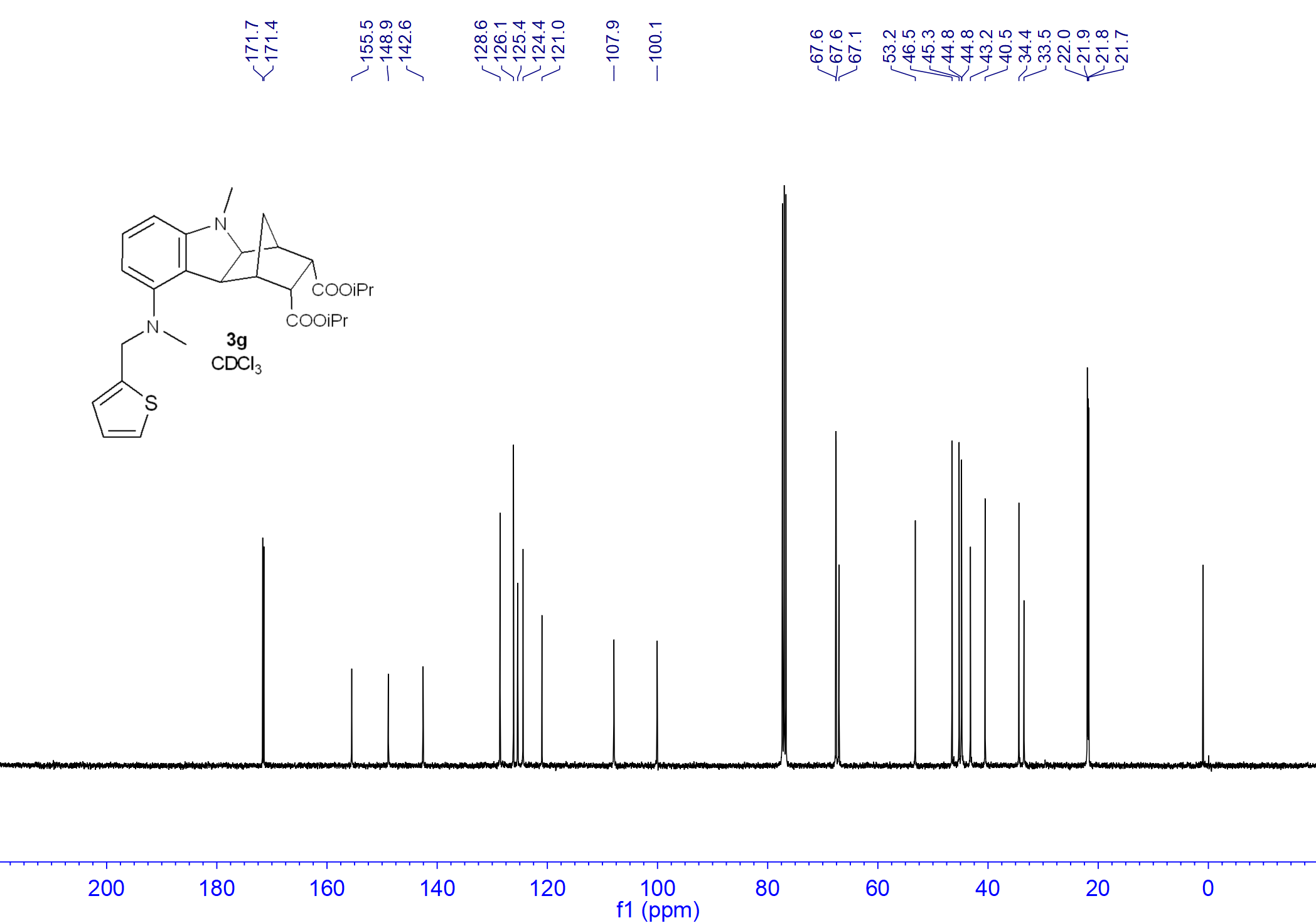

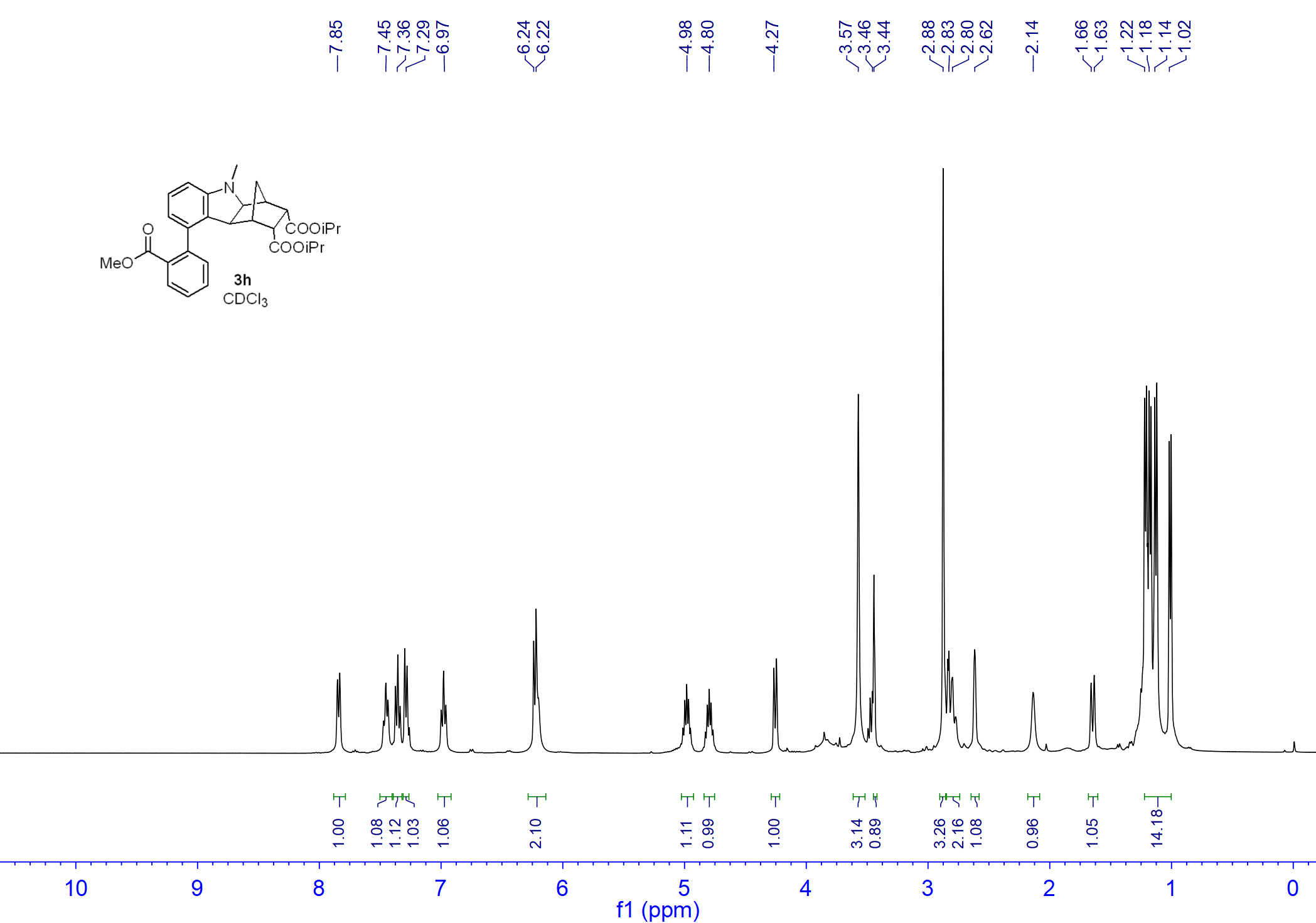


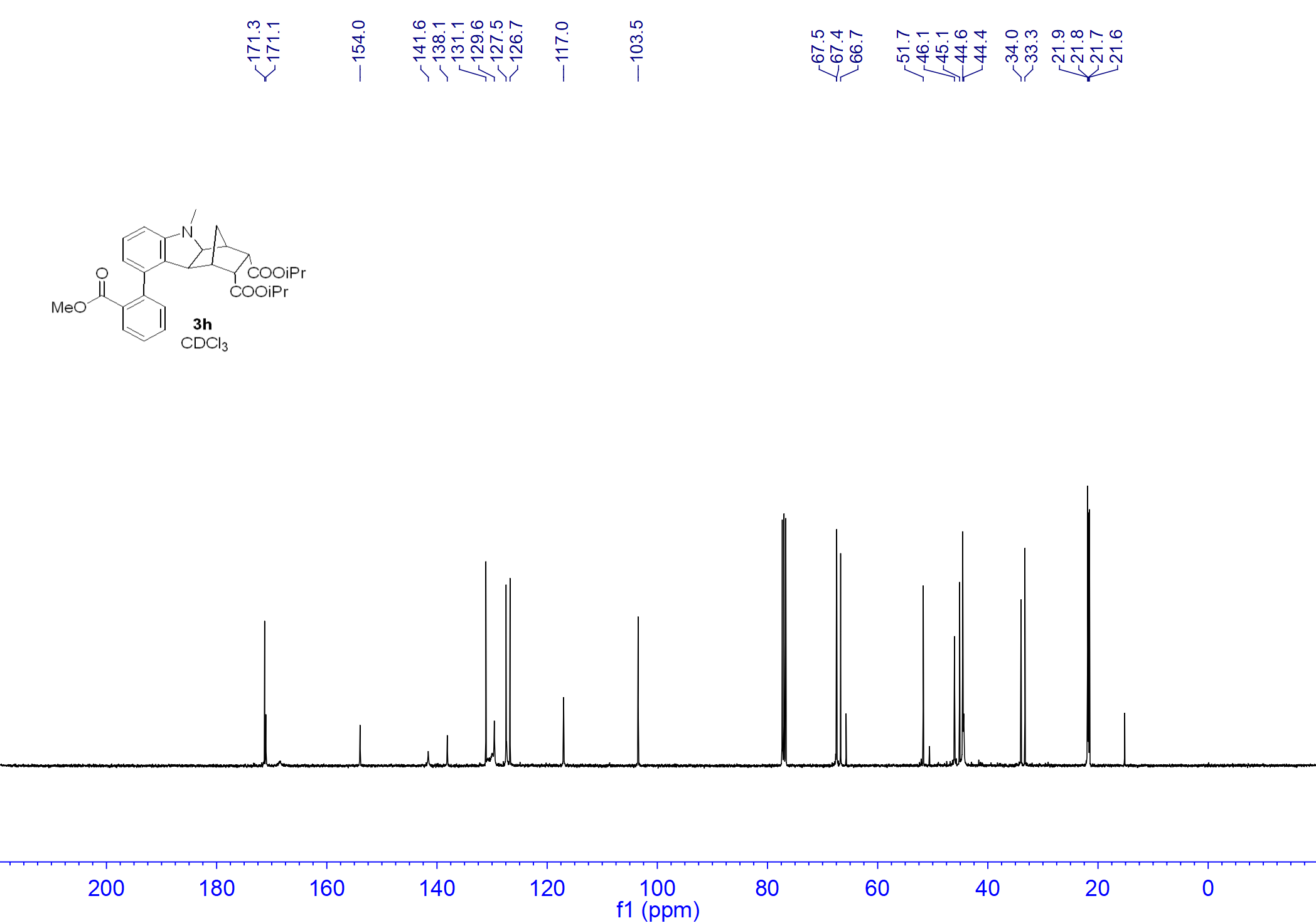

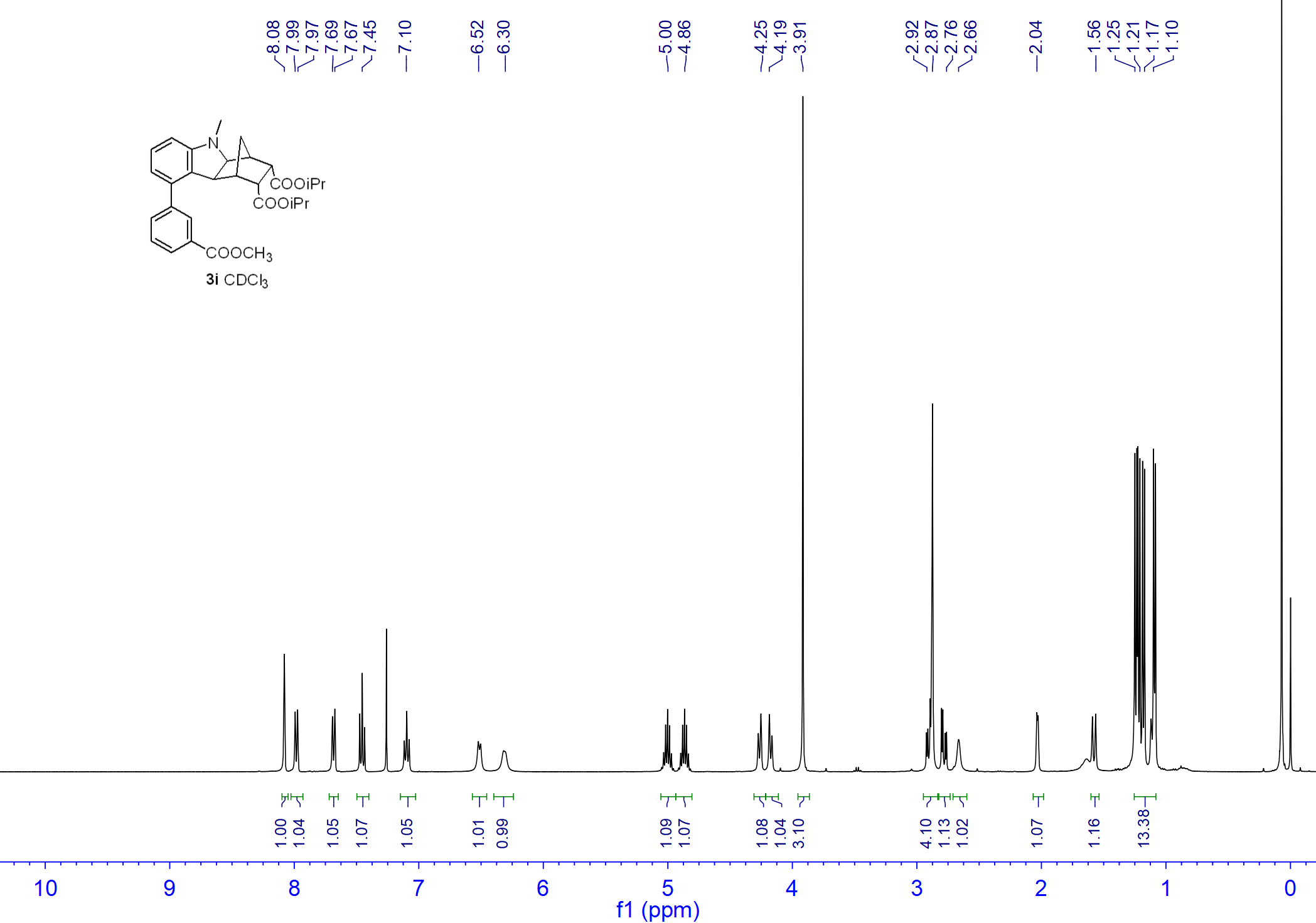


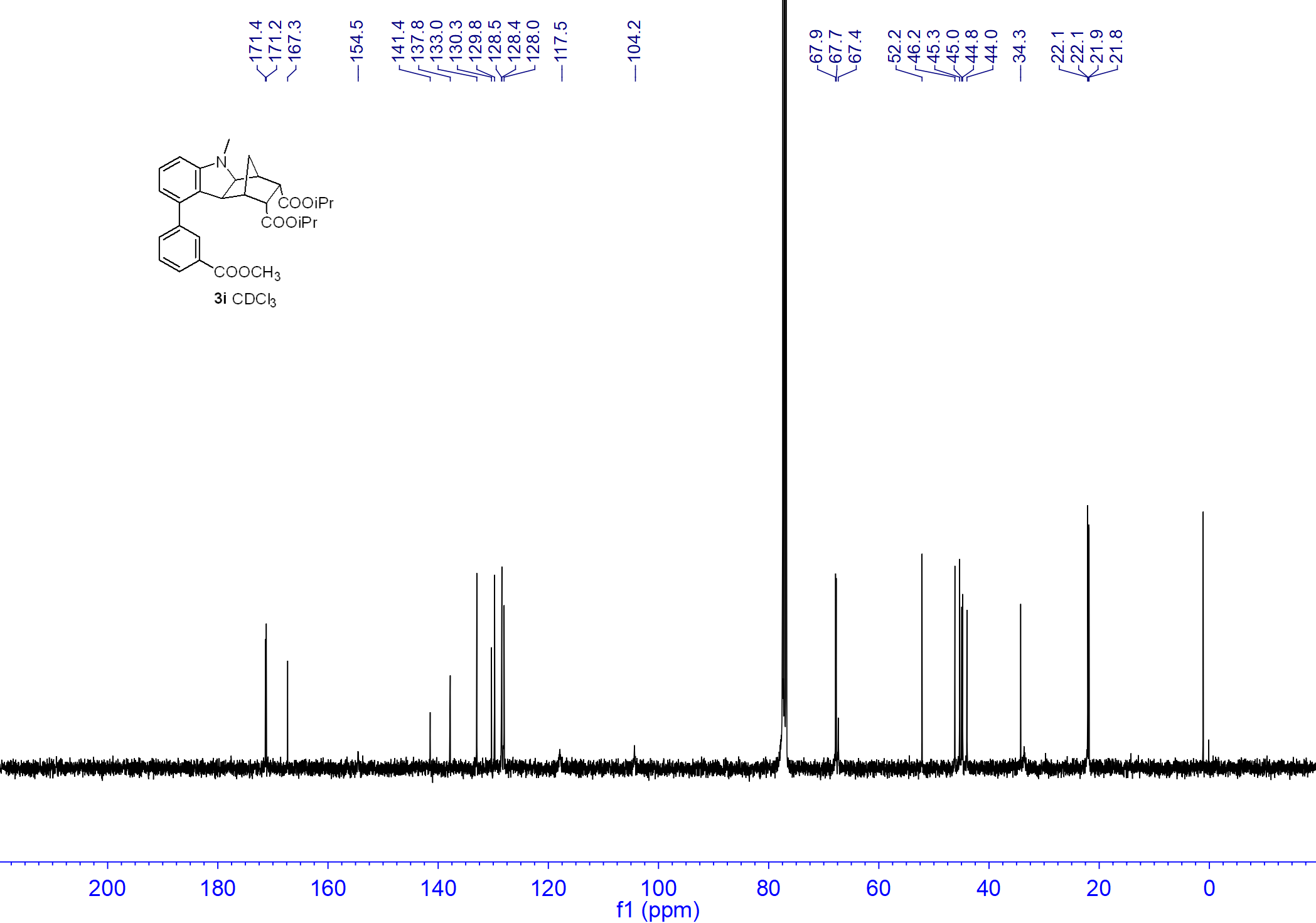

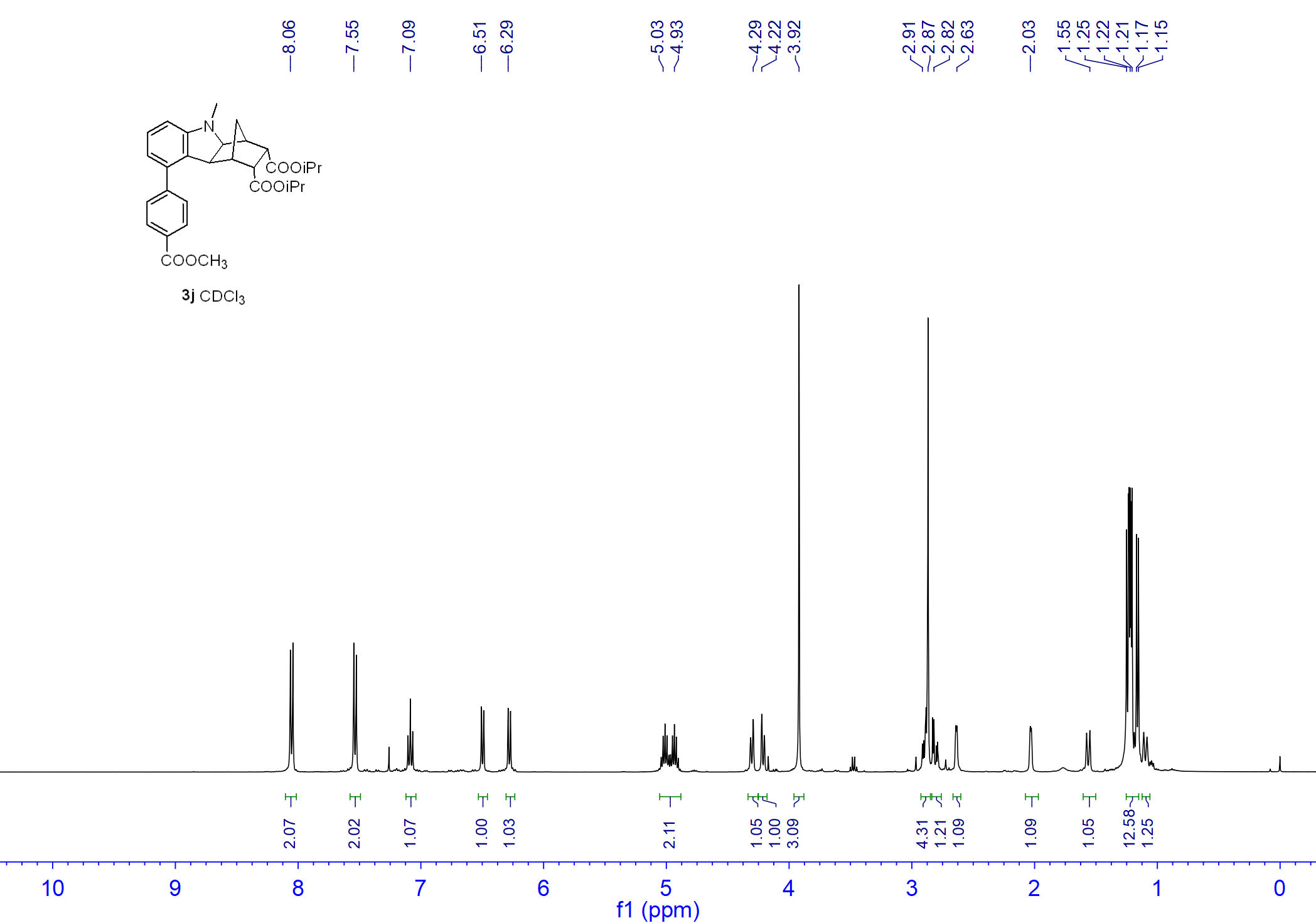


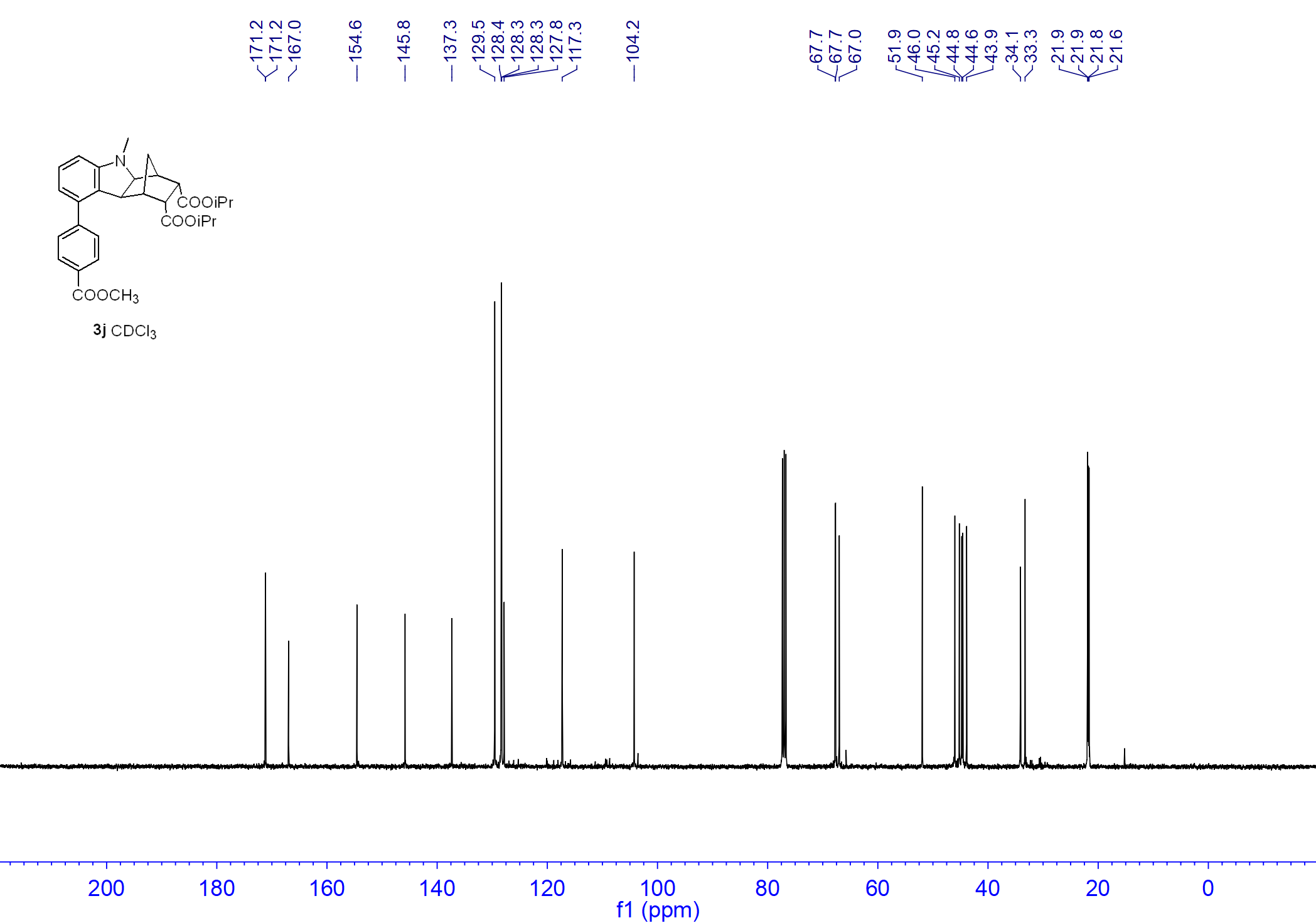

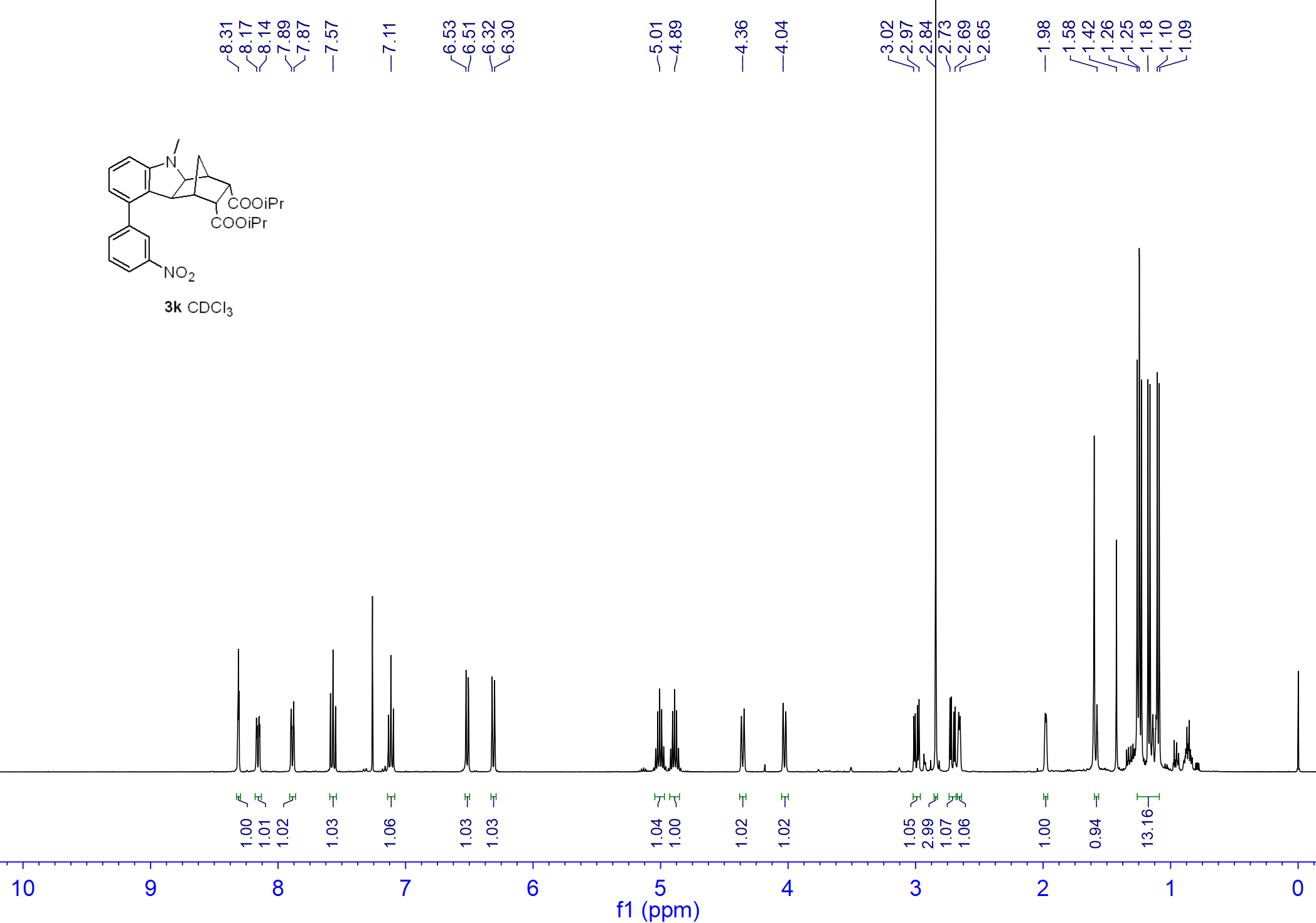


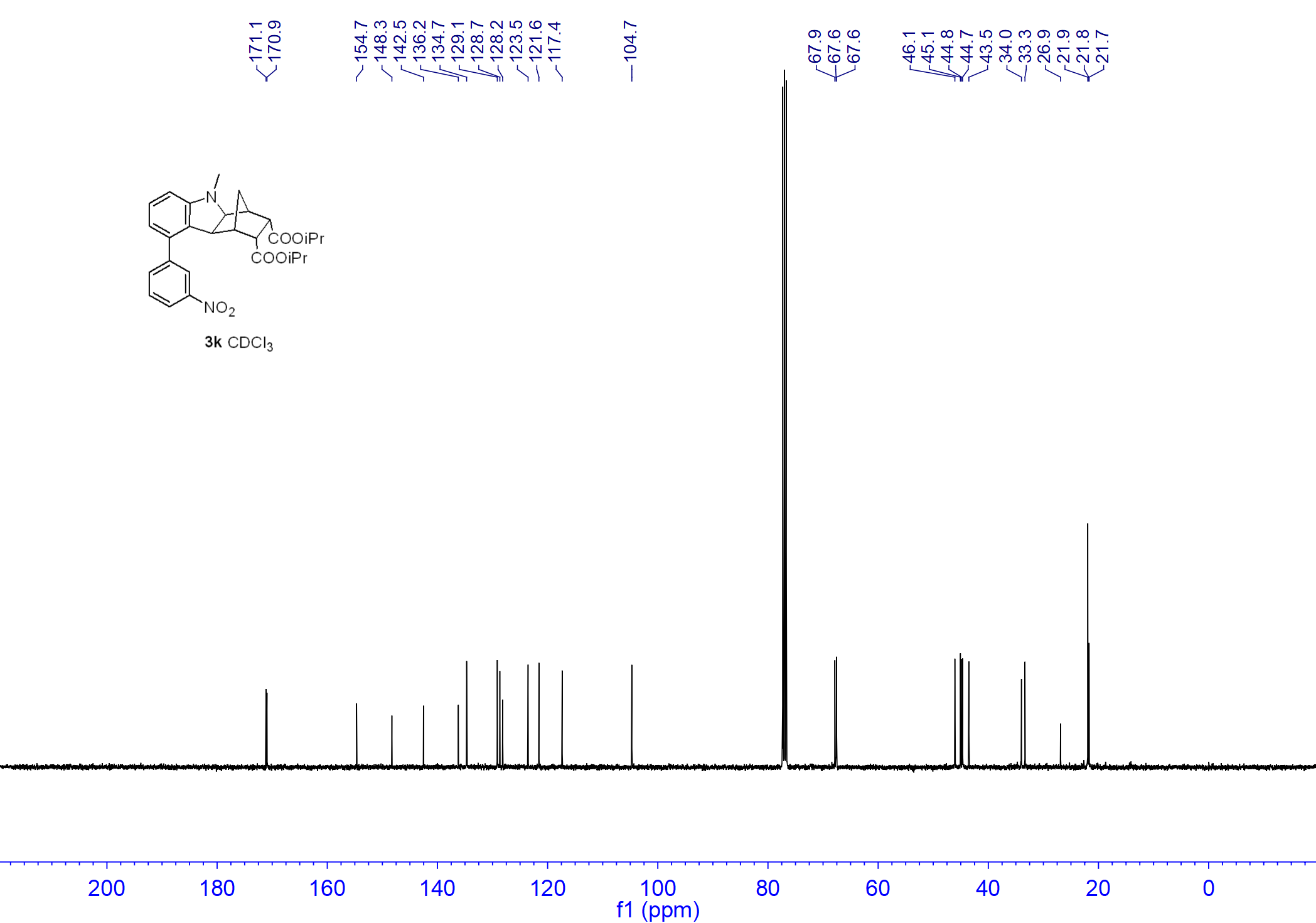

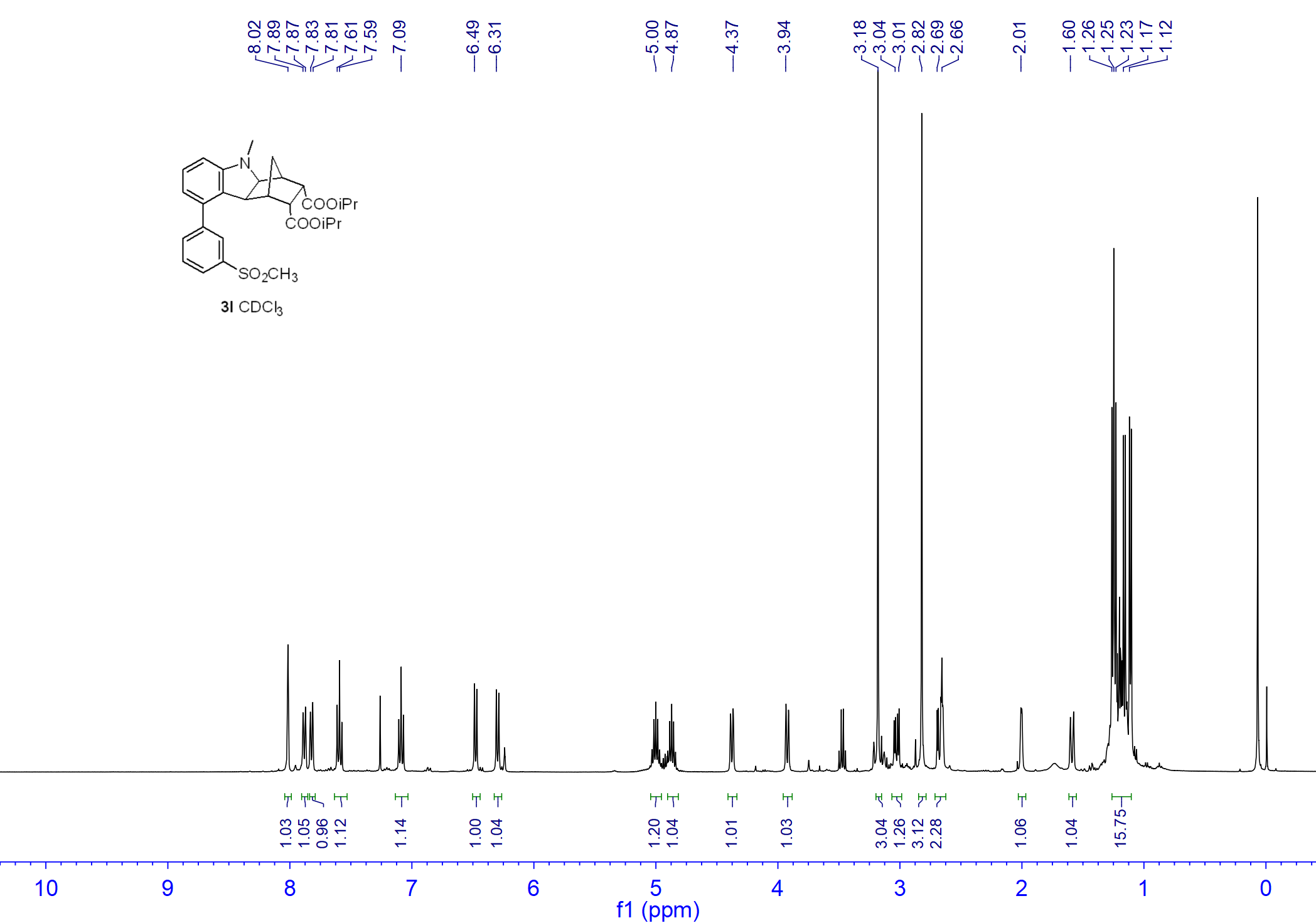


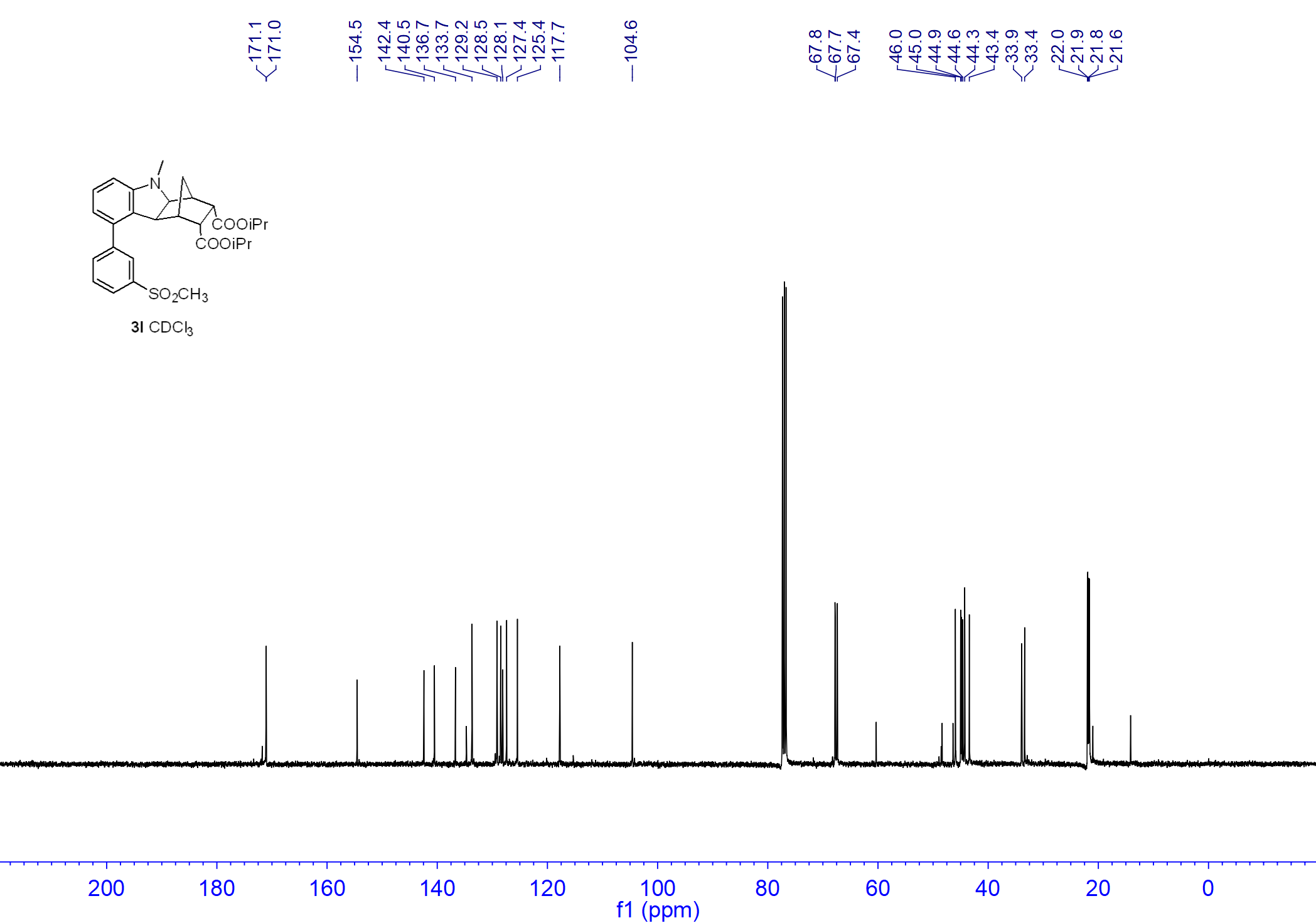

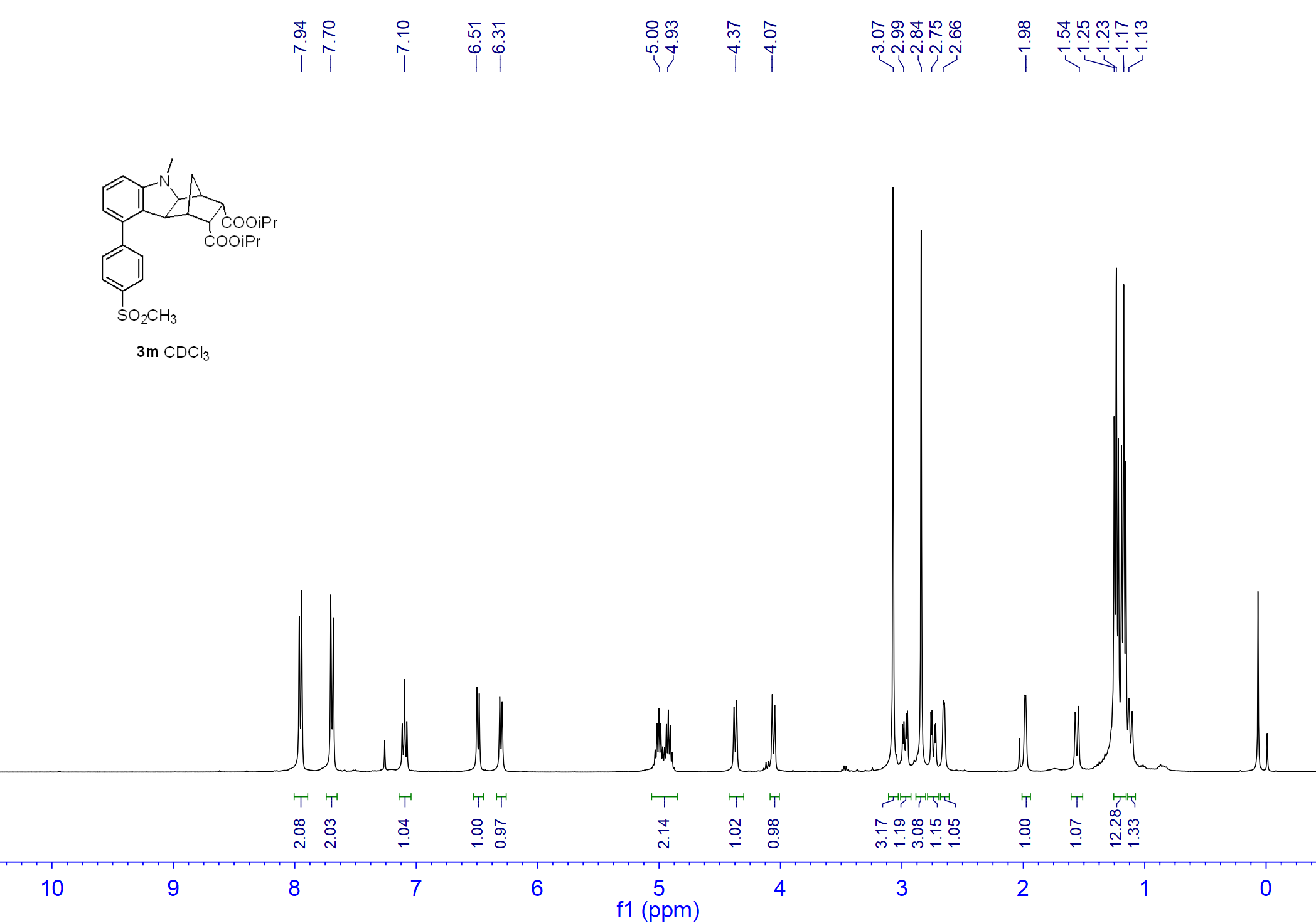


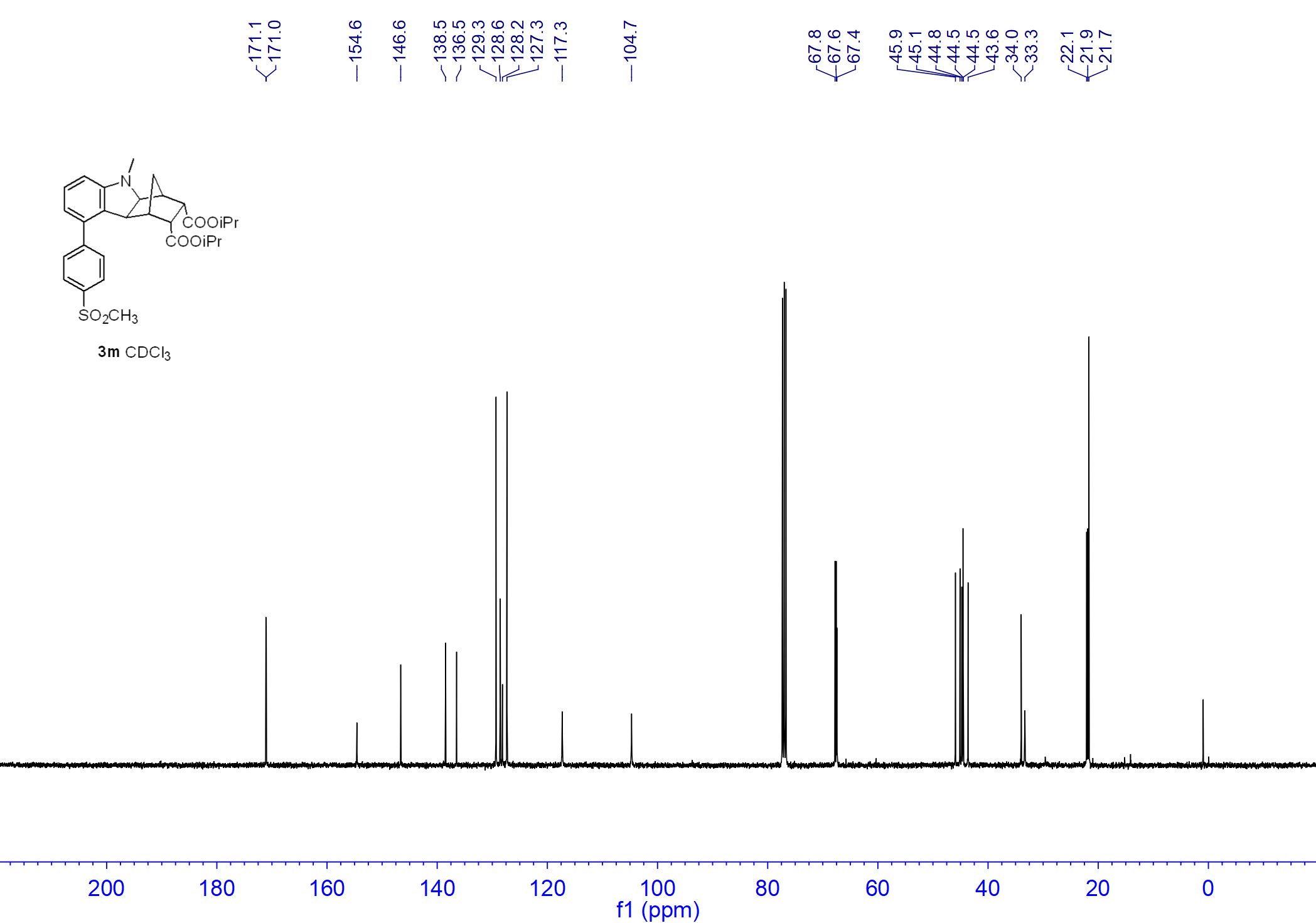

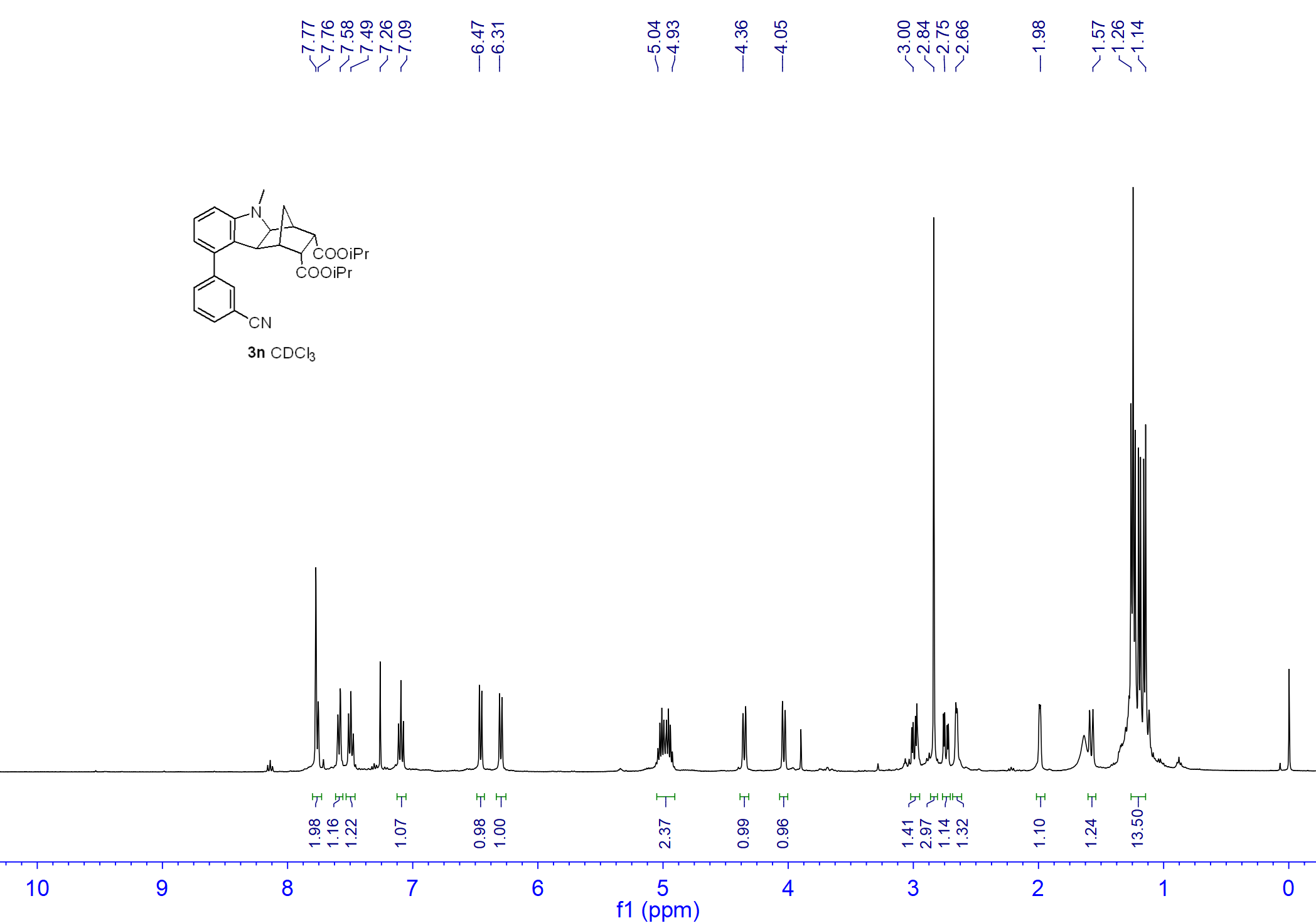


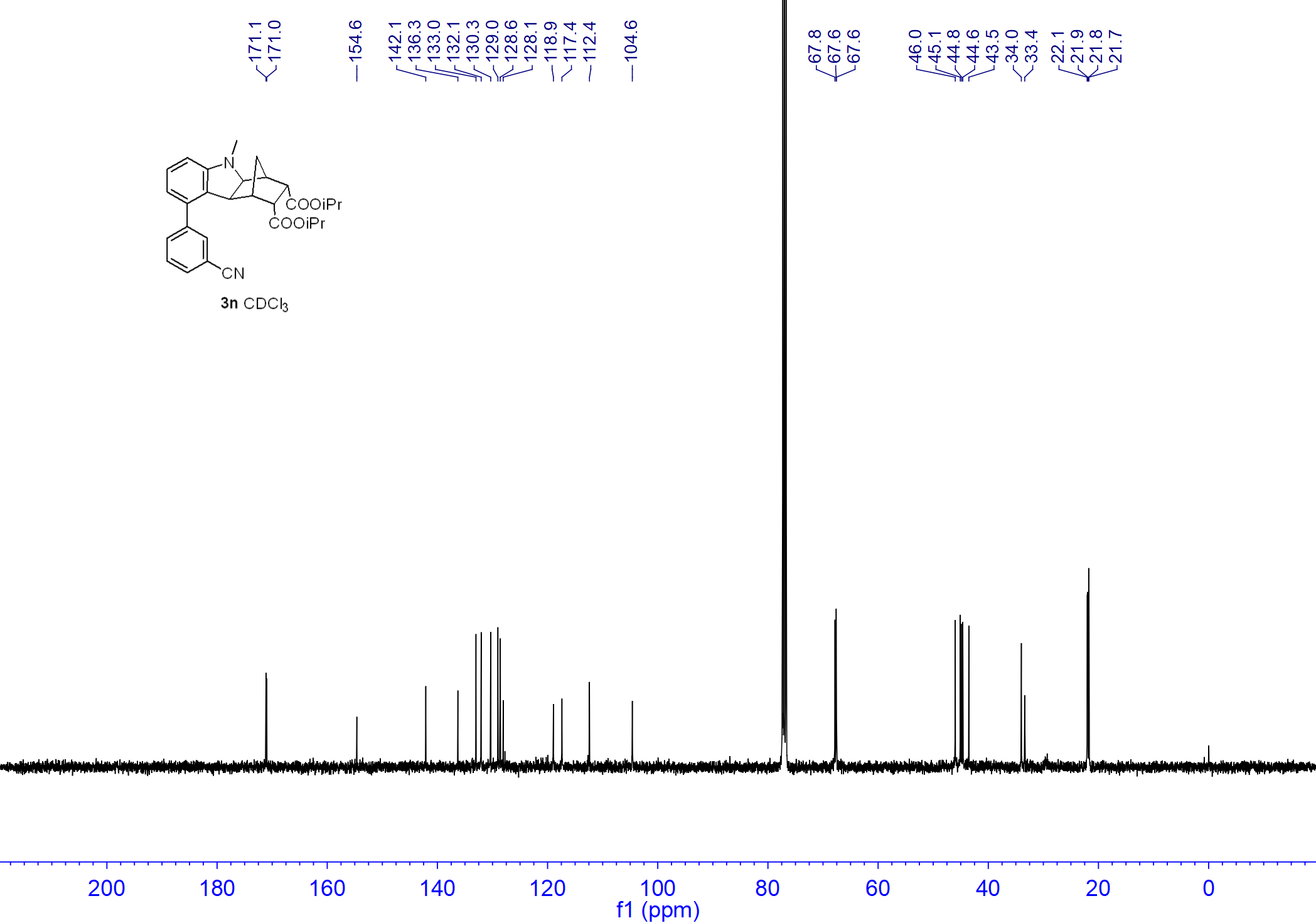

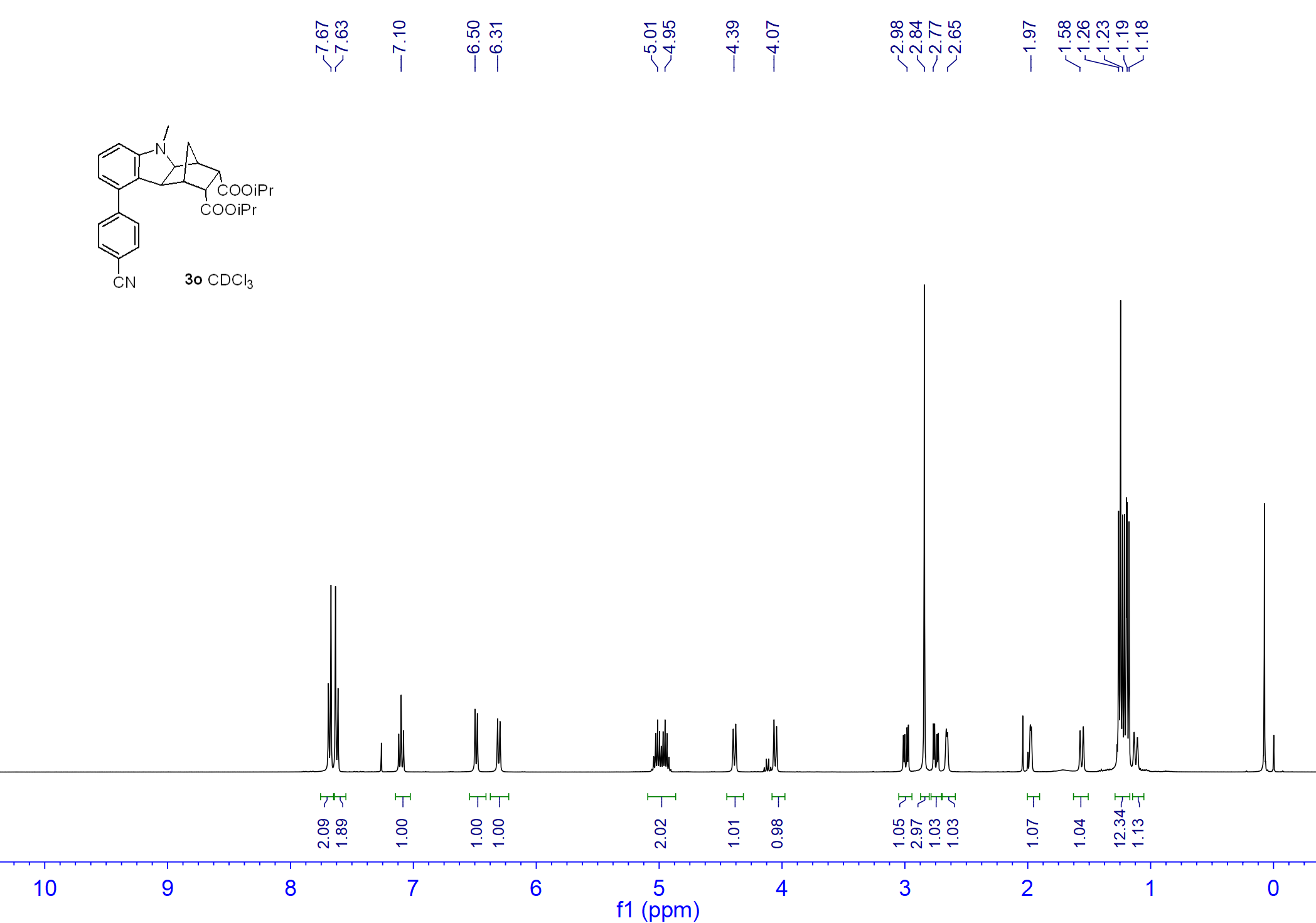


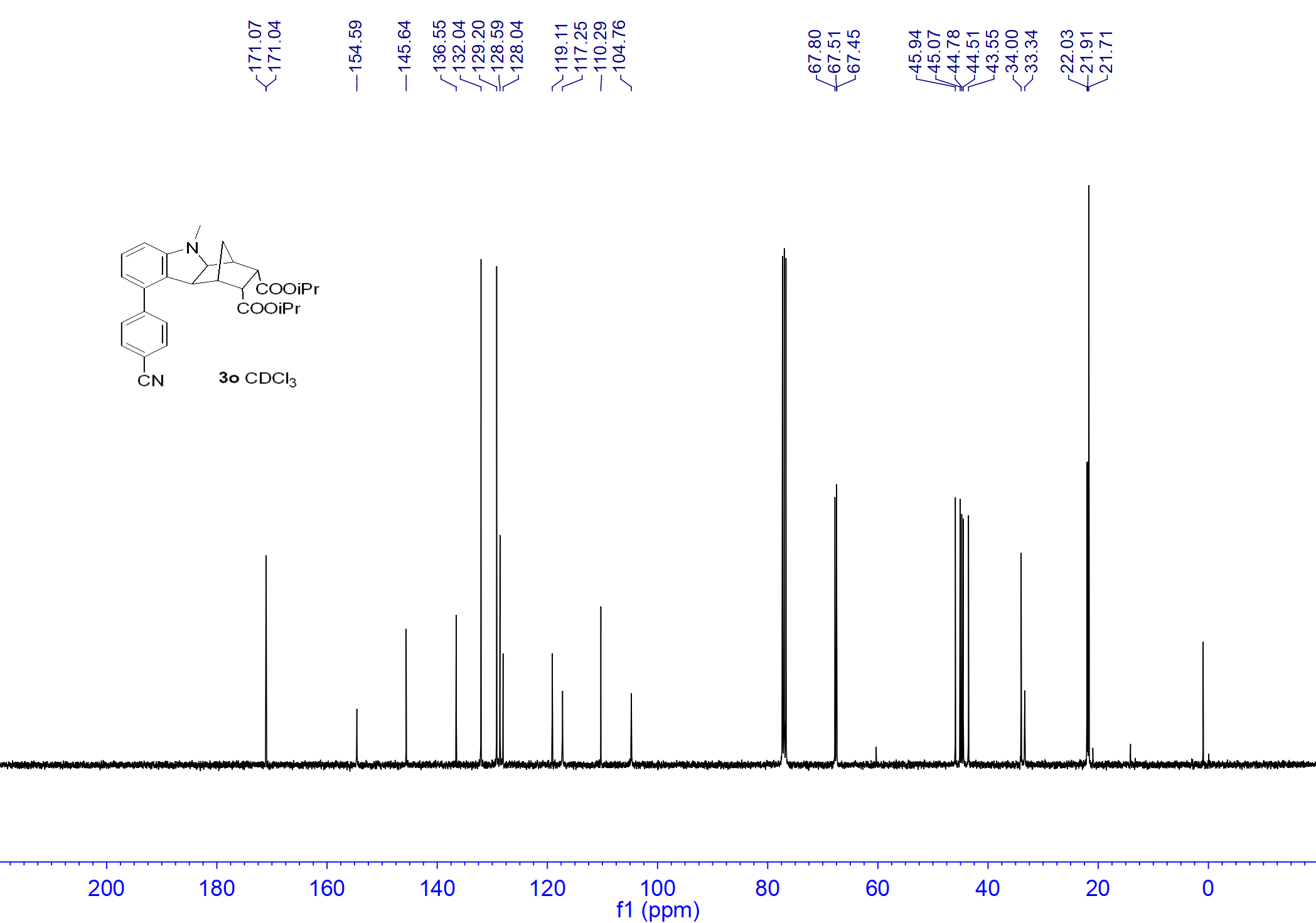

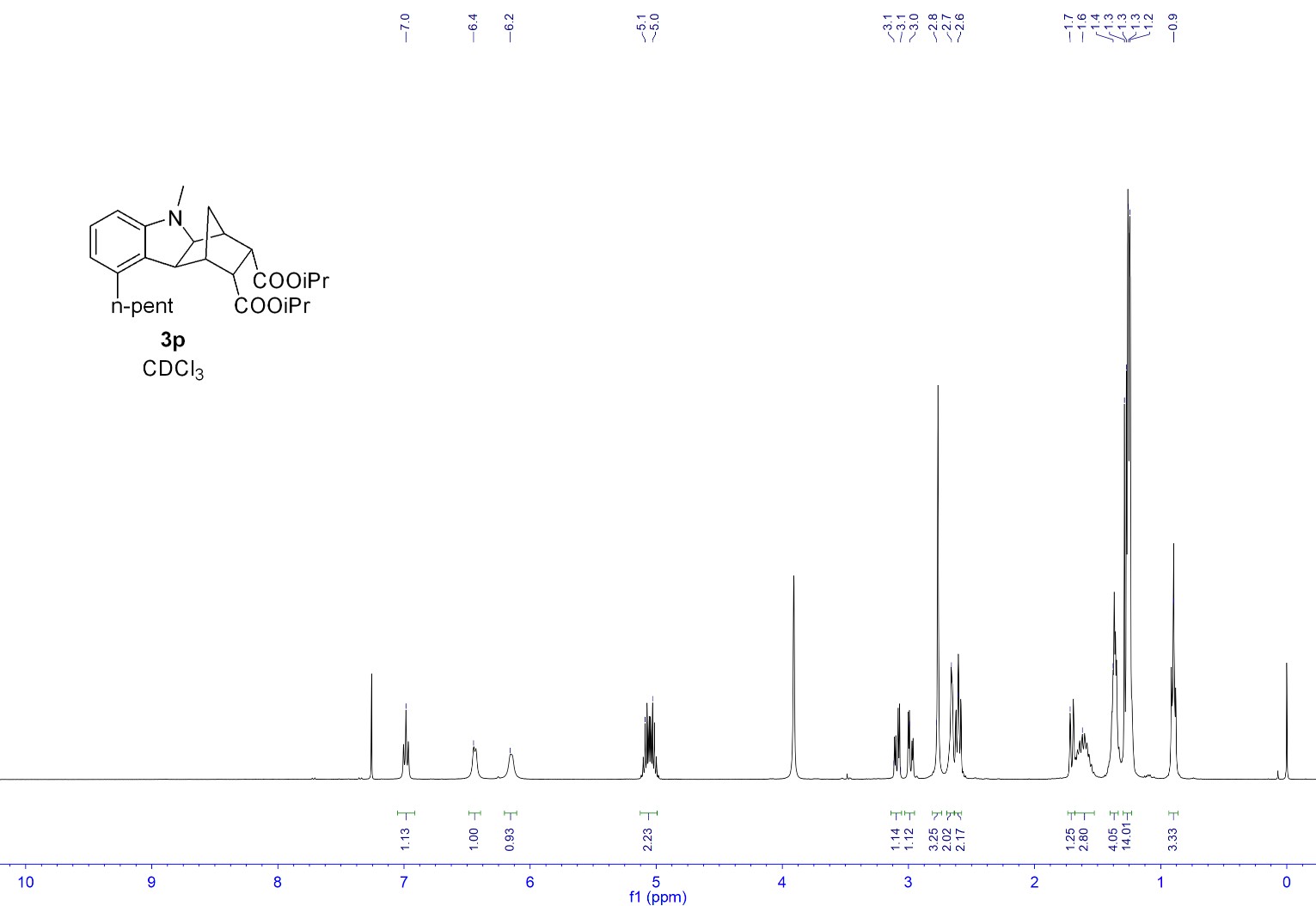


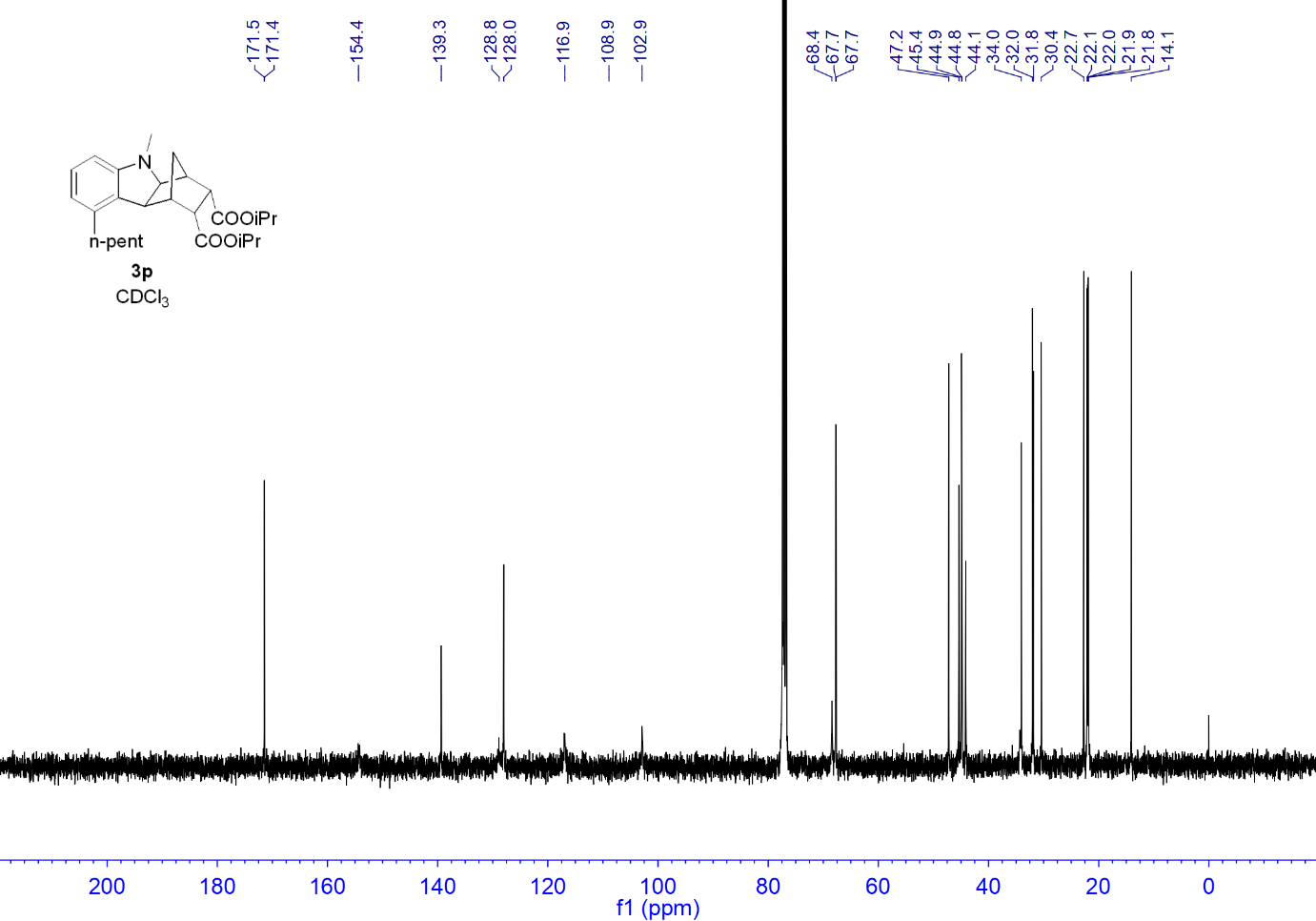

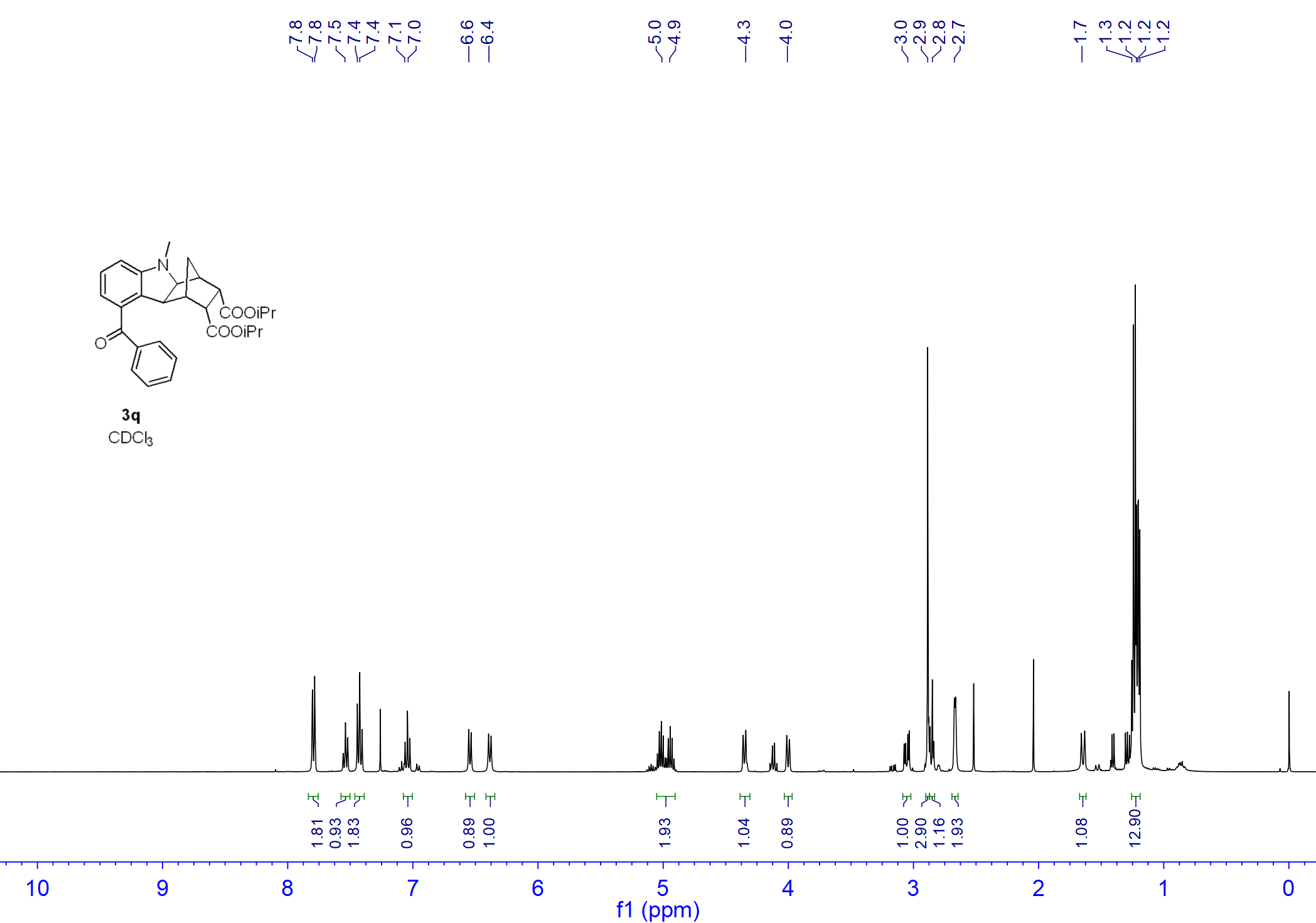


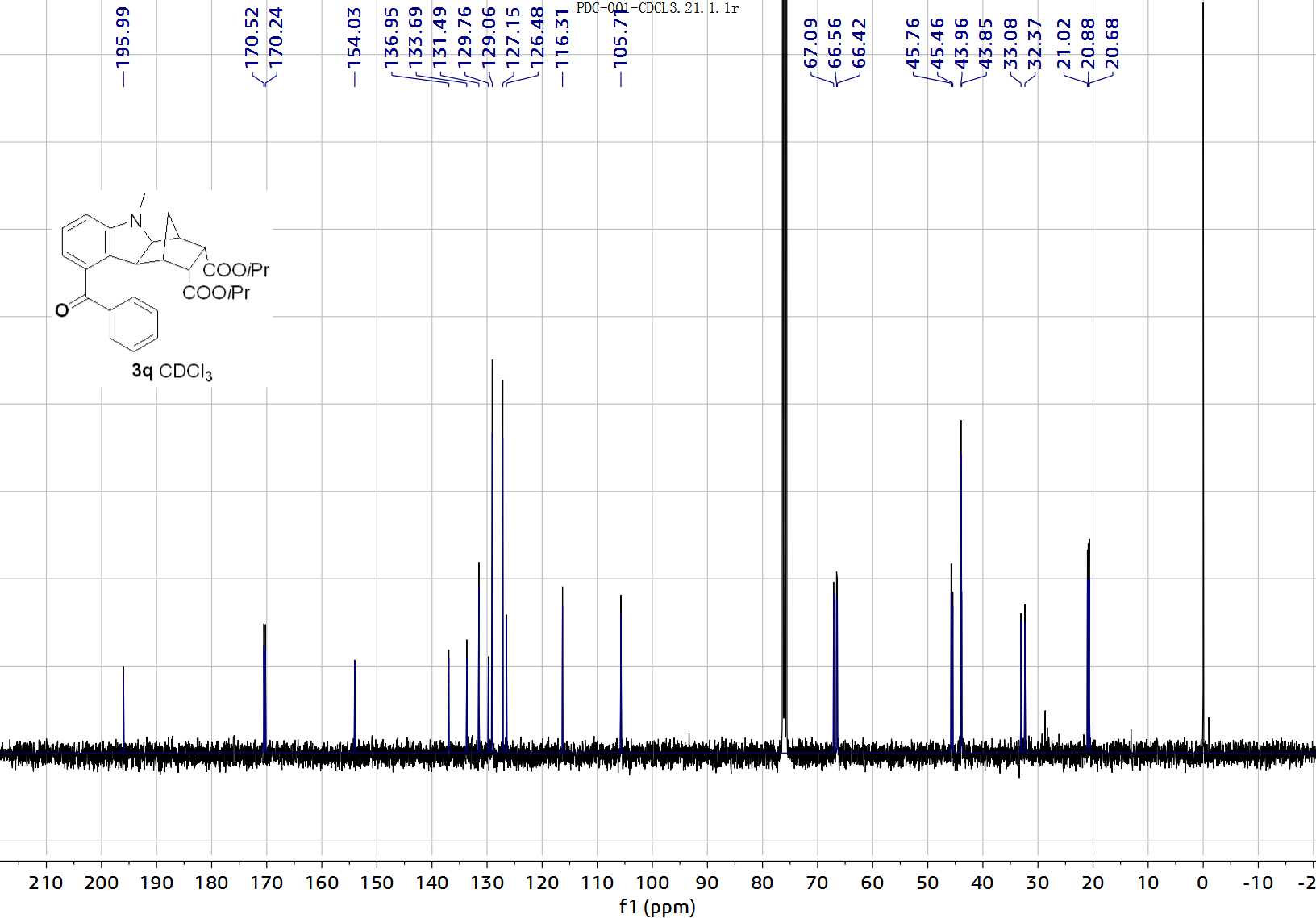

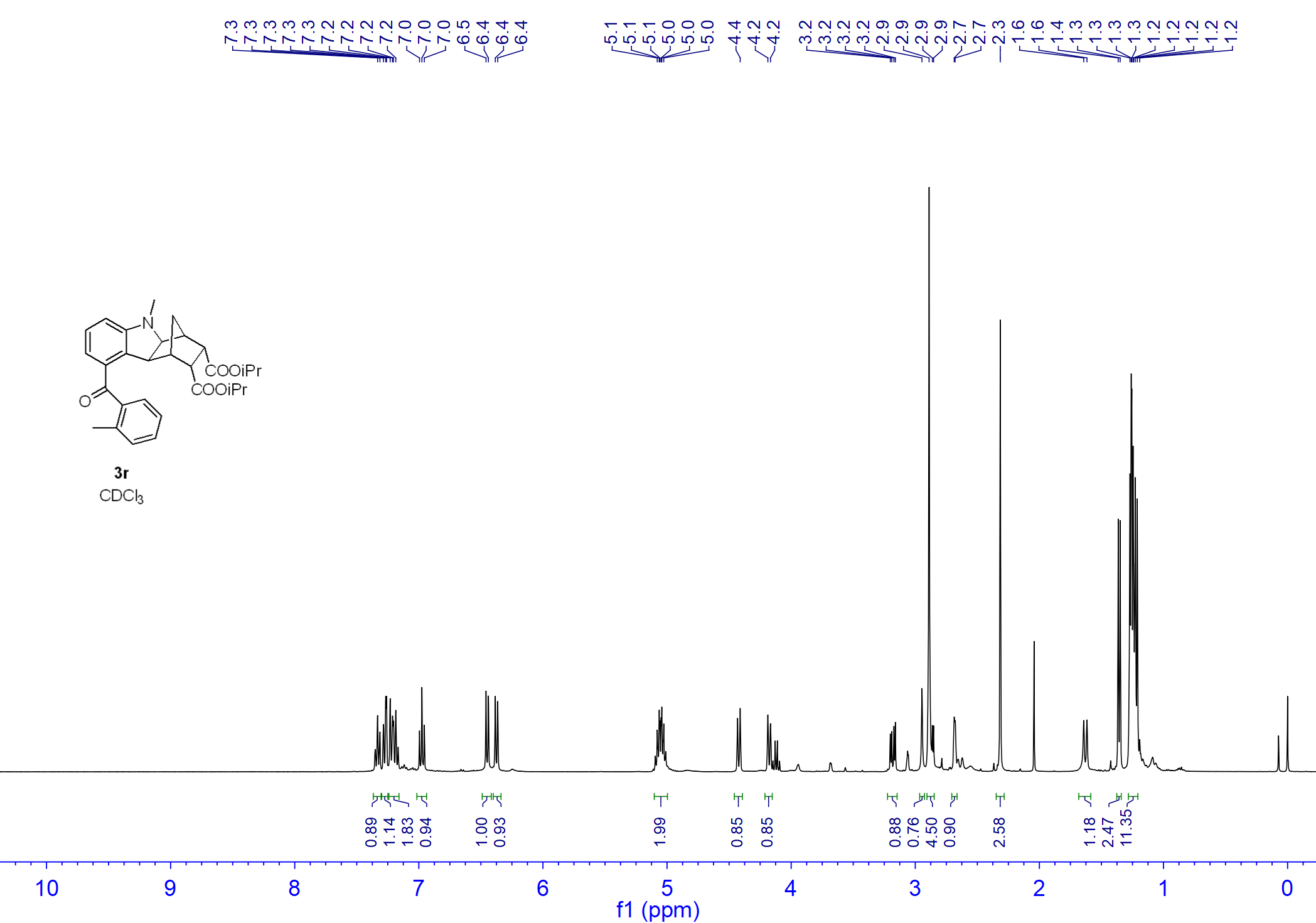


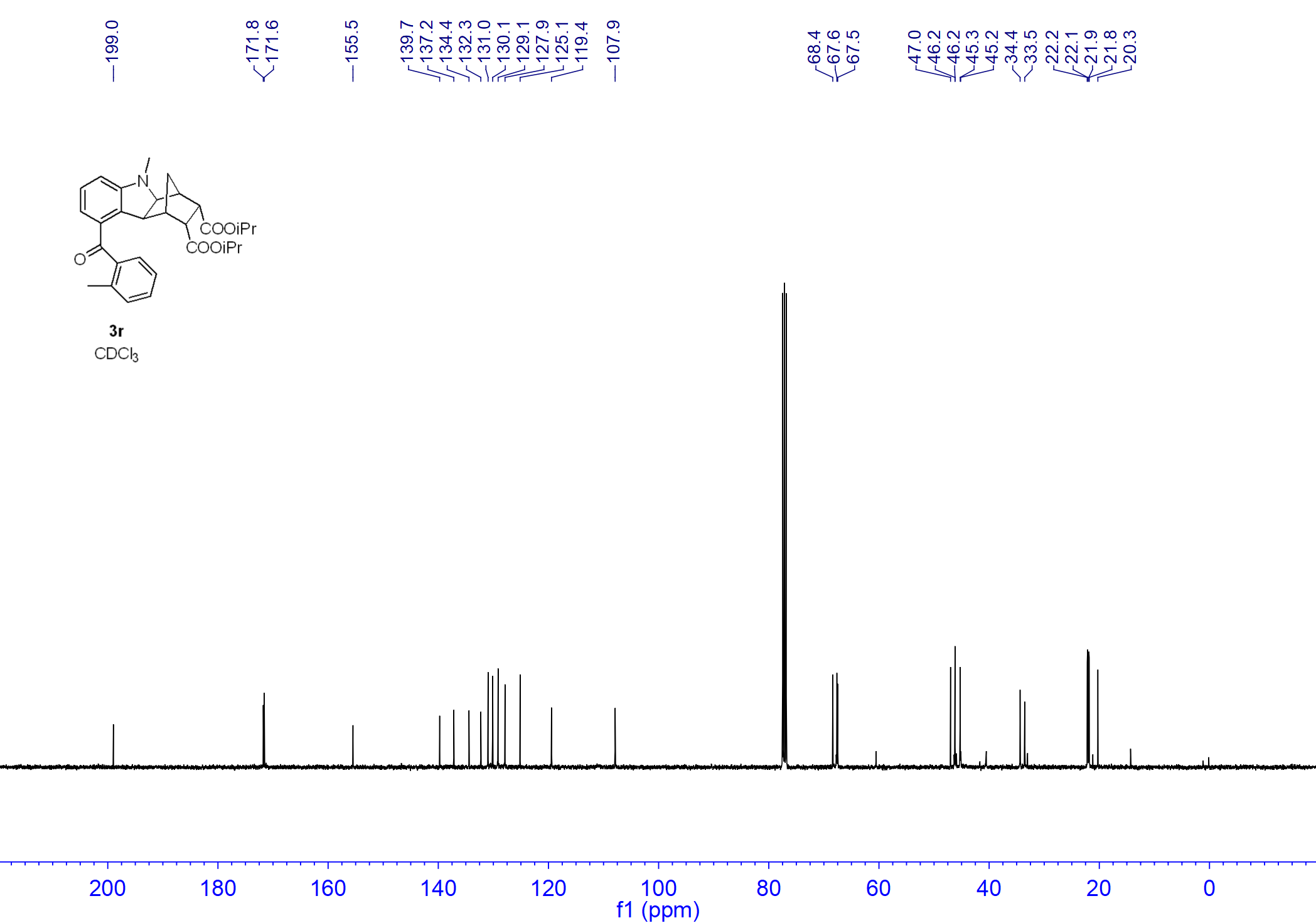

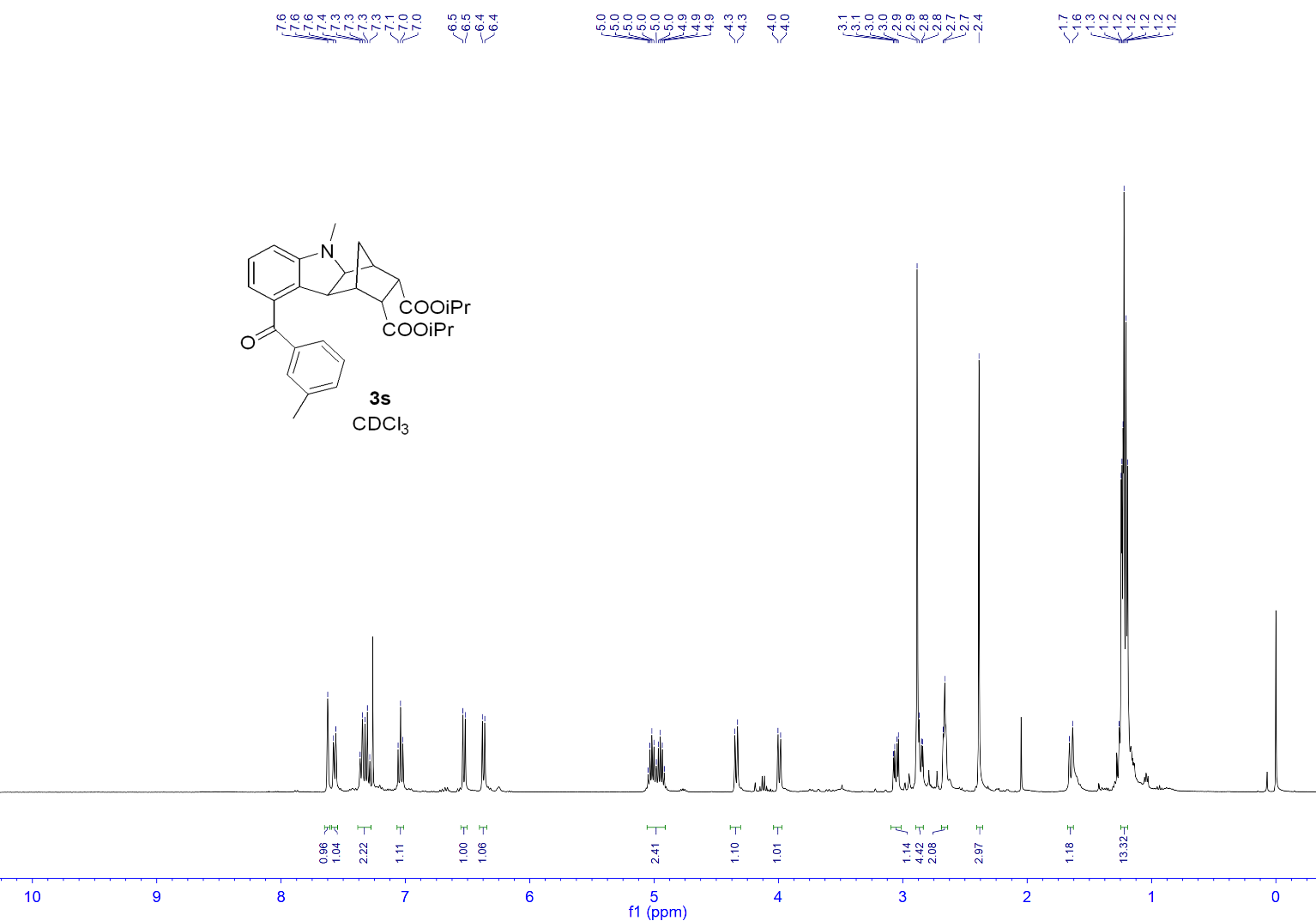


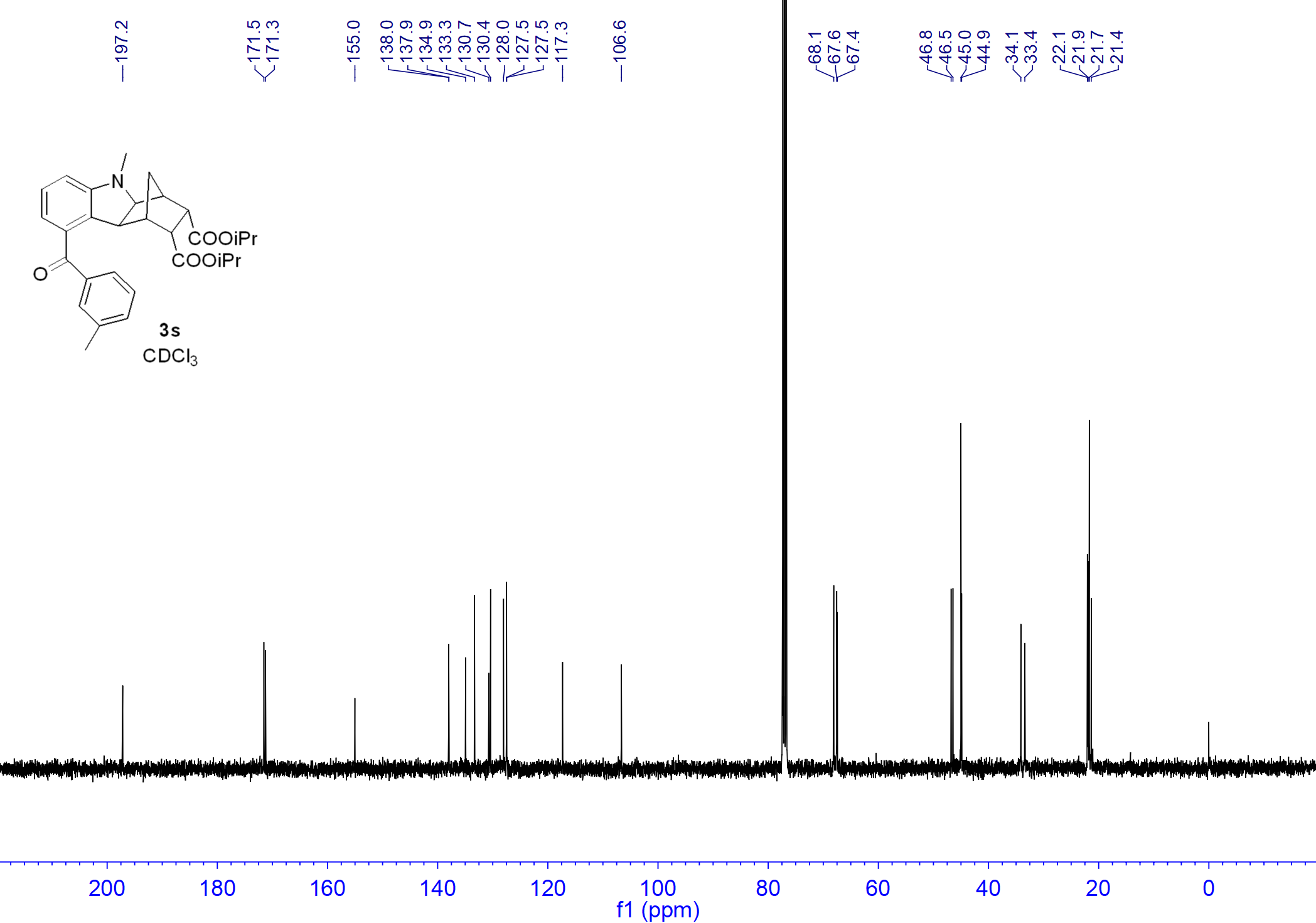

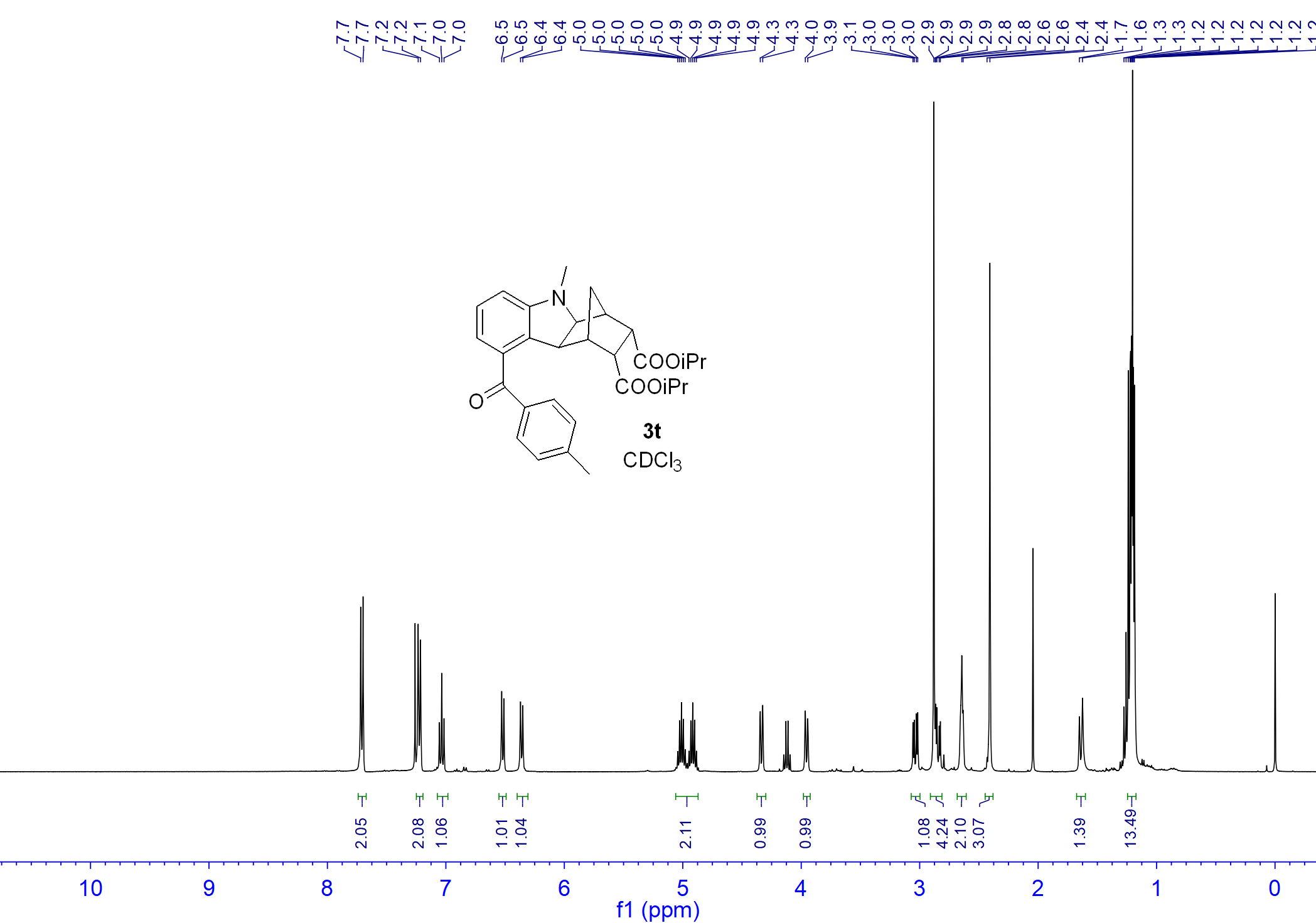


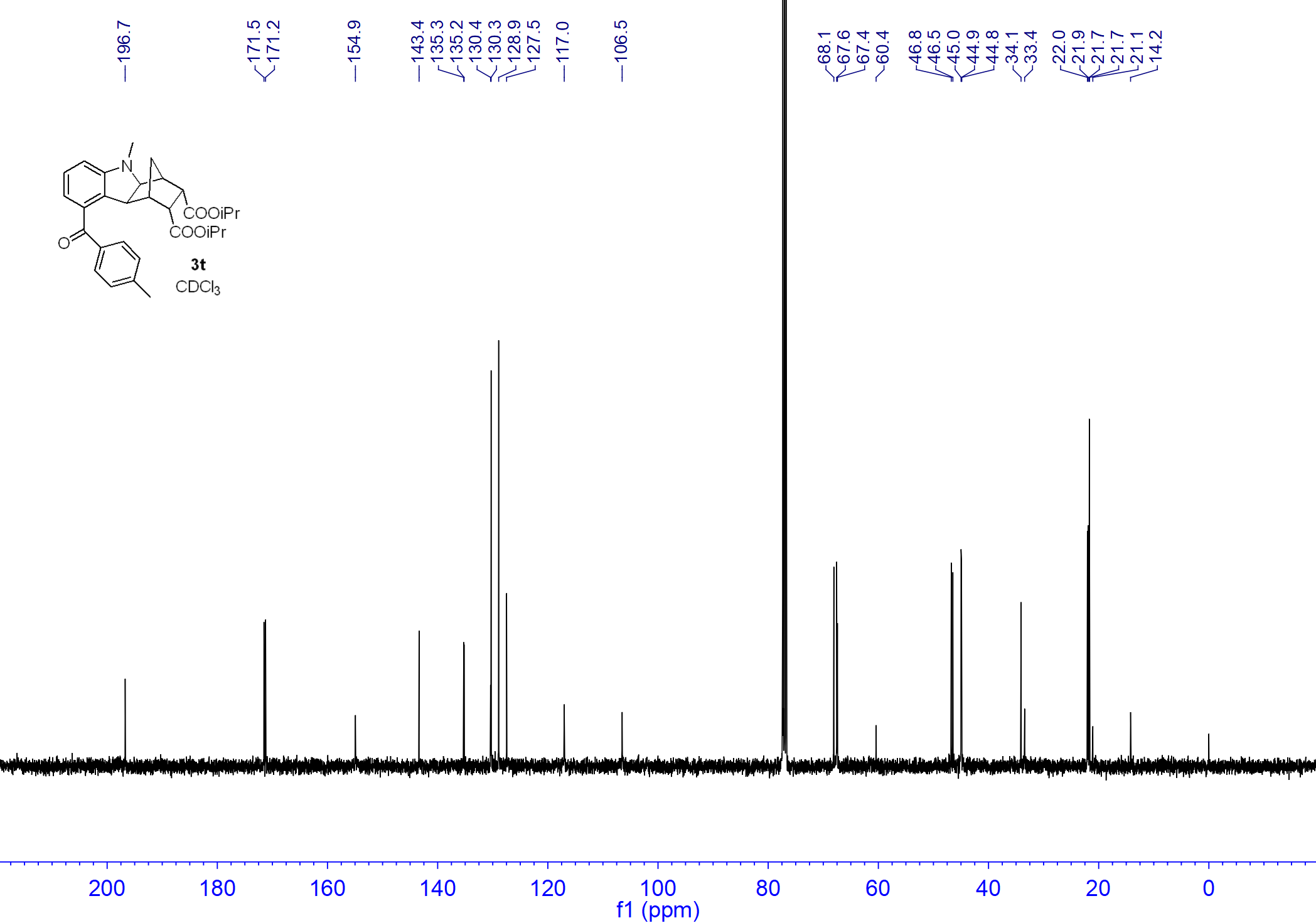

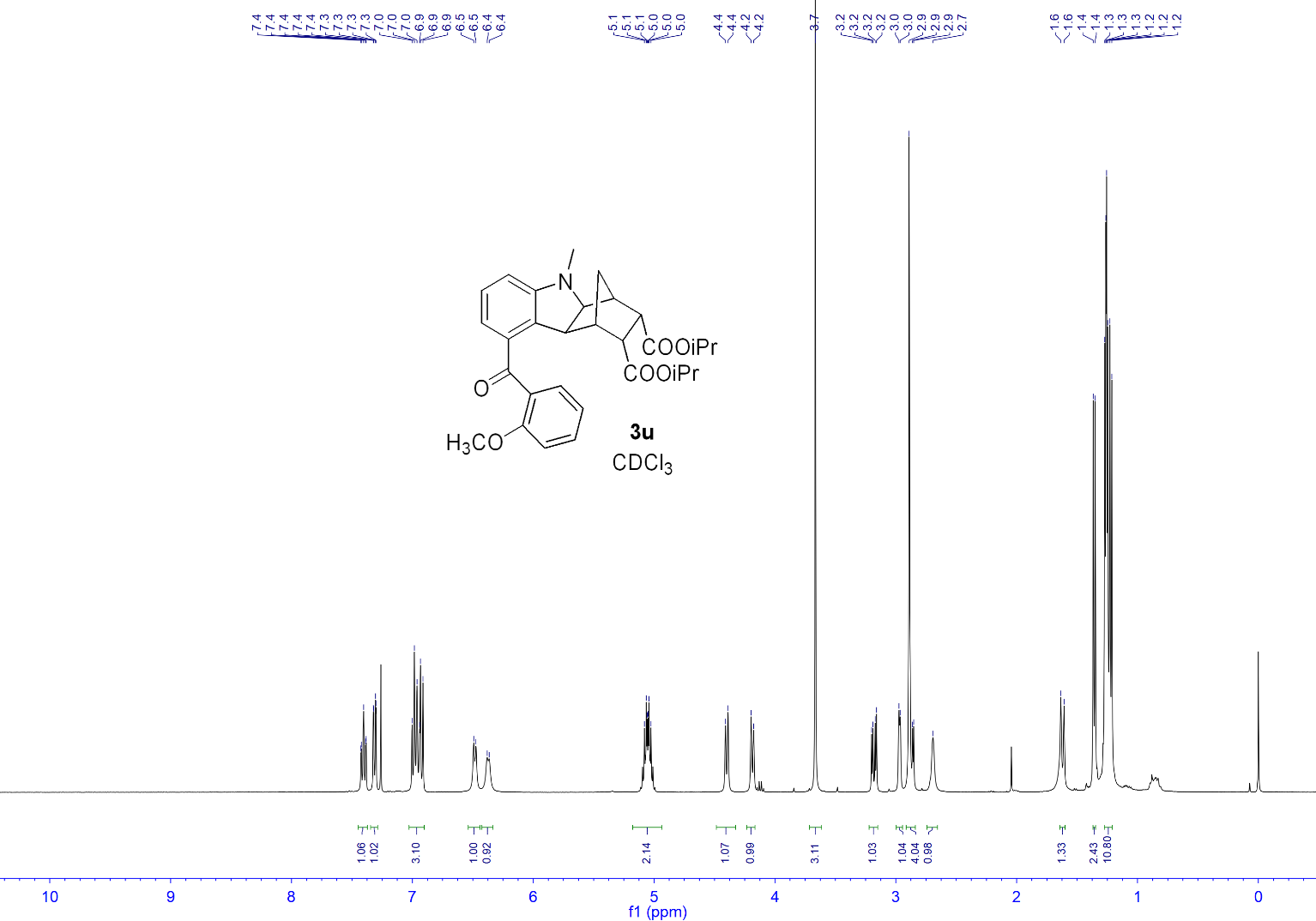


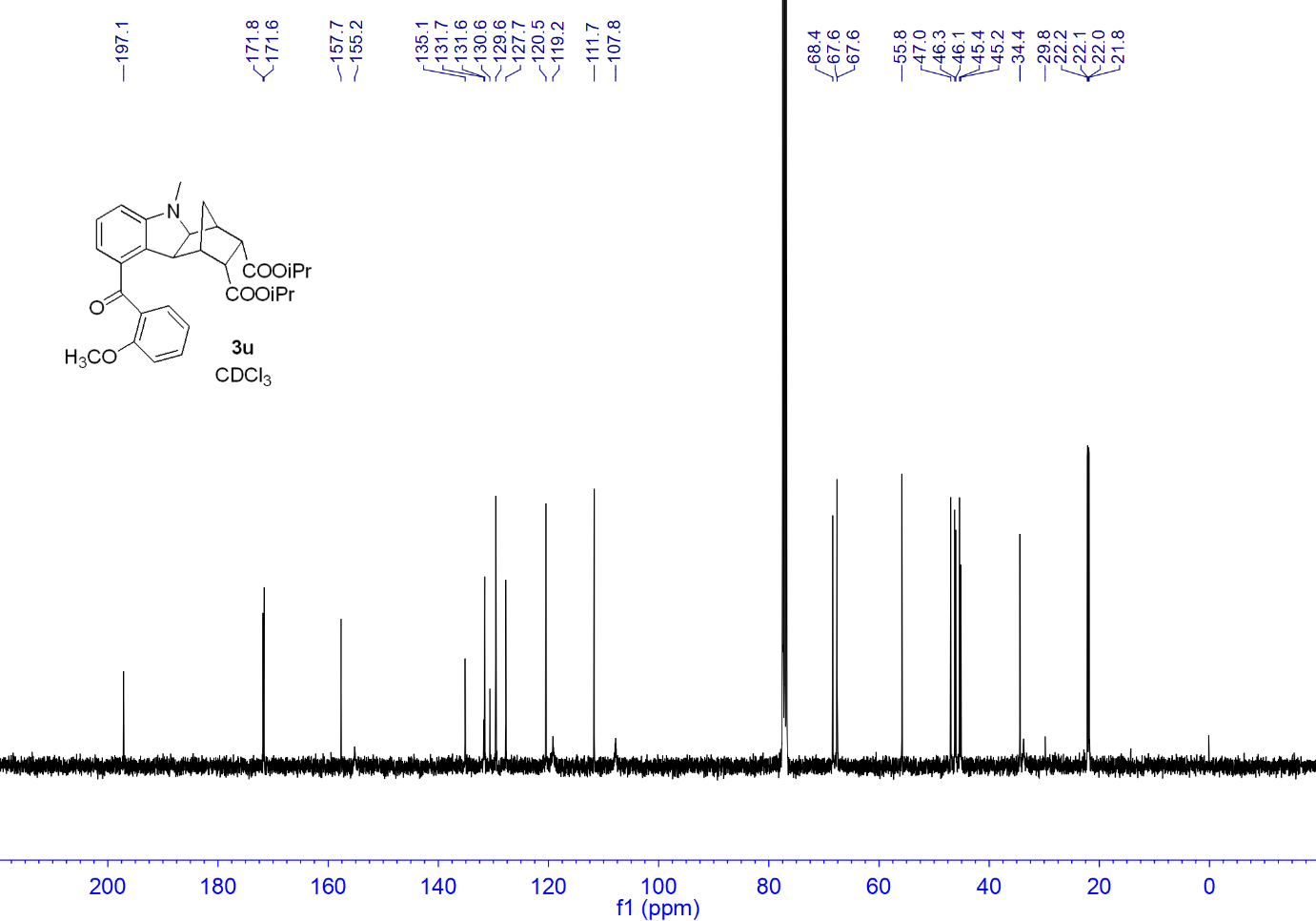

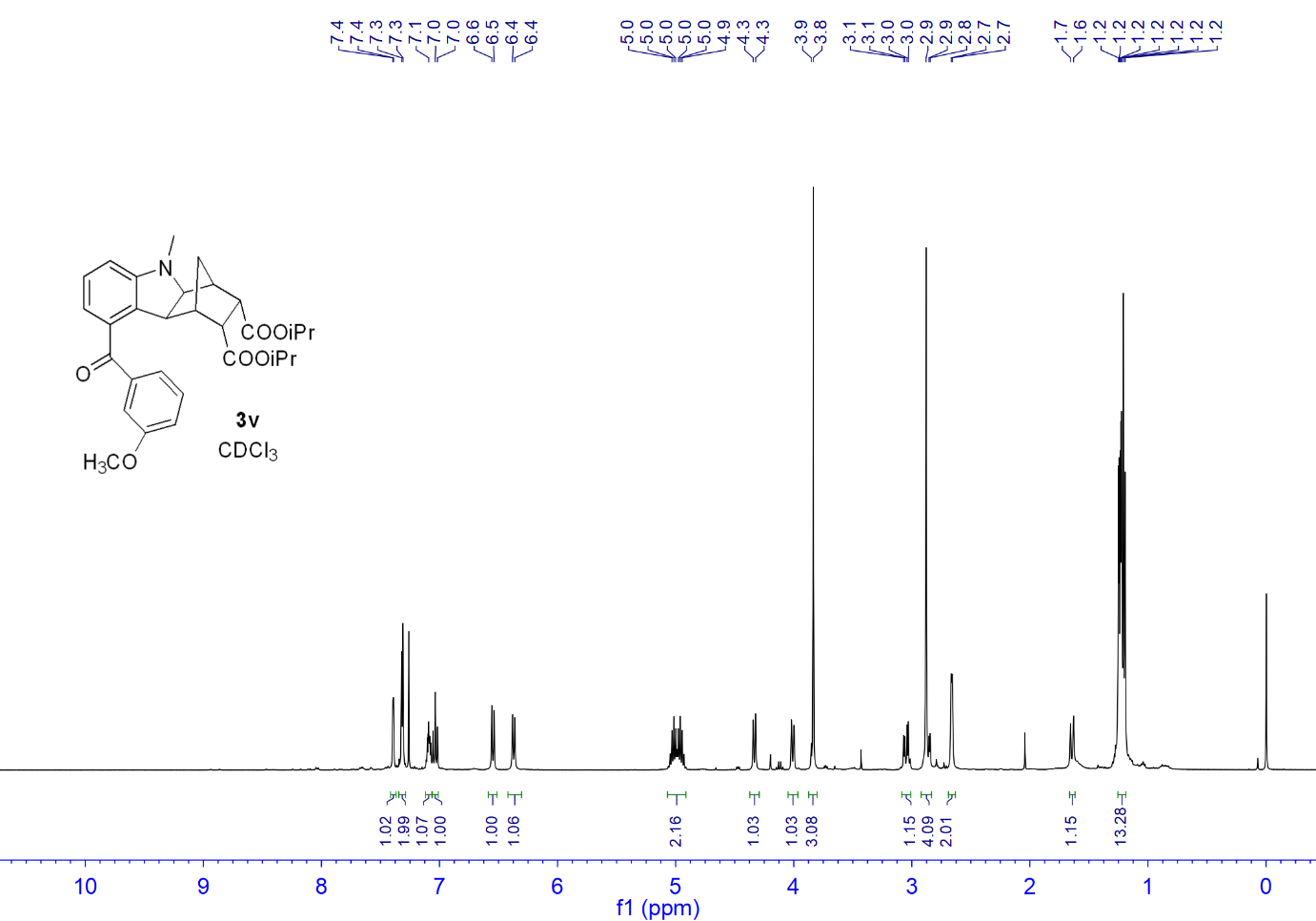


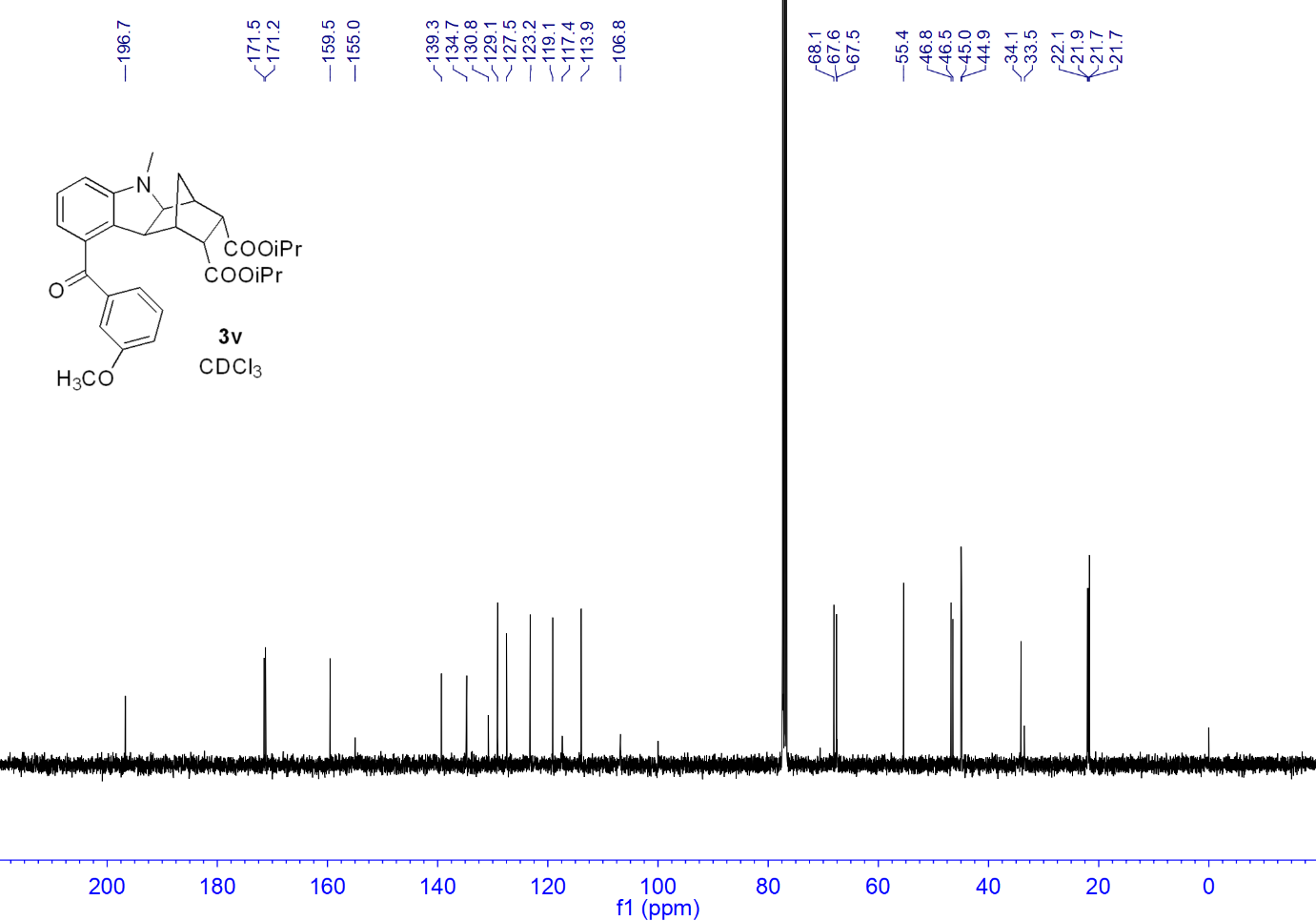

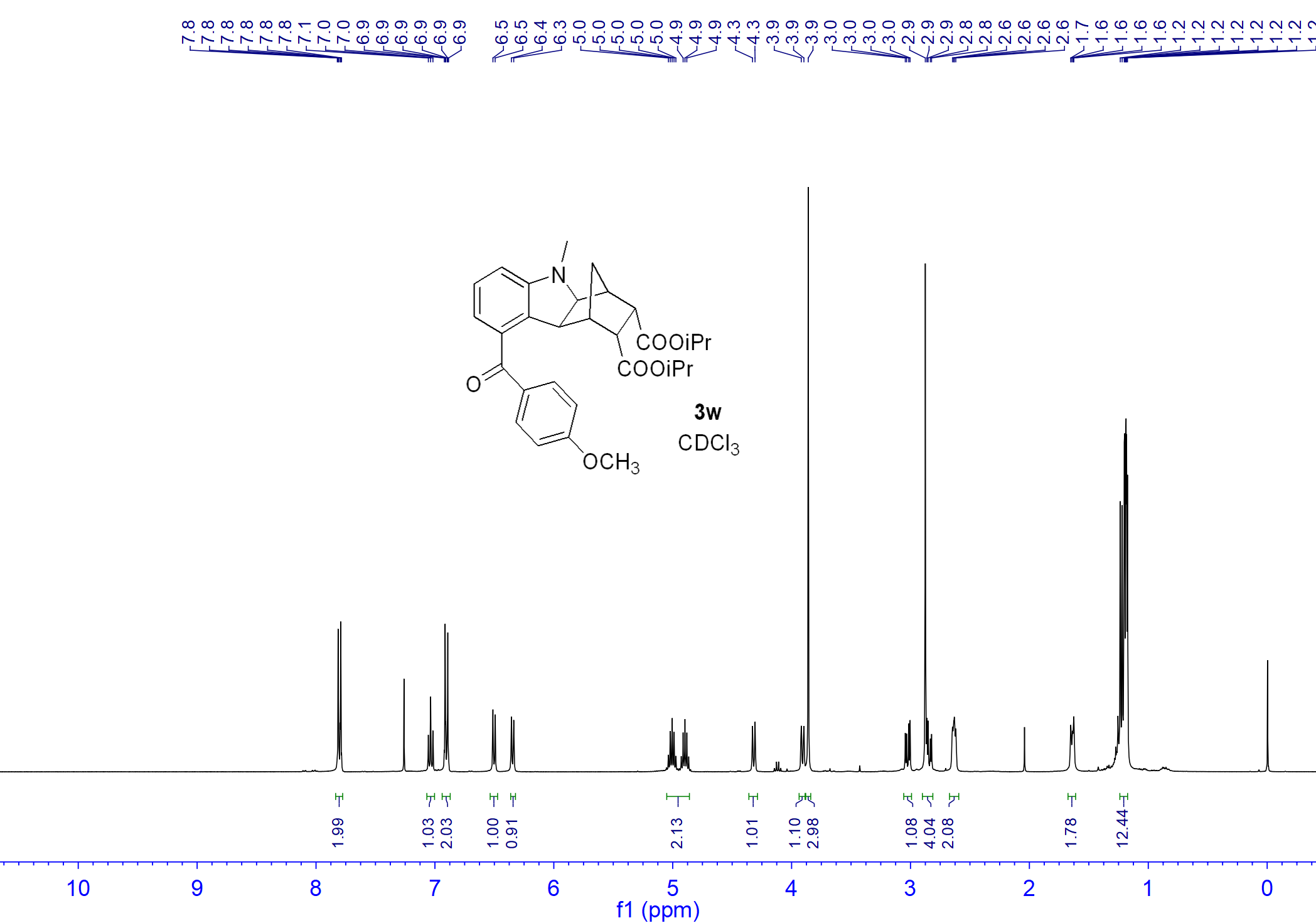


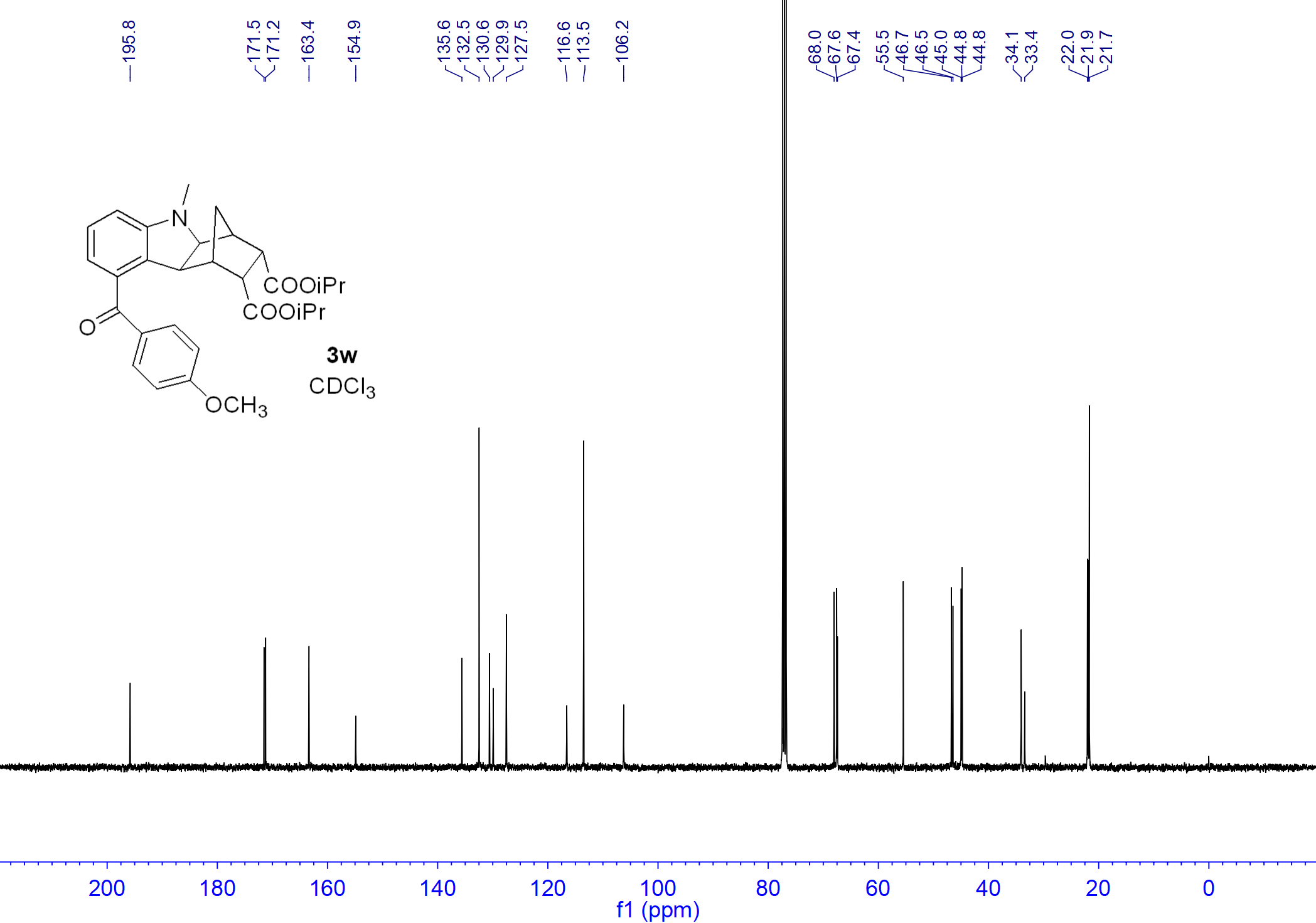

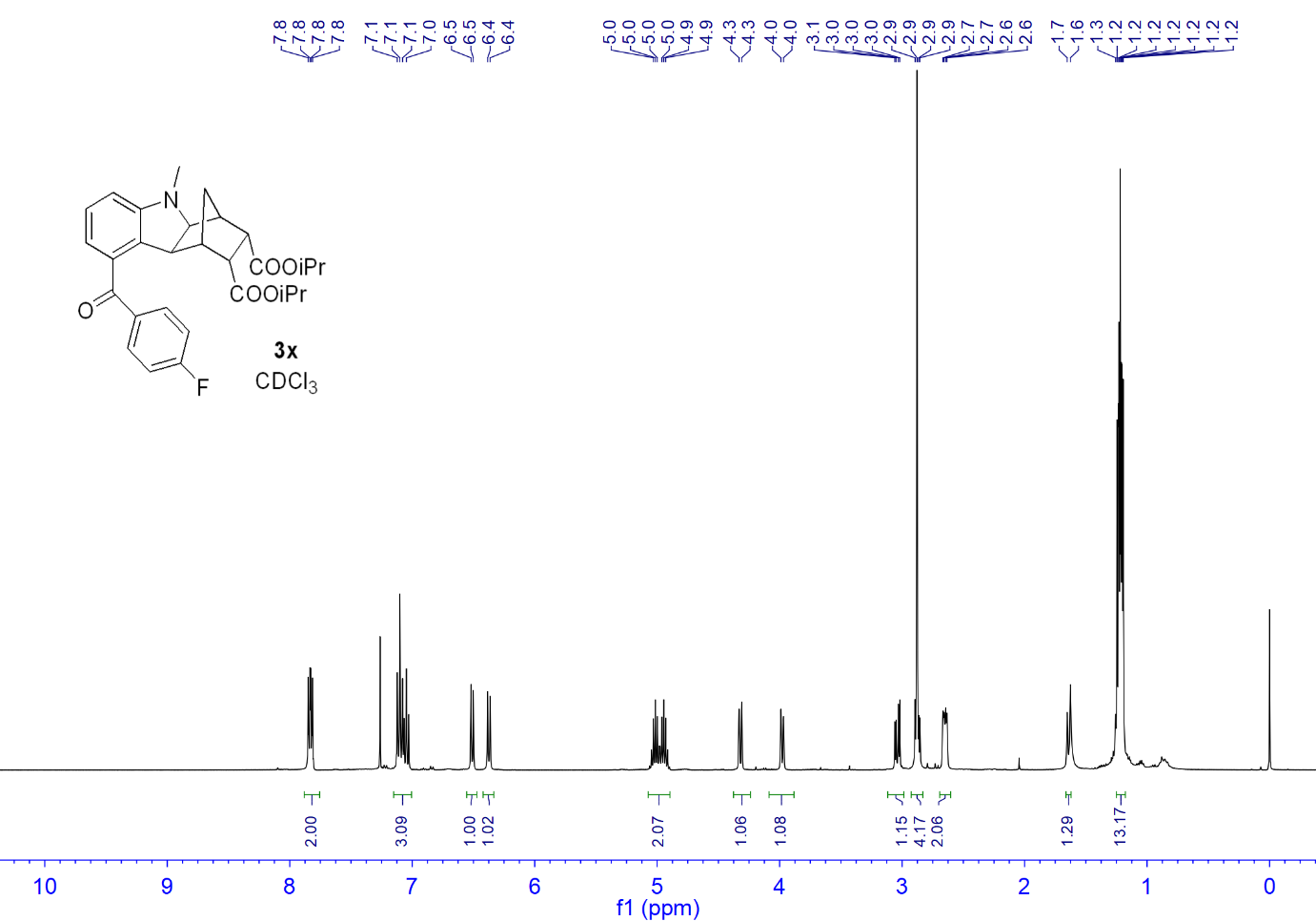


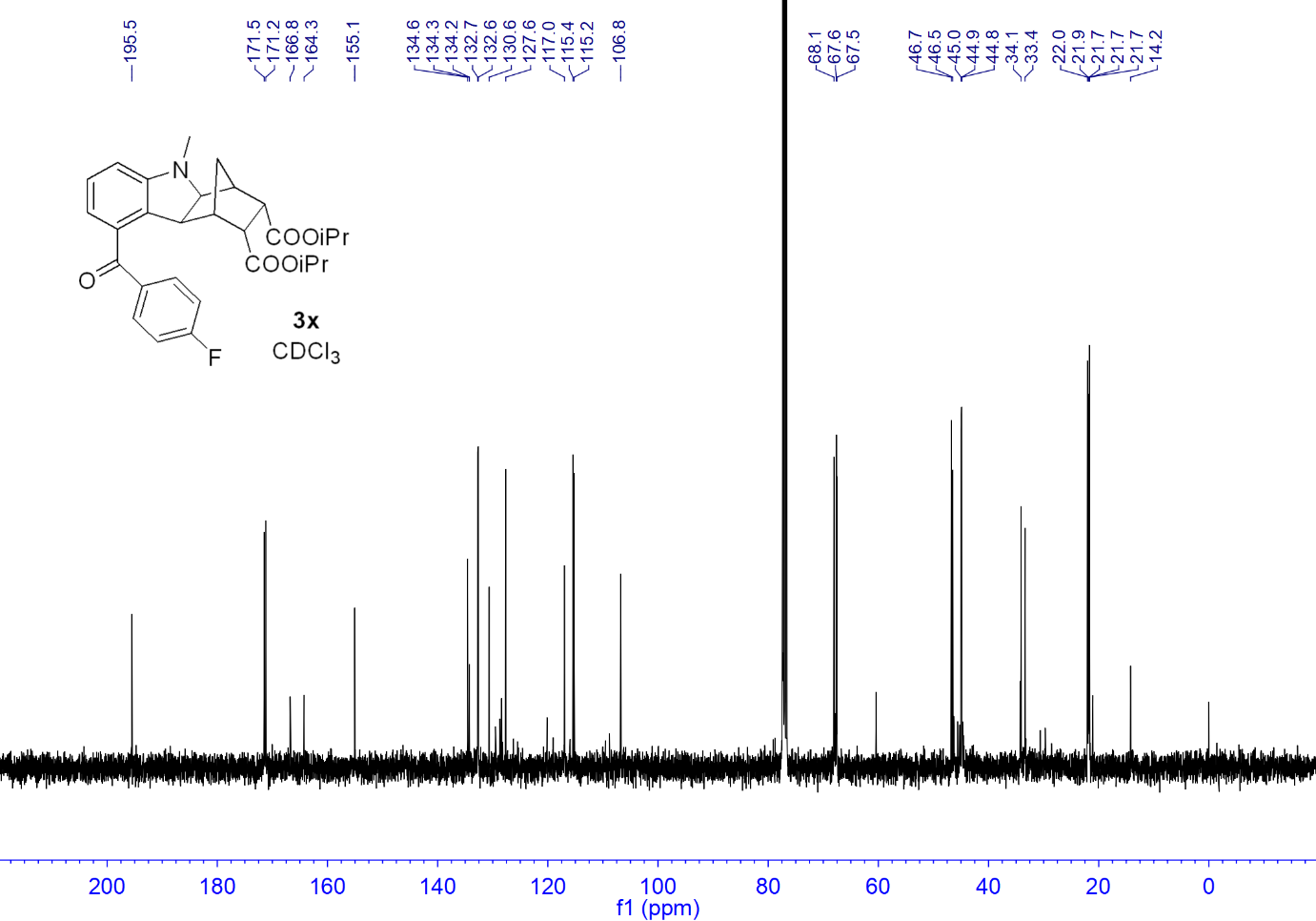


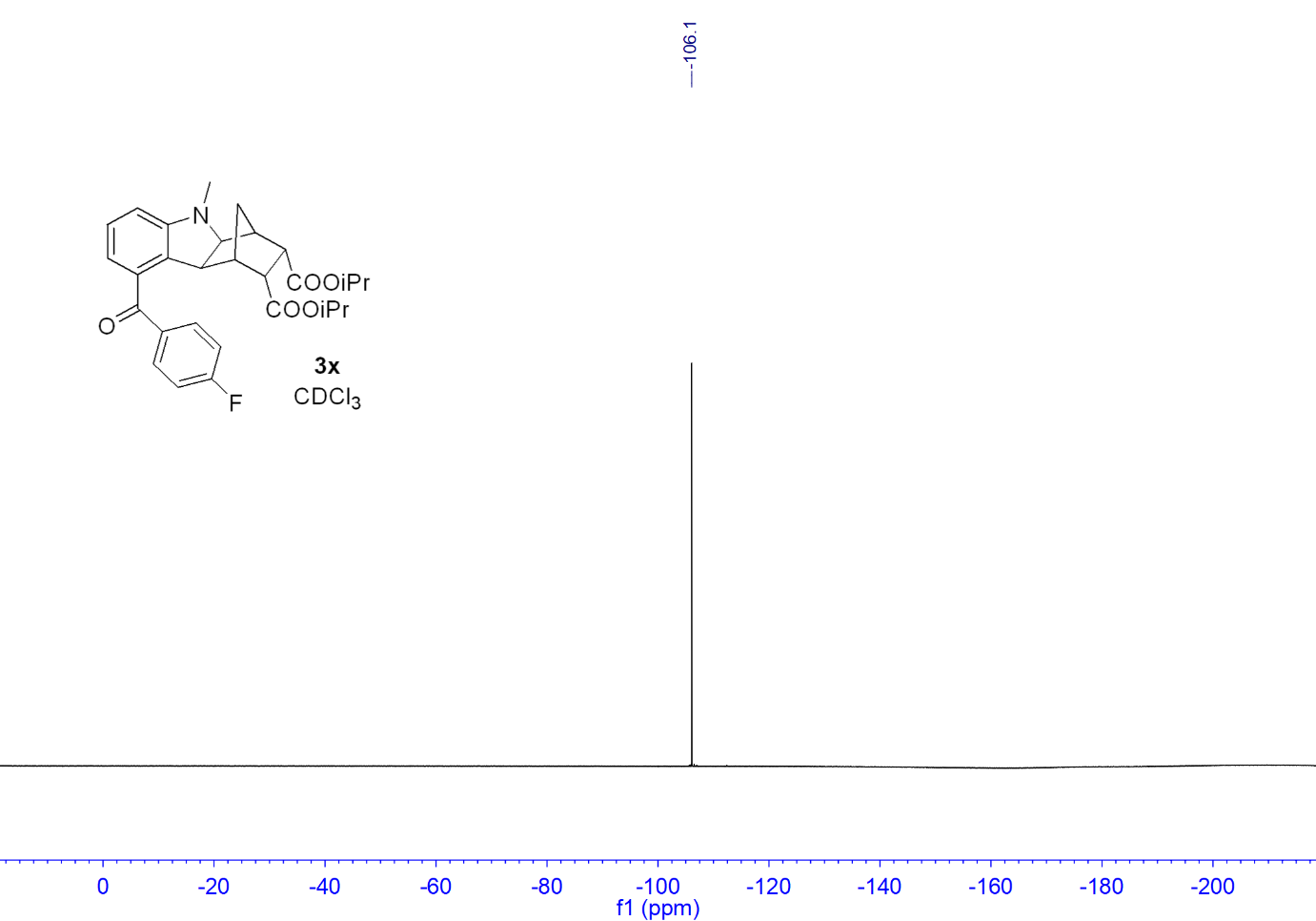

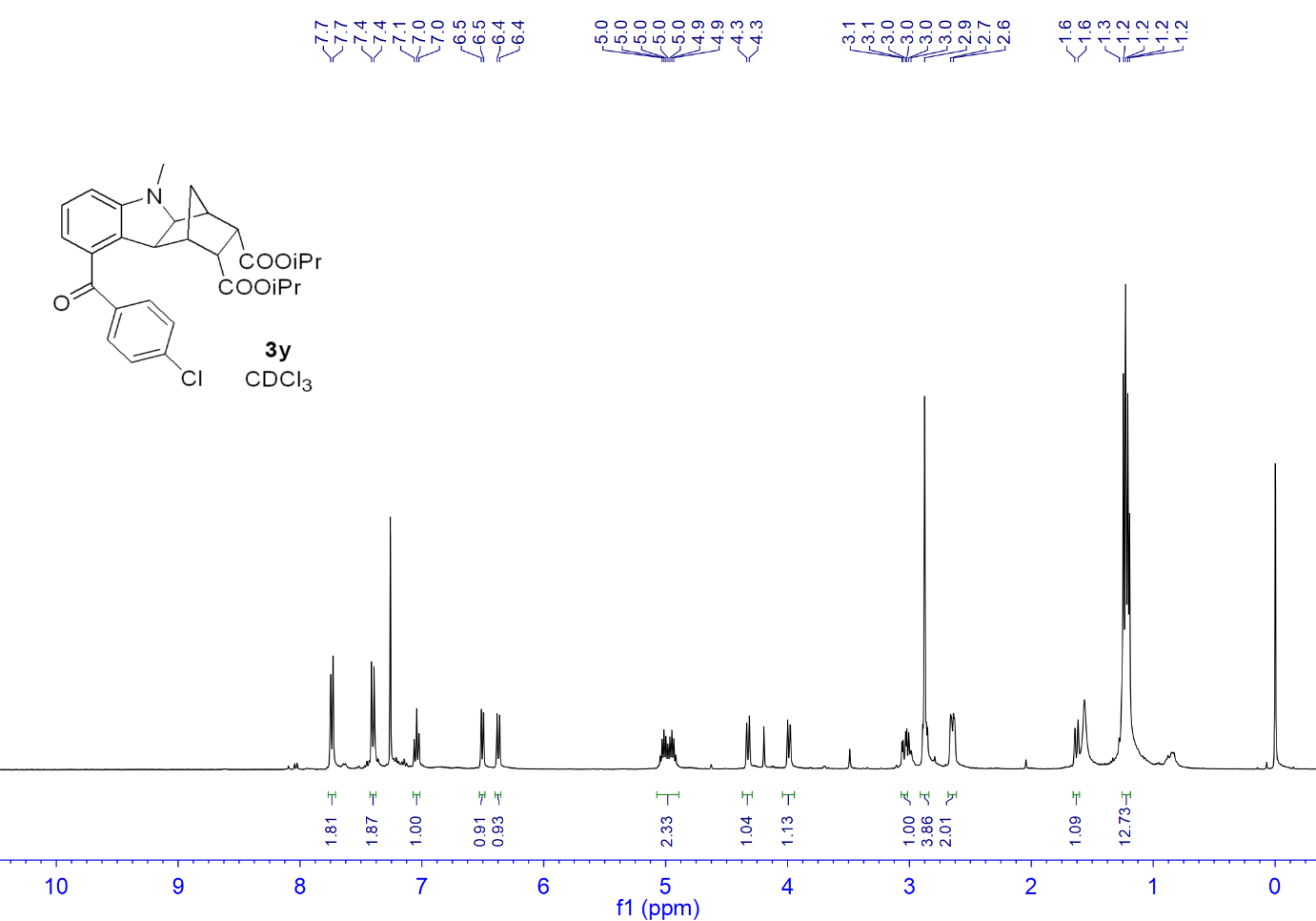


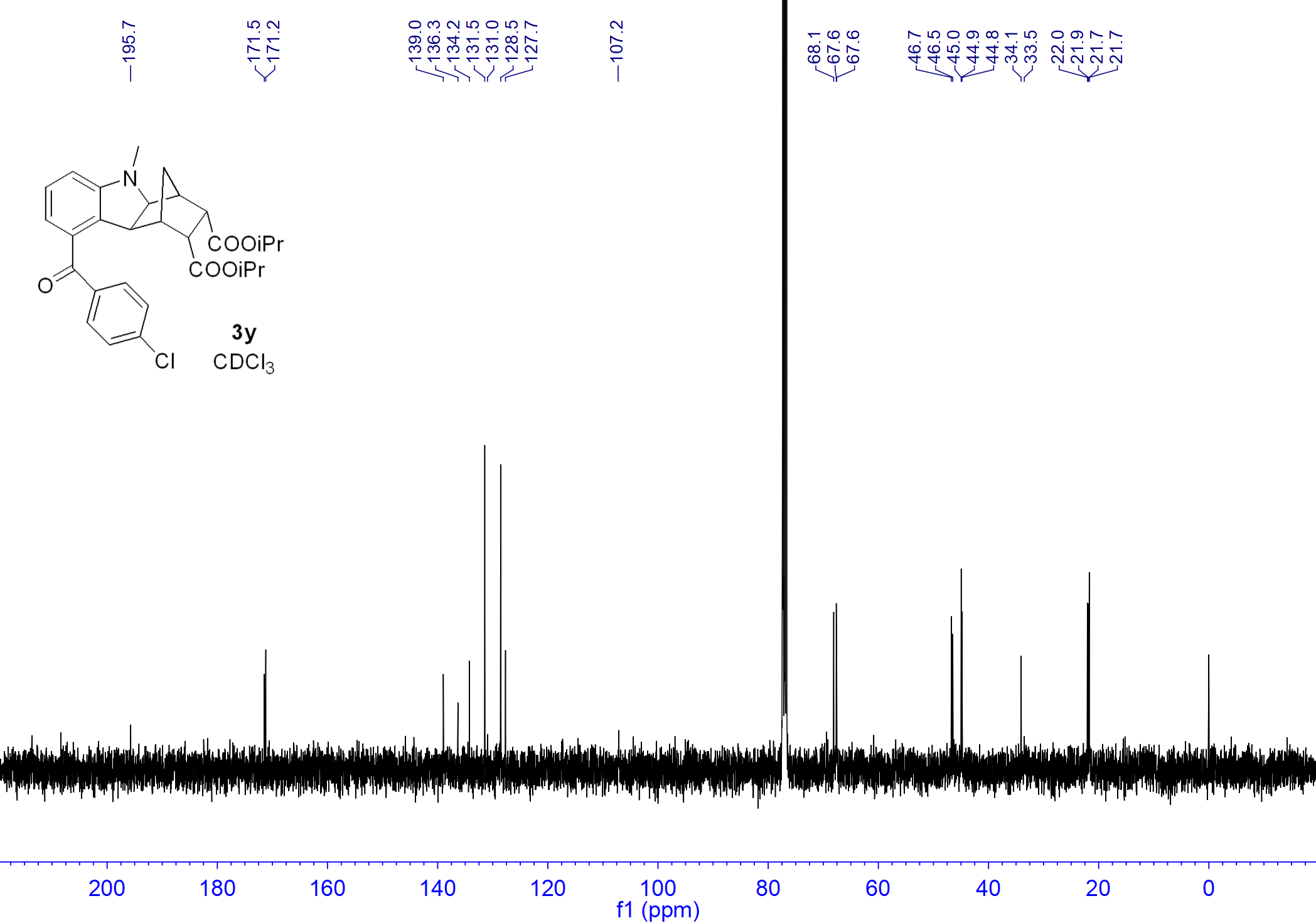

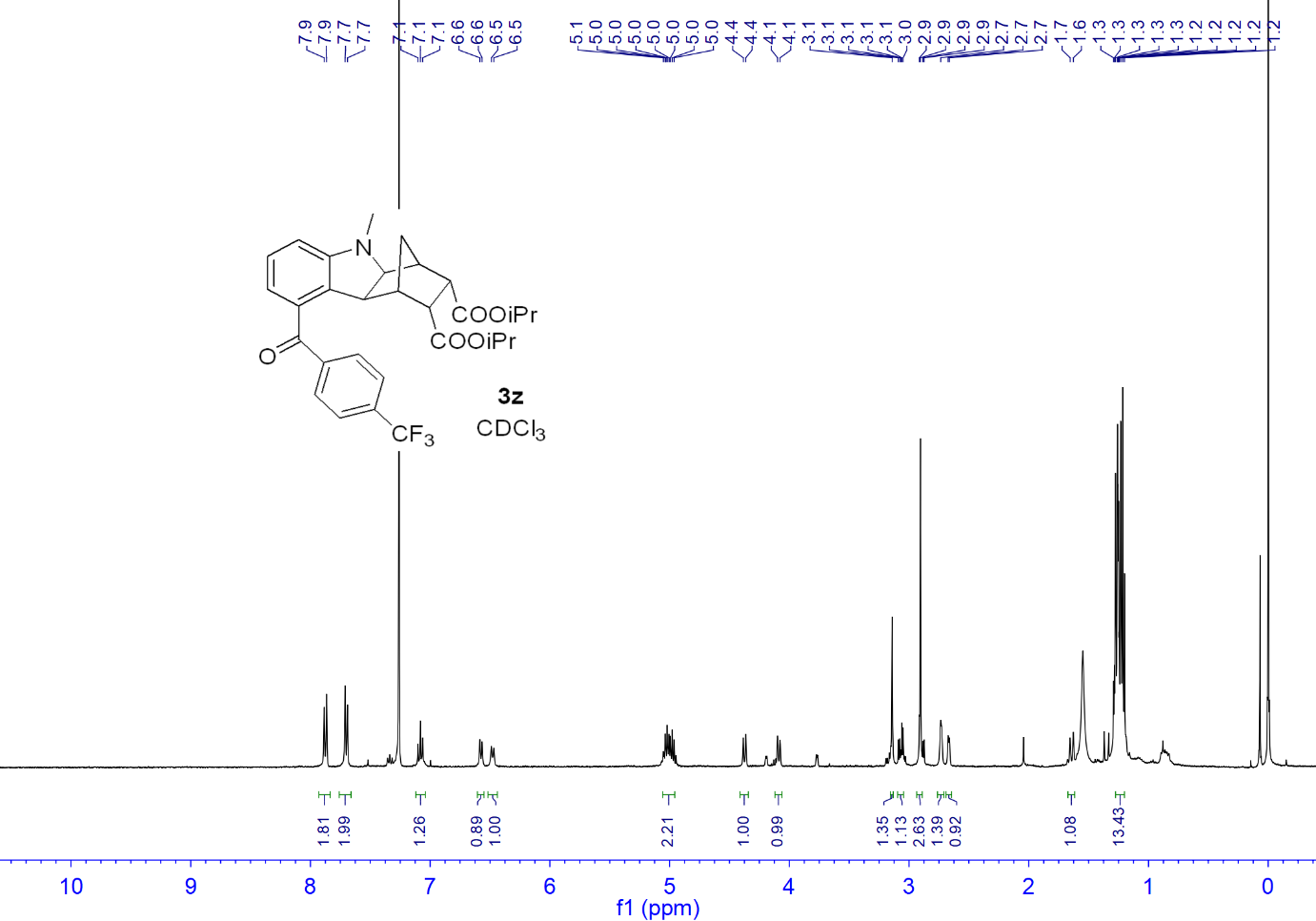


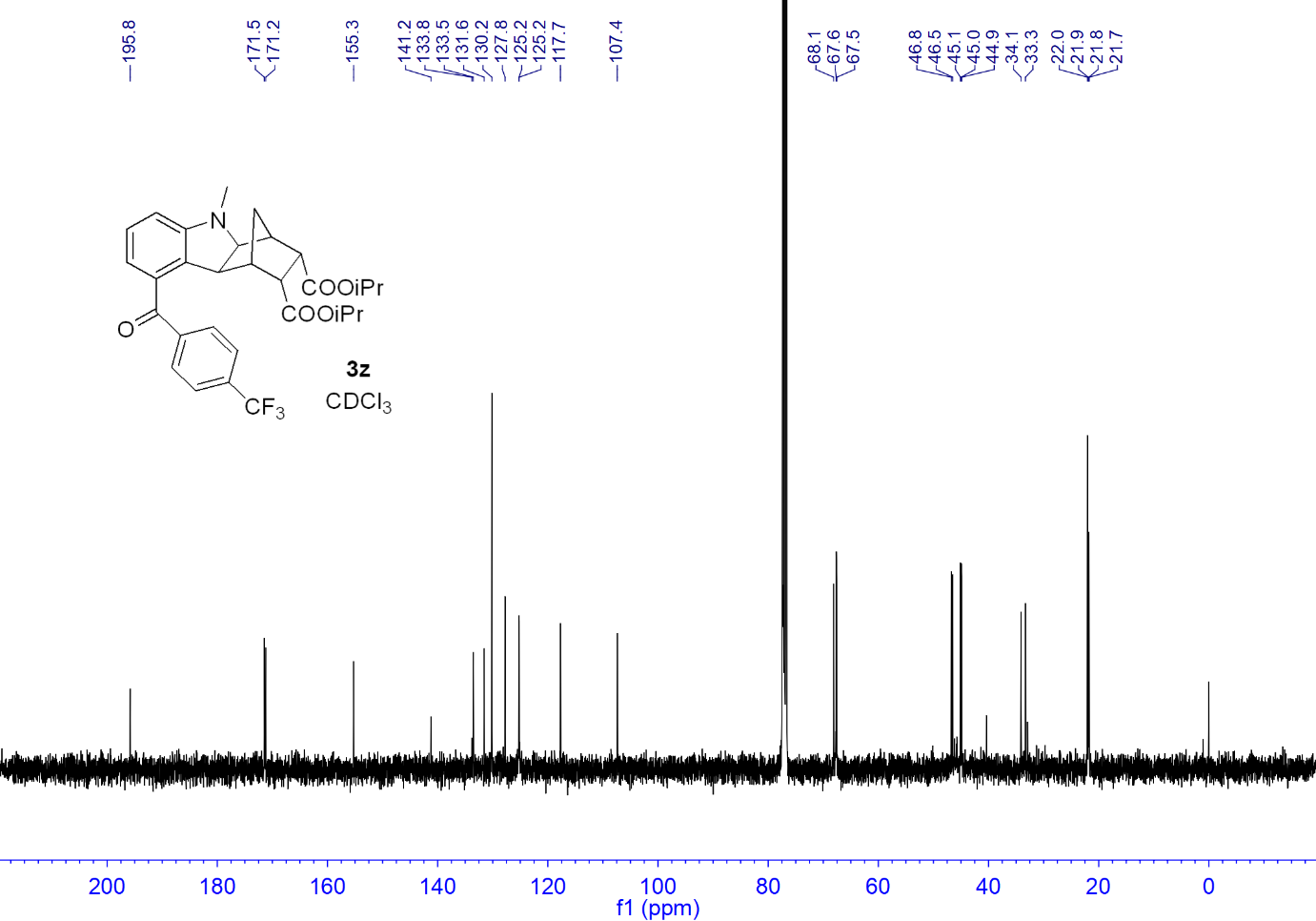


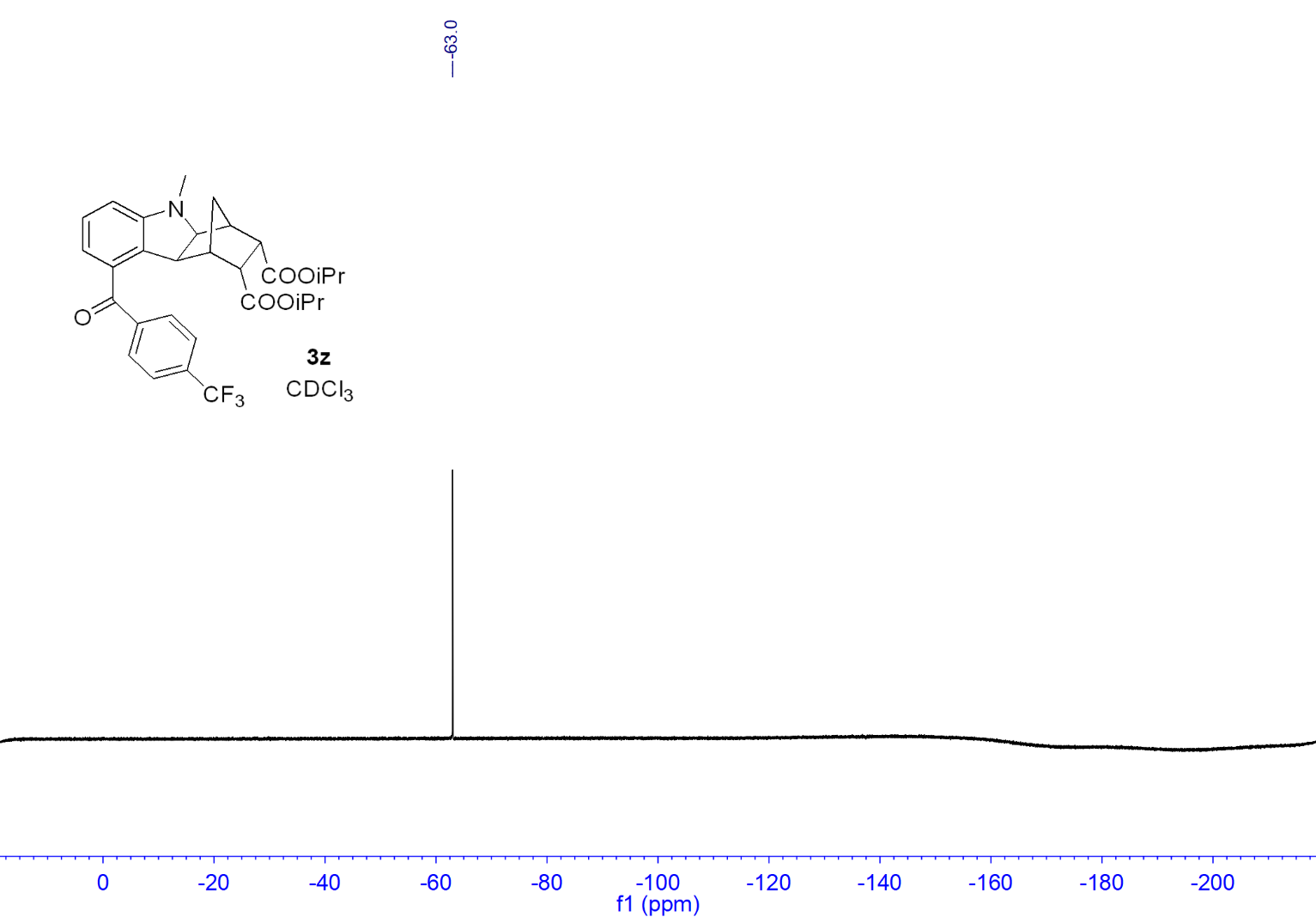

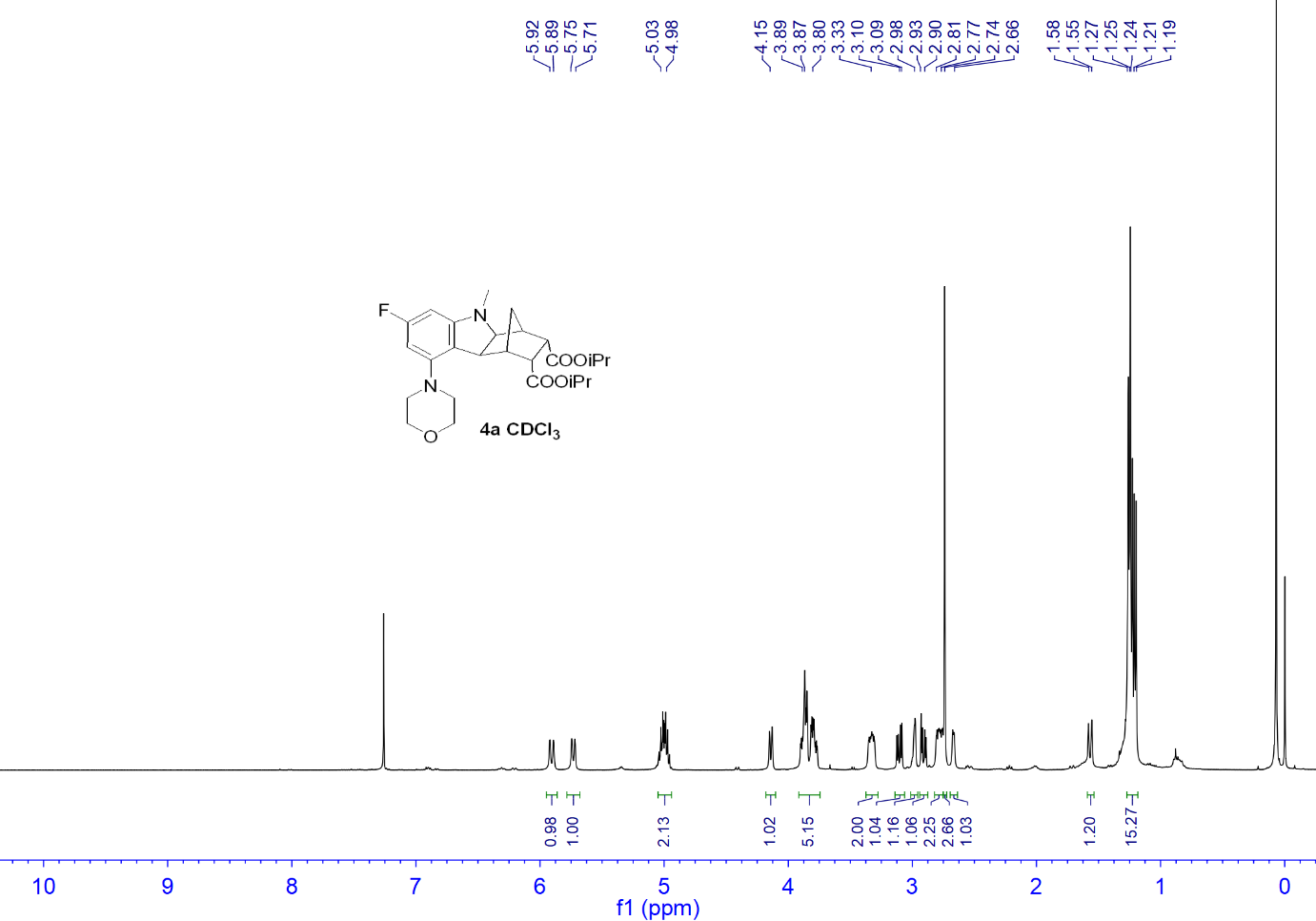


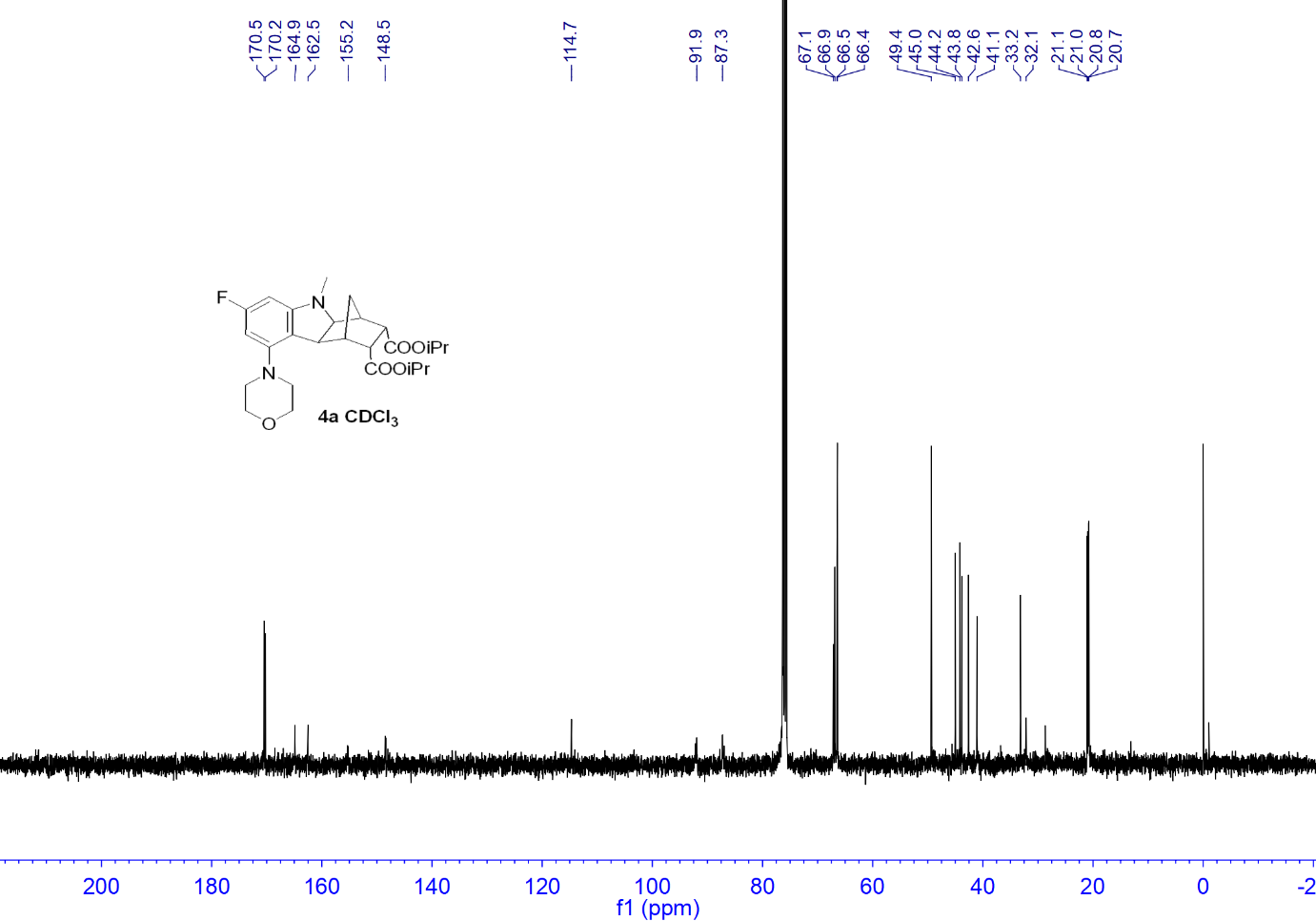

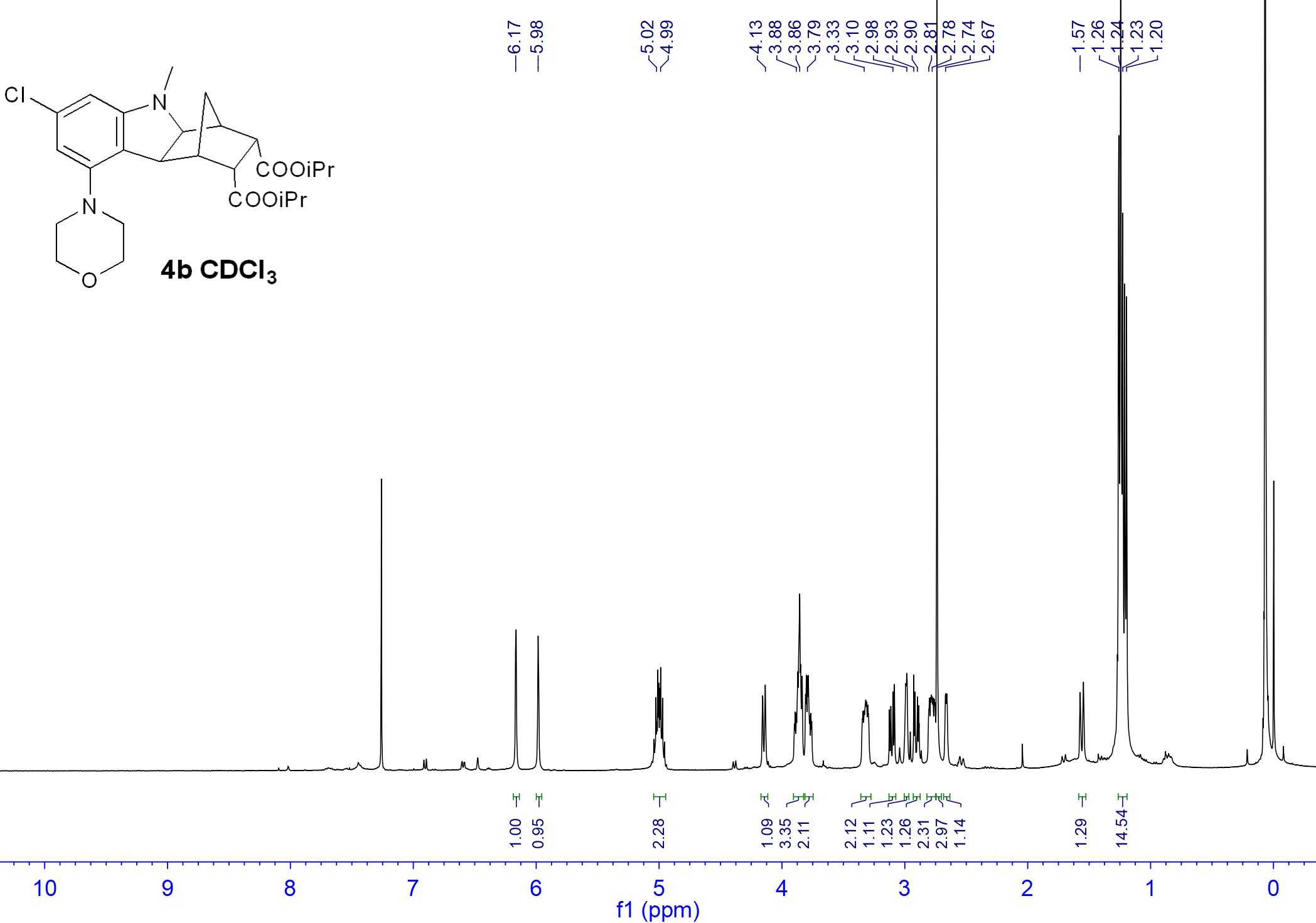


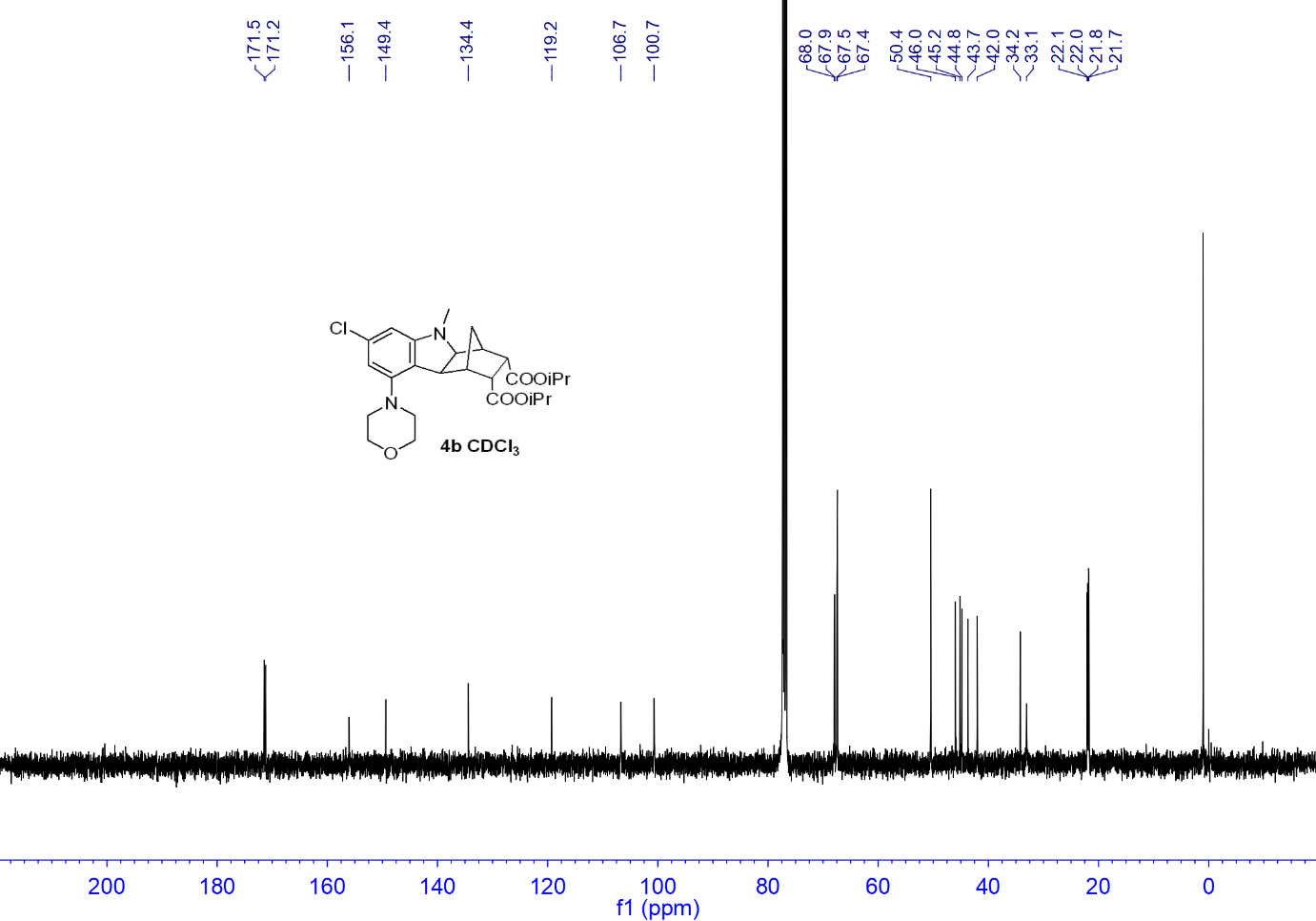

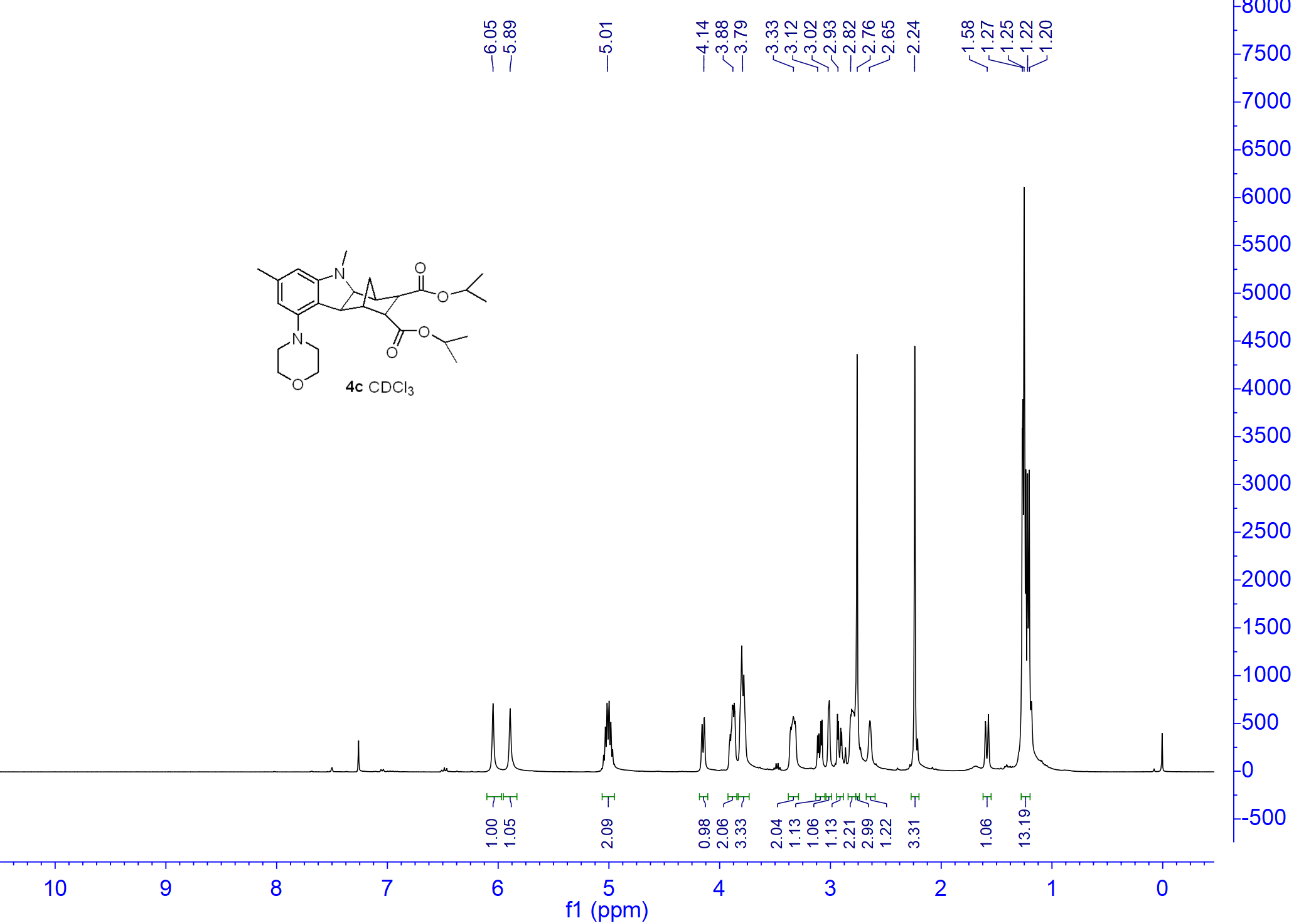


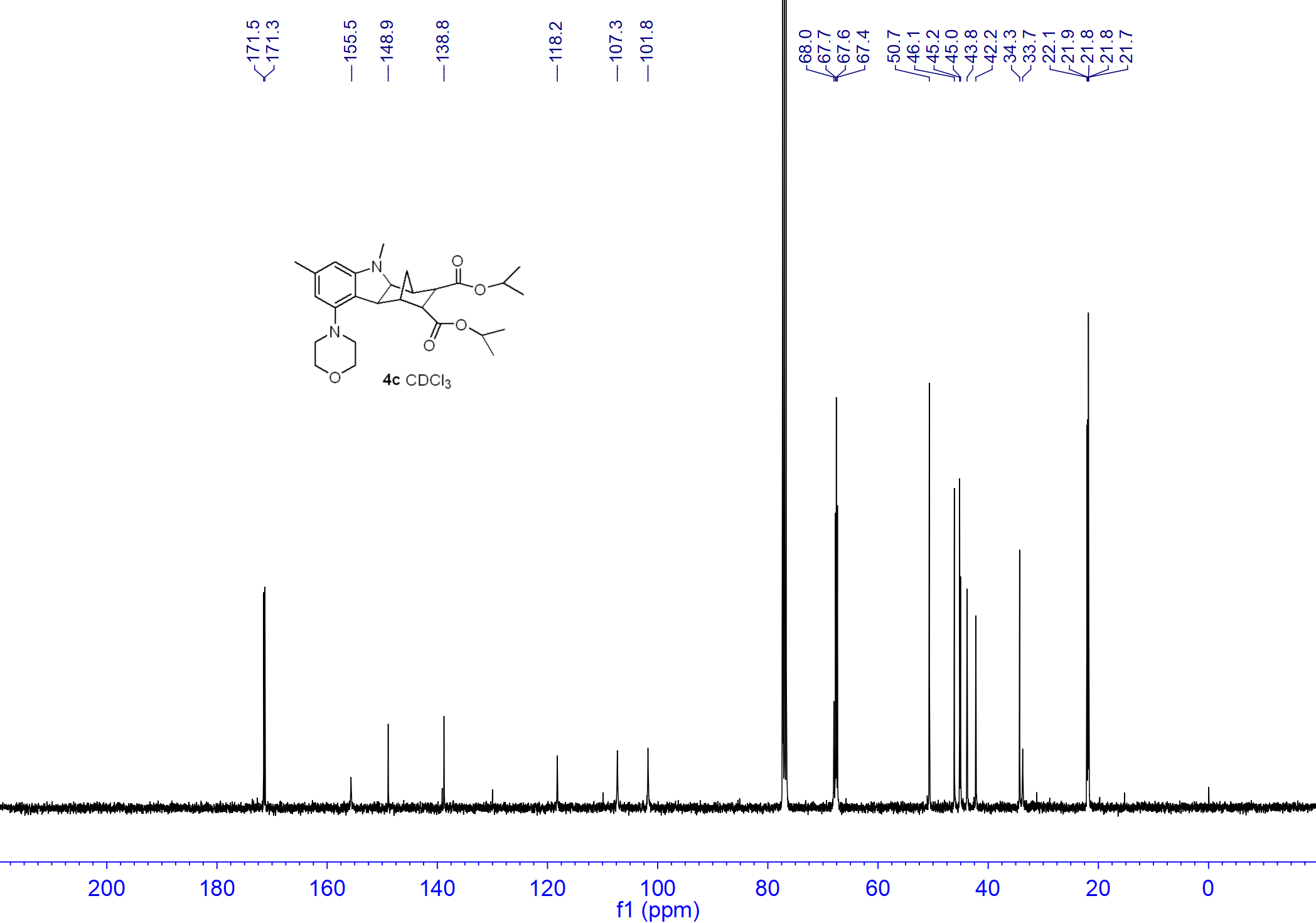

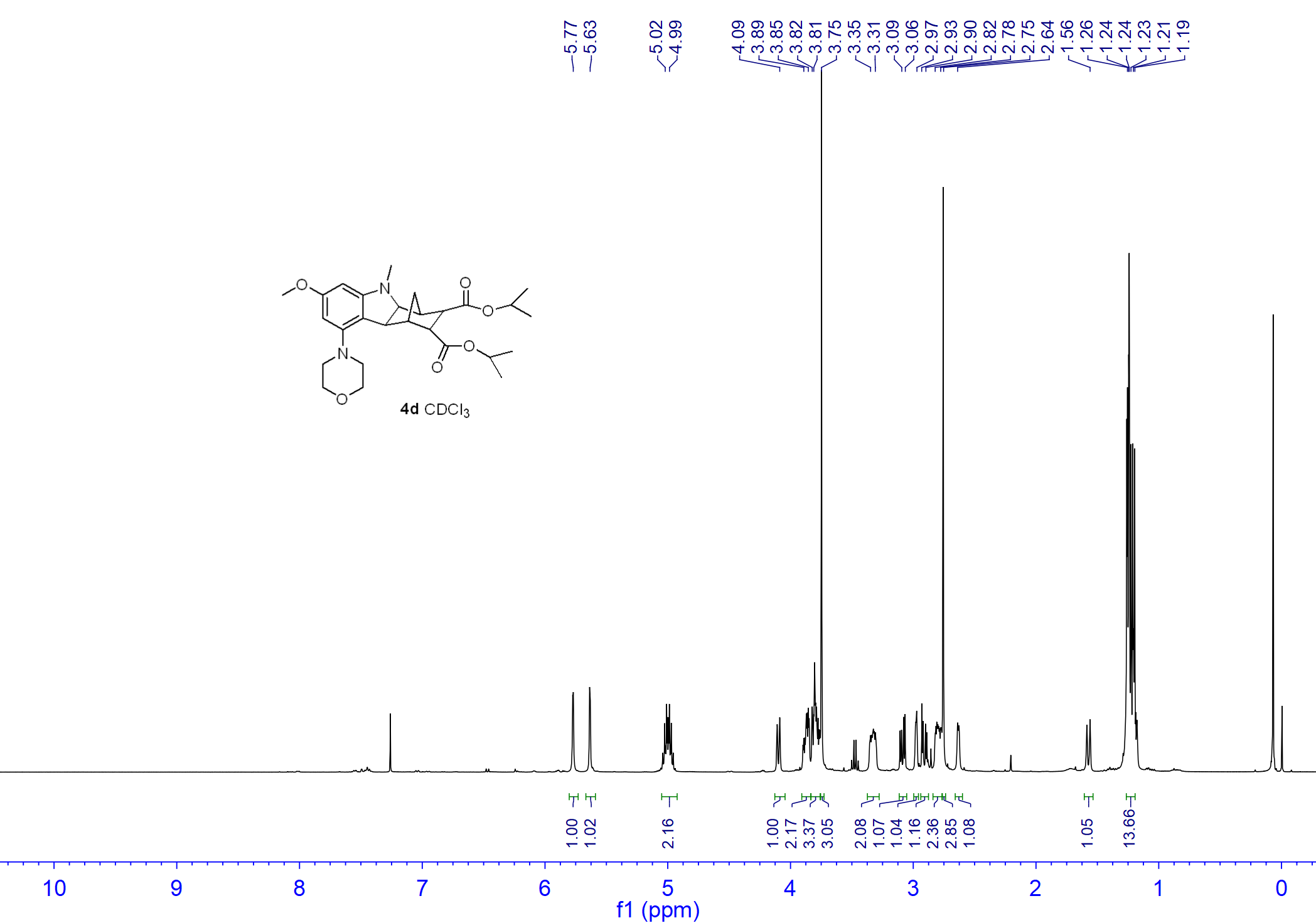


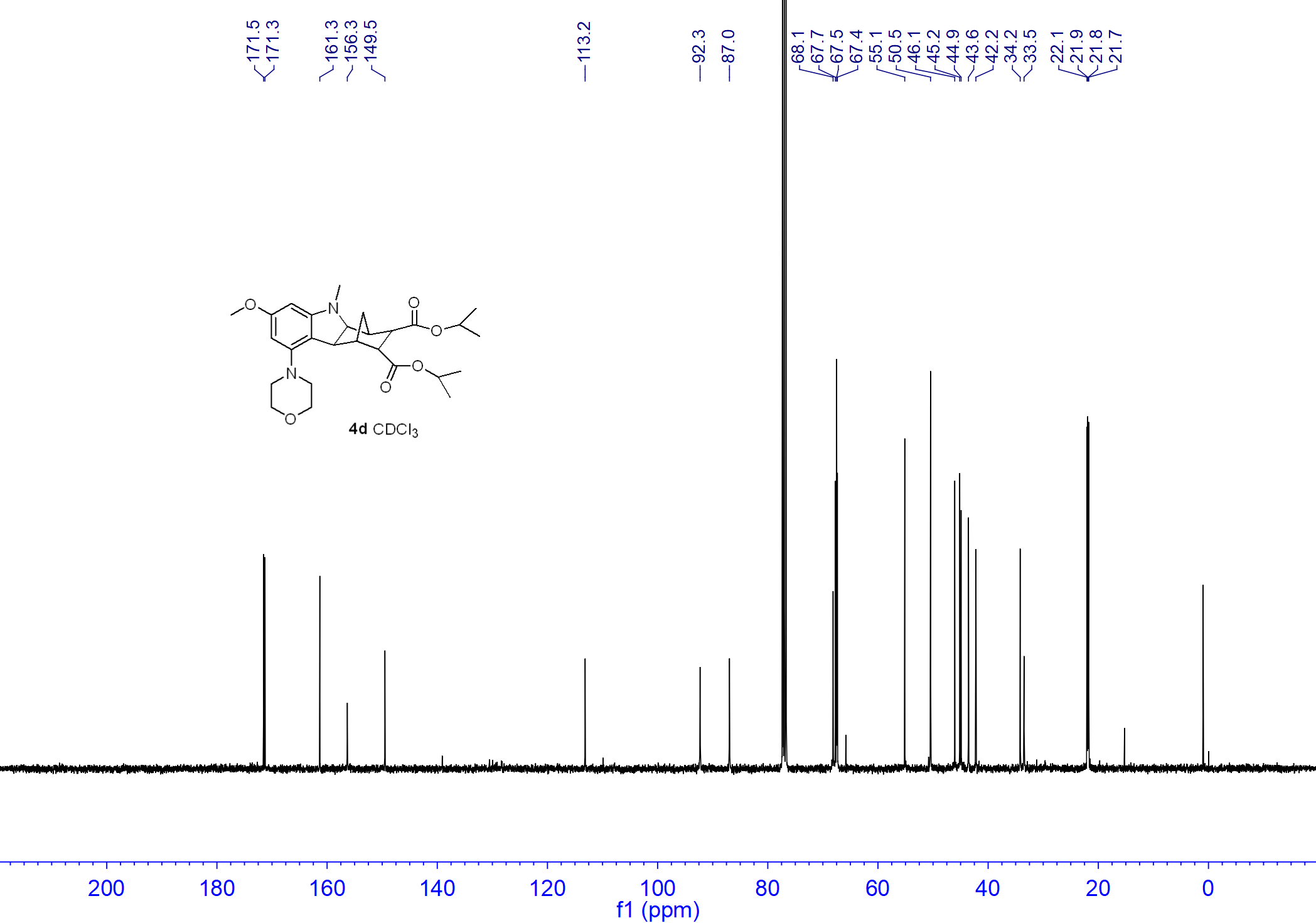

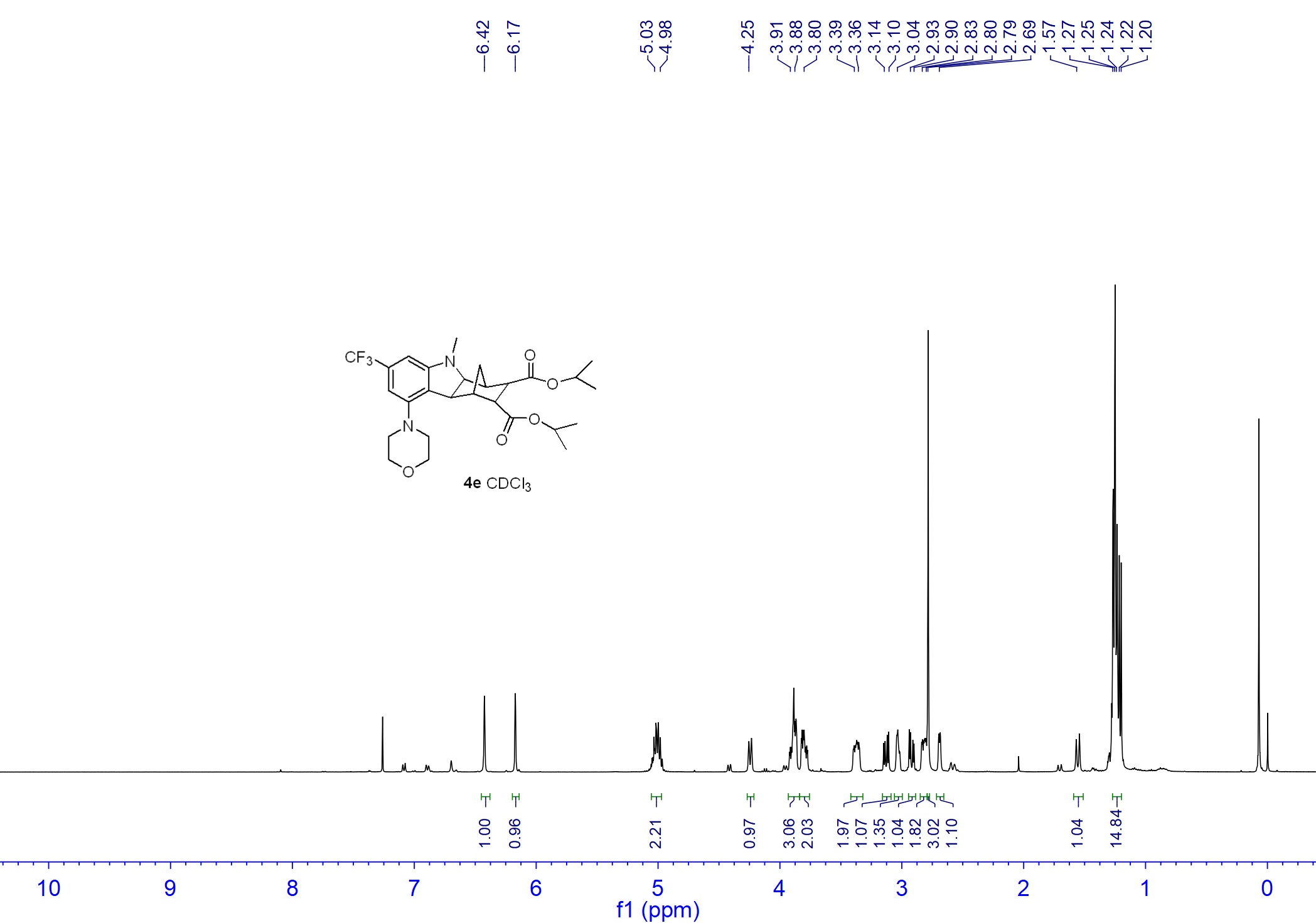


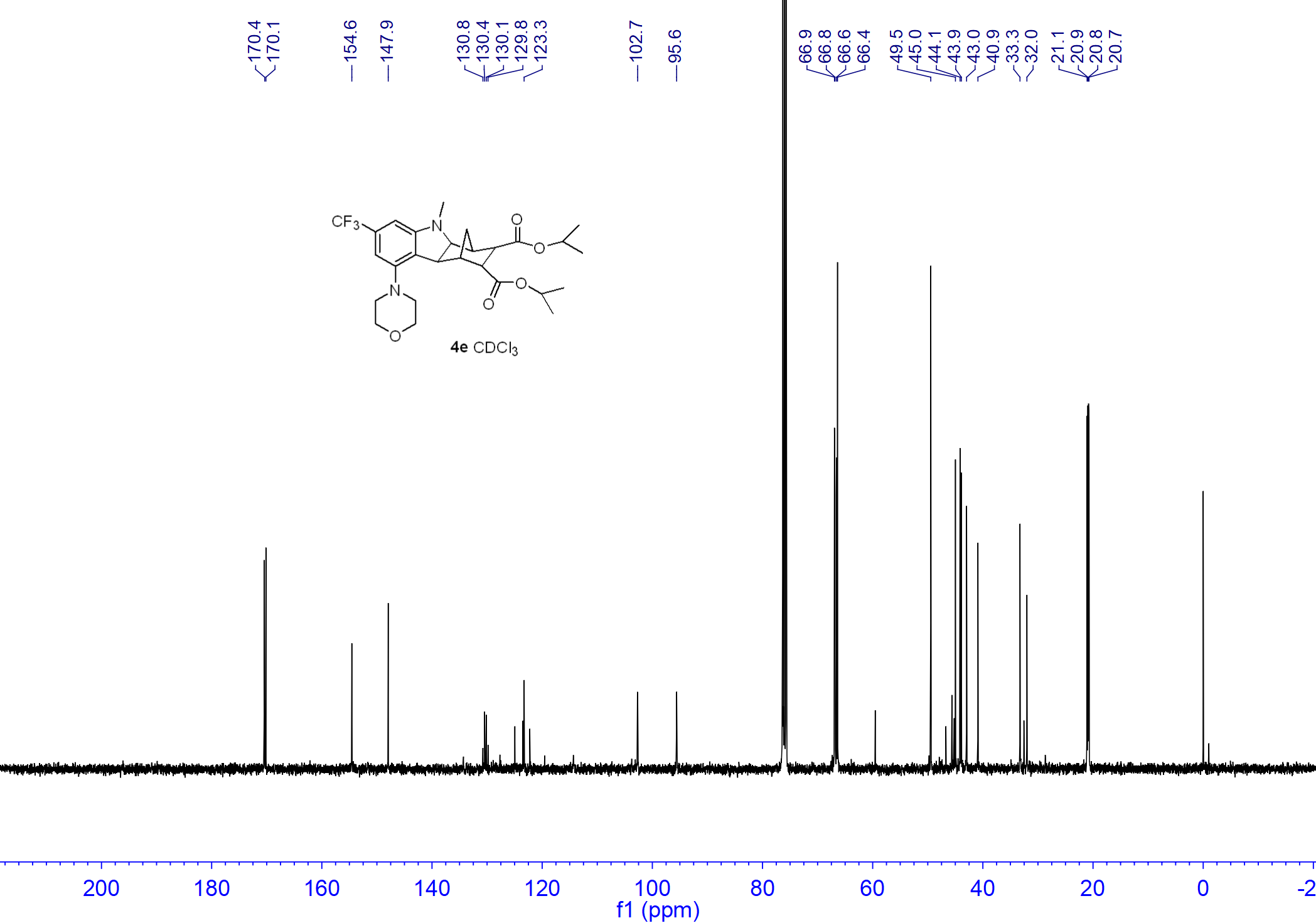

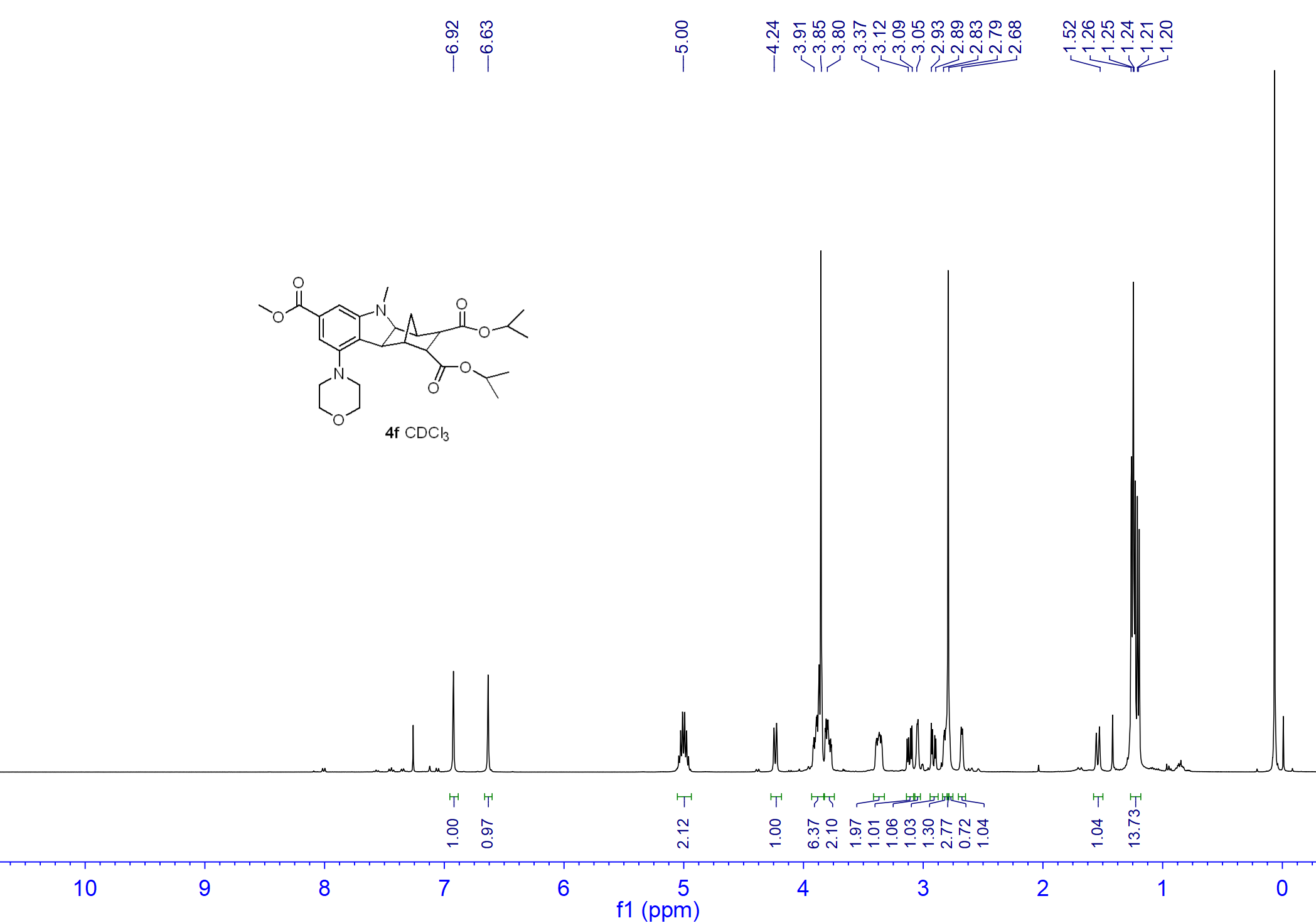


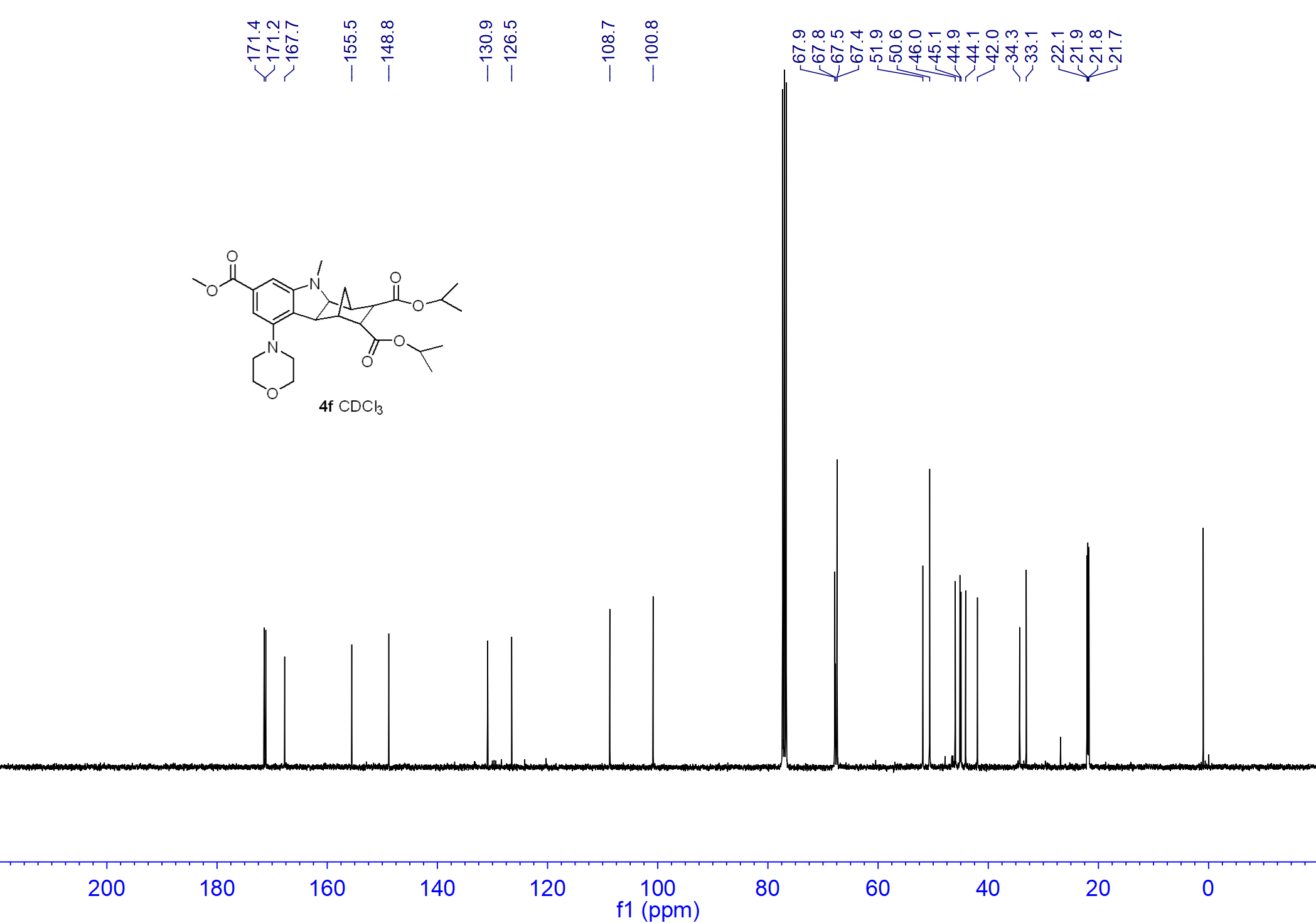

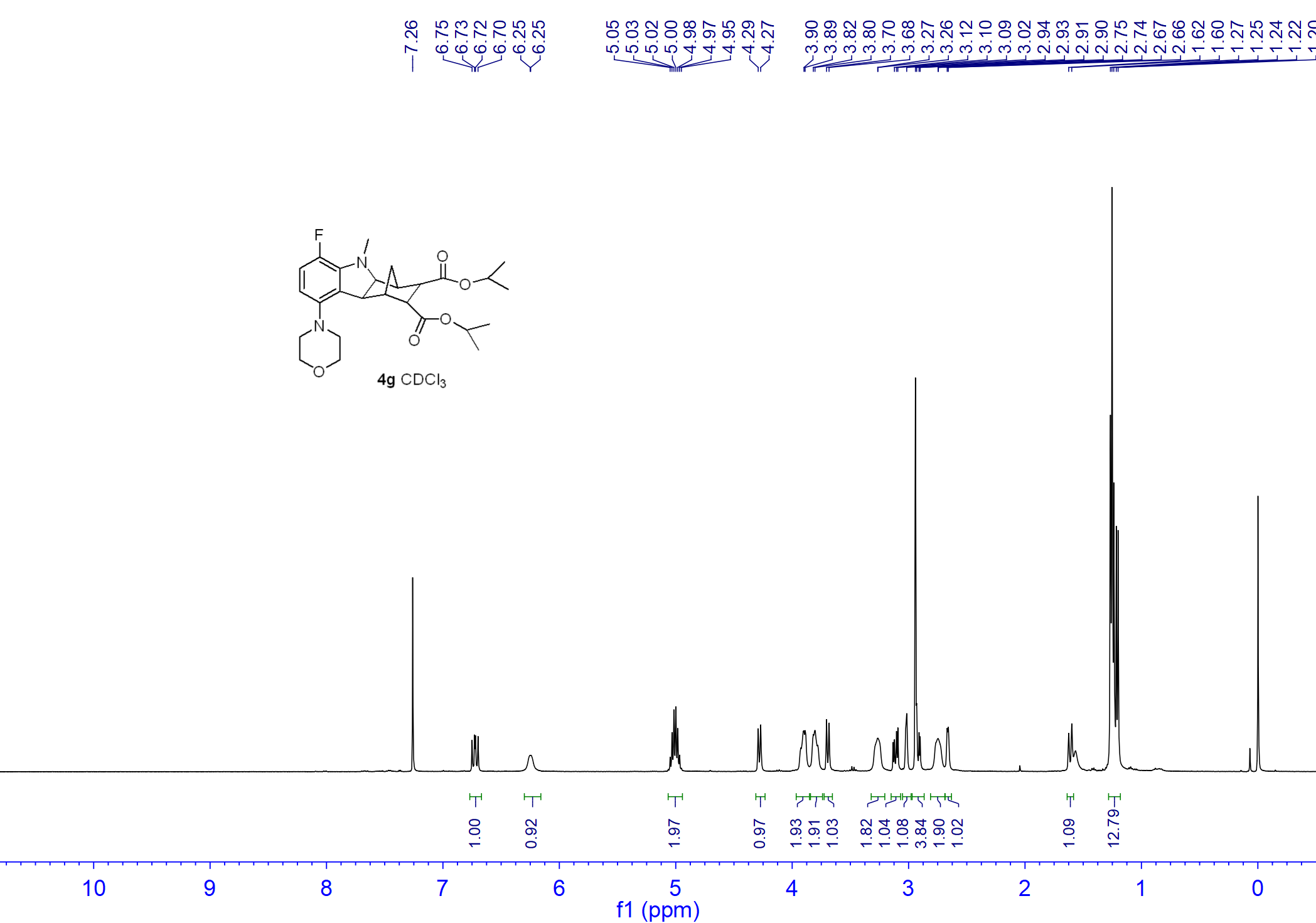


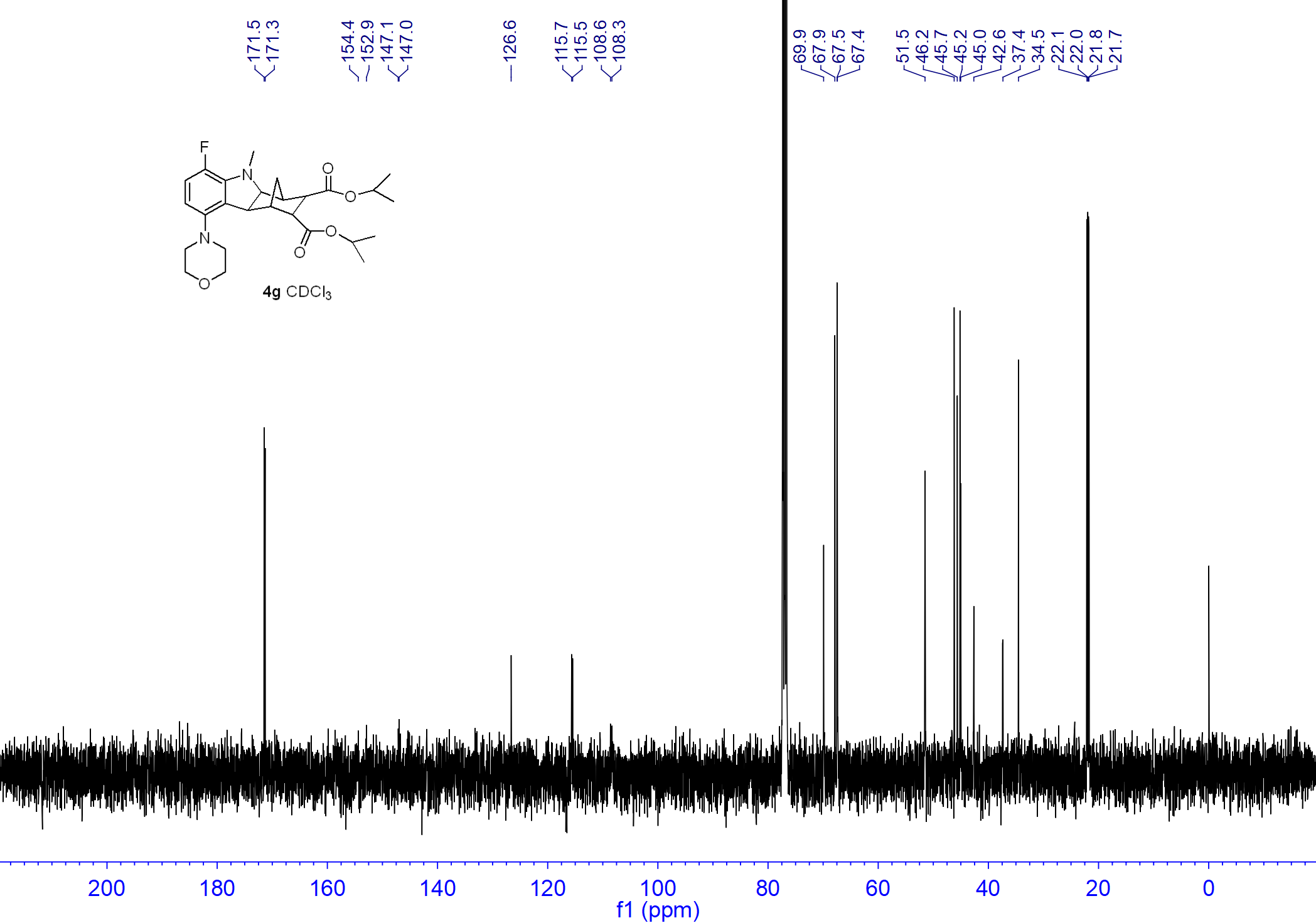

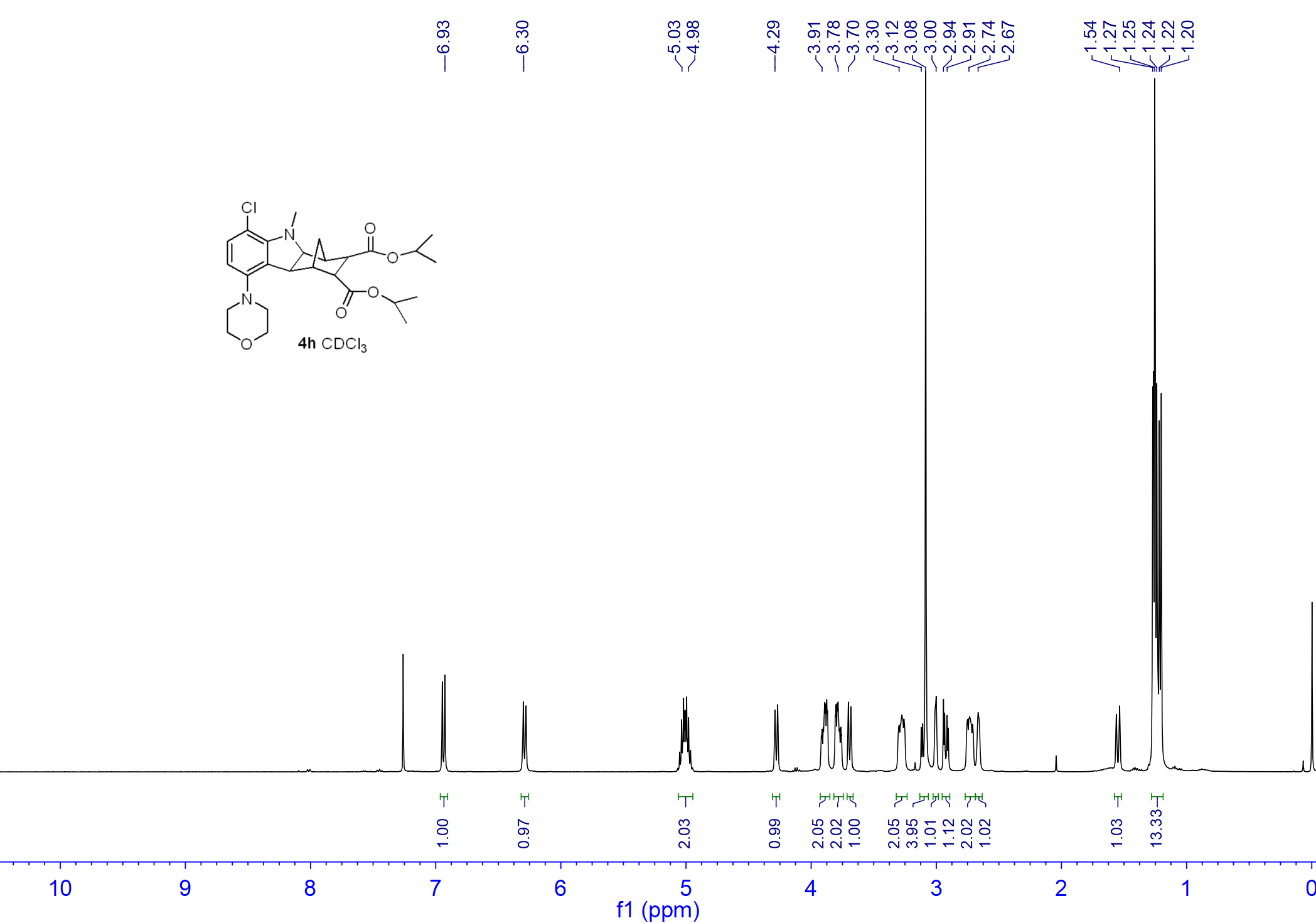


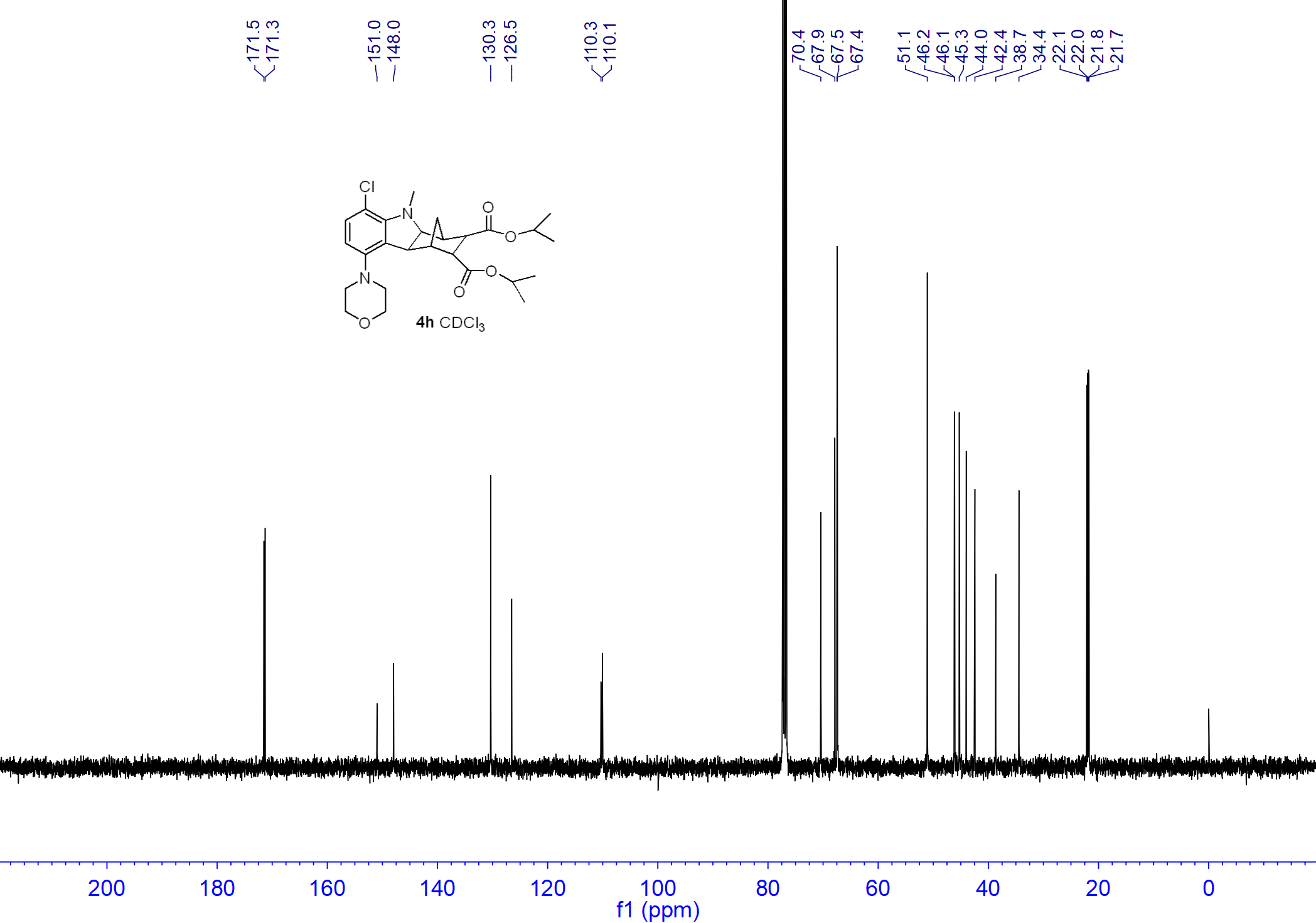

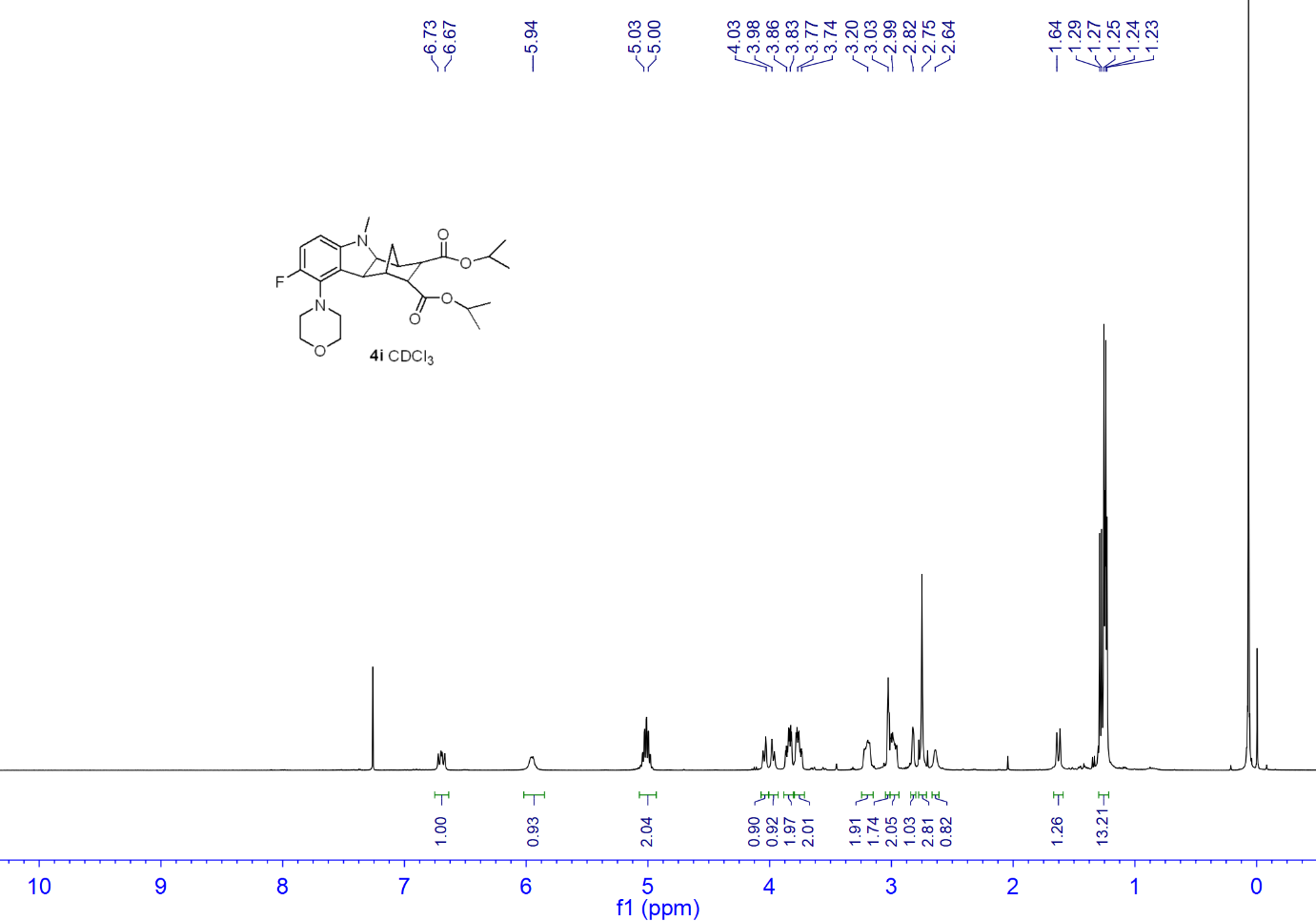


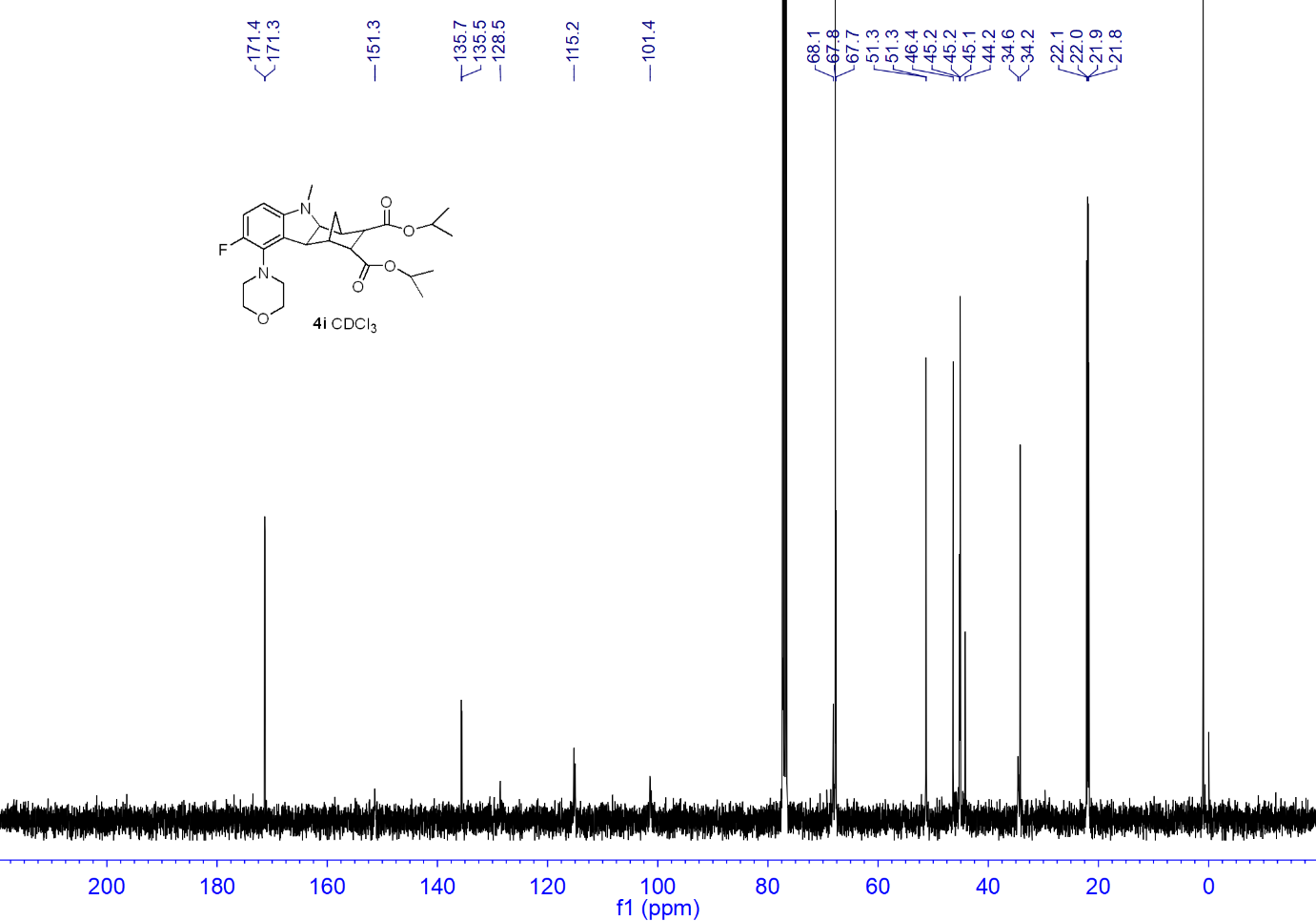

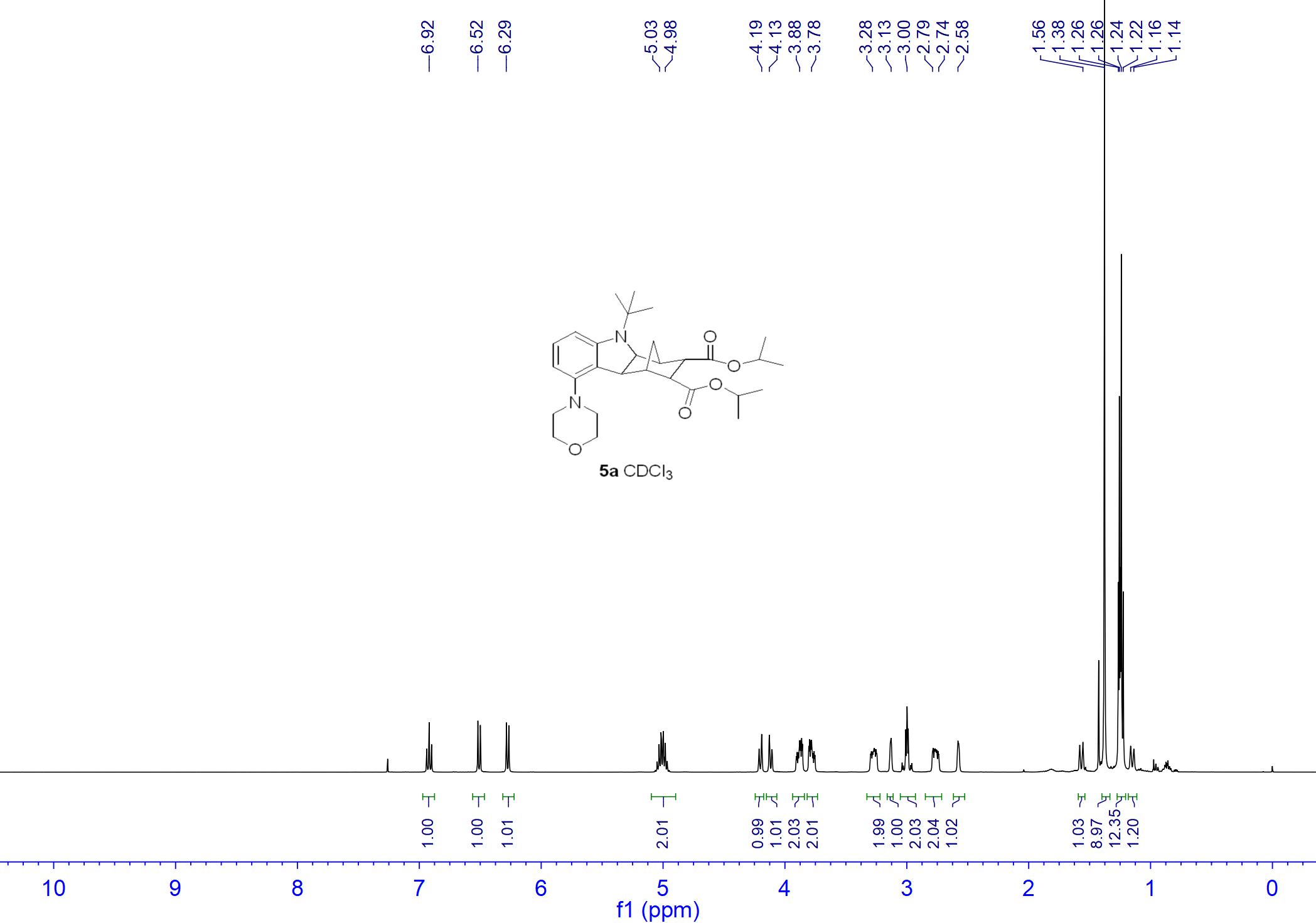


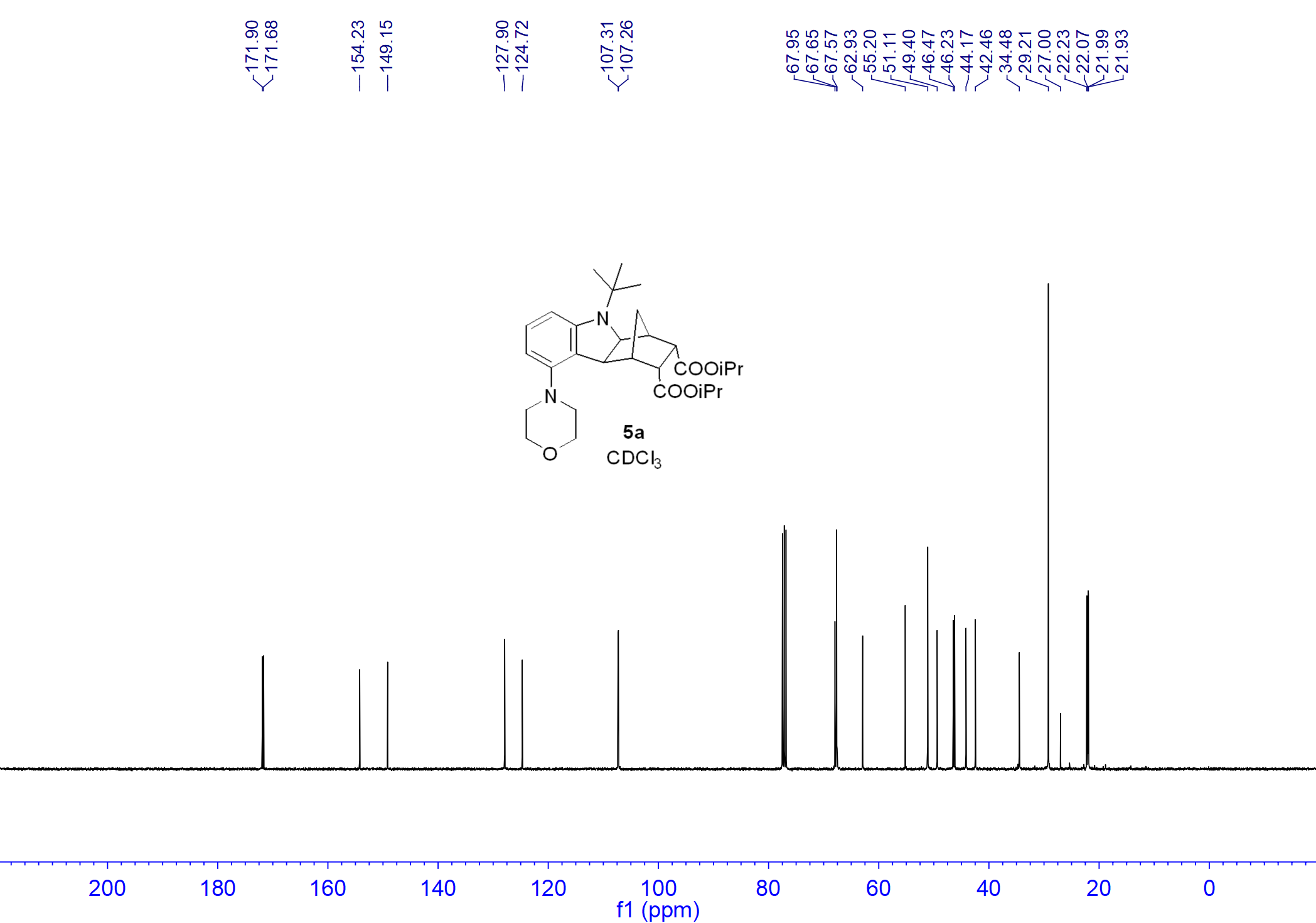

Supplement: Supplementary file 4 — Supplementary Data 2 [file 42004_2022_759_MOESM4_ESM.docx]
