# Supplementary material for: Manipulations of phenylnorbornyl palladium species for multicomponent construction of a bridged polycyclic privileged scaffold
Source: Commun Chem. 2022 Oct 29;5:140. doi: 10.1038/s42004-022-00759-4 (PMC9814782; doi:10.1038/s42004-022-00759-4)
Supplement: Supplementary file 5 — Supplementary Data 3 [file 42004_2022_759_MOESM5_ESM.docx]

Manipulations of Phenylnorbornyl Palladium Species for Multicomponent Construction of a Bridged Polycyclic Privileged Scaffold

Lina Yin,*^[a]^ Ting Guan,^‡[a]^ Jie Cheng,^‡[a]^ Dongchao Pan,^‡[a]^ Jinyang Lu,^‡[a]^ Jiahui Huang,^[a]^ Jiaqi Wu,^[a]^ Xiaoli Chen,^[a]^ Taiyun You,^[a]^ Xuting Huo,^[a]^ Yuting He,^[a]^ Jiayun Pang,*^[b]^ and Qingzhong Hu*^[a]^

[a] T. Guan, J. Cheng, D. Pan, J. Lu, M. Liu, J. Huang, J. Wu, X. Chen, T. You, X. Huo, Y. He, Prof. Dr. L. Yin, and Prof. Dr. Q. Hu
School of Pharmaceutical Sciences, Guangzhou University of Chinese Medicine
232 East Waihuan Road, Panyu, Guangzhou, China PR
E-mail: huqqzh@gzucm.edu.cn (Q. Hu); [linayin@gzucm.edu.cn](mailto:linayin@gzucm.edu.cn) (L. Yin)

[b] Dr. J Pang
School of Science, Faculty of Engineering and Science, University of Greenwich
Medway Campus, Central Avenue, Chatham Maritime, ME4 3RL, United Kingdom
Email: [j.pang@gre.ac.uk](mailto:j.pang@gre.ac.uk) (J. Pang)

^‡^ These authors contribute equally to this work.

**Cartesian coordinates of all the structures reported and their absolute energies in Hartree. For naming of the structures, see the manuscript.**

**Int_I** (5,6-DiCOOiPr NBE)

M06-2X single point: -2684.881339 Hartree

B3LYP-D3 optimization: -2349.008606 Hartree

B3LYP-D3 thermal correction to Gibbs free energy: 0.594400 Hartree

Pd -0.30594700 -0.43534500 0.51710200

P -0.21358500 -0.04147700 2.95922900

C 3.30495100 -2.54204500 0.55158000

H 3.96582100 -2.01253200 1.23186300

C 3.31319400 -3.93170200 0.48428200

H 3.99542700 -4.51404500 1.09884000

C 2.41496000 -4.59197600 -0.35657500

H 2.40819800 -5.67572900 -0.36898600

C 1.51172800 -3.87445000 -1.15987000

C 1.54012600 -2.45045400 -1.14046400

C 2.42334200 -1.81794700 -0.25826600

C 0.64252600 -1.66543200 -2.09219800

H -0.02837800 -2.38910000 -2.54426800

C -0.25540700 -0.49991700 -1.55926500

H -1.29316200 -0.70775600 -1.81885300

C 0.24119500 0.75328700 -2.32347600

H 0.04765900 1.69890600 -1.82061700

C -0.28911600 0.70558000 -3.77902600

H 0.11647900 1.57385500 -4.31675200

C 0.42456700 -0.56006500 -4.36944000

H 0.98356800 -0.31318800 -5.27579300

C 1.43689500 -0.94204300 -3.22232000

H 2.28739800 -1.51496300 -3.59677200

C 1.72234300 0.43570200 -2.59674000

H 2.19154000 1.13532800 -3.30020000

H 2.30728400 0.41108900 -1.68067000

C -0.71999500 -1.18390400 4.28300700

C -1.18283300 -2.47104400 4.23277200

C -1.36521800 -2.89476800 5.58820300

H -1.37204500 -3.03211300 3.32904400

C -0.99804500 -1.83851300 6.36796500

H -1.72151200 -3.85547800 5.93096300

H -0.96492600 -1.68019600 7.43553600

O -0.59880300 -0.78884200 5.60027700

C 1.46474600 0.41485400 3.54111700

C 1.98966100 1.43474700 4.28792300

C 3.38223900 1.13469000 4.46167500

H 1.44351100 2.28547200 4.66626200

C 3.60205200 -0.04460800 3.80765900

H 4.11138000 1.70551600 5.02077500

H 4.46968700 -0.67147900 3.66802100

O 2.44853500 -0.49328900 3.24648300

C -1.12150100 1.48264800 3.42844600

C -0.94547500 2.80041500 3.09693000

C -2.07951700 3.50007300 3.63146400

H -0.13225800 3.19649400 2.49638400

C -2.86670400 2.56258400 4.23465600

H -2.27636200 4.56196500 3.57666700

H -3.80080200 2.60394400 4.77423500

O -2.30109700 1.32654500 4.12106700

I -1.91466900 -2.70083400 0.42805500

N 0.58701100 -4.54530500 -1.98767000

H -0.32264000 -4.09842800 -1.93857800

C 0.44813700 -5.98961400 -1.88377200

H 0.22800800 -6.34321500 -0.86348000

H -0.36575600 -6.30481600 -2.54159000

H 1.36231900 -6.48608600 -2.22569900

H 2.40517700 -0.73746700 -0.15638700

C -0.46907000 -1.73817200 -4.68381800

O -1.62963200 -1.86881600 -4.33569600

O 0.23046700 -2.68805400 -5.32878100

C -0.40285400 -3.99396500 -5.49893600

C 0.73670200 -4.99928800 -5.51953500

C -1.24892400 -3.97497400 -6.76534300

H -1.04028900 -4.16331800 -4.62633100

H 1.29237800 -4.94015600 -4.58046400

H 0.33746700 -6.01250900 -5.62924900

H 1.41381500 -4.80074100 -6.35670300

H -2.03458600 -3.21963600 -6.68425200

H -0.62622600 -3.74878000 -7.63702100

H -1.71944600 -4.95133100 -6.91882300

C -1.78255600 0.84135300 -3.97857500

O -2.59723400 1.07586300 -3.09838400

O -2.09425500 0.80822400 -5.28506200

C -3.49823500 0.93203800 -5.65947000

C -4.20662800 -0.40008000 -5.44288400

C -3.49254700 1.38617800 -7.11131700

H -3.94440500 1.69994400 -5.01878500

H -4.14676200 -0.69982100 -4.39578400

H -5.25906600 -0.31044900 -5.73150200

H -3.73764200 -1.18148000 -6.04709800

H -2.95392300 2.33168000 -7.22174400

H -3.00940200 0.63486000 -7.74410500

H -4.51898200 1.52613700 -7.46353300

C 1.36058700 2.09829300 0.63513700

O 1.44146300 3.34641300 0.90971000

O 2.36328500 1.32621100 0.49553700

O 0.11793900 1.61528800 0.51191200

Cs -2.91357900 1.86151500 -0.05161800

Cs 4.44821700 3.23388800 1.44545400

**TS1** (5,6-DiCOOiPr NBE)

M06-2X single point: -2684.843977 Hartree

B3LYP-D3 optimization: -2348.972113 Hartree

B3LYP-D3 thermal correction to Gibbs free energy: 0.589668 Hartree

Imaginary frequency: -838.2052 cm^-1^

Pd -0.22891100 -0.40749500 0.42892400

P -0.21012600 -0.05289600 2.85639500

C 2.62275600 -1.79885900 1.18219200

H 2.78143600 -1.15942200 2.04274400

C 3.25016000 -3.03783100 1.10903900

H 3.88052500 -3.38383100 1.92550500

C 3.07678900 -3.85621000 -0.01189900

H 3.57843400 -4.81701000 -0.05283100

C 2.26448700 -3.43658000 -1.08035300

C 1.60821500 -2.18048500 -0.99429700

C 1.79870100 -1.34231300 0.12924400

C 0.65303000 -1.74227200 -2.08162600

H -0.00397500 -2.58636400 -2.33008800

C -0.22099300 -0.54091600 -1.63816600

H -1.26560800 -0.70666200 -1.88814100

C 0.35701200 0.65068000 -2.45352400

H 0.28094100 1.62021700 -1.96645800

C -0.26006500 0.57550900 -3.87343200

H 0.19390200 1.36786000 -4.48623700

C 0.29868200 -0.78572400 -4.42452300

H 0.79819400 -0.64219900 -5.38540000

C 1.37243400 -1.17357700 -3.33905000

H 2.15677100 -1.82138500 -3.73624000

C 1.79216300 0.20902400 -2.81035100

H 2.25175300 0.84384100 -3.57750800

H 2.45115800 0.17180500 -1.94685000

C -1.02291400 -1.28601500 3.90698100

C -1.55311800 -2.50337500 3.57937400

C -1.99041100 -3.10215100 4.80329600

H -1.63686200 -2.89794100 2.57778400

C -1.69388800 -2.20689800 5.78879800

H -2.46580500 -4.06411100 4.92913900

H -1.83665200 -2.20690600 6.85897100

O -1.10176900 -1.09767400 5.26960300

C 1.37730600 0.13011800 3.77803700

C 2.26383800 1.16053900 3.93183800

C 3.35557100 0.64097600 4.70812800

H 2.17661400 2.13976900 3.48013400

C 3.06352300 -0.66786600 4.95733800

H 4.22442500 1.18008900 5.06152200

H 3.56007400 -1.45466700 5.50435800

O 1.86453800 -0.99628800 4.39982000

C -1.03304800 1.51666700 3.33527800

C -0.68897600 2.82452800 3.11844300

C -1.76988900 3.61304400 3.63907700

H 0.20301100 3.16126100 2.60472100

C -2.69371700 2.73135500 4.12090500

H -1.84315400 4.69180000 3.65548500

H -3.65484500 2.84102600 4.59965800

O -2.25996200 1.45052800 3.94894500

I -3.04664200 -0.80316700 0.49927600

N 2.12136300 -4.21698400 -2.23198600

H 1.29793300 -3.99487300 -2.77256900

C 2.44092800 -5.63402800 -2.21565400

H 1.95491300 -6.18093600 -1.39225100

H 2.12179400 -6.07737400 -3.16191500

H 3.52231000 -5.78874500 -2.12847300

H 1.88642100 -0.08127300 0.07138400

C -0.70136000 -1.90635200 -4.59602900

O -1.78901400 -1.99185700 -4.05841500

O -0.17679000 -2.87542100 -5.37761900

C -0.97406400 -4.08244800 -5.57351700

C 0.02105100 -5.18073800 -5.91489600

C -2.00369100 -3.82854900 -6.66832900

H -1.48230200 -4.30010900 -4.62906600

H 0.75811100 -5.29901800 -5.11515100

H -0.50219300 -6.13214800 -6.04823300

H 0.55376100 -4.94408100 -6.84133900

H -2.67304200 -3.01560700 -6.37779900

H -1.50389800 -3.56037300 -7.60474200

H -2.60307200 -4.72832800 -6.83858700

C -1.73958900 0.86237600 -3.99040100

O -2.40807000 1.40642200 -3.12582700

O -2.20406800 0.57094700 -5.21500900

C -3.62557000 0.78004900 -5.47499300

C -4.44335600 -0.31056900 -4.79185800

C -3.75916900 0.77607000 -6.99009600

H -3.89415800 1.76012700 -5.06623000

H -4.30416300 -0.27627400 -3.71002500

H -5.50545800 -0.16957400 -5.01713600

H -4.13024900 -1.29597800 -5.14597200

H -3.13413900 1.55323300 -7.43920900

H -3.45359900 -0.19376100 -7.39595000

H -4.79998000 0.95737200 -7.27422500

C 1.80795100 2.13244700 0.64180300

O 2.35174900 3.06323500 1.32578800

O 2.56869500 1.15907600 0.16257200

O 0.53777400 2.08634300 0.41690800

Cs -2.17657900 3.00545000 -0.35999300

Cs 5.19114900 1.54918700 1.42066900

**II** (5,6-DiCOOiPr NBE)

M06-2X single point: -2684.885268 Hartree

B3LYP-D3 optimization: -2349.021387 Hartree

B3LYP-D3 thermal correction to Gibbs free energy: 0.591371 Hartree

Pd -0.47523400 -0.82136400 0.67673700

P -0.19881900 -0.18592300 3.06666800

C 1.94909100 -2.52794300 1.42007600

H 1.81602300 -2.19900500 2.44154500

C 2.93060600 -3.48687200 1.15220700

H 3.54469900 -3.87146100 1.96470400

C 3.12137000 -3.98304900 -0.14010500

H 3.87614800 -4.74136900 -0.31804400

C 2.30851000 -3.52264400 -1.19262000

C 1.32510300 -2.53320200 -0.92214500

C 1.13379600 -2.03104800 0.38237300

C 0.42201200 -1.99641400 -1.99546400

H -0.15005700 -2.82551400 -2.43522100

C -0.54439500 -0.91355500 -1.40609200

H -1.58560500 -1.11624500 -1.65989800

C -0.05257800 0.40225800 -2.08054400

H -0.24191200 1.31923200 -1.52900100

C -0.52388200 0.47320400 -3.55691600

H -0.05555500 1.36183300 -4.00594400

C 0.17365700 -0.76480100 -4.20794200

H 0.71623400 -0.48595500 -5.11477900

C 1.18985000 -1.19576000 -3.09060500

H 2.06438400 -1.70554200 -3.49491300

C 1.43881200 0.12296200 -2.34089300

H 1.93350300 0.89716700 -2.93684700

H 1.98995300 -0.02377400 -1.41434300

C -1.06121600 -1.22792500 4.26794100

C -1.90374300 -2.28282500 4.04310400

C -2.30374700 -2.76624600 5.32852800

H -2.20946900 -2.64038500 3.07048700

C -1.67319700 -1.97385900 6.24294100

H -2.96923900 -3.59021700 5.54122600

H -1.65886600 -1.95367800 7.32228300

O -0.91208400 -1.03234600 5.62262000

C 1.45389600 0.00095900 3.86218500

C 2.34750700 1.03766100 3.87750500

C 3.54177400 0.54021600 4.50009000

H 2.17498900 2.02264500 3.47187600

C 3.29422600 -0.76346000 4.81623700

H 4.45019500 1.08980200 4.70440100

H 3.86385800 -1.53510700 5.31106400

O 2.02867700 -1.10792900 4.43725100

C -0.85139000 1.49846800 3.38304600

C -0.62059600 2.69300700 2.75497900

C -1.42652300 3.66693600 3.43234400

H 0.02141000 2.85869400 1.90098900

C -2.09125000 2.99605800 4.41616900

H -1.49358700 4.72420200 3.21627000

H -2.80668400 3.28902700 5.16915000

O -1.74597700 1.67801200 4.40696000

I -3.27960100 -0.12044100 0.96733300

N 2.46937000 -4.00403200 -2.49739300

H 1.64906200 -3.89146900 -3.07647500

C 3.19295300 -5.23752500 -2.75257600

H 2.83577100 -6.08737400 -2.14955700

H 3.08787700 -5.49258500 -3.80982300

H 4.26236100 -5.11024500 -2.54976000

H 0.86711600 1.08498100 0.56829700

C -0.73250800 -1.92536600 -4.55710400

O -1.87963500 -2.08128200 -4.18639400

O -0.05368700 -2.83646500 -5.29013600

C -0.76984500 -4.05010400 -5.67467200

C 0.29581500 -5.11582300 -5.87723900

C -1.59279400 -3.76090600 -6.92452000

H -1.43292300 -4.31982900 -4.84746300

H 0.84646000 -5.29794500 -4.94956200

H -0.17006000 -6.05586600 -6.18716800

H 1.00699600 -4.80889800 -6.65049000

H -2.31956200 -2.96986100 -6.72418500

H -0.94082900 -3.44456600 -7.74509600

H -2.13482200 -4.65908800 -7.23619600

C -1.99212200 0.71072600 -3.81916500

O -2.78309800 1.13213800 -2.99080400

O -2.28814400 0.55164200 -5.11944000

C -3.67279900 0.74897800 -5.53579200

C -4.51049800 -0.45551100 -5.12198500

C -3.61378600 0.95670600 -7.04140100

H -4.04290600 1.65052900 -5.03588800

H -4.50981800 -0.56818500 -4.03662200

H -5.54214200 -0.32326100 -5.46366500

H -4.10208000 -1.36904100 -5.56211700

H -2.97890600 1.81113300 -7.29297400

H -3.20893700 0.06604900 -7.53282500

H -4.61849500 1.14101900 -7.43319700

C 1.81524600 2.71533500 -0.04113600

O 2.98013900 3.09633400 -0.24663900

O 1.74766400 1.49439800 0.67914100

O 0.70776900 3.22862900 -0.34546400

Cs -2.31486500 3.34553500 -0.68204900

Cs 4.43248600 0.11992300 0.54373600

**Int_V (**(5,6-DiCOOiPr NBE)

M06-2X single point: -2684.89643848 Hartree

B3LYP-D3 optimization: -2349.02690405 Hartree

B3LYP-D3 thermal correction to Gibbs free energy: 0.595749 Hartree

Pd 0.04734100 0.35682900 0.19130700

P -0.15063400 -0.16760900 2.56600000

C 4.59615800 2.51048300 -1.28891300

H 5.08807800 3.45364600 -1.50725800

C 5.05341300 1.67911700 -0.27089600

H 5.91455600 1.96277600 0.32967200

C 4.41577500 0.46522400 -0.02098000

H 4.79809800 -0.18093100 0.76130500

C 3.29458300 0.04955400 -0.77707100

C 2.78428600 0.92494900 -1.78949800

C 3.47207800 2.11610000 -2.02482500

C 1.54880600 0.51335300 -2.59240900

H 1.73183800 -0.51222800 -2.93679800

C 0.14061000 0.51174700 -1.87509600

H -0.33817500 -0.43611300 -2.10603800

C -0.64400900 1.66579500 -2.55832000

H -1.40160600 2.13954600 -1.93456900

C -1.20202100 1.13402000 -3.89776400

H -1.70472000 1.95921300 -4.42391600

C 0.10705700 0.82757400 -4.70517100

H 0.10557700 1.37110900 -5.65340000

C 1.23565000 1.44979500 -3.79126900

H 2.10804700 1.72336200 -4.38752200

C 0.45880100 2.58909400 -3.09892400

H 0.08906900 3.35272400 -3.79397000

H 1.02092700 3.07056700 -2.30173400

C -1.57245800 0.60678900 3.39909300

C -2.42772000 1.56991200 2.93083700

C -3.41319000 1.77537700 3.94982900

H -2.32646400 2.08050100 1.98336200

C -3.08970100 0.92310400 4.96529500

H -4.24045500 2.47052000 3.93286400

H -3.52544700 0.72072800 5.93221500

O -1.97734200 0.20339600 4.65258700

C -0.45102400 -1.93098500 2.93695100

C 0.39115900 -2.96783600 3.22635800

C -0.40673300 -4.16012000 3.21046100

H 1.45099500 -2.88756800 3.41838400

C -1.67566300 -3.76617600 2.91460900

H -0.07568700 -5.17071600 3.40256200

H -2.61403000 -4.28877700 2.80874100

O -1.72470900 -2.41002400 2.74409200

C 1.31147400 0.16430300 3.58230400

C 2.51192500 0.70225000 3.19935300

C 3.33886600 0.72437700 4.36671400

H 2.76182900 1.03902000 2.20293000

C 2.58484000 0.20232600 5.37655600

H 4.35467200 1.08468800 4.44118700

H 2.77009200 0.02288200 6.42502000

O 1.34918000 -0.14796000 4.92514900

I 0.53846800 2.99071500 0.67116600

N 2.73107800 -1.18034400 -0.55245500

H 1.85286300 -1.48643300 -0.99625900

C 3.11142300 -2.03100600 0.55090400

H 3.03135500 -1.52597900 1.52533800

H 2.42485600 -2.88005000 0.54905800

H 4.14075600 -2.41004400 0.45843200

H 3.12278300 2.78690900 -2.80127900

C 0.41497100 -0.61195400 -5.03158400

O -0.06759400 -1.60160400 -4.50973800

O 1.38468400 -0.66575500 -5.96837400

C 1.94188300 -1.96796200 -6.29931800

C 3.32345200 -1.69299500 -6.87225900

C 1.01239100 -2.69221700 -7.26813500

H 2.03106800 -2.53246300 -5.36529000

H 3.93823200 -1.15370900 -6.14691000

H 3.82034300 -2.63585000 -7.12036100

H 3.24795300 -1.09039200 -7.78265800

H 0.01899400 -2.81413400 -6.83002900

H 0.91531700 -2.12071100 -8.19653100

H 1.41675000 -3.68014000 -7.51240600

C -2.29293600 0.09483100 -3.78819700

O -2.90214800 -0.16182300 -2.76290100

O -2.62686000 -0.41642900 -4.98745300

C -3.70428800 -1.39576900 -5.01677600

C -3.18652200 -2.74538600 -4.52973400

C -4.19493100 -1.42147400 -6.45608500

H -4.49474000 -1.03968900 -4.34796100

H -2.78212500 -2.67238900 -3.51656200

H -4.00014600 -3.47867200 -4.53576900

H -2.39940300 -3.09334200 -5.20647700

H -4.53594000 -0.43041800 -6.76787700

H -3.39068600 -1.73855700 -7.12814100

H -5.02742200 -2.12446700 -6.55635400

C -0.54033500 -2.52867200 -0.89768600

O -1.51108600 -3.31866900 -1.18301700

O 0.62812700 -2.64374300 -1.40681700

O -0.79299200 -1.56423200 -0.02293500

Cs -3.71142300 -1.27206500 0.08406700

Cs 0.06072100 -4.63047900 -3.52008100

**TS4** (5,6-DiCOOiPr NBE)

M06-2X single point: -2684.88015411 Hartree

B3LYP-D3 optimization: -2349.01035118 Hartree

B3LYP-D3 thermal correction to Gibbs free energy: 0.592283 Hartree

Imaginary frequency: -425.6702 cm^-1^

Pd 0.09185600 0.35525300 0.24300300

P -0.14607500 -0.13257600 2.61585500

C 4.74751600 2.27546900 -1.24961200

H 5.32583400 3.15670600 -1.51163800

C 5.11090900 1.46212800 -0.17979400

H 5.99180300 1.69704100 0.41509600

C 4.35674300 0.33335000 0.13710200

H 4.67170400 -0.28554400 0.96951700

C 3.19074200 -0.02975300 -0.59680500

C 2.81168700 0.82517700 -1.69430600

C 3.60279400 1.93400400 -1.98545000

C 1.57926400 0.45844400 -2.52018600

H 1.72637200 -0.57467100 -2.85329100

C 0.16901100 0.50976200 -1.82092000

H -0.34802800 -0.42307700 -2.03124300

C -0.57459000 1.67083800 -2.52771400

H -1.33781000 2.16339100 -1.92611200

C -1.11621200 1.13442100 -3.87431600

H -1.63034500 1.95211900 -4.40068100

C 0.20014200 0.84697400 -4.68113100

H 0.21489500 1.45009200 -5.59155200

C 1.32244600 1.40587400 -3.72498500

H 2.21647900 1.65866100 -4.29747800

C 0.56405000 2.56531500 -3.04627300

H 0.22882300 3.34134100 -3.74518300

H 1.13107300 3.02472300 -2.23894400

C -1.60552300 0.61901400 3.41023000

C -2.48241600 1.55386500 2.92499700

C -3.48795500 1.73592700 3.92903300

H -2.38212700 2.06406300 1.97740100

C -3.15386900 0.89963400 4.95418100

H -4.33449100 2.40692400 3.89632900

H -3.59643300 0.69021600 5.91649600

O -2.01675300 0.21150500 4.66073900

C -0.42733200 -1.89050600 3.03054800

C 0.41163400 -2.90481300 3.39716200

C -0.37021700 -4.10797000 3.38642900

H 1.46023900 -2.80283600 3.63383500

C -1.62795900 -3.74288500 3.01523900

H -0.03650100 -5.10624800 3.63126400

H -2.55387700 -4.28193800 2.88457400

O -1.68503500 -2.39436800 2.79322700

C 1.27965800 0.26872400 3.65584300

C 2.46854700 0.83819100 3.28337100

C 3.26505500 0.92726400 4.46842500

H 2.73430100 1.14352800 2.28072400

C 2.50598500 0.40982800 5.47696000

H 4.26569300 1.32545900 4.55434200

H 2.67236600 0.26988200 6.53457600

O 1.29465800 0.00059200 5.00895800

I 0.39967100 3.02040100 0.69144800

N 2.43757500 -1.12160000 -0.27904200

H 1.52406100 -1.80471200 -0.94394700

C 2.87109500 -1.96245400 0.81654900

H 2.92342600 -1.43497600 1.78190600

H 2.14295700 -2.77205700 0.92908500

H 3.86430200 -2.42322600 0.65413300

H 3.32635200 2.58406200 -2.80904700

C 0.48169300 -0.57160400 -5.09913000

O 0.13070300 -1.58554900 -4.51966500

O 1.26050700 -0.58123600 -6.20086900

C 1.74207800 -1.86406100 -6.68731900

C 2.97800800 -1.55511100 -7.51790900

C 0.63606800 -2.55808500 -7.47655700

H 2.01902800 -2.46403600 -5.81411900

H 3.72794200 -1.03863200 -6.91310300

H 3.41427400 -2.48317400 -7.89964200

H 2.71762300 -0.91842000 -8.36901200

H -0.24882200 -2.70357700 -6.85289700

H 0.35432500 -1.95158500 -8.34295900

H 0.98186500 -3.53300200 -7.83561200

C -2.18342500 0.06834900 -3.78126900

O -2.80750500 -0.20548600 -2.76899400

O -2.46826800 -0.46300400 -4.98407600

C -3.50581200 -1.48461300 -5.03768500

C -2.94727200 -2.81431700 -4.54043100

C -3.96230400 -1.52652400 -6.48780600

H -4.32354400 -1.16003700 -4.38606400

H -2.55235500 -2.72617300 -3.52516300

H -3.73730900 -3.57286400 -4.54941200

H -2.14416700 -3.14262300 -5.20790900

H -4.33761600 -0.54993000 -6.80562800

H -3.13012700 -1.80759800 -7.14168800

H -4.76174300 -2.26382000 -6.60829000

C -0.41255400 -2.55391500 -0.89341200

O -1.26541200 -3.39511600 -1.30279200

O 0.81527700 -2.57047500 -1.38884900

O -0.71227900 -1.67272400 0.00428600

Cs -3.61798500 -1.25517100 0.08603700

Cs 0.49182300 -4.57769300 -3.58703300

**V*** (5,6-DiCOOiPr NBE)

M06-2X single point: -2684.89082949 Hartree

B3LYP-D3 optimization: -2349.02284126 Hartree

B3LYP-D3 thermal correction to Gibbs free energy: 0.596554 Hartree

Pd 0.02094800 0.55712600 0.40065800

P -0.12307800 0.30898800 2.92288700

C 4.89301200 1.14791800 -1.14724300

H 5.75327900 1.72845700 -1.46799100

C 4.94143200 0.37455000 0.00815400

H 5.84841700 0.33570300 0.60715300

C 3.82000200 -0.35201100 0.41484800

H 3.87520100 -0.93162600 1.32813300

C 2.61148300 -0.31391900 -0.31584900

C 2.58053100 0.44179100 -1.52877900

C 3.71454400 1.15600200 -1.90863000

C 1.34108500 0.31383800 -2.40435500

H 1.41760700 -0.68659300 -2.84268100

C -0.05389400 0.37596000 -1.69621200

H -0.53794100 -0.59836500 -1.74095800

C -0.82033800 1.43778800 -2.50409500

H -1.69684700 1.84851300 -2.01034800

C -1.16218000 0.90183900 -3.92105900

H -1.77363300 1.65939900 -4.43145200

C 0.22845000 0.89216400 -4.65397400

H 0.22416600 1.65782200 -5.43222800

C 1.19330200 1.38419200 -3.52115600

H 2.12922400 1.74171600 -3.95021500

C 0.29949100 2.43866000 -2.83927000

H -0.00701400 3.25184000 -3.50714100

H 0.75326600 2.85849100 -1.93938100

C -1.33785600 1.12405600 4.02506600

C -2.55663900 1.70516800 3.79401400

C -3.13329500 1.98339800 5.07499100

H -2.96774100 1.93743200 2.82423000

C -2.22886600 1.55183800 5.99857000

H -4.08431000 2.45564200 5.27549900

H -2.20407200 1.56326700 7.07779900

O -1.13674600 1.01971000 5.38567100

C -0.41315500 -1.42660100 3.42969600

C 0.10895400 -2.29610200 4.34682100

C -0.49866800 -3.57063800 4.09506600

H 0.85208500 -2.05190000 5.09075600

C -1.35140600 -3.39050600 3.04693800

H -0.31411600 -4.49700200 4.62014600

H -1.99452500 -4.04424100 2.47935400

O -1.32053100 -2.09118200 2.63576400

C 1.48237000 0.68118100 3.67714900

C 2.57391600 1.26177400 3.08825200

C 3.59971800 1.31172700 4.08442200

H 2.63779500 1.58111900 2.05852200

C 3.06267300 0.75734100 5.20833300

H 4.60005600 1.70418700 3.97366700

H 3.44325600 0.57752200 6.20253200

O 1.77641500 0.36757700 4.98724000

I -1.30584400 3.01058500 0.57265400

N 1.45676300 -1.00371700 0.06919200

H 0.77433500 -2.15091000 -1.02242300

C 1.64022100 -2.00289300 1.11770600

H 1.95301200 -1.59843800 2.08926000

H 0.70009000 -2.54189700 1.23635900

H 2.40880200 -2.72925300 0.80855800

H 3.70339000 1.73620200 -2.82540600

C 0.68054400 -0.38270900 -5.31760800

O 0.59475100 -1.50792900 -4.85386500

O 1.26637300 -0.11651200 -6.50138000

C 1.83712400 -1.22924300 -7.24794700

C 2.86731800 -0.61418000 -8.18240300

C 0.72368000 -1.97590600 -7.97476500

H 2.32799700 -1.89279200 -6.52824800

H 3.62744900 -0.07064400 -7.61488000

H 3.35991200 -1.39900500 -8.76427800

H 2.38694300 0.08204700 -8.87670500

H -0.01115300 -2.35497100 -7.26208000

H 0.21643800 -1.30695900 -8.67704700

H 1.13853400 -2.81846200 -8.53731800

C -2.04372400 -0.32779000 -3.97565000

O -2.65911800 -0.78907600 -3.02871600

O -2.18167200 -0.77627500 -5.23453200

C -3.08794400 -1.89898400 -5.46499900

C -2.39276300 -3.20964400 -5.11934600

C -3.49267900 -1.79441400 -6.92772800

H -3.95583300 -1.75774800 -4.81339500

H -2.07060800 -3.24108400 -4.07561000

H -3.08111400 -4.04397900 -5.29108900

H -1.52295600 -3.34654900 -5.77014400

H -3.96990100 -0.83207900 -7.13270300

H -2.61481100 -1.89106900 -7.57507600

H -4.19656000 -2.59362200 -7.17831400

C -0.66465300 -3.49671600 -1.34827600

O -1.02762200 -4.38033600 -2.16856100

O 0.55544400 -2.90064600 -1.65510000

O -1.25562400 -3.09954500 -0.31194600

Cs -3.26768700 -0.95254800 0.09054600

Cs 1.33820100 -4.43317700 -4.13209600

**V** (5,6-DiCOOiPr NBE)

M06-2X single point: -2684.877769 Hartree

B3LYP-D3 optimization: -2349.01768 Hartree

B3LYP-D3 thermal correction to Gibbs free energy: 0.586927 Hartree

Pd 1.16559000 0.00574100 1.09235700

P 0.86667600 0.34616900 3.49844300

C 6.07662400 -1.11037800 -0.46961100

H 7.09441800 -0.90811000 -0.78900200

C 5.82111300 -1.87808500 0.66149000

H 6.64204200 -2.29396800 1.24032600

C 4.50619700 -2.11658100 1.07114800

H 4.33388500 -2.71760000 1.95627100

C 3.40632500 -1.58700800 0.35850600

C 3.67464600 -0.81463100 -0.81582800

C 4.99337600 -0.60009700 -1.20181500

C 2.49244400 -0.36945100 -1.67564200

H 2.26112300 -1.24251300 -2.29509700

C 1.20650100 0.06146100 -0.92745200

H 0.34180400 -0.54587700 -1.17697200

C 0.99002800 1.53606400 -1.28943700

H 0.40580400 2.13449000 -0.58280900

C 0.37771800 1.59401700 -2.70821300

H 0.21089500 2.64567200 -2.96941900

C 1.54752900 1.04167900 -3.60210100

H 1.84193800 1.78511500 -4.34477500

C 2.73108900 0.87795600 -2.57173200

H 3.69542600 0.90852300 -3.07851200

C 2.42354200 2.01887200 -1.58195300

H 2.46580700 3.01744000 -2.02728500

H 3.07438000 1.98574100 -0.70325200

C -0.30311900 -0.73975100 4.36298200

C -1.19049800 -1.65090000 3.84850500

C -1.90664800 -2.18855700 4.96671500

H -1.32375600 -1.89940600 2.79974900

C -1.40354000 -1.57459800 6.07614000

H -2.68582000 -2.93691500 4.94211800

H -1.61443100 -1.65652000 7.13186300

O -0.42962700 -0.68752000 5.73422200

C 2.41061000 0.24710800 4.44388600

C 3.67201600 -0.04949000 3.99606800

C 4.53530400 0.02015100 5.13476700

H 3.94093000 -0.29903800 2.97872400

C 3.74099600 0.35605100 6.19139500

H 5.60104300 -0.15502400 5.16063500

H 3.93279600 0.52480400 7.24031000

O 2.44665200 0.49949100 5.79549200

C 0.21314500 2.01655300 3.80005100

C -0.03781400 3.02768900 2.90647900

C -0.60387700 4.10800200 3.65478500

H 0.14086800 3.01950300 1.83912600

C -0.65621600 3.68253100 4.94929200

H -0.92749700 5.06246900 3.26624200

H -0.99698000 4.13650600 5.86761700

O -0.16679400 2.41518000 5.06154000

I -1.66086700 4.40763500 -0.61041100

N 2.07774000 -1.83235300 0.70973200

H -4.57743200 -2.99613100 0.90172600

C 1.83947300 -2.73269700 1.83065800

H 2.24968600 -2.38858500 2.79448100

H 0.76205600 -2.86692300 1.94811800

H 2.28415300 -3.71916500 1.62073400

H 5.20143400 -0.02287800 -2.09714800

C 1.26398100 -0.24866200 -4.33755700

O 0.52870000 -1.14397100 -3.94273800

O 1.98257900 -0.32638500 -5.46464600

C 1.91583100 -1.56953700 -6.23342900

C 2.86270800 -2.58671200 -5.60665800

C 2.28054400 -1.19148700 -7.65958800

H 0.88529700 -1.93348500 -6.18088600

H 2.57125000 -2.79998100 -4.57521400

H 2.83871300 -3.52165000 -6.17490900

H 3.88797200 -2.20392100 -5.60954000

H 1.58922500 -0.43888400 -8.04800500

H 3.29671400 -0.78754400 -7.70286000

H 2.23111800 -2.07520000 -8.30254400

C -0.97527400 0.93991300 -2.89145600

O -1.64316700 0.40912500 -2.01871900

O -1.37699700 1.06345300 -4.16470600

C -2.63346200 0.42626100 -4.54773600

C -2.48193700 0.08008400 -6.02064500

C -3.78393700 1.37829700 -4.24688100

H -2.73428000 -0.48379800 -3.94849800

H -1.62419500 -0.58234600 -6.16888700

H -3.38266000 -0.42565600 -6.38130700

H -2.33130200 0.98628400 -6.61558800

H -3.80909200 1.61947400 -3.18152900

H -3.66752100 2.30794100 -4.81238100

H -4.73674600 0.91692700 -4.52570800

C -2.71450600 -2.87033200 0.52045000

O -1.69111300 -2.16851200 0.73520300

O -2.84376600 -3.90021100 -0.17235300

O -3.87269500 -2.38141400 1.15243100

Cs -2.83175300 0.79883300 0.92275300

Cs -0.03295400 -3.42339800 -1.63873100

**TS2** (5,6-DiCOOiPr NBE)

M06-2X single point: -2684.841741 Hartree

B3LYP-D3 optimization: -2348.981176 Hartree

B3LYP-D3 thermal correction to Gibbs free energy: 0.584856 Hartree

Imaginary frequency: -310.5202 cm^-1^

Pd 1.30719800 0.08116600 1.30091700

P 0.84803300 0.66620100 3.46672400

C 6.23677900 -1.44595400 -0.53118800

H 7.28323700 -1.34952500 -0.80340800

C 5.86553900 -2.15178000 0.61395100

H 6.62911700 -2.61156100 1.23629400

C 4.52103300 -2.27061600 0.98654300

H 4.24207100 -2.81280800 1.88446000

C 3.53074200 -1.65846000 0.19700300

C 3.90693600 -0.99008000 -0.98670700

C 5.24143900 -0.87760900 -1.34416100

C 2.70285900 -0.60771000 -1.82717500

H 2.53708200 -1.43440300 -2.52679800

C 1.45756300 -0.43887000 -0.93324200

H 0.60293300 -1.05494700 -1.16248700

C 1.11768500 1.08844400 -1.08465700

H 0.52275400 1.58236100 -0.30784700

C 0.42778700 1.26594700 -2.46803000

H 0.18830500 2.33310900 -2.54813000

C 1.56737200 0.94676800 -3.49427100

H 1.74880200 1.80129400 -4.14865500

C 2.80889400 0.75221500 -2.55249500

H 3.75437300 0.90080800 -3.07573400

C 2.48502700 1.72322300 -1.40213900

H 2.40379100 2.76863400 -1.71309500

H 3.19960000 1.64599400 -0.57890000

C -0.35932300 -0.43235500 4.28465300

C -1.08204100 -1.47430600 3.76097700

C -1.92321100 -1.95908000 4.81382300

H -1.04471900 -1.83864100 2.74156900

C -1.64906600 -1.18805000 5.90475600

H -2.63405500 -2.77025900 4.75276900

H -2.01880200 -1.17544000 6.91924400

O -0.70395400 -0.25362600 5.60741900

C 2.32827900 0.55536600 4.52386800

C 3.58353900 0.10873900 4.19967200

C 4.37029100 0.19466900 5.39211900

H 3.89444400 -0.24192300 3.22485600

C 3.54124700 0.68708200 6.35638000

H 5.41075700 -0.07054000 5.51285500

H 3.67663900 0.92762200 7.40015000

O 2.29661100 0.91156300 5.85263800

C 0.17068300 2.32646900 3.79165900

C -0.16970600 3.30254600 2.88961500

C -0.71024200 4.39162300 3.64510500

H -0.06430400 3.25483500 1.81402900

C -0.65944000 4.00588800 4.95217600

H -1.08210000 5.32928400 3.25815600

H -0.94270200 4.48214500 5.87895500

O -0.13079000 2.75549800 5.06609200

I -1.68809000 4.03746500 -0.85537800

N 2.17374800 -1.69549100 0.44251000

H -4.32112900 -3.30616100 1.61083800

C 1.56442300 -2.84898400 1.08515600

H 1.75744100 -2.87995000 2.16930900

H 0.48013500 -2.81513200 0.95307500

H 1.95439300 -3.78184300 0.64288000

H 5.51957500 -0.37149300 -2.26530300

C 1.33400800 -0.26324700 -4.37051200

O 0.64654800 -1.23070400 -4.06812400

O 2.03026400 -0.17663600 -5.50733800

C 1.99662500 -1.31985300 -6.42257300

C 2.97112000 -2.38069800 -5.92456700

C 2.34785000 -0.75865900 -7.79048200

H 0.97566100 -1.71365800 -6.41581900

H 2.68140900 -2.73563700 -4.93252700

H 2.97763700 -3.23349300 -6.61028700

H 3.98420000 -1.97030400 -5.87111100

H 1.63743100 0.01892700 -8.08341800

H 3.35370900 -0.32772600 -7.78269200

H 2.31874900 -1.55715500 -8.53763100

C -0.90236300 0.58884700 -2.69084800

O -1.50077500 -0.09876300 -1.87633300

O -1.37180600 0.88724900 -3.90798600

C -2.65262700 0.31792300 -4.31739100

C -2.54427700 0.10297700 -5.81880700

C -3.76532300 1.26963600 -3.89945300

H -2.76923200 -0.63926100 -3.80094400

H -1.70697700 -0.56280000 -6.04794100

H -3.46623100 -0.34563800 -6.20078300

H -2.38364100 1.05581000 -6.33241900

H -3.74998900 1.42359000 -2.81772600

H -3.63921300 2.24172600 -4.38505400

H -4.73866900 0.85777200 -4.18486300

C -2.65977600 -3.07805600 0.70716300

O -1.66470800 -2.31193700 0.61394500

O -2.91630200 -4.12917500 0.08396800

O -3.61649400 -2.64281400 1.64065900

Cs -2.80898100 0.61687000 1.05993400

Cs -0.75399400 -3.44589300 -2.19062800

**1b (by-product)** (5,6-DiCOOiPr NBE)

M06-2X single point: -2684.937608 Hartree

B3LYP-D3 optimization: -2349.0693 Hartree

B3LYP-D3 thermal correction to Gibbs free energy: 0.590915 Hartree

Pd 1.84300900 -0.30726100 2.04158100

P 1.01650700 0.51609800 3.92212400

C 6.48294800 -1.73475100 -0.76597800

H 7.53209800 -1.79863100 -1.03841300

C 6.08871800 -1.99803700 0.55210000

H 6.83461400 -2.26512000 1.29481300

C 4.74543000 -1.91165000 0.92817000

H 4.43137700 -2.09488300 1.95126100

C 3.81173600 -1.55903900 -0.04444100

C 4.19088400 -1.30293100 -1.36185000

C 5.53286100 -1.38300700 -1.72997400

C 2.98908200 -0.93399000 -2.19236300

H 2.81528800 -1.67043600 -2.98006800

C 1.83757800 -0.87144600 -1.12717400

H 0.95591000 -1.43885000 -1.40412100

C 1.49218500 0.63790600 -1.06666500

H 1.02964900 0.93159800 -0.12259300

C 0.68201100 1.04637400 -2.31550300

H 0.48741400 2.12254200 -2.23632400

C 1.69089400 0.82814800 -3.49933400

H 1.81231100 1.74193400 -4.08299900

C 3.02579000 0.51794100 -2.73105100

H 3.91659900 0.74174500 -3.32118200

C 2.82960400 1.32043800 -1.42475000

H 2.72733800 2.39752100 -1.58303800

H 3.62217300 1.14614500 -0.69402400

C -0.33562700 -0.51943400 4.59532000

C -1.00693900 -1.56030500 4.00432300

C -2.05347500 -1.93970100 4.90488800

H -0.80739100 -1.98717000 3.02964600

C -1.94109900 -1.11457800 5.98475500

H -2.78547400 -2.71838900 4.74965300

H -2.48467800 -1.02582700 6.91365900

O -0.90803600 -0.24284800 5.81781700

C 2.13405200 0.73982100 5.34636700

C 3.46721000 0.44202300 5.44958600

C 3.87474100 0.82040700 6.76838300

H 4.07137500 0.00860200 4.66539200

C 2.76215600 1.32491200 7.37417300

H 4.85972400 0.73213700 7.20371500

H 2.57236400 1.73508600 8.35469100

O 1.69485100 1.28600900 6.53050300

C 0.19201100 2.14129600 3.76443900

C 0.14321500 2.98603300 2.68488400

C -0.71942800 4.07105200 3.04164900

H 0.64646600 2.84032900 1.74010200

C -1.13634200 3.81401000 4.31421900

H -0.99882300 4.90459300 2.41430200

H -1.78632500 4.33350800 5.00248000

O -0.59783600 2.64789700 4.77347800

I -1.64784200 3.90656900 -0.92751000

N 2.38140200 -1.45403200 0.15512100

H -4.15278100 -3.30973500 1.79258600

C 1.80205000 -2.81023600 0.40247100

H 2.25056100 -3.23053000 1.30356200

H 0.72388300 -2.72964400 0.55197700

H 2.02787600 -3.48217500 -0.44197400

H 5.83577200 -1.17293400 -2.75226000

C 1.29499600 -0.28097100 -4.45167200

O 0.69630600 -1.30068600 -4.12823500

O 1.71548400 -0.03304500 -5.69467100

C 1.45146400 -1.04014800 -6.72470400

C 2.51492900 -2.12788700 -6.63697100

C 1.45102300 -0.28491100 -8.04350600

H 0.46306800 -1.46276200 -6.52193900

H 2.47877100 -2.62311400 -5.66353200

H 2.34699000 -2.87879500 -7.41510400

H 3.51101500 -1.69753700 -6.77955500

H 0.69092600 0.50095700 -8.03774700

H 2.42723700 0.17526900 -8.22449900

H 1.23464800 -0.97362600 -8.86545800

C -0.69581400 0.46683500 -2.50832400

O -1.27733000 -0.26198400 -1.71580300

O -1.21813800 0.90542500 -3.65891600

C -2.56519300 0.50018600 -4.04989800

C -2.42549400 -0.30821500 -5.33425700

C -3.38673100 1.77115200 -4.20429100

H -2.97164000 -0.11935600 -3.24655900

H -1.77181700 -1.17115500 -5.18112600

H -3.40688600 -0.65634700 -5.67089700

H -1.98771700 0.31049400 -6.12446400

H -3.38126500 2.34318100 -3.27249700

H -2.96941500 2.40147200 -4.99596000

H -4.41881500 1.51857100 -4.46825300

C -2.50519900 -3.17915700 0.84601600

O -1.45587600 -2.49499700 0.74476100

O -2.87267100 -4.18077200 0.19345600

O -3.39889700 -2.70305000 1.82305000

Cs -2.44250600 0.51611500 1.24123600

Cs -1.15759800 -3.44772900 -2.35664900

**Int_I(a)** (unsubstituted NBE)

M06-2X single point: -2071.927353 Hartree

B3LYP-D3 optimization: -1735.945678 Hartree

B3LYP-D3 thermal correction to Gibbs free energy: 0.413907 Hartree

Pd -0.11656500 -0.37652500 -0.29263600

P 0.96521400 0.54833800 -2.09031300

C -2.76157700 1.18111900 -1.33846500

C -3.00495800 2.29355100 -2.17982700

C -2.61467300 1.42099000 0.03855500

C -3.07981000 3.58561000 -1.63470600

C -2.68349200 2.71216300 0.56777000

H -2.42863900 0.60175900 0.73100400

C -2.91269800 3.80479100 -0.26931800

H -3.26891000 4.42496800 -2.30074600

H -2.55381500 2.85730800 1.63686000

H -2.96409300 4.81398400 0.13081500

C -1.05860900 -1.28453500 2.41868900

O -0.46115100 -1.75671800 3.44723600

O -0.61177000 -1.70260200 1.22366100

O -2.02886600 -0.45918300 2.47308500

Cs -0.83504800 1.12136400 4.71035000

C -2.72012700 -0.22495200 -1.91993800

C -3.97809500 -1.07241800 -1.52662300

C -2.27253400 -2.46042500 -1.01179900

C -1.55131800 -1.21180700 -1.54864300

C -3.49371400 -1.86406400 -0.29258600

H -4.22011400 -2.62545900 0.01944100

H -3.21236800 -1.26500000 0.57420300

C -4.13541700 -2.20187300 -2.57193600

H -5.09484700 -2.71437600 -2.44056400

H -4.10427100 -1.82576400 -3.60073400

C -2.93883900 -3.15137200 -2.23705700

H -3.29294500 -4.15179700 -1.96187000

H -2.24629200 -3.26947300 -3.07816200

H -4.87576900 -0.46185000 -1.39209600

H -1.64060800 -3.11633200 -0.41472900

H -0.98315300 -1.46784300 -2.44415400

H -2.68809400 -0.10089000 -3.00373300

Cs 2.14572200 -2.75407300 1.80864400

I 1.77213900 1.30677300 1.51222900

C 2.75687600 0.19746400 -2.00028300

C 3.87282400 0.89371400 -2.37360700

O 3.12815600 -0.98450700 -1.40026100

C 5.00023200 0.10243000 -1.98187900

H 3.87800500 1.85689300 -2.86177500

C 4.49160800 -1.02118200 -1.40206100

H 6.04580800 0.34116400 -2.11401900

H 4.94192500 -1.90355000 -0.97286200

C 0.54145500 -0.06143900 -3.76386700

C -0.44966400 0.28077600 -4.64184800

O 1.17140600 -1.20690200 -4.19065500

C -0.42103000 -0.70450300 -5.68339400

H -1.15467500 1.09402200 -4.52095200

C 0.57429700 -1.57739300 -5.36147500

H -1.05982900 -0.75183300 -6.55379300

H 0.97788900 -2.45852100 -5.83624500

C 0.89801300 2.33625500 -2.33461200

C 0.27081700 3.29325400 -1.58949000

O 1.53079000 2.92107100 -3.41403300

C 0.52452500 4.54372400 -2.23677300

H -0.30542200 3.11169100 -0.69567300

C 1.29078400 4.25948900 -3.32882900

H 0.17704700 5.51951700 -1.93005100

H 1.72486400 4.86466300 -4.11046600

N -3.14654000 2.13389100 -3.59611100

H -2.95715000 3.02630200 -4.04271300

C -4.46312300 1.65618500 -4.04697200

H -5.29310900 2.30019800 -3.71607000

H -4.47034300 1.60760200 -5.14000900

H -4.64728400 0.65058200 -3.66389600

**TS1(a)** (unsubstituted NBE)

M06-2X single point: -2071.885687 Hartree

B3LYP-D3 optimization: -1735.90829 Hartree

B3LYP-D3 thermal correction to Gibbs free energy: 0.408244 Hartree

Imaginary frequency: -1452.3115 cm^-1^

Pd -0.41257300 0.44473300 -0.61605700

P 1.25598800 0.37208100 -2.20241000

C -3.14800200 1.53382800 -0.78675800

C -3.86177200 2.75569900 -0.86493200

C -2.31465400 1.26614100 0.33419600

C -3.74496700 3.69173000 0.17451100

C -2.20648400 2.25458800 1.33492600

H -2.11417400 0.05390500 0.94464300

C -2.90808300 3.45433500 1.26477600

H -4.30586600 4.62278900 0.11184700

H -1.52676600 2.08054200 2.16210000

H -2.80982600 4.20460200 2.04636200

C -1.08856900 -1.70400300 1.67808100

O -0.56790600 -2.28440900 2.68954200

O -0.67785500 -1.88375000 0.47781900

O -2.09109600 -0.83998000 1.87696700

Cs -1.10768700 0.02643300 4.62693300

C -3.12398800 0.59147700 -1.96426000

C -4.08720000 -0.63896500 -1.92019400

C -2.05164000 -1.59123200 -2.19391600

C -1.73391000 -0.08825300 -2.15216400

C -3.23593600 -1.71106900 -1.21713300

H -3.70765900 -2.70181500 -1.24678500

H -2.96721700 -1.47559400 -0.19135400

C -4.17248700 -1.21209400 -3.35612400

H -4.96146500 -1.96948400 -3.42076400

H -4.39770300 -0.44166700 -4.10240300

C -2.75797100 -1.85508700 -3.55655200

H -2.83475600 -2.93354500 -3.73774400

H -2.20853100 -1.42319700 -4.39956700

H -5.05565200 -0.42190600 -1.46180900

H -1.20040700 -2.23954900 -1.99545700

H -1.26666900 0.25233800 -3.07842700

H -3.34610300 1.19308200 -2.85421300

Cs 2.12575100 -2.74068300 0.87525900

I 1.48062500 1.34812800 1.53799600

C 3.02992200 0.57182400 -1.81098200

C 3.91731700 1.59182200 -2.01038900

O 3.63155800 -0.43045900 -1.08693500

C 5.13959900 1.20166200 -1.37076500

H 3.72000100 2.50898100 -2.54497500

C 4.91306800 -0.02632400 -0.82937300

H 6.06088800 1.76420700 -1.32516700

H 5.52258400 -0.71863700 -0.26913300

C 1.24978300 -1.15484300 -3.21329600

C 0.93913500 -1.43328900 -4.51506200

O 1.42607800 -2.33249800 -2.52022700

C 0.92634500 -2.86229900 -4.63826300

H 0.74189900 -0.70448100 -5.28673100

C 1.22811700 -3.35492200 -3.40585800

H 0.71589400 -3.43918900 -5.52721700

H 1.33916300 -4.35157900 -3.00706400

C 1.03823600 1.65614300 -3.46309600

C 0.13530800 2.68452200 -3.50419300

O 1.84197200 1.70022900 -4.58293900

C 0.39073100 3.40553900 -4.71286100

H -0.61943600 2.87982100 -2.75527600

C 1.42990600 2.76661100 -5.32316500

H -0.12945900 4.27976600 -5.07625600

H 1.96969800 2.93681300 -6.24256200

N -4.62118700 3.10778100 -2.01862100

H -4.83558700 4.09884200 -1.97935700

C -5.86229900 2.36713600 -2.27623400

H -6.52026800 2.29919700 -1.39492700

H -6.40900900 2.87160600 -3.07844100

H -5.64470100 1.35358700 -2.61370400

**II(a)** (unsubstituted NBE)

M06-2X single point: -2071.919655 Hartree

B3LYP-D3 optimization: -1735.944588 Hartree

B3LYP-D3 thermal correction to Gibbs free energy: 0.409869 Hartree

Pd -0.43859400 0.87305200 -0.82754200

P 1.41752700 0.34526100 -2.30682000

C -3.29811200 1.71782800 -0.84436600

C -4.38069500 2.57949200 -0.52282000

C -2.13064200 1.71611500 -0.03888100

C -4.32655400 3.31604800 0.67491900

C -2.09868400 2.50306300 1.12635100

H -2.36559900 -0.32584700 1.35710000

C -3.19864900 3.28033100 1.49151300

H -5.16882400 3.95309500 0.94039300

H -1.18988900 2.54621800 1.71646400

H -3.17056300 3.88477200 2.39698800

C -1.21454800 -1.67527600 2.12721500

O -0.54367200 -1.89318300 3.16684100

O -1.15012100 -2.23886100 1.01041300

O -2.14254600 -0.62726800 2.25351500

Cs -0.82884700 0.91242900 4.57673200

C -3.24153500 0.87946500 -2.08732200

C -4.05927900 -0.45912800 -2.06107900

C -1.95882400 -1.16484800 -2.49242200

C -1.78076600 0.36056900 -2.34084900

C -3.06376800 -1.46608400 -1.46817600

H -3.42967000 -2.49911800 -1.52122100

H -2.75176800 -1.26775700 -0.44532800

C -4.16646000 -0.96747600 -3.51885200

H -4.86452000 -1.80956900 -3.58262500

H -4.52409300 -0.19343800 -4.20732600

C -2.70287400 -1.42602700 -3.83261900

H -2.66536400 -2.49091000 -4.09016800

H -2.25342800 -0.87440200 -4.66527500

H -5.01345000 -0.38879500 -1.53883600

H -1.04673800 -1.74946200 -2.38766700

H -1.35761300 0.80795600 -3.24638000

H -3.58528600 1.48700900 -2.93796400

Cs 1.84429100 -2.58149300 0.90477300

I 1.59230400 1.58949000 1.30998400

C 3.19738000 0.40584800 -1.86594300

C 4.18788500 1.31692800 -2.10176100

O 3.67452800 -0.59219200 -1.04782100

C 5.34448000 0.86111900 -1.38664600

H 4.10088200 2.20557300 -2.70878100

C 4.98136500 -0.29516200 -0.76828000

H 6.31463400 1.33489700 -1.34472000

H 5.50079500 -0.99937700 -0.13660600

C 1.33319600 -1.22573700 -3.24863800

C 1.09057500 -1.55334300 -4.55291800

O 1.33799500 -2.37290000 -2.48130500

C 0.93956700 -2.97910400 -4.59978300

H 1.02584600 -0.85797300 -5.37594200

C 1.09983500 -3.42447900 -3.32406400

H 0.73645400 -3.58545100 -5.47070600

H 1.07971100 -4.40104200 -2.86517800

C 1.39414000 1.56686300 -3.64882400

C 0.64456700 2.70453500 -3.78472200

O 2.24262200 1.44231500 -4.72935300

C 1.04587900 3.31707400 -5.01381600

H -0.10296000 3.04507800 -3.08242500

C 2.01077500 2.51073500 -5.54128700

H 0.66537900 4.23189200 -5.44423800

H 2.60329600 2.55277900 -6.44277500

N -5.44913600 2.87030200 -1.41074300

H -5.99189700 3.63414600 -1.02533700

C -6.35984300 1.84775000 -1.92626900

H -6.72694600 1.15376000 -1.15358300

H -7.22397100 2.35682900 -2.36307000

H -5.89473000 1.26135100 -2.71876000

**Int_V(a)**

M06-2X single point: -2071.91424476 Hartree

B3LYP-D3 optimization: -1735.93065435 Hartree

B3LYP-D3 thermal correction to Gibbs free energy: 0.414831 Hartree

Pd 0.11065600 0.49937700 0.35823200

P -0.06542300 0.19350900 2.88172400

C 4.75468000 1.60594500 -1.19199000

H 5.51098700 2.32121600 -1.50181000

C 4.89803800 0.89341900 -0.00612200

H 5.77163700 1.03701500 0.62368500

C 3.90847900 -0.01394600 0.38451100

H 4.02349700 -0.54913900 1.31906000

C 2.76762700 -0.20215700 -0.40436300

C 2.62051000 0.49868700 -1.62638500

C 3.62511500 1.39431400 -1.99257300

C 1.39317400 0.20605500 -2.47052000

H 1.48261200 -0.84617300 -2.77553000

C 0.00808100 0.31236600 -1.73875200

H -0.47288000 -0.66806400 -1.70010900

C -0.79360900 1.26836300 -2.63261900

H -1.66312600 1.71954800 -2.15852100

C -1.15158100 0.45301600 -3.91187400

H -1.91043700 0.98434800 -4.49916100

H -1.53460900 -0.53907400 -3.65634700

C 0.20832600 0.38140400 -4.67135000

H 0.15805700 0.90836400 -5.63072000

H 0.52514500 -0.64663300 -4.87550100

C 1.18003300 1.10925400 -3.71408600

H 2.10223200 1.41835000 -4.21045800

C 0.28685600 2.23456900 -3.14990600

H -0.07329800 2.92571500 -3.92085400

H 0.75904700 2.80421200 -2.34403500

C -1.42310400 0.84492500 3.91552000

C -2.60842200 1.45793100 3.60450500

C -3.33162000 1.60662400 4.83139500

H -2.90261100 1.79126600 2.62106200

C -2.53973100 1.07374300 5.80448200

H -4.30241200 2.06118900 4.96720500

H -2.64470500 0.96839500 6.87379400

O -1.38060700 0.59938300 5.27260900

C -0.16399900 -1.57862100 3.33240000

C 0.42446500 -2.42046400 4.23606100

C -0.00310600 -3.74823200 3.89650200

H 1.09179400 -2.12197700 5.03070100

C -0.82245000 -3.62662000 2.81156700

H 0.26602300 -4.66735000 4.39803100

H -1.34276000 -4.31381800 2.16435500

O -0.93574800 -2.31485700 2.46282500

C 1.45494500 0.71618000 3.71800700

C 2.51624700 1.40070400 3.18721200

C 3.47622300 1.54851000 4.23816700

H 2.59809000 1.73859000 2.16423600

C 2.93326300 0.94525300 5.33379800

H 4.43682000 2.03965000 4.18288600

H 3.27287800 0.80526400 6.34902000

O 1.70469800 0.43251700 5.04382100

I -1.16773600 2.90009800 0.46040900

N 1.68889600 -1.04776100 0.01509000

H 1.29463700 -1.67379900 -0.76366000

C 1.99478200 -2.00569900 1.08364000

H 2.19181400 -1.51016600 2.03360400

H 1.12548600 -2.65444900 1.17594400

H 2.88129300 -2.60279400 0.82304500

H 3.52834400 1.95535800 -2.91485400

C -0.58375600 -3.06882800 -1.31243600

O -0.90602600 -3.72296400 -0.23463100

O 0.70124800 -2.90483500 -1.59082100

O -1.47806000 -2.56218800 -2.07856000

Cs -3.11716200 -1.32669900 0.10643700

Cs 1.46739800 -5.55468800 -0.34531000

**TS4(a)**

M06-2X single point: -2071.90814777 Hartree

B3LYP-D3 optimization: -1735.92820931 Hartree

B3LYP-D3 thermal correction to Gibbs free energy: 0.410704 Hartree

Imaginary frequency: -962.2845 cm-1

Pd 0.10563200 0.46162400 0.32976000

P -0.08646100 0.16648200 2.86569100

C 4.79805800 1.52882600 -1.21524800

H 5.57287400 2.22361700 -1.52629300

C 4.93159000 0.79090900 -0.04452200

H 5.81877700 0.89346200 0.57534400

C 3.91640600 -0.08650400 0.34856700

H 4.03400000 -0.63491000 1.27503600

C 2.74492100 -0.23138600 -0.41657900

C 2.62259800 0.49349400 -1.63454400

C 3.64966700 1.36109900 -2.00106700

C 1.39727900 0.23346800 -2.49238500

H 1.49987500 -0.80378900 -2.83812600

C 0.00953700 0.30934100 -1.76744300

H -0.46663300 -0.67079500 -1.77193200

C -0.79403800 1.29394200 -2.63249200

H -1.66588600 1.72791500 -2.14684600

C -1.14426300 0.53235900 -3.94462900

H -1.89465100 1.08943300 -4.51893700

H -1.54141300 -0.46584800 -3.73713800

C 0.22243700 0.48226400 -4.69427400

H 0.17897000 1.03892000 -5.63708900

H 0.54052600 -0.53889800 -4.92930300

C 1.18675600 1.17833300 -3.70598700

H 2.10837500 1.50905100 -4.18861800

C 0.28719400 2.28046400 -3.10798800

H -0.06997800 2.99911500 -3.85501400

H 0.75309600 2.82067300 -2.27883400

C -1.44710900 0.81477200 3.90186800

C -2.64717700 1.40317200 3.59969700

C -3.35887000 1.55097800 4.83344900

H -2.95839400 1.72405600 2.61755800

C -2.54643900 1.04182700 5.80218800

H -4.33578400 1.99009200 4.97611700

H -2.63672700 0.94547000 6.87369700

O -1.38577900 0.58282100 5.26100300

C -0.17331200 -1.59474500 3.36744400

C 0.47621100 -2.41578700 4.24685600

C 0.01658000 -3.74932900 3.97835900

H 1.20266500 -2.10058700 4.98076400

C -0.88275100 -3.64937600 2.95727400

H 0.31392700 -4.65640500 4.48590000

H -1.46679900 -4.35685900 2.39117200

O -1.01536300 -2.34721600 2.57941400

C 1.43110300 0.71968000 3.68745100

C 2.49442000 1.38118900 3.13203800

C 3.45280900 1.56788700 4.17801800

H 2.58094100 1.67451600 2.09570000

C 2.90721100 1.00848800 5.29544300

H 4.41437100 2.05478700 4.10486300

H 3.24465100 0.90837500 6.31611400

O 1.67843700 0.48627200 5.02376300

I -1.22243900 2.86301500 0.48348900

N 1.66127400 -1.05691600 -0.01545200

H 1.18441900 -1.92613500 -0.89157000

C 1.95692700 -1.95491900 1.09969100

H 2.18795700 -1.43632800 2.03361900

H 1.08318700 -2.58948300 1.24520300

H 2.83048300 -2.58606500 0.86272900

H 3.56205000 1.93297700 -2.91791700

C -0.54480900 -3.06831700 -1.28272900

O -0.79784300 -3.64244000 -0.16320600

O 0.76648100 -2.81898800 -1.57124700

O -1.42621600 -2.70936300 -2.11430600

Cs -3.08642200 -1.32802500 0.07985000

Cs 1.59603700 -5.48312100 -0.16172800

**V*(a)**

M06-2X single point: -2071.90881698 Hartree

B3LYP-D3 optimization: -1735.93186898 Hartree

B3LYP-D3 thermal correction to Gibbs free energy: 0.412083 Hartree

Pd 0.11393200 0.44917000 0.31230400

P -0.08344700 0.14608600 2.84768000

C 4.82387800 1.59164700 -1.21371700

H 5.59515500 2.28993600 -1.52588100

C 4.96976300 0.83812700 -0.05441300

H 5.86562800 0.93242200 0.55508600

C 3.95858100 -0.04181300 0.34426400

H 4.08903800 -0.60025100 1.26353800

C 2.76956800 -0.17864900 -0.40357400

C 2.64784600 0.55338400 -1.62284100

C 3.66736600 1.42631400 -1.99118400

C 1.43541100 0.26234000 -2.49058100

H 1.57868800 -0.76824500 -2.84344500

C 0.03856200 0.29560500 -1.78182300

H -0.41448200 -0.69490600 -1.80731100

C -0.78334900 1.26906300 -2.64640300

H -1.66600400 1.68311900 -2.16276000

C -1.10926500 0.51731600 -3.96851900

H -1.85755700 1.07270900 -4.54743100

H -1.50350900 -0.48614300 -3.77882500

C 0.26752300 0.48987400 -4.70159600

H 0.22448800 1.04318300 -5.64635000

H 0.60640200 -0.52591400 -4.93113400

C 1.20884100 1.20429700 -3.70346600

H 2.12502400 1.55945500 -4.17877900

C 0.28024700 2.28213100 -3.10565400

H -0.08718800 2.99842500 -3.85002600

H 0.72782700 2.82597500 -2.26897500

C -1.44091800 0.78472000 3.89755700

C -2.63949200 1.38532400 3.61420200

C -3.34272200 1.51513700 4.85484800

H -2.95480300 1.72617200 2.64023600

C -2.52747500 0.98378200 5.80911100

H -4.31608000 1.95749500 5.01126600

H -2.61182900 0.86838100 6.87924300

O -1.37324400 0.52781900 5.25248400

C -0.17927700 -1.61234600 3.36601400

C 0.52636600 -2.44669700 4.18674700

C -0.02244000 -3.76136700 4.00543100

H 1.34095400 -2.15235700 4.83113000

C -1.02763100 -3.63522400 3.09181600

H 0.28080000 -4.67068900 4.50570000

H -1.72083300 -4.32588900 2.63836200

O -1.13933100 -2.33654800 2.69452900

C 1.43419100 0.69890400 3.66951200

C 2.49025700 1.37392600 3.11678300

C 3.44728300 1.56479400 4.16301100

H 2.57583000 1.66869800 2.08079400

C 2.90837200 0.99387700 5.27793800

H 4.40425400 2.06082300 4.09110400

H 3.24790700 0.89136000 6.29770000

O 1.68474600 0.46062900 5.00469300

I -1.29623700 2.83038100 0.46598400

N 1.69899500 -1.00351900 -0.01938000

H 1.08698300 -2.18419100 -1.09063600

C 1.98880600 -1.89618500 1.09123900

H 2.25156900 -1.39585000 2.03221900

H 1.10538500 -2.51193400 1.26382200

H 2.84571700 -2.55040300 0.84408300

H 3.57443300 1.99961300 -2.90714200

C -0.63900600 -3.09954500 -1.31945400

O -0.83227900 -3.47861100 -0.12150400

O 0.69701400 -2.90461900 -1.69388300

O -1.50985100 -2.88285900 -2.18873000

Cs -3.11102400 -1.24778900 0.04257400

Cs 1.43079800 -5.46724000 0.20409700

**V(a)** (unsubstituted NBE)

M06-2X single point: -2071.920539 Hartree

B3LYP-D3 optimization: -1735.938141 Hartree

B3LYP-D3 thermal correction to Gibbs free energy: 0.407383 Hartree

Pd -0.33367100 1.23977200 0.72375100

P 0.12915600 1.25657300 3.18839500

C 4.59831500 -0.38000900 -0.34750400

H 5.66663600 -0.26874200 -0.51060600

C 4.10752700 -1.25502100 0.61901600

H 4.78956200 -1.84484500 1.22705600

C 2.73094200 -1.35930300 0.83921600

H 2.37577500 -2.02007600 1.62077300

C 1.80886100 -0.58841800 0.09663600

C 2.30553100 0.27910300 -0.92819300

C 3.68605400 0.37165700 -1.10931400

C 1.30466600 0.93642500 -1.87926400

H 0.98101200 0.13431100 -2.55294900

C -0.01567300 1.56838000 -1.29599000

H -0.86111700 1.09456300 -1.79671800

C 0.08924400 3.06720100 -1.67936700

H -0.47980100 3.74278300 -1.04416100

C -0.28505200 3.17194800 -3.17844100

H -0.33469500 4.22341600 -3.48685000

H -1.25701800 2.71781400 -3.39625200

C 0.90201300 2.43493000 -3.87228200

H 1.38940700 3.07497500 -4.61526200

H 0.58407400 1.51962900 -4.38247400

C 1.86934400 2.12641900 -2.69984000

H 2.89758800 1.99740100 -3.04186600

C 1.61134800 3.29973100 -1.73417400

H 1.88566500 4.27778700 -2.14592200

H 2.10428900 3.16580200 -0.76500200

C -1.13507200 0.97965800 4.46619000

C -2.48362400 0.78811900 4.32340000

C -3.01669000 0.61712100 5.64030400

H -3.01932100 0.80452100 3.38527900

C -1.95515000 0.71319800 6.49144400

H -4.04793000 0.44862500 5.91523100

H -1.85615600 0.64697300 7.56448700

O -0.80314400 0.93382100 5.80183200

C 1.53239000 0.27499600 3.84528200

C 2.87421000 0.53082100 3.91455100

C 3.49627300 -0.66869700 4.39180900

H 3.35637100 1.46204100 3.65611000

C 2.49374800 -1.57074800 4.57818300

H 4.54892600 -0.83030600 4.57425600

H 2.45975100 -2.58862100 4.93481600

O 1.28868600 -1.01539200 4.24910100

C 0.79081800 2.92111200 3.52368400

C 1.00831000 3.98006500 2.68221400

C 1.58307100 5.02402200 3.47413100

H 0.76549000 4.00755900 1.63022900

C 1.67302400 4.53018300 4.74212400

H 1.88077300 6.00845100 3.14331200

H 2.02735100 4.94056600 5.67571200

O 1.20185800 3.25499400 4.79602200

I -2.45658200 3.10228400 1.03749300

N 0.43031300 -0.66303200 0.26435800

H -0.00741500 -1.54079800 -1.29410800

C -0.05902500 -1.68489900 1.18314700

H 0.28632900 -1.58145200 2.21860500

H -1.15294700 -1.65794700 1.20513200

H 0.23649600 -2.68606500 0.83174800

H 4.08233700 1.03401300 -1.87124000

C -0.46503100 -1.97232200 -3.18968300

O -0.01330600 -2.64648600 -4.13928900

O 0.12994200 -2.27469400 -1.94731300

O -1.34650000 -1.07448600 -3.18218900

Cs -3.34555100 -0.44147300 -1.04593500

Cs 2.91964100 -2.98101300 -2.99744300

**TS2(a)** (unsubstituted NBE)

M06-2X single point: -2071.870446 Hartree

B3LYP-D3 optimization: -1735.886505 Hartree

B3LYP-D3 thermal correction to Gibbs free energy: 0.40482 Hartree

Imaginary frequency: -294.4792 cm^-1^

Pd -0.19297600 1.23646400 1.06366600

P 0.36172500 1.58308300 3.22546000

C 4.59409600 -0.41098700 -0.38246300

H 5.67303500 -0.31125600 -0.45577000

C 4.02203500 -1.25117800 0.57639700

H 4.65946700 -1.81357400 1.25442400

C 2.62987500 -1.36763400 0.70239300

H 2.20267100 -2.00222500 1.46996700

C 1.79732800 -0.60766300 -0.13876800

C 2.37637600 0.22505900 -1.12897900

C 3.75409300 0.32365800 -1.24502500

C 1.33403600 0.86374900 -2.02682100

H 1.19460300 0.22383500 -2.90549800

C -0.01855100 0.94324200 -1.28534900

H -0.83984700 0.41826700 -1.75265200

C -0.27904700 2.47832200 -1.17923000

H -0.98292000 2.82415500 -0.41161500

C -0.77581500 2.84682000 -2.61479900

H -1.15982900 3.87213500 -2.59937800

H -1.58747300 2.19879700 -2.95589300

C 0.51871400 2.72936500 -3.48018800

H 0.76795000 3.68638500 -3.94862100

H 0.42259300 1.98202200 -4.27362700

C 1.59646800 2.33127000 -2.44222100

H 2.62158300 2.51331300 -2.77344100

C 1.13677500 3.09591600 -1.18857400

H 1.11756600 4.18260800 -1.32571500

H 1.72460700 2.85438000 -0.29905800

C -0.96499300 1.53592600 4.47363500

C -2.31986700 1.46045500 4.28806800

C -2.91017400 1.44069800 5.59241100

H -2.82062000 1.41605100 3.32940000

C -1.87512200 1.50047100 6.47822700

H -3.96184800 1.38585700 5.83459500

H -1.82062900 1.50913800 7.55664900

O -0.68245100 1.55787600 5.82346500

C 1.55426600 0.36517200 3.91481700

C 2.83459400 0.43622400 4.39266500

C 3.25248400 -0.91090300 4.65426900

H 3.39960700 1.34230600 4.55126100

C 2.19976100 -1.70780100 4.32169500

H 4.20581800 -1.23543100 5.04662600

H 2.02783500 -2.77266100 4.35743700

O 1.15854800 -0.95148000 3.86377700

C 1.25883200 3.12942700 3.59457900

C 1.55099500 4.17530800 2.76020400

C 2.24886500 5.14961800 3.54301300

H 1.28988000 4.22974900 1.71328400

C 2.33348300 4.63023900 4.80069700

H 2.63240400 6.10488600 3.21464900

H 2.76169700 4.98839600 5.72487100

O 1.73971700 3.40587400 4.85640100

I -3.70661200 0.95000200 0.33805200

N 0.41469200 -0.62602400 -0.17459200

H 0.26293400 -1.71526800 -1.76107100

C -0.33449200 -1.58664600 0.63678300

H -0.09167400 -1.53407800 1.70563300

H -1.40300100 -1.38786800 0.52535000

H -0.12630600 -2.60789200 0.27546900

H 4.18950900 0.96751300 -2.00530300

C 0.24331500 -2.14301600 -3.68486000

O 0.83112600 -2.86831000 -4.51060800

O 0.53009000 -2.46435600 -2.33754100

O -0.54752800 -1.17962900 -3.86034400

Cs -3.32189700 -1.49195100 -2.74633200

Cs 3.45487900 -3.31934400 -2.82740100

**1a (by-product)** (unsubstituted NBE)

M06-2X single point: -2071.960369 Hartree

B3LYP-D3 optimization: -1735.980983 Hartree

B3LYP-D3 thermal correction to Gibbs free energy: 0.408492 Hartree

Pd 1.55794200 0.36505100 1.84685800

P 0.94614300 1.32167700 3.74014000

C 4.22172700 -2.58211400 -2.36825600

H 4.97977000 -3.11860100 -2.93126700

C 3.85857600 -3.02555000 -1.08967900

H 4.33695700 -3.90435300 -0.66737200

C 2.89226200 -2.34442100 -0.34283900

H 2.61794600 -2.67574600 0.65413300

C 2.29353300 -1.21718700 -0.90573100

C 2.65048200 -0.76085600 -2.17904100

C 3.62132500 -1.44140500 -2.91465400

C 1.85942200 0.46569200 -2.54479700

H 1.25777200 0.32769900 -3.44771900

C 0.95594500 0.69016500 -1.28109100

H -0.10531600 0.66944400 -1.50962500

C 1.37356900 2.09698300 -0.81347300

H 1.09908500 2.29184500 0.22605300

C 0.79458000 3.09818000 -1.84720400

H 0.85736600 4.12094700 -1.46082800

H -0.25068300 2.87870400 -2.08568400

C 1.72499700 2.90173000 -3.09024900

H 2.28368600 3.81491700 -3.31924000

H 1.16233400 2.61862700 -3.98466500

C 2.68394000 1.78120800 -2.62694600

H 3.59137700 1.68234800 -3.22882800

C 2.88026500 2.12921600 -1.13641000

H 3.33187000 3.11475800 -0.98094000

H 3.45792900 1.38500200 -0.57905400

C -0.61489700 0.62726300 4.39608700

C -1.47371300 -0.29119200 3.84821600

C -2.57250900 -0.42469400 4.75581700

H -1.34589300 -0.81379000 2.91133200

C -2.30231600 0.41065000 5.79936600

H -3.43630300 -1.06242400 4.63657700

H -2.81829500 0.64187600 6.71927600

O -1.12037600 1.05974300 5.60316000

C 1.95660100 1.48155200 5.26132000

C 2.12719700 2.46866200 6.19499800

C 3.08701900 1.97997200 7.13868300

H 1.61746900 3.42099000 6.20636300

C 3.43126600 0.73064900 6.71572700

H 3.46708100 2.49059800 8.01198700

H 4.10714300 -0.02220600 7.09182100

O 2.76013000 0.41066300 5.57461100

C 0.48345900 3.08725000 3.50560300

C 0.87268600 3.98906200 2.54854600

C 0.16678000 5.20862900 2.81041800

H 1.58166000 3.79578100 1.75727600

C -0.60731600 4.96786900 3.90782000

H 0.23089400 6.13574800 2.25872400

H -1.30605500 5.56679000 4.47246000

O -0.42794700 3.68982600 4.34624800

I -3.78788700 -1.63993800 0.74226600

N 1.24453400 -0.43365500 -0.30560700

H -2.88578400 -0.91975000 -1.75591700

C 0.02515500 -1.26398900 -0.07769300

H 0.27287000 -2.12837800 0.53830200

H -0.73226000 -0.68963600 0.44957800

H -0.42280600 -1.59910100 -1.02070500

H 3.90702300 -1.08598100 -3.90159300

C -1.88935900 0.25987700 -2.95920800

O -1.15878500 0.23101400 -3.97392200

O -2.39543000 -1.00211200 -2.59662100

O -2.19185400 1.22223700 -2.20810500

Cs -2.38013800 2.20089500 0.69201500

Cs -0.21259900 -2.76614700 -3.98813700

**IV*** (5,6-DiCOOiPr NBE)

M06-2X single point: -2971.44390634 Hartree

B3LYP-D3 optimization: -2635.63336334 Hartree

B3LYP-D3 thermal correction to Gibbs free energy: 0.701993 Hartree

Pd -0.00937600 0.07184900 0.27734600

P 0.32591300 0.30979500 2.73634700

C 4.94701800 -0.99427700 -0.96262200

H 5.97480400 -0.76430900 -1.22568100

C 4.65909800 -1.73247200 0.17357100

H 5.45847300 -2.09249700 0.81706200

C 3.33458200 -2.00798700 0.49426800

H 3.12187100 -2.57186900 1.39187500

C 2.26867100 -1.53252800 -0.30280400

C 2.55299600 -0.79447300 -1.50597900

C 3.90937700 -0.55111900 -1.80682400

C 1.35381400 -0.50524600 -2.43577600

H 1.20813600 -1.46710400 -2.94679200

C -0.05320600 -0.12100700 -1.81266600

H -0.76757200 -0.92756000 -1.96236800

C -0.46032300 1.15440100 -2.57964700

H -1.23044700 1.74363100 -2.09070700

C -0.82057200 0.87025500 -4.06051900

H -1.08825200 1.82683800 -4.52903700

C 0.55286500 0.44460400 -4.68685500

H 0.84635200 1.14767600 -5.47107800

C 1.53798300 0.63293100 -3.48367000

H 2.55986400 0.73765600 -3.81951500

C 0.90334100 1.83951300 -2.77166300

H 0.87371500 2.74641500 -3.38734300

H 1.39183600 2.06468200 -1.81917200

C -1.20398300 -0.06596200 3.63486500

C -2.24692400 -0.84658100 3.21155100

C -3.17047700 -0.90817700 4.30447800

H -2.32293500 -1.32981200 2.24536900

C -2.62535300 -0.16206100 5.30853100

H -4.11284600 -1.43652600 4.33733000

H -2.95127400 0.09463900 6.30531000

O -1.42470300 0.35323000 4.92649700

C 1.56603600 -0.66642300 3.68114300

C 2.93418700 -0.64115400 3.65797600

C 3.37766100 -1.74260700 4.45934200

H 3.54656000 0.06263100 3.11352500

C 2.25132000 -2.36365500 4.90957800

H 4.39810100 -2.02975700 4.66912000

H 2.07202800 -3.21732000 5.54479200

O 1.13811000 -1.72163400 4.44858600

C 0.87719000 1.96718900 3.24304900

C 1.37874200 2.99525700 2.49141000

C 1.81355100 4.00321100 3.40902200

H 1.40140000 3.02576800 1.41251800

C 1.54944900 3.51667100 4.65572000

H 2.25465900 4.96045100 3.17142300

H 1.68630000 3.91086700 5.65139400

O 0.98978200 2.27898800 4.57982800

I -1.71811600 2.36480600 0.55047500

N 0.94224500 -1.78867100 0.03507700

H -0.40656600 -2.74402400 -0.90193600

C 0.72337200 -2.71803200 1.14453200

H 1.19873600 -2.43071600 2.08746900

H -0.35126100 -2.80909800 1.30594300

H 1.11696800 -3.71228800 0.87750800

C 0.63606700 -0.93158900 -5.28863500

O -0.12880200 -1.85786500 -5.05983200

O 1.69988400 -1.04761100 -6.10000100

C 1.92445100 -2.34428400 -6.73958200

C 3.39228900 -2.37390900 -7.13145500

C 0.98138900 -2.48592800 -7.92931800

H 1.70901200 -3.11788900 -5.99586200

H 4.03274900 -2.29130800 -6.25147800

H 3.62024000 -3.31696500 -7.63704100

H 3.62592900 -1.54974700 -7.81213100

H -0.05899500 -2.44061500 -7.60145800

H 1.15960700 -1.68371000 -8.65214900

H 1.14934500 -3.44579500 -8.42768100

C -2.02240700 0.00132100 -4.34344500

O -2.72263800 -0.57193100 -3.52369900

O -2.30079600 0.00977700 -5.66514100

C -3.49468900 -0.68192700 -6.12427500

C -3.18024000 -1.18640300 -7.52392700

C -4.67264700 0.28367700 -6.06640700

H -3.68464000 -1.51871800 -5.44623900

H -2.31571100 -1.85561100 -7.50504800

H -4.03800500 -1.73095200 -7.93020700

H -2.95274300 -0.34999900 -8.19189600

H -4.83335100 0.62376300 -5.03976700

H -4.48184400 1.15535800 -6.69992900

H -5.58533400 -0.20898500 -6.41605900

C -2.34037500 -3.08003700 -0.76945100

O -3.31184500 -3.49344400 -1.45595000

O -1.09170100 -3.30069200 -1.34930500

O -2.37684000 -2.47792200 0.33241600

Cs -4.34101100 -0.38513400 -0.75696400

Cs -1.98955100 -4.08906200 -4.17124500

C 4.97295000 1.43181000 -2.77663900

C 5.08395400 -0.67924700 -3.95057400

C 5.16609100 2.14532500 -4.11392500

H 5.95538700 1.32596200 -2.28141800

H 4.33224300 2.03506800 -2.12459800

C 5.25635400 0.09601900 -5.25597600

H 6.08241300 -0.95603000 -3.56499200

H 4.52965600 -1.60640600 -4.13135900

H 5.73238800 3.07178500 -3.97932700

H 4.18225600 2.39535200 -4.54472700

H 5.88315900 -0.46109400 -5.95935700

H 4.26868400 0.25847200 -5.71720400

N 4.32429000 0.14048800 -3.00909500

O 5.90904700 1.34213900 -5.02790700

**IV** (5,6-DiCOOiPr NBE)

M06-2X single point: -2971.433185 Hartree

B3LYP-D3 optimization: -2635.632298 Hartree

B3LYP-D3 thermal correction to Gibbs free energy: 0.698745 Hartree

Pd 0.63714900 -0.00107200 1.38772900

P 0.39670900 0.39445100 3.79776800

C 5.64549800 -1.23940900 0.07080900

H 6.69117900 -1.13937000 -0.19224800

C 5.28847600 -1.92309000 1.22310800

H 6.05628100 -2.33922100 1.87035400

C 3.94672700 -2.09856800 1.54289100

H 3.68782300 -2.65324000 2.43518400

C 2.92552400 -1.55736500 0.72846100

C 3.28535900 -0.79026400 -0.43096000

C 4.65389100 -0.69965800 -0.77393700

C 2.14640500 -0.24715600 -1.31336400

H 1.95806400 -1.04645800 -2.03744300

C 0.79105300 0.09406100 -0.62365800

H -0.03684200 -0.52051200 -0.96842100

C 0.54949100 1.58768200 -0.87534400

H -0.10759100 2.09998300 -0.16297100

C 0.04597300 1.77628500 -2.32363900

H -0.10191100 2.84922700 -2.49450000

C 1.28483700 1.30629900 -3.16990600

H 1.61337000 2.10223000 -3.84005500

C 2.39719200 1.09318600 -2.07092600

H 3.39947600 1.19374500 -2.46680300

C 1.98217500 2.12752700 -1.01047600

H 2.02787200 3.16397000 -1.35858200

H 2.57051300 2.02893300 -0.09283100

C -0.41595600 -0.80523100 4.89193800

C -1.34203500 -1.77553500 4.61225000

C -1.66201200 -2.39593700 5.86303400

H -1.77048400 -2.01453200 3.64489300

C -0.90612600 -1.76803400 6.80840500

H -2.35840700 -3.20543800 6.02912100

H -0.79304400 -1.89275400 7.87481000

O -0.14285900 -0.79405600 6.24221800

C 2.02865200 0.65731600 4.54978400

C 3.27352100 0.41858600 4.02823400

C 4.21707300 0.82056000 5.02600900

H 3.48333900 -0.00458600 3.05576800

C 3.48182400 1.28165800 6.07811500

H 5.29475300 0.77619800 4.96382100

H 3.74114700 1.69229100 7.04227500

O 2.15045300 1.19176300 5.81143200

C -0.50992100 1.94170200 4.10444100

C -0.86873200 2.93733200 3.23198900

C -1.59305400 3.90919800 3.99278900

H -0.65983400 2.99123800 2.17150300

C -1.62632000 3.43842200 5.27215500

H -2.02907000 4.82435600 3.61972100

H -2.05590400 3.80946900 6.19043100

O -0.97376600 2.24468800 5.36407600

I -2.19656000 4.40135200 -0.28587600

N 1.57560000 -1.81020700 0.99908700

H -1.89130700 -4.33273900 2.04167700

C 1.28345900 -2.72779600 2.09551600

H 1.61381400 -2.38356200 3.08936000

H 0.20647600 -2.89662600 2.13790800

H 1.76362700 -3.70189000 1.90869900

C 1.03656300 0.07530700 -4.01368000

O 0.32271900 -0.86610100 -3.69402000

O 1.72702200 0.11030200 -5.16322700

C 1.50973300 -0.97926300 -6.11819900

C 2.30230700 -2.21486200 -5.70784700

C 1.91992700 -0.41833900 -7.46996100

H 0.43956600 -1.20785000 -6.10149000

H 2.02450000 -2.53552300 -4.70211200

H 2.09223800 -3.03329800 -6.40350400

H 3.37631600 -2.01461300 -5.73275300

H 1.33323700 0.47134800 -7.71294900

H 2.98013400 -0.14698100 -7.46686800

H 1.75637600 -1.16840900 -8.24935100

C -1.29527400 1.16205700 -2.65255300

O -2.03962000 0.61809000 -1.85654000

O -1.60021200 1.36124900 -3.94508900

C -2.89256000 0.87602900 -4.42300600

C -2.83146900 -0.63305800 -4.63283000

C -3.17059600 1.65670200 -5.69753200

H -3.63458700 1.11538000 -3.65489300

H -2.62111500 -1.13963900 -3.68947800

H -3.79051000 -0.99155600 -5.02017500

H -2.04516700 -0.88490100 -5.35071900

H -3.17738600 2.73152800 -5.49806500

H -2.40416700 1.44613200 -6.45070600

H -4.14474500 1.37080400 -6.10524200

C -2.57061700 -2.86609300 1.01783500

O -2.58423300 -2.36802900 -0.12886300

O -1.81790200 -4.05792600 1.11647400

O -3.09629500 -2.45910300 2.08049300

Cs -3.31607500 0.58825900 1.02051200

Cs -0.10430200 -3.41931000 -1.62763400

C 6.37125500 0.58447100 -2.00587300

C 4.91009800 -0.92538200 -3.19111900

C 6.50406200 1.40668800 -3.28446200

H 7.21367600 -0.12956800 -1.97803100

H 6.44535300 1.23361900 -1.12816100

C 5.13683500 -0.09389600 -4.45592600

H 5.62129600 -1.77130100 -3.17291200

H 3.89914900 -1.34212100 -3.21064900

H 7.48815500 1.88156200 -3.33513900

H 5.73061100 2.19174000 -3.30672500

H 5.14779000 -0.74125000 -5.33752400

H 4.31808700 0.63246100 -4.57432400

N 5.06863100 -0.08114300 -2.00072500

O 6.39441500 0.57047900 -4.43254100

**TS3** (5,6-DiCOOiPr NBE)

M06-2X single point: -2971.40363 Hartree

B3LYP-D3 optimization: -2635.600379 Hartree

B3LYP-D3 thermal correction to Gibbs free energy: 0.696211 Hartree

Imaginary frequency: -308.0299 cm^-1^

Pd 0.78250300 0.06123600 1.63603100

P 0.46333600 0.77066900 3.79454400

C 5.73007600 -1.30085700 0.08007400

H 6.79301500 -1.18929500 -0.09583800

C 5.28945400 -2.02155000 1.18723400

H 6.01943000 -2.45533400 1.86593800

C 3.92856500 -2.20105500 1.43976000

H 3.59270900 -2.75976400 2.30637700

C 2.99477000 -1.61211200 0.56799200

C 3.43170300 -0.89074300 -0.56601600

C 4.80059000 -0.74973600 -0.82969900

C 2.26170600 -0.46989800 -1.44553000

H 2.13601600 -1.25651200 -2.20004500

C 0.95470400 -0.38202400 -0.62261300

H 0.14582000 -1.02528800 -0.93254800

C 0.55878600 1.13465900 -0.72627300

H -0.10969000 1.55832100 0.03112500

C -0.04980200 1.35365800 -2.13933000

H -0.32222400 2.41380100 -2.19042800

C 1.16670700 1.12036400 -3.10152200

H 1.35506800 2.00922900 -3.70701100

C 2.35367600 0.93561100 -2.09143900

H 3.32770500 1.14888200 -2.52031600

C 1.91613000 1.83305500 -0.92175200

H 1.81584100 2.88916500 -1.18859700

H 2.58177800 1.74189300 -0.05945600

C -0.52948200 -0.32930100 4.85744400

C -1.17541500 -1.50057000 4.55363800

C -1.80553900 -1.94259700 5.76226700

H -1.22162200 -1.98928100 3.58976700

C -1.49629600 -1.01711200 6.71465800

H -2.40526000 -2.83134200 5.89715700

H -1.73491400 -0.92004600 7.76323700

O -0.72257700 -0.02810000 6.18874600

C 2.05989500 0.88668600 4.66834000

C 3.29863800 0.46299800 4.25966500

C 4.20454100 0.75405700 5.32888700

H 3.52384300 -0.00827100 3.31226300

C 3.45653700 1.33447500 6.31027300

H 5.26721300 0.56089500 5.35957100

H 3.69018200 1.72683100 7.28858900

O 2.15176700 1.42275500 5.93214400

C -0.31404600 2.39877900 4.03569700

C -0.82380600 3.25603200 3.09426600

C -1.36069800 4.37590300 3.80532900

H -0.83217000 3.11410100 2.02211700

C -1.13984100 4.12457700 5.12721800

H -1.84369000 5.24351600 3.37975500

H -1.35816600 4.66899200 6.03377300

O -0.50752400 2.92931000 5.29228200

I -2.61496700 3.73420100 -0.53606100

N 1.62420200 -1.71091700 0.70364500

H -1.48273500 -4.77881200 1.36782100

C 1.01976000 -2.83276900 1.40292600

H 1.20502100 -2.82104100 2.48865100

H -0.06271700 -2.81502300 1.26961500

H 1.40543800 -3.78281600 0.99682000

C 1.02800600 -0.05067800 -4.04766500

O 0.29258900 -1.01387900 -3.87331500

O 1.84607400 0.06435500 -5.10014900

C 1.76088900 -0.96123000 -6.14687000

C 2.48917400 -2.22871800 -5.71536800

C 2.35155400 -0.31535000 -7.38927400

H 0.69766600 -1.17793000 -6.29051300

H 2.07302000 -2.61842700 -4.78425100

H 2.38029900 -2.99319600 -6.49101800

H 3.55620900 -2.03739600 -5.57645000

H 1.80572700 0.59522300 -7.64921200

H 3.40211000 -0.05705200 -7.22490700

H 2.29364600 -1.01033200 -8.23201900

C -1.34159100 0.63788700 -2.45361000

O -1.91017500 -0.15268000 -1.71303200

O -1.80370600 1.02637600 -3.64540800

C -3.06386300 0.47476100 -4.13957100

C -2.75630200 -0.16935700 -5.48487700

C -4.06205900 1.62118000 -4.20719200

H -3.40130800 -0.27629600 -3.42148900

H -1.98548100 -0.93753100 -5.37468800

H -3.66037800 -0.62605700 -5.89937900

H -2.39255100 0.58185900 -6.19335000

H -4.18100900 2.07998300 -3.22163900

H -3.71543500 2.38988200 -4.90503000

H -5.03391900 1.25180600 -4.55002700

C -2.65604600 -3.35435200 0.86861200

O -3.29385100 -2.87414100 -0.09365500

O -1.91094800 -4.51342600 0.54122200

O -2.54080200 -2.94926900 2.04797900

Cs -3.26243700 0.11013600 1.30384300

Cs -1.26502900 -3.35679400 -2.42417000

C 6.56633600 0.53903800 -1.97268400

C 5.06586500 -0.84324800 -3.25427400

C 6.73361900 1.43368500 -3.19784800

H 7.37788700 -0.21012200 -1.98096600

H 6.65905400 1.13281100 -1.05833000

C 5.31136800 0.06287600 -4.46097000

H 5.76122000 -1.70092600 -3.28881800

H 4.04897500 -1.23867600 -3.30294800

H 7.73586200 1.87106900 -3.22004600

H 5.99152300 2.24795800 -3.16501600

H 5.29477600 -0.52196300 -5.38559900

H 4.51568000 0.82232200 -4.52048100

N 5.24002100 -0.07693600 -2.01298400

O 6.58996600 0.68222100 -4.39996900

**2b (product)** (5,6-DiCOOiPr NBE)

M06-2X single point: -2971.504102 Hartree

B3LYP-D3 optimization: -2635.692435 Hartree

B3LYP-D3 thermal correction to Gibbs free energy: 0.701134 Hartree

Pd 1.21180500 -0.08089200 2.44999500

P 0.42841200 0.77114000 4.33972800

C 6.01102600 -1.25575100 -0.03833800

H 7.08037200 -1.24191200 -0.21374400

C 5.53865800 -1.55294800 1.24473700

H 6.25245100 -1.76075500 2.03654200

C 4.17327500 -1.58004500 1.52460300

H 3.79788500 -1.77542300 2.52381600

C 3.29761600 -1.29692800 0.47628900

C 3.74211600 -1.01642200 -0.81273400

C 5.12231600 -0.97689900 -1.09189700

C 2.57495800 -0.67033400 -1.70401800

H 2.44889100 -1.38946100 -2.51491100

C 1.36193400 -0.66935500 -0.71142400

H 0.52610800 -1.27752500 -1.04334000

C 0.94822000 0.82144300 -0.66375200

H 0.40495200 1.08918500 0.24459000

C 0.21217600 1.20708200 -1.96397400

H -0.06378300 2.26386200 -1.88123200

C 1.32585700 1.08624700 -3.07282400

H 1.45384500 2.03754000 -3.59131800

C 2.60379300 0.79408200 -2.21299700

H 3.53429000 1.04909000 -2.71973100

C 2.27889900 1.56164700 -0.91177400

H 2.14507900 2.63697500 -1.05940500

H 3.02080700 1.40447000 -0.12597500

C -0.68461300 -0.35942700 5.25139500

C -1.20444900 -1.57206800 4.87399700

C -2.05509700 -2.00259800 5.94378200

H -1.02193200 -2.08937500 3.94179900

C -1.98916500 -1.02984700 6.89729200

H -2.63042400 -2.91606500 5.99347800

H -2.44031700 -0.91084600 7.87110400

O -1.16515800 -0.02283800 6.49829700

C 1.67194000 1.23484500 5.59392100

C 3.03428000 1.09629000 5.54948500

C 3.54406100 1.62421200 6.77838100

H 3.59025000 0.66961500 4.72675400

C 2.45704400 2.04998400 7.48298800

H 4.57643200 1.68197700 7.09211100

H 2.33458300 2.51558800 8.44936800

O 1.31129900 1.82365600 6.78393900

C -0.59638700 2.27431900 4.18096500

C -0.84317000 3.04473300 3.07389100

C -1.76327200 4.06548100 3.47355100

H -0.43544700 2.89742800 2.08440700

C -2.01147800 3.84926800 4.79659100

H -2.18465600 4.83272400 2.84093900

H -2.63088400 4.34655800 5.52803600

O -1.31524200 2.76696800 5.24844800

I -2.48369900 3.65300800 -0.50324200

N 1.85497700 -1.23399200 0.59673800

H -0.87586800 -4.78578900 1.19259200

C 1.29472300 -2.59639100 0.82950500

H 1.75612200 -3.02459600 1.72044800

H 0.22276900 -2.53032200 1.00653400

H 1.50391800 -3.25707700 -0.02622600

C 1.04311900 0.03016500 -4.11963400

O 0.52609000 -1.05847400 -3.89219400

O 1.44213200 0.41565800 -5.33503500

C 1.18422800 -0.48558400 -6.46113700

C 2.30201600 -1.51601600 -6.54714900

C 1.07911000 0.40989900 -7.68453500

H 0.23064400 -0.98338400 -6.26545500

H 2.37154200 -2.08604800 -5.61713300

H 2.10474600 -2.21269700 -7.36776100

H 3.25951400 -1.02350200 -6.73577800

H 0.27633000 1.14145600 -7.55933600

H 2.01798700 0.94700000 -7.85035100

H 0.86482000 -0.19541300 -8.57026800

C -1.09874900 0.52767800 -2.26929700

O -1.64780100 -0.30844300 -1.56420100

O -1.59690600 0.99249800 -3.42021700

C -2.89240400 0.51705000 -3.90039300

C -2.63789500 -0.19476100 -5.22364600

C -3.79868300 1.73260800 -4.02147300

H -3.28775500 -0.17768100 -3.15530400

H -1.93174000 -1.01947700 -5.09535700

H -3.57552400 -0.58752900 -5.62855300

H -2.21127200 0.50234200 -5.95218700

H -3.88078700 2.24232800 -3.05791400

H -3.39329700 2.43965000 -4.75229300

H -4.79486100 1.42197400 -4.35286000

C -2.20513400 -3.44807800 0.87949100

O -3.00514000 -3.01327500 0.02331200

O -1.42775000 -4.54733800 0.43389100

O -1.95200900 -3.04081900 2.03636700

Cs -2.84238700 -0.00044400 1.47300300

Cs -1.34675600 -3.39135000 -2.60499900

C 6.93571500 -0.10684500 -2.51498000

C 5.25401900 -1.54187700 -3.47682000

C 7.09410000 0.55886600 -3.88050700

H 7.68264700 -0.91524600 -2.42601900

H 7.12714200 0.62521300 -1.72485900

C 5.47480500 -0.85135600 -4.82026300

H 5.89584300 -2.43809800 -3.41581100

H 4.21595600 -1.87073900 -3.40431500

H 8.12649500 0.88825400 -4.02761600

H 6.42974000 1.43641800 -3.93789400

H 5.33520200 -1.56238600 -5.63935300

H 4.74313200 -0.03377500 -4.93305100

N 5.56649400 -0.60714900 -2.38688700

O 6.80076400 -0.35140100 -4.93572900

**Int_I** (DavePhos+5,6-DiCOOiPr NBE)

B3LYP-D3 optimization: -2727.97232206 Hartree

Pd -0.48944900 -0.51172700 0.45095800

P -0.61816700 -0.61123100 3.05175100

C 4.24877600 1.12267000 -1.62101800

H 4.78245900 2.03260200 -1.88647100

C 4.87690000 0.10936200 -0.89634300

H 5.90513600 0.22451900 -0.56001000

C 4.19258400 -1.07608900 -0.62044500

H 4.70021100 -1.86696900 -0.07995200

C 2.87448500 -1.28491200 -1.08288100

C 2.21705400 -0.25072000 -1.82252500

C 2.93103800 0.92361900 -2.06655200

C 0.87313100 -0.56578100 -2.46971700

H 1.00452200 -1.57297900 -2.87701500

C -0.46531900 -0.58480800 -1.62248100

H -0.95630900 -1.53741100 -1.80779500

C -1.30933600 0.56748900 -2.22739900

H -2.00778000 1.02505300 -1.53342000

C -1.94412200 0.06228100 -3.54163600

H -2.52660400 0.88202100 -3.98835000

C -0.69651400 -0.16042900 -4.46844500

H -0.78171900 0.44735200 -5.37198000

C 0.48060100 0.42271700 -3.59810600

H 1.31805100 0.72392400 -4.23285500

C -0.24888900 1.52094500 -2.79662100

H -0.66538700 2.32035500 -3.42198000

H 0.36040900 1.95457500 -2.00467900

I -1.03938200 -3.23202900 0.56690400

N 2.24232700 -2.49414500 -0.90256900

H 1.23051300 -2.48190400 -0.86059600

C 2.85131400 -3.61211300 -0.21871400

H 3.16087400 -3.37949800 0.81065800

H 2.11596900 -4.41665800 -0.17047000

H 3.73445800 -3.96982800 -0.76279100

H 2.46587200 1.71053100 -2.64966600

C -0.42382300 -1.57898300 -4.91181500

O -0.60727400 -2.58315700 -4.25240200

O 0.13407900 -1.58893900 -6.14570500

C 0.53128400 -2.88214900 -6.67931800

C 1.64393800 -2.59468700 -7.67646100

C -0.68524900 -3.55996700 -7.30059700

H 0.90320900 -3.48734000 -5.84649200

H 2.47828500 -2.08537000 -7.18617600

H 2.01204400 -3.52949700 -8.11081100

H 1.27762100 -1.95648500 -8.48714000

H -1.45253500 -3.71945900 -6.54065300

H -1.10078700 -2.93624000 -8.09895400

H -0.40671800 -4.53027300 -7.72452400

C -2.96246500 -1.04426700 -3.37282000

O -3.51552800 -1.30864200 -2.32372700

O -3.27596100 -1.62761700 -4.54928100

C -4.21529100 -2.74057600 -4.50190300

C -3.53203200 -3.97577000 -3.92254900

C -4.69179200 -2.92418100 -5.93548000

H -5.04699500 -2.44251900 -3.85421600

H -3.21589900 -3.79189600 -2.89427400

H -4.22802800 -4.82155900 -3.93221000

H -2.64685200 -4.23237700 -4.50984800

H -5.14613600 -2.00447200 -6.31575800

H -3.85258900 -3.18809600 -6.58691400

H -5.43349200 -3.72740800 -5.98553200

C -0.25259900 0.86534500 4.19542700

C -0.91446900 2.17378400 3.71290800

C -0.52086400 0.69272900 5.70166000

C -0.86696100 3.28328200 4.79596700

H -0.41428600 2.52740500 2.81000800

C 0.24422400 1.77561200 6.49895500

H -1.59468100 0.78928800 5.87912300

C 0.34624400 3.10759700 5.71785100

H -0.83309000 4.27292000 4.31961300

H 1.25204100 1.41374400 6.73841600

H 1.25823800 3.11439800 5.10382900

C -2.09386300 -1.43650800 3.87481300

C -1.86165400 -2.59147600 4.64518600

C -3.43855200 -0.98262700 3.74851700

C -2.87493300 -3.25032500 5.33540000

H -0.86883900 -3.01390700 4.69267700

C -4.44370600 -1.65323300 4.47325800

C -4.17716100 -2.76113900 5.26826000

H -2.64390400 -4.14253700 5.91047000

H -5.46275000 -1.29707300 4.37810100

H -4.98565400 -3.25728500 5.79779800

C 0.89579700 -1.69463900 3.46933000

C 2.06187900 -1.17929600 2.58005400

C 1.41281900 -1.83685000 4.91496600

C 3.45283900 -1.61667700 3.09035500

H 1.90001900 -1.52123100 1.55555700

C 2.47787000 -2.95615000 4.99603700

H 1.85501200 -0.88489400 5.23497200

C 3.37451600 -3.00340400 3.73691700

H 4.16324200 -1.60321900 2.25732300

H 1.97901600 -3.92368000 5.12762700

H 2.95178100 -3.70533100 3.00830800

H 0.44129900 3.95203200 6.40973600

H -1.77476000 3.26440600 5.41230200

H -0.26066500 1.94371800 7.45790500

H -0.25613200 -0.29685300 6.06870300

H -1.95444100 1.96550400 3.43464100

H 0.83504700 0.96988100 4.07108600

H 0.61783300 -2.05466700 5.62956400

H 3.09045300 -2.79710000 5.89221000

H 4.37206600 -3.38027900 3.98826900

H 3.83274000 -0.90042700 3.83349800

H 2.02655100 -0.08526100 2.53318600

H 0.60546200 -2.68184100 3.09329800

C -3.94062200 0.03787800 2.76513700

C -4.88340700 1.05686900 3.10091700

C -3.67199500 -0.19409500 1.41019500

C -5.48830400 1.78522500 2.05964600

C -4.27760600 0.53319000 0.38507200

H -3.01345700 -1.01170400 1.14268400

C -5.20160800 1.51604000 0.71506200

H -6.22969100 2.54218700 2.29502000

H -4.04119400 0.28479400 -0.64223700

H -5.71558900 2.07533100 -0.06399000

N -5.28293700 1.27026300 4.45082200

C -4.24099500 1.65248800 5.38702600

H -3.95937900 2.71963300 5.28777700

H -4.58002000 1.48811900 6.41547600

H -3.35394200 1.04608100 5.22553400

C -6.52087400 1.98718300 4.68715400

H -6.80554000 1.86133300 5.73685000

H -6.45104600 3.07666000 4.49450600

H -7.32116000 1.58000800 4.06453800

C -0.60318000 2.50625200 0.44978700

O -1.81554600 2.40132700 0.10540100

O 0.21533500 1.45702100 0.41742800

O -0.08094200 3.61163300 0.88992500

Cs -2.94513000 4.54314300 1.63030700

Cs 2.75118700 2.52490300 1.31720000

broblasts expressing human CYP11B1 with deoxycorticosterone (100 nM) as the substrate;
